# Association of endometriosis with hematuria markers: A Mendelian randomization study

**DOI:** 10.1097/MD.0000000000045026

**Published:** 2026-02-20

**Authors:** Shu-ping Huang, Ze-chao Zhang, Jing Li, Liang-ying Li, Wen-jia Ding, Cai-ying Xie, Yi-ting Zhang, Wei-hong Li

**Affiliations:** a Department of Graduate School, Guangxi University of Chinese Medicine, Nanning, Guangxi Zhuang Autonomous Region, China; b Department of Yao College of Medicine, Guangxi University of Chinese Medicine, Nanning, Guangxi Zhuang Autonomous Region, China.

**Keywords:** blood and urine biomarkers, endometriosis, Mendelian randomization

## Abstract

Identification of appropriate biomarkers is of great clinical significance for early diagnosis of endometriosis (EMs). This study aimed to comprehensively analyze the association between EMs and blood and urine biomarkers (BUB). Candidate EMs-related single nucleotide polymorphisms were obtained from recent genome-wide association studies. The UK Biobank cohort included 3,63,228 participants with BUB test data to calculate the polygenic risk score for EMs. Two-way, 2-sample Mendelian randomization (MR) was used to assess the potential relationship between candidate BUB and EMs. We found a significant association between EMs and triglycerides and calcium in both forward and reverse MR (*r* > 1, *P* < .05). In the positive MR analysis, we also found that insulin-like growth factor-1 was a risk factor for EMs, and high-density lipoprotein was a protective factor for EMs. Our findings provide insights into the role of BUB in the early detection and treatment of EMs.

## 1. Introduction

Endometriosis (EMs) is a chronic inflammatory disease characterized by pain and infertility. It significantly affects patients’ daily lives, leading to serious social, economic, and emotional burdens.^[1]^ EMs appear to be one of the most common benign gynecological proliferations in premenopausal women. It is estimated that 10–15% of women of reproductive age suffer from pelvic endometriosis, but their biological significance is unknown.^[2]^ Despite its high prevalence, the disease remains poorly understood. The present study proves that there is no relationship between the extent of the disease and its symptoms, and no blood tests are currently available for the diagnosis of EMs.^[3]^ Blood and urine biomarkers (BUB) are readily accessible in clinical practice and have the potential to serve as diagnostic and therapeutic targets for EMs. Owing to the large number of BUBs, their exact role in EMs is unknown. This effect may occur via genetic pathways, and there may be specific exposure–outcome associations.

In view of these issues, Mendelian randomization (MR) offers a novel analytical approach to elucidate the relationship between EMs and BUB. MR uses single nucleotide polymorphisms (SNPs) as instrumental variables (IV) to infer causality in the observed associations between exposure or risk factors and clinically relevant outcomes. This approach offers the advantage of minimizing confounding and reducing bias from reverse causality.^[4,5]^ Owing to the fruitful findings of large-scale genome-wide association studies (GWAS) conducted at the BUB and disease level to date, MR analysis has been widely used in a variety of scenarios. The MR method was utilized to investigate the relationship between EMs and BUB. This study aimed to elucidate the specific mode of action between them and offer a new research direction for the diagnosis and treatment of EMs. In this study, a bidirectional MR design was used to investigate potential associations between BUB and EMs. The bidirectional MR design allowed for the exploration of not only whether BUB could influence the development of EMs but also if EMs could have an impact on the levels of BUB. First, a large number of SNPs were carefully selected as IVs for both BUB and EMs. These SNPs were chosen based on their strong associations with either BUB or EMs, as identified from previous GWAS.

The first direction of the bidirectional MR focused on determining if BUB had a causal effect on EMs. By using the selected SNPs related to BUB as IVs, the analysis estimated the potential causal relationship between the levels of BUB and the risk of developing EMs. This will not only help in improving the diagnosis and treatment of EMs but also in reducing the social, economic, and emotional burdens associated with this chronic disease.

## 2. Materials and methods

### 2.1. Research design description

**Figure 1.**
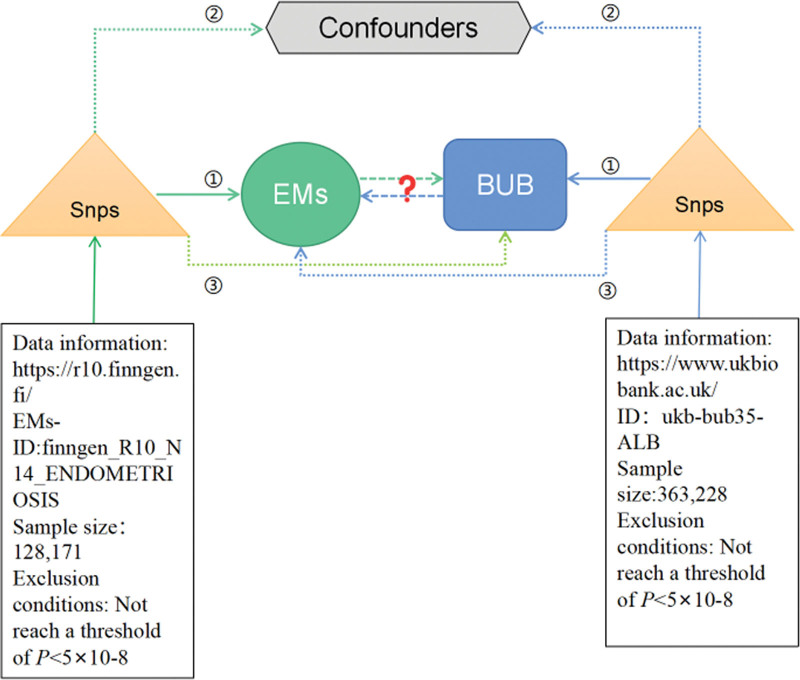
Flow chart of the bidirectional MR study. The MR analysis depends on 3 core assumptions: ①②③. Blue represents forward MR analysis, BUB represents exposure, and EMs represents outcome. Green represents reverse MR analysis, EMs for exposure, and BUB for outcome. BUB = blood and urine biomarkers, EMs = endometriosis, MR = Mendelian randomization. SNP = single nucleotide polymorphism.

Figure 1 outlines the key steps of a bidirectional MR study that investigates the interplay between BUB and EMs. This study involved 2 MR analyses using summary statistical data from GWAS to unveil potential associations between BUB and EMs. In the forward MR analysis, BUB was treated as the exposure variable, and EMs as the outcome. In reverse MR analysis, EMs are considered the exposure variable and BUB the outcome variable. The fundamental MR assumptions underpinning this study are shown in Figure 1. Given that this study relied on publicly available data, ethical approval was not required.

### 2.2. MR tool variable selection

The IVs for the MR analysis were extracted from 2 distinct GWAS summary results. Initially, a genome-wide significance threshold (*P* < 5 × 10^−8^) was employed.^[6]^ Subsequently, SNP independence was evaluated based on pairwise linkage disequilibrium analysis. SNPs demonstrating an *r*^2^ > 0.001 within a 10,000 kb window were excluded, effectively addressing associations with multiple SNPs and SNPs exhibiting higher *P*-values.^[7]^ Linkage disequilibrium signifies a nonrandom association between alleles at distinct loci. In essence, if 2 genes are not inherited independently, some level of linkage is evident. The parameter *r*^2^ ranges from 0 to 1, where *r*^2^ = 1 signifies complete linkage disequilibrium, and *r*^2^ = 0 denotes complete linkage equilibrium, indicating random allocation of the 2 SNPs. The linkage disequilibrium region length is denoted by “kb”. The threshold *r*^2^ = 0.001 and a 10,000 kb window were used to exclude SNPs with *r*^2^ exceeding 0.001 within 10,000 kb. Additionally, *F*-statistics were computed to gauge the strength of individual SNPs. SNPs with *F*-statistic values exceeding 10 were deemed to be sufficiently robust to mitigate potential bias.

### 2.3. EMs data source and tool variable selection

The EMs data originated from FINNGEN pipeline version 10 (available at https://r10.finngen.fi/). This dataset encompasses a population primarily diagnosed with EMs. GWAS data were leveraged to identify SNPs associated with EMs, which were subsequently selected as IVs (Table 1).

**Table 1:** Detailed information of included data sources.

Traits	Sample size	Year	Web source
Blood and urine biomarkers	3,63,228	2016	https://biobank.ndph.ox.ac.uk/
Endometriosis	1,28,171	2023	https://r10.finngen.fi/

### 2.4. BUB data source and tool variable selection

A summary-level GWAS dataset was acquired from the UK Biobank (available at https://biobank.ndph.ox.ac.uk; Tables 1 and S1, Supplemental Digital Content, https://links.lww.com/MD/R313).

### 2.5. MR statistical analysis

SNPs pertaining to BUB and EMs were employed in the subsequent forward and reverse MR analyses. The random-effects inverse variance weighted (IVW) method, encompassing the core MR assumptions, constitutes the primary statistical approach for estimating potential bidirectional causal associations between BUB and EMs.^[4]^ When multiple IVs are available, the IVW method is the most robust because it accounts for variant specificity and causal estimation heterogeneity. The IVW method further encompasses sensitivity analyses, including simple mode, weighted mode, weighted median, and MR-Egger regression, to assess the robustness of the research findings’ robustness.^[8]^ If IVs influence outcomes through alternate pathways indicative of potential pleiotropy, causal estimation via IVW might incur bias. The MR-Egger was used to assess pleiotropy. In MR-Egger, a *P*-value exceeding .05 MR. signifies the absence of level pleiotropy. Heterogeneity testing using MR heterogeneity was conducted to identify the SNP-induced heterogeneity. If heterogeneity was detected, a random-effects model was employed; otherwise, a fixed-effects model was assumed by default. Single SNPs were sequentially eliminated from MR analyses to assess their collective impacts.^[9]^ Twosamplemr (v.0.5.6) within the R package (v.4.3.0) facilitated major statistical analysis and graphical representation.^[10]^ Odds ratio (OR) and the accompanying 95% confidence interval (CI) gauged the extent of risk alteration for each additional standard deviation of the exposure factors. Statistical significance was set at *P* < .05.^[11]^

## 3. Results

### 3.1. EMs forward MR

**Figure 2.**
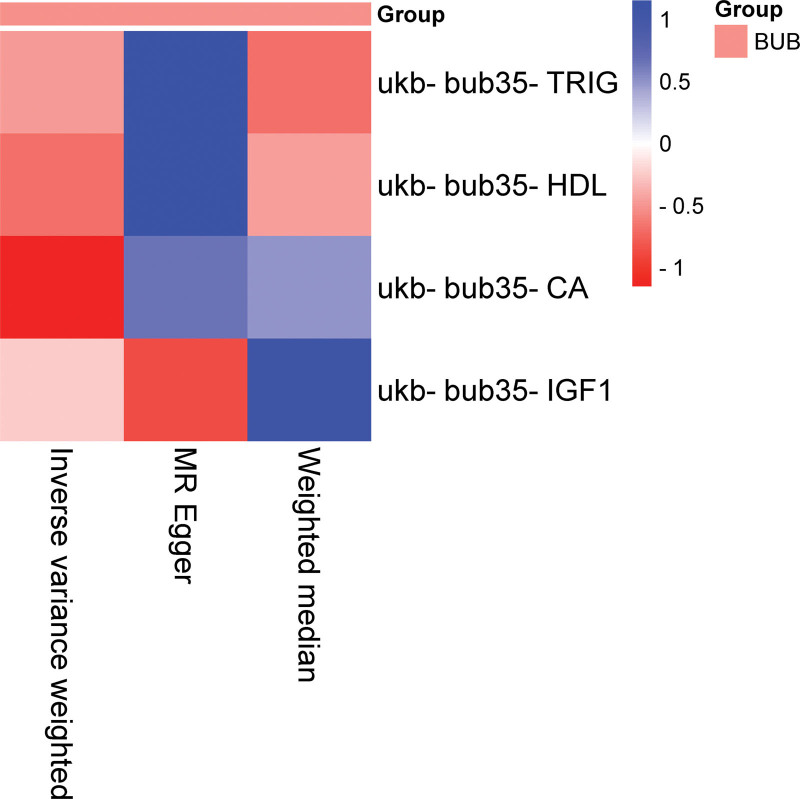
Heatmap illustrating significant correlations in forward MR. The figure showcases varying *P*-values in distinct blocks, color coded from red to blue denoting ascending *P*-values. The *X*-axis delineates 3 separate outcomes: MR-Egger, weighted median, and inverse variance weighted. The *Y*-axis signifies different BUB. BUB = blood and urine biomarkers, MR = Mendelian randomization.

**Figure 3.**
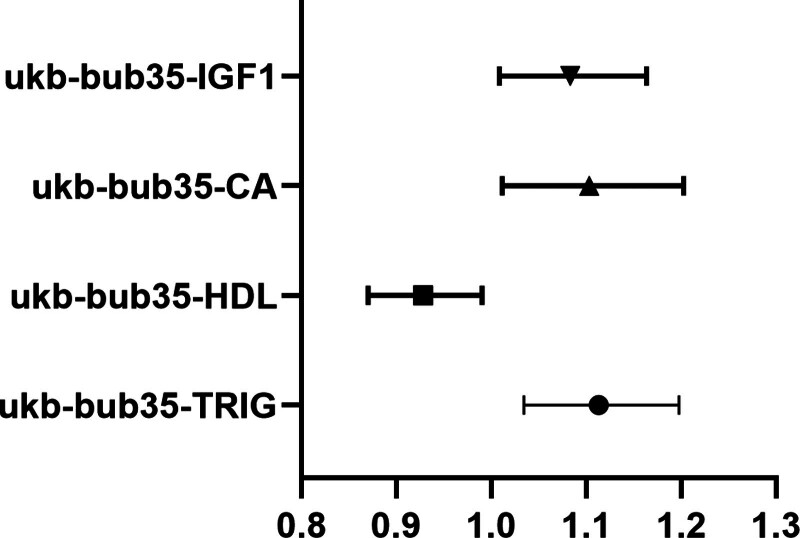
Forest plot depicting significant correlations in forward MR. The *Y*-axis represents diverse circulating metabolites, while the *X*-axis indicates OR values and corresponding 95% CIs. Various shapes in the graph symbolize distinct metabolite ORs, with the horizontal line denoting the range of the 95% CI. CI = confidence interval, MR = Mendelian randomization, OR = odds ratio.

The IVW analysis reveals a significant genetic correlation (*P* < .05) between 4 BUB and EMs (Fig. 2, Tables 2 and S2, Supplemental Digital Content, https://links.lww.com/MD/R314). Triglycerides (TRIG; *P* = .00423271438450461, OR = 1.11308243948851, CI = 1.03429530465191–1.1978711607075), calcium (CA; *P* = .0265735051693922, OR = 1.10300330586783, CI = 1.01145818532749–1.20283399788935), and insulin-like growth factor-1 (IGF1; *P* = .0287197493301776, OR = 1.08321310492312, CI = 1.00834134280327–1.16364427487935, Table S2, Supplemental Digital Content, https://links.lww.com/MD/R314) were identified as risk factors for EMs. High-density lipoprotein (HDL; *P* = .0242773860266827, OR = 0.928281161933669, CI = 0.870079033726982–0.990376600513873, Table S2, Supplemental Digital Content, https://links.lww.com/MD/R314) was a protective factor for EMs (Table 2, Fig. 3). No substantial evidence of horizontal pleiotropy among SNPs is observed (Table 3, *P* > .05). Through MR-Egger outcomes, only one (ukb-bub35-GGT) substantial heterogeneity is not detected in relation to the association (Tables 4 and S2, Supplemental Digital Content, https://links.lww.com/MD/R314). Forest plot, scatter plot, funnel plot and SNP leave-one-out analysis plot can be found in OA_Supplemental Digital Content, http://links.lww.com/MD/R464.

**Table 2:** Forward MR IVW.

id.exposure	id.outcome	Method	nsnp	*P*-value	OR
ukb-bub35-TRIG	finngen_R10_N14_ENDOMETRIOSIS	IVW	215	.004232714	1.113082439
ukb-bub35-HDL	finngen_R10_N14_ENDOMETRIOSIS	IVW	237	.024277386	0.928281162
ukb-bub35-CA	finngen_R10_N14_ENDOMETRIOSIS	IVW	183	.026573505	1.103003306
ukb-bub35-IGF1	finngen_R10_N14_ENDOMETRIOSIS	IVW	305	.028719749	1.083213105

IGF1 = insulin-like growth factor-1, IVW = inverse variance weighted, MR = Mendelian randomization, nsnp = number of single nucleotide polymorphisms, OR = odds ratio.

**Table 3:** Forward MR horizontal pleiotropy.

id.exposure	id.outcome	Egger_intercept	se	*P*-value
ukb-bub35-TRIG	finngen_R10_N14_ENDOMETRIOSIS	−0.001588586	0.002039047	.436795722
ukb-bub35-HDL	finngen_R10_N14_ENDOMETRIOSIS	−0.001116256	0.001926312	.562821653
ukb-bub35-CA	finngen_R10_N14_ENDOMETRIOSIS	0.003385149	0.002499647	.177344591
ukb-bub35-IGF1	finngen_R10_N14_ENDOMETRIOSIS	−0.004104903	0.002041938	.045285633

IGF1 = insulin-like growth factor-1, MR = Mendelian randomization, se = standard error.

**Table 4:** Forward MR heterogeneity.

id.exposure	id.outcome	Method	*Q*_*P*val
ukb-bub35-TRIG	finngen_R10_N14_ENDOMETRIOSIS	MR-Egger	3.17E−09
ukb-bub35-HDL	finngen_R10_N14_ENDOMETRIOSIS	MR-Egger	1.52E−09
ukb-bub35-CA	finngen_R10_N14_ENDOMETRIOSIS	MR-Egger	5.59E−05
ukb-bub35-IGF1	finngen_R10_N14_ENDOMETRIOSIS	MR-Egger	2.03E−07

IGF1 = insulin-like growth factor-1, MR = Mendelian randomization.

Furthermore, to validate the robustness of these findings, we conducted several sensitivity analyses. We explored the potential biological mechanisms underlying these associations. The elevated levels of TRIG might contribute to EMs by promoting inflammation and oxidative stress in the endometrial tissue. Excess TRIG can lead to the production of reactive oxygen species, which can damage the DNA and disrupt normal cellular functions in the endometrium. Similarly, CA is known to play a crucial role in cell-signaling pathways. An imbalance in CA levels could potentially disrupt the normal growth and differentiation of endometrial cells, increasing the risk of EMs.

Regarding IGF1, it is a well-known growth factor that stimulates cell proliferation and inhibits apoptosis. Higher levels of IGF1 in the circulation might lead to excessive growth of endometrial cells, facilitating the development of EMs. On the other hand, HDL, with its anti-inflammatory and antioxidant properties, could protect the endometrium from the detrimental effects of inflammation and oxidative stress, thereby reducing the risk of EMs.

In addition, we investigated the potential for using these BUB factors as biomarkers for the early detection of EMs. A combination of these factors could potentially improve the diagnostic accuracy of EMs, especially in the early stages when symptoms are often nonspecific. Future studies could focus on developing a diagnostic model based on these BUB factors to enhance the early identification and treatment of EMs.

Considering the public health perspective, these findings suggest that lifestyle modifications aimed at controlling BUB levels could be an effective preventive strategy for EMs. Dietary changes to reduce TRIG intake and increase HDL levels, along with regular physical activity, could potentially lower the risk of EMs. Further large-scale, long-term intervention studies are needed to confirm these hypotheses and develop evidence-based preventive measures.

### 3.2. EMs reverse MR

**Figure 4.**
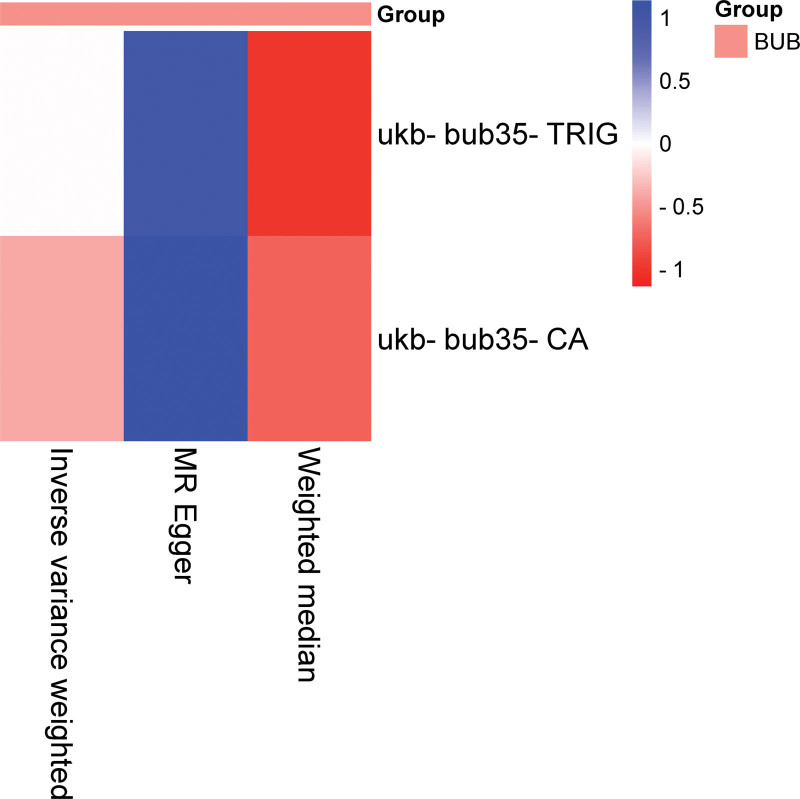
Heatmap illustrating significant correlations in reverse MR. The figure showcases varying *P*-values in distinct blocks, color coded from red to blue denoting ascending *P*-values. The *X*-axis delineates 3 separate outcomes: MR-Egger, weighted median, and inverse variance weighted. The *Y*-axis signifies different BUB. BUB = blood and urine biomarkers, MR = Mendelian randomization.

**Figure 5.**
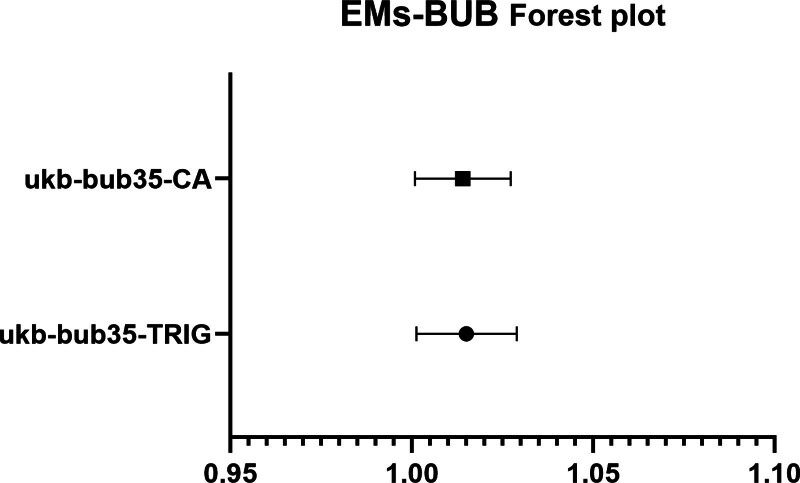
Forest plot depicting significant correlations in reverse MR. The *Y*-axis represents diverse circulating metabolites, while the *X*-axis indicates OR values and corresponding 95% CIs. Various shapes in the graph symbolize distinct metabolite ORs, with the horizontal line denoting the range of the 95% CI. CI = confidence interval, MR = Mendelian randomization, OR = odds ratio.

The IVW analysis reveals a significant genetic correlation (*P* < .05) between 2 BUB and EMs (Fig. 4, Tables 5 and S2, Supplemental Digital Content, https://links.lww.com/MD/R314). EMs was identified as a risk factor for TRIG (*P* = .0314124075984745, OR = 1.01505516104807, CI = 1.00133287952017–1.02896549293782, Table S2, Supplemental Digital Content, https://links.lww.com/MD/R314) and CA (*P* = .0363165467253371, OR = 1.01400110223924, CI = 1.00088619658911–1.02728785634806; Fig. 5, Tables 5 and S2, Supplemental Digital Content, https://links.lww.com/MD/R314). No substantial evidence of horizontal pleiotropy among SNPs is observed (Table 6, *P* > .05). Through MR-Egger outcomes, no substantial heterogeneity is detected in relation to the association (Tables 7 and S2, Supplemental Digital Content, https://links.lww.com/MD/R314). Forest plot, scatter plot, funnel plot and SNP leave-one-out analysis plot can be found in OA_Supplemental Digital Content, http://links.lww.com/MD/R464.

**Table 5:** Reverse MR IVW.

id.exposure	id.outcome	Method	nsnp	*P*-value	OR
finngen_R10_N14_ENDOMETRIOSIS	ukb-bub35-TRIG	IVW	23	.031412408	1.015055161
finngen_R10_N14_ENDOMETRIOSIS	ukb-bub35-CA	IVW	23	.036316547	1.014001102

IVW = inverse variance weighted, MR = Mendelian randomization, nsnp = number of single nucleotide polymorphisms, OR = odds ratio.

**Table 6:** Reverse MR horizontal pleiotropy.

id.exposure	id.outcome	Egger_intercept	se	*P*-value
finngen_R10_N14_ENDOMETRIOSIS	ukb-bub35-TRIG	−0.002872582	0.00207299	.180374083
finngen_R10_N14_ENDOMETRIOSIS	ukb-bub35-CA	−0.001674598	0.002018708	.416129614

MR = Mendelian randomization, se = standard error.

**Table 7:** Reverse MR heterogeneity.

id.exposure	id.outcome	Method	*Q*_*P*val
finngen_R10_N14_ENDOMETRIOSIS	ukb-bub35-TRIG	MR-Egger	0.05495642
finngen_R10_N14_ENDOMETRIOSIS	ukb-bub35-CA	MR-Egger	0.132700468

MR = Mendelian randomization.

Furthermore, the sensitivity analysis was carried out to validate the reliability of these findings. The leave-one-out analysis showed that no single SNP had a disproportionate influence on the overall results of the IVW analysis for the associations between BUB, EMs, TRIG, and CA. This indicates that the significant genetic correlations and risk factor relationships identified are robust and not driven by a single genetic variant. In addition, we compared our results with previous studies in the field. Some previous research has also suggested a connection between similar genetic factors and lipid or CA-related phenotypes, but our study provides more direct evidence through the MR approach. Our findings not only confirm some of the previous speculations but also expand the understanding of the genetic basis of the relationships between BUB, EMs, TRIG, and CA. Looking ahead, future research could focus on validating these findings in larger and more diverse populations. Longitudinal studies could be conducted to explore the dynamic changes in these associations over time. Moreover, functional studies on the identified genes and pathways are needed to fully elucidate the molecular mechanisms by which EMs act as a risk factor for TRIG and CA. This could potentially lead to the development of novel therapeutic strategies targeting these genetic and biological processes.

## 4. Discussion

EMs is a common health concern among women and is affected by a complex interaction of biological and environmental factors. The main aim of this study was to explore the mutual relationship between BUB and EMs through bidirectional MR, thereby revealing the underlying pathogenic mechanism. This 2-way MR investigation found a clear association between BUB and EMs. The results of this study suggest that the risk of EMs increases when TRIG, CA, and IGF1 levels are elevated, while the risk of EMs decreases with higher levels of HDL. However, when EMs occurs, the risk of TRIG and CA also increases. This means that there is a close internal relationship between TRIG, CA and EMs. The risk of TRIG and CA increases when EMs occur, and the risk of EMs increases when TRIG and CA are exposed. It may be clinically observed that TRIG and CA are not only contributing factors to EMs but also potential markers of clinical severity. Therefore, TRIG and CA have the potential to be used as diagnostic markers of EMs. At the same time, they can also be used as therapeutic targets for studying drug effects. TRIG can lead to aggravated systemic inflammatory response and increased risk of death,^[12]^ while the elevation of TRIG will cause metabolic syndrome, leading to metabolic disorders and vascular damage.^[13]^ The promotion of TRIG to systemic inflammatory response and metabolic syndrome may lead to aggravated inflammation and disease progression of EMs. CA may lead to vascular dysfunction^[14]^ by regulating endothelial cell secretion and related cell pathways, thus aggravating EMs. IGF1 is a peptide hormone which plays a pivotal role in regulating cell proliferation, differentiation and apoptosis, plays an important role in mediating and modulating the sex hormone-induced growth and differentiation of endometrial cells.^[15]^ A retrospective study revealed that patients with high TRIG levels exhibited more severe EMs lesions, indicating a potential positive correlation between TRIG levels and the degree of EMs lesions.^[16]^ An in vitro study demonstrated the presence of CA accumulation in the cytosol of EMs cells, which could be alleviated by inhibiting CA accumulation.^[17]^ However, the relationship between the specific content of serum CA and triglyceride and EMs, as well as the specific threshold, needs to be further studied. In a clinical randomized controlled trial, serum total cholesterol, low-density lipoprotein, and triglyceride levels were significantly higher in women with EMs compared to controls, while HDL levels were significantly lower.^[18]^ HDL was also decreased in a category of infertility that included EMs.^[19]^ The present study suggests that HDL has a protective effect on EMs, which may stem from the predominant role of HDL in regulating blood lipids. Several retrospective case-control studies have shown that IGF1 is associated with EMs, particularly with a positive correlation in women under 40 years of age.^[20]^ The correlation between IGF1 and EMs was consistent with the results of our study.

From a diagnostic perspective, the results of this study may help improve the early diagnosis of EMs. At present, the diagnosis of EMs mainly relies on ultrasound imaging, as specific markers for serum and urine detection have not been developed. The present study found that TRIG, CA, and IGF1 in BUB may be involved in the occurrence of EMs; therefore, they may have the potential to be used as biomarkers of EMs disease for early diagnosis of EMs.

From a therapeutic perspective, the results of this study may contribute to the development of innovative treatment strategies for individuals with EMs. If we can understand how TRIG, CA, and IGF1 affect the development of EMs, we may be able to develop targeted drugs that could be used to treat EMs. At present, the results mostly indicate the regulation of blood lipids and stable CA in patients with EMs, demonstrating that maintaining the stability of the internal environment is beneficial for treating EMs.

Overall, the results of this study provide new insights for the detection and treatment of EMs. However, these results still need to be verified by more experimental and clinical studies. In addition, we need to explore how to translate these findings into clinical practice and how to use these BUBs for early diagnosis and prevention of EMs. At the same time, MR provides us with new possibilities for screening therapeutic targets and markers of EMs. In the future, we can utilize multi-omics and big data screening, followed by MR analysis, to uncover potential abnormal targets in EMs, enabling targeted research.

The great advantage of this study over traditional observational studies is that the results obtained through MR avoid reverse causality and confounding bias. Similarly, utilizing comprehensive and extensive GWAS data for MR analysis can enhance the accuracy of the results. We need more detailed experiments for verification to clarify the specific mechanism of BUB on EMs. While the study offers advancements, it is not without its limitations. The reliance on GWAS data assumes that the genetic variants used are valid proxies for the exposures under investigation, which may not always be the case. The study also faces challenges in generalizing the findings to broader populations, as the datasets utilized predominantly represent specific demographic groups. Future research should aim to address these gaps by incorporating more diverse populations and refining the selection criteria for IVs.

## Author contributions

**Conceptualization:** Ze-chao Zhang, Wei-hong Li.

**Data curation:** Jing Li.

**Formal analysis:** Shu-ping Huang.

**Investigation:** Ze-chao Zhang.

**Methodology:** Ze-chao Zhang, Cai-ying Xie.

**Project administration:** Jing Li.

**Resources:** Wen-jia Ding, Cai-ying Xie.

**Software:** Jing Li, Yi-ting Zhang.

**Supervision:** Yi-ting Zhang.

**Validation:** Liang-ying Li, Wen-jia Ding.

**Visualization:** Liang-ying Li, Cai-ying Xie.

**Writing – original draft:** Shu-ping Huang.

**Writing – review & editing:** Ze-chao Zhang, Wei-hong Li.

## Supplementary Material
