# Supplementary material for: Association of endometriosis with hematuria markers: A Mendelian randomization study
Source: Medicine (Baltimore). 2026 Feb 20;105(8):e45026. doi: 10.1097/MD.0000000000045026 (PMC12928905; doi:10.1097/MD.0000000000045026)
Supplement: Supplementary file 3 [file medi-105-e45026-s003.pdf]

GCST90257064

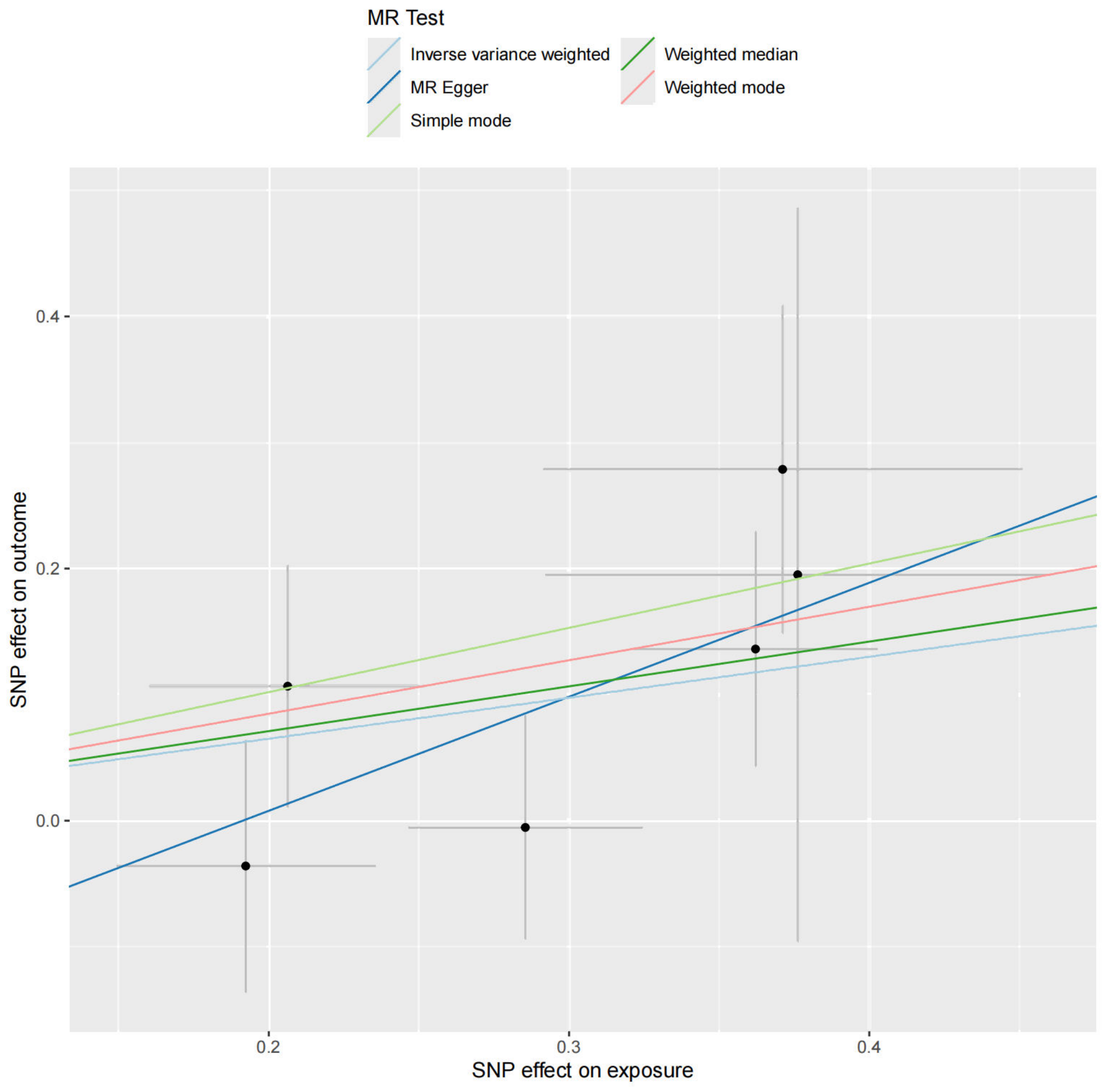

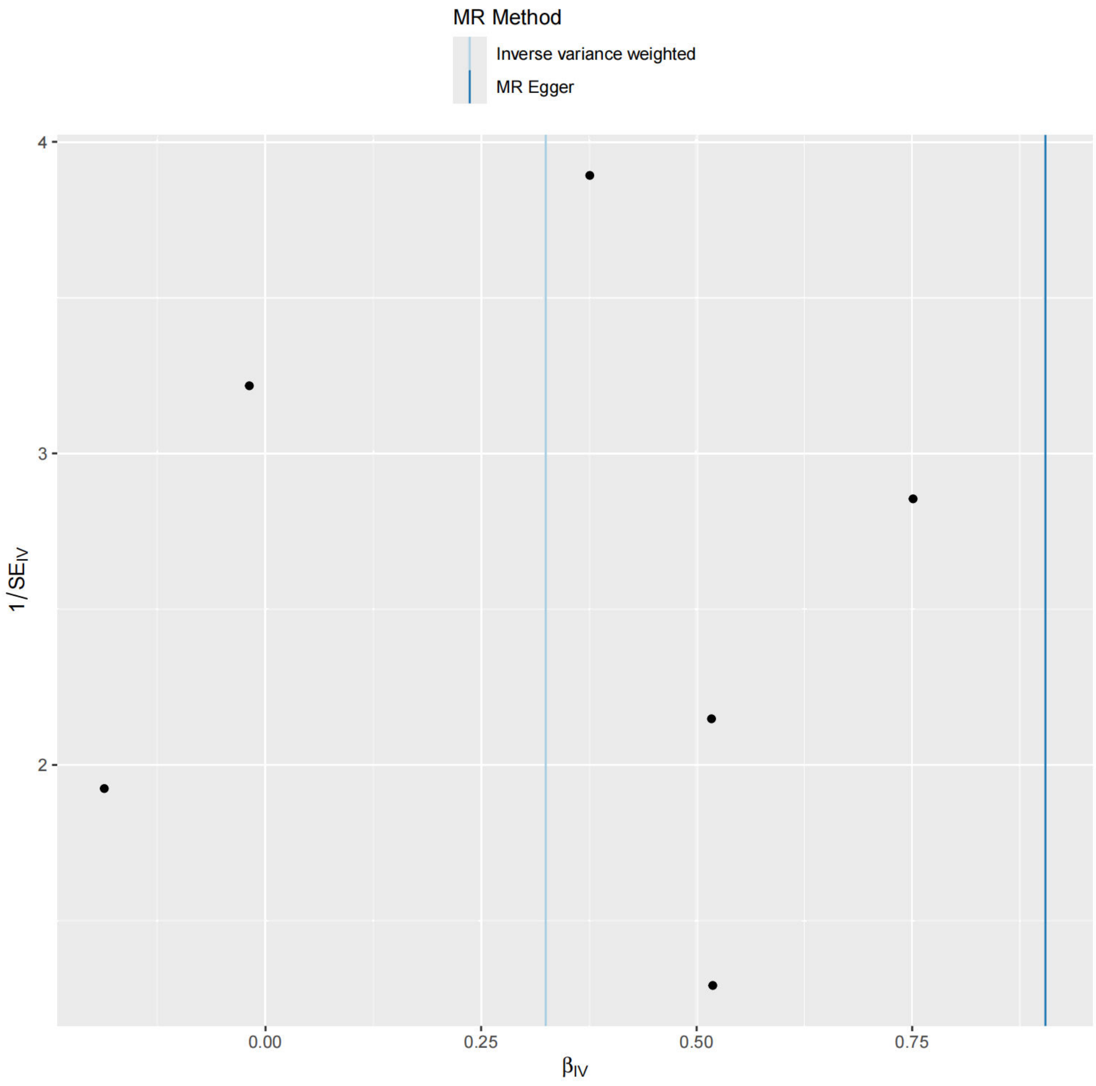
MR Method
Inverse variance weighted
MR Egger
4
3
2
1/SE_IV
0.00
0.25
0.50
0.75
β_IV

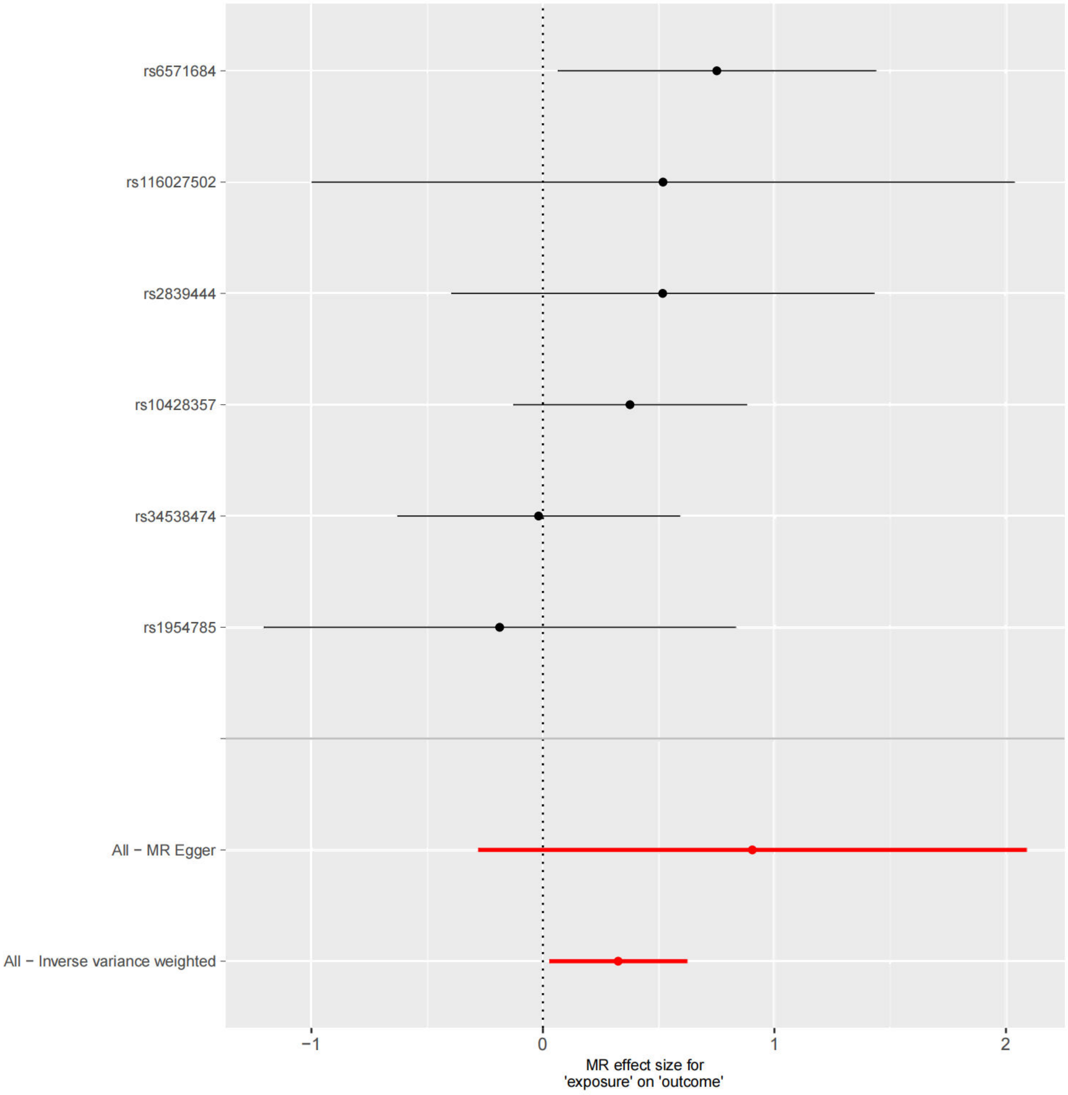
rs6571684
rs116027502
rs2839444
rs10428357
rs34538474
rs1954785
All − MR Egger
All − Inverse variance weighted
−1
0
1
2
MR effect size for
'exposure' on 'outcome'

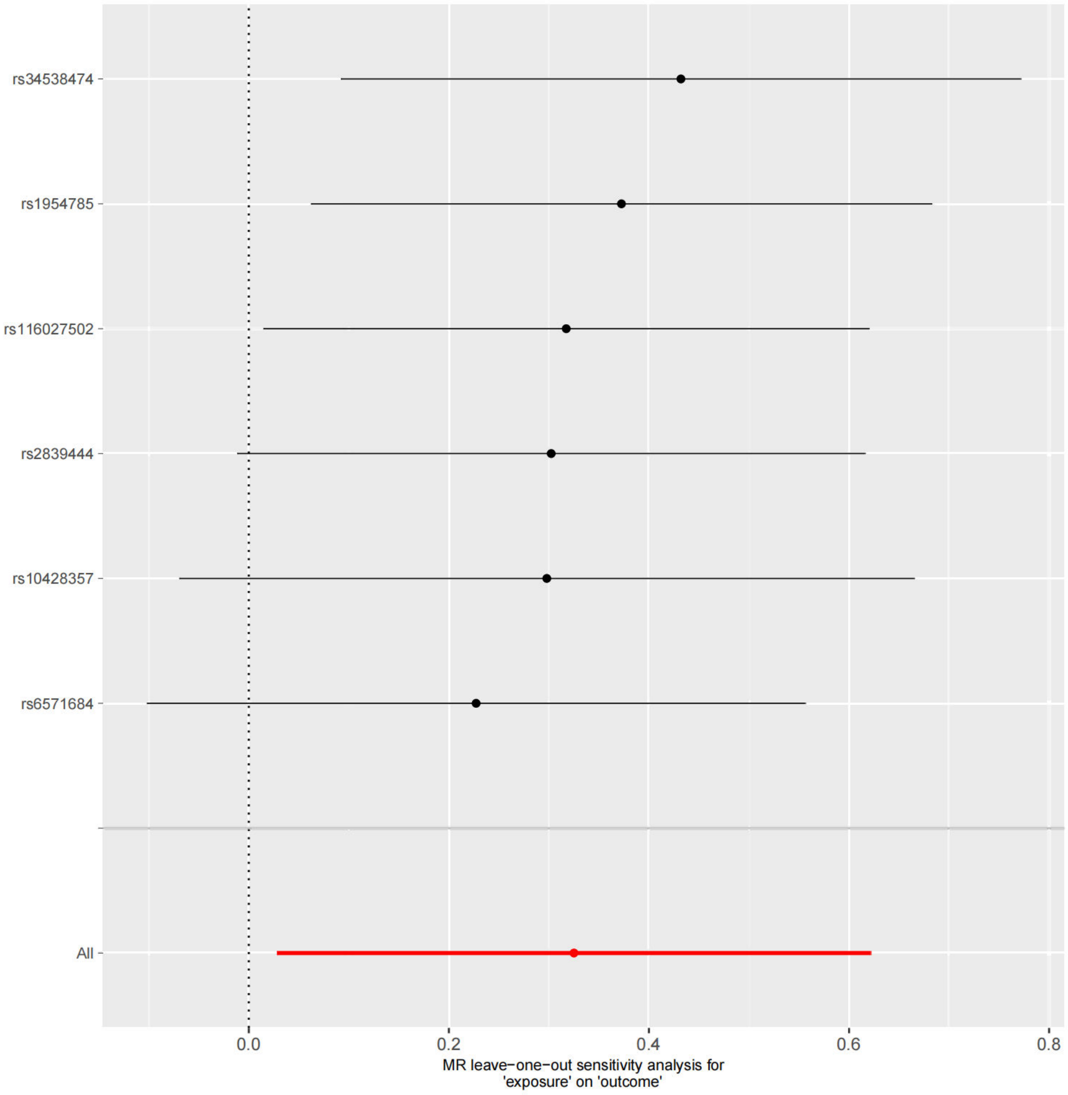
rs34538474
rs1954785
rs116027502
rs2839444
rs10428357
rs6571684
All
0.0
0.2
0.4
0.6
0.8
MR leave−one−out sensitivity analysis for
'exposure' on 'outcome'

GCST90257082

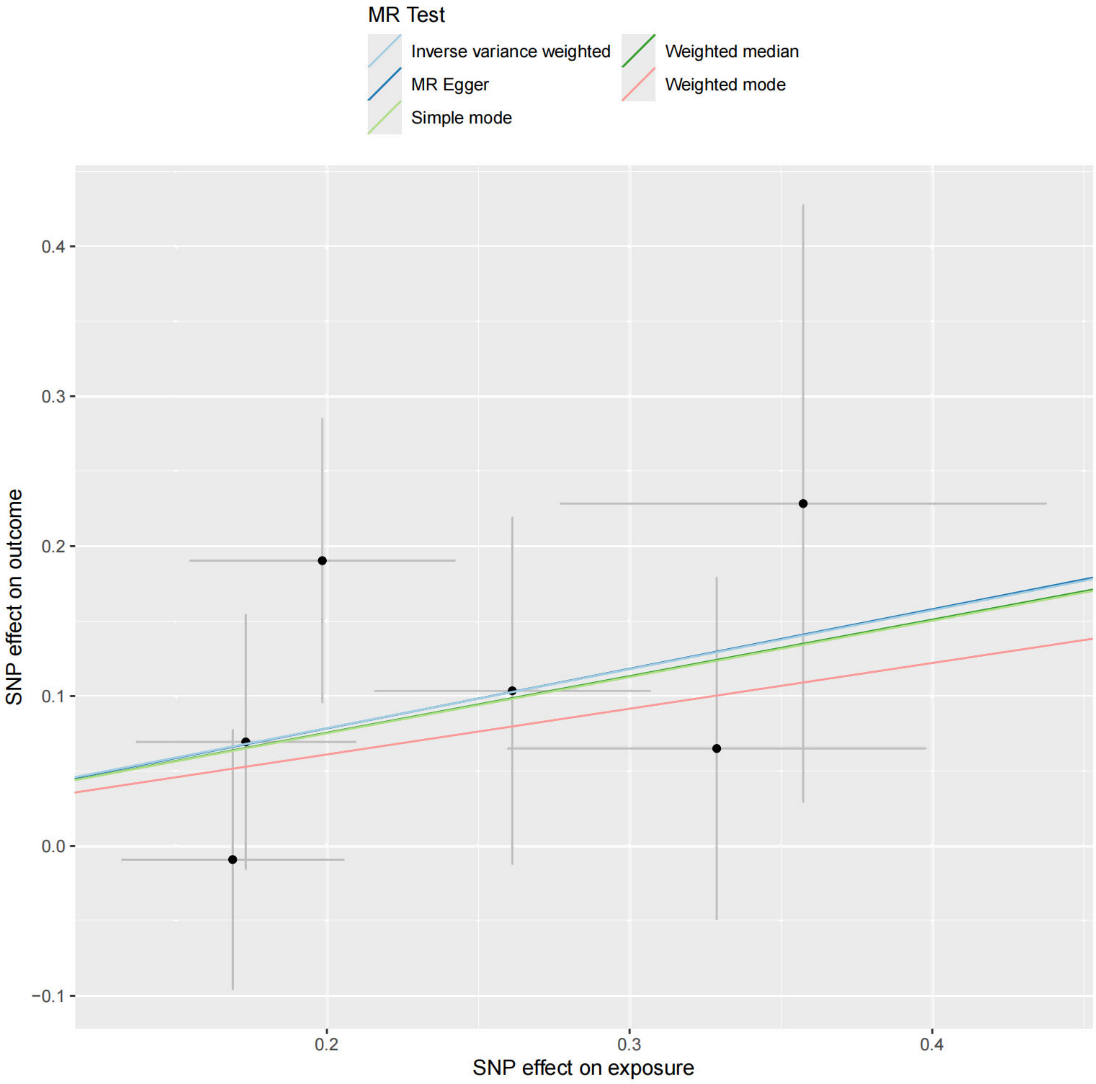

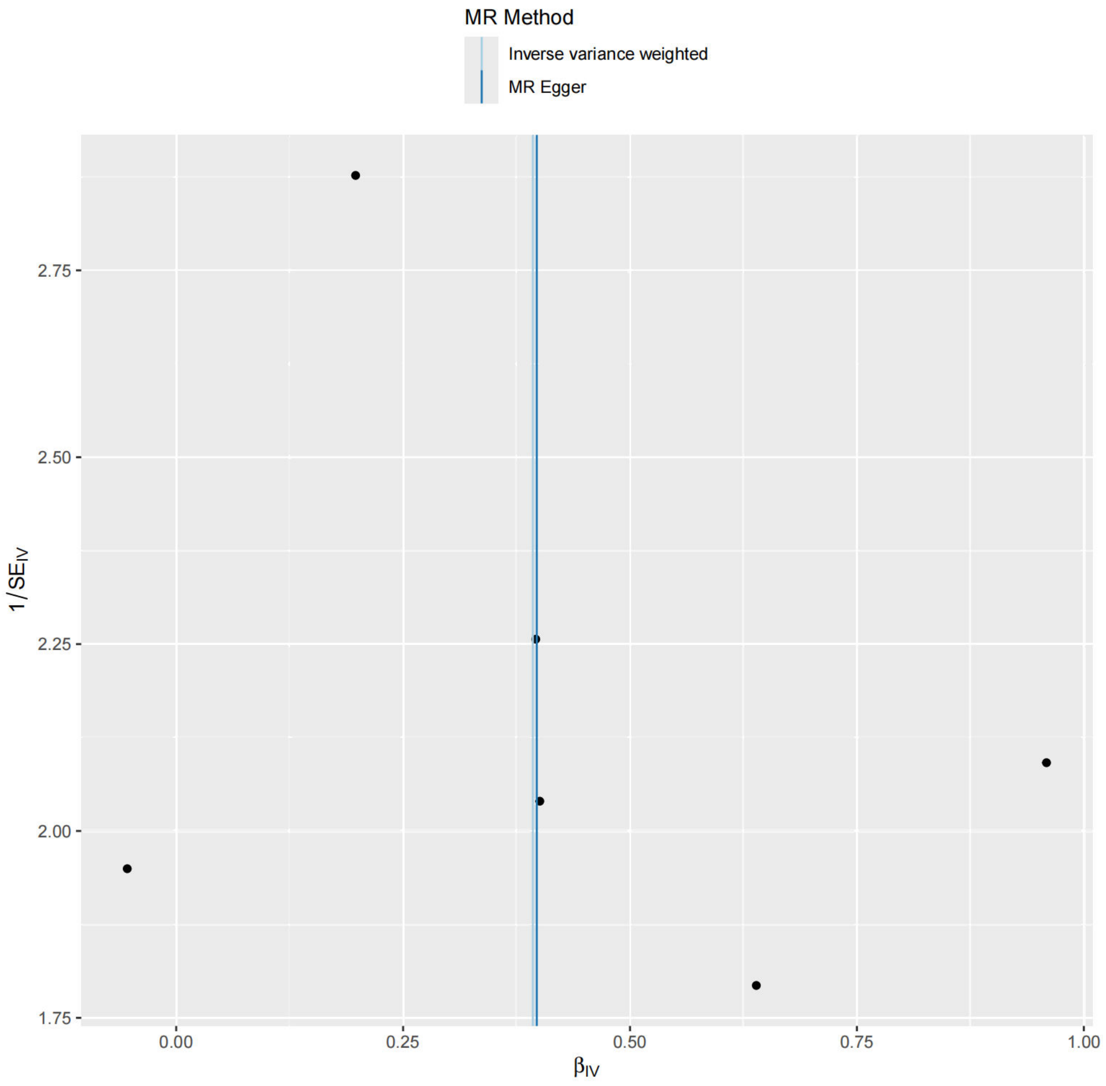
MR Method
Inverse variance weighted
MR Egger
1/SE_IV
2.75
2.50
2.25
2.00
1.75
0.00
0.25
0.50
0.75
1.00
β_IV

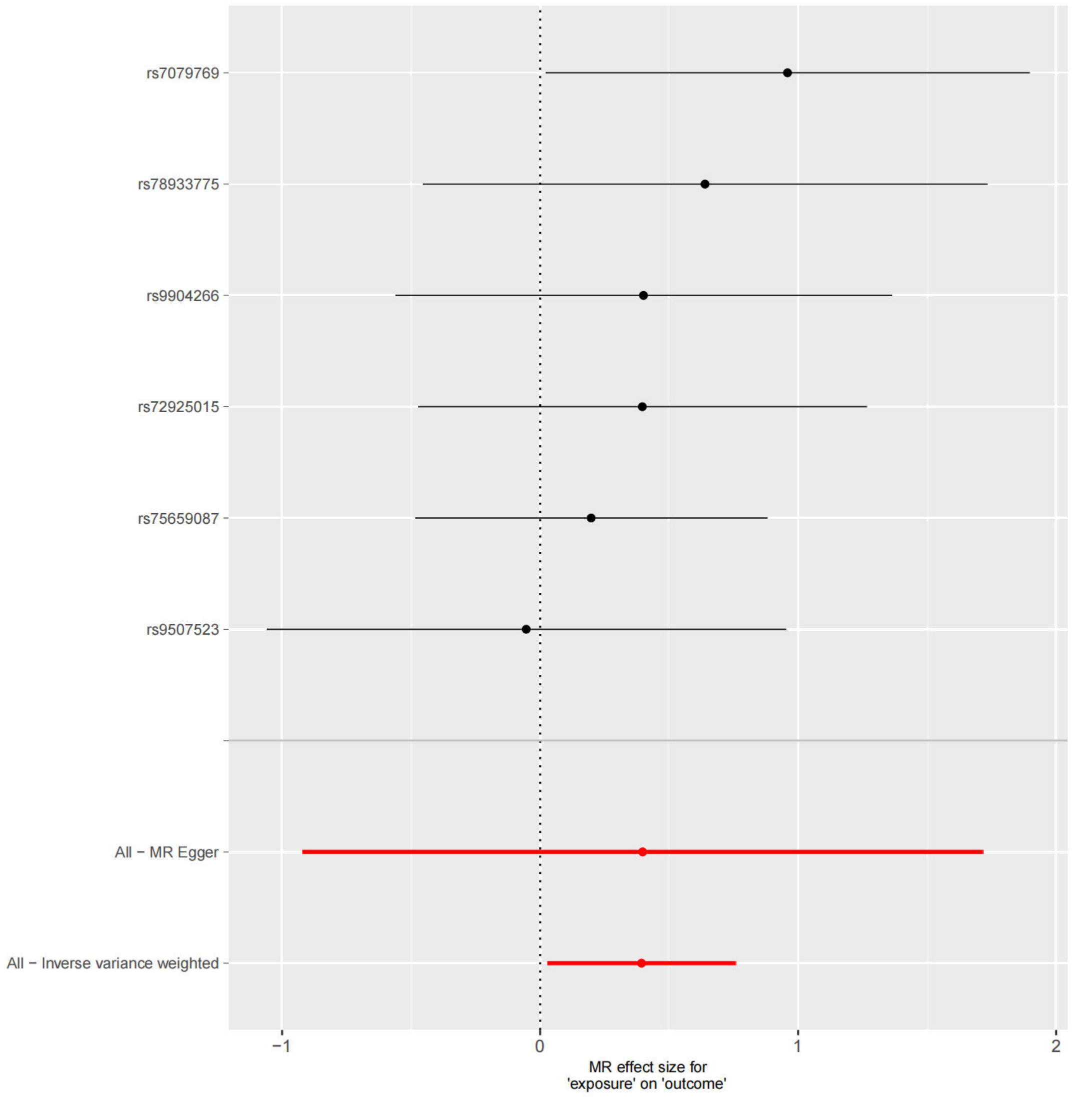

rs7079769
rs78933775
rs9904266
rs72925015
rs75659087
rs9507523
All − MR Egger
All − Inverse variance weighted
−1
0
1
2
MR effect size for
'exposure' on 'outcome'

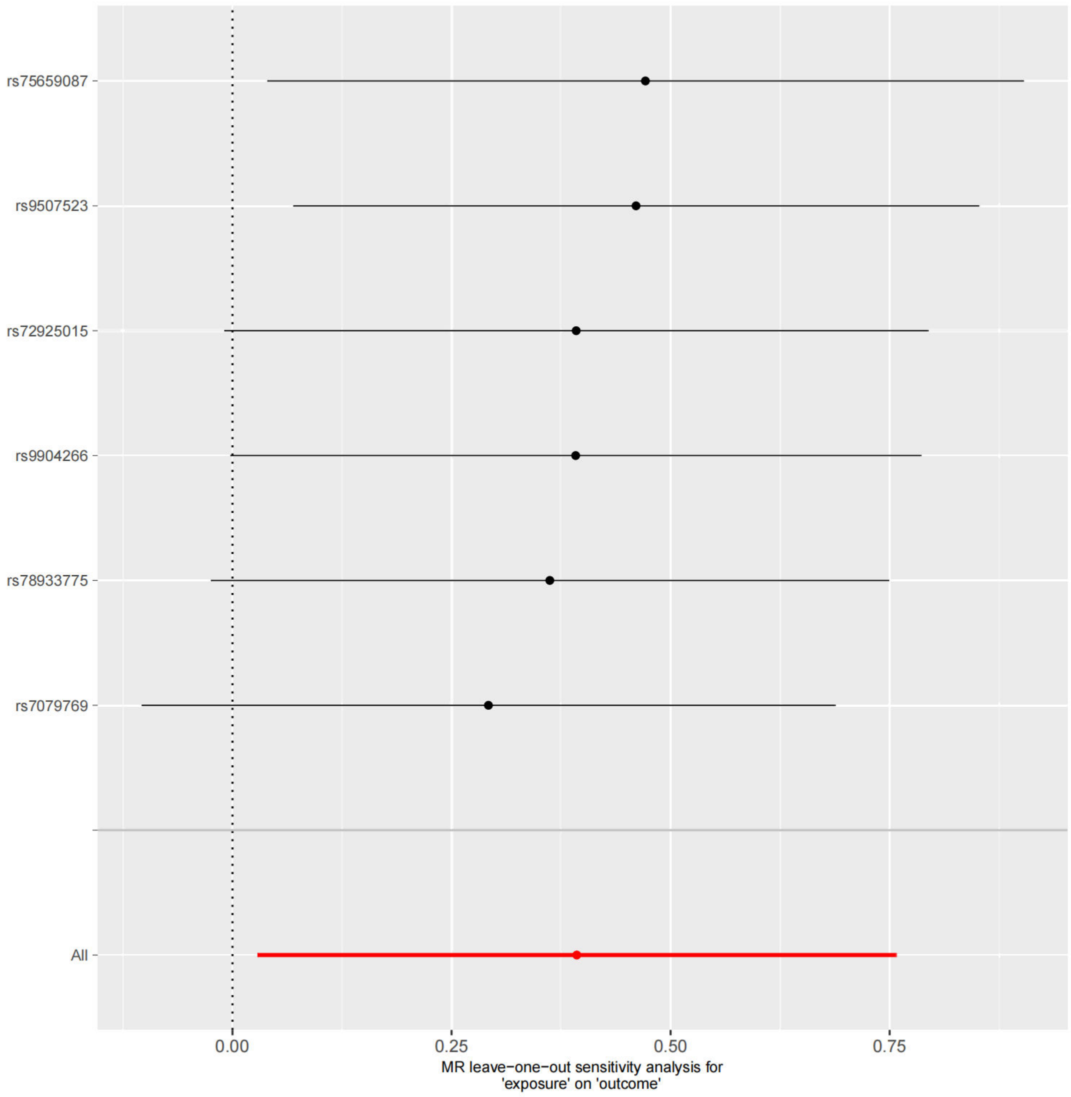
rs75659087
rs9507523
rs72925015
rs9904266
rs78933775
rs7079769
All
0.00
0.25
0.50
0.75
MR leave−one−out sensitivity analysis for
'exposure' on 'outcome'

GCST90257025

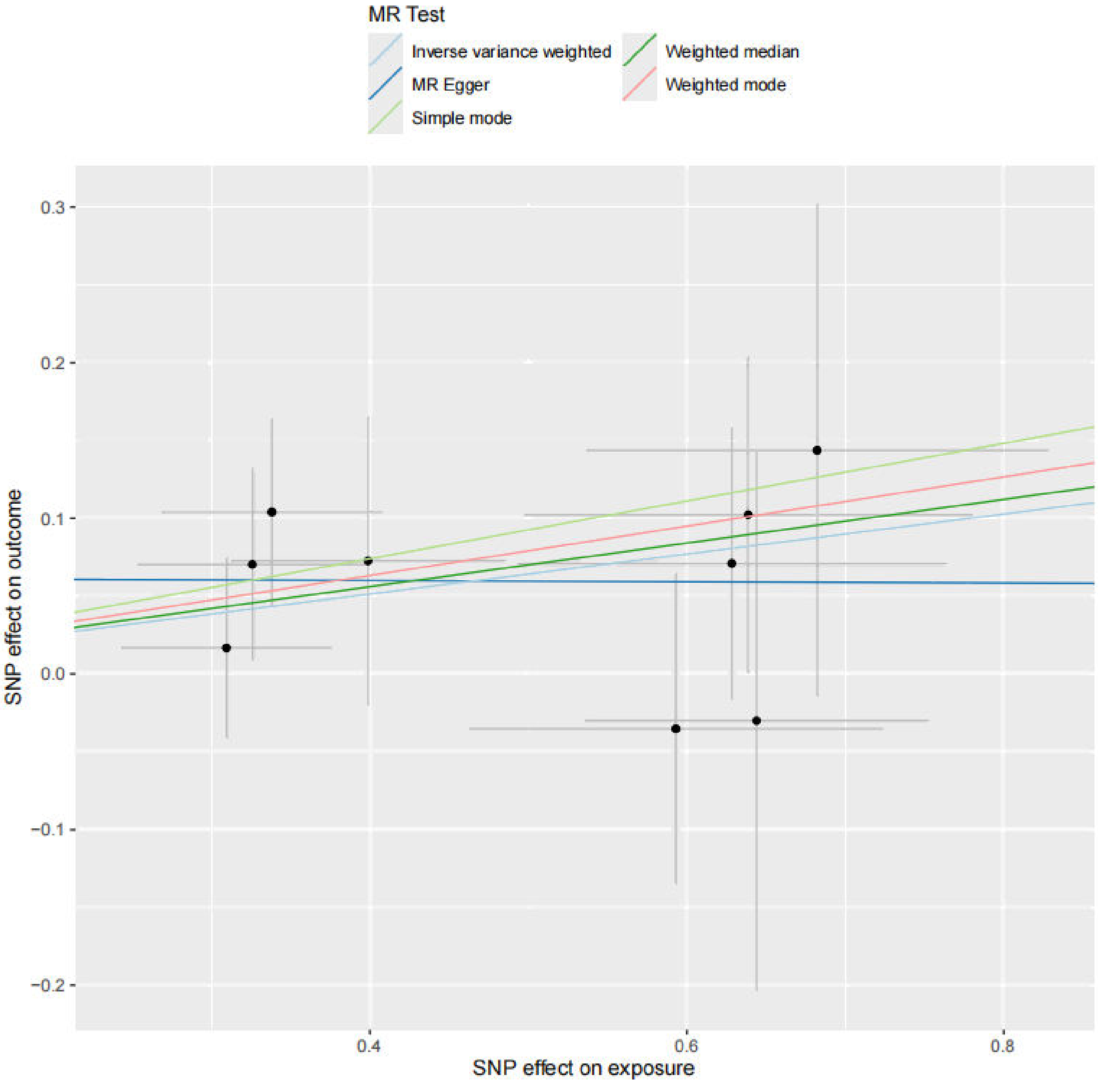

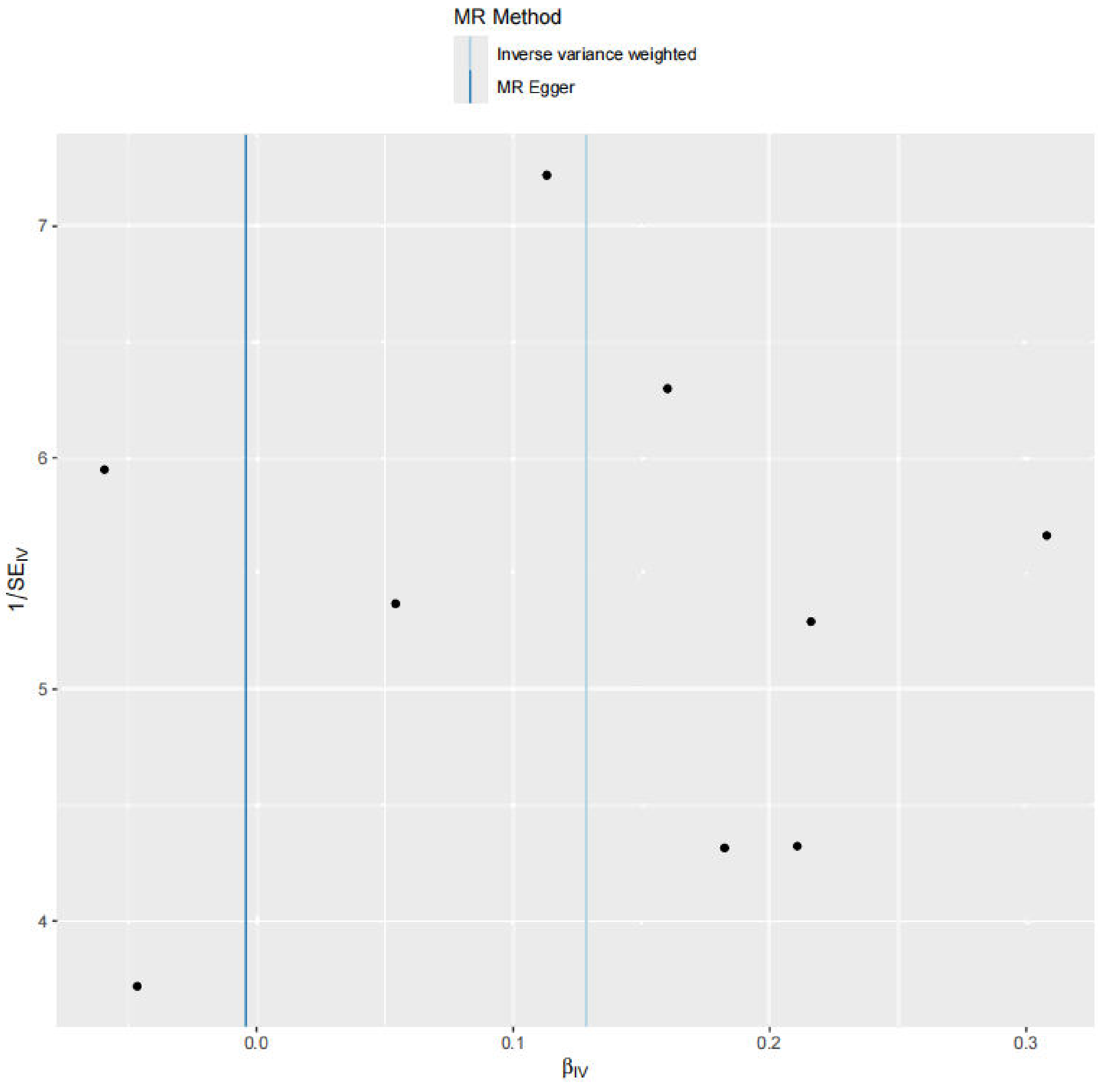

MR Method
Inverse variance weighted
MR Egger
7
6
5
4
1/SE$_{IV}$
0.0
0.1
0.2
0.3
β$_{IV}$

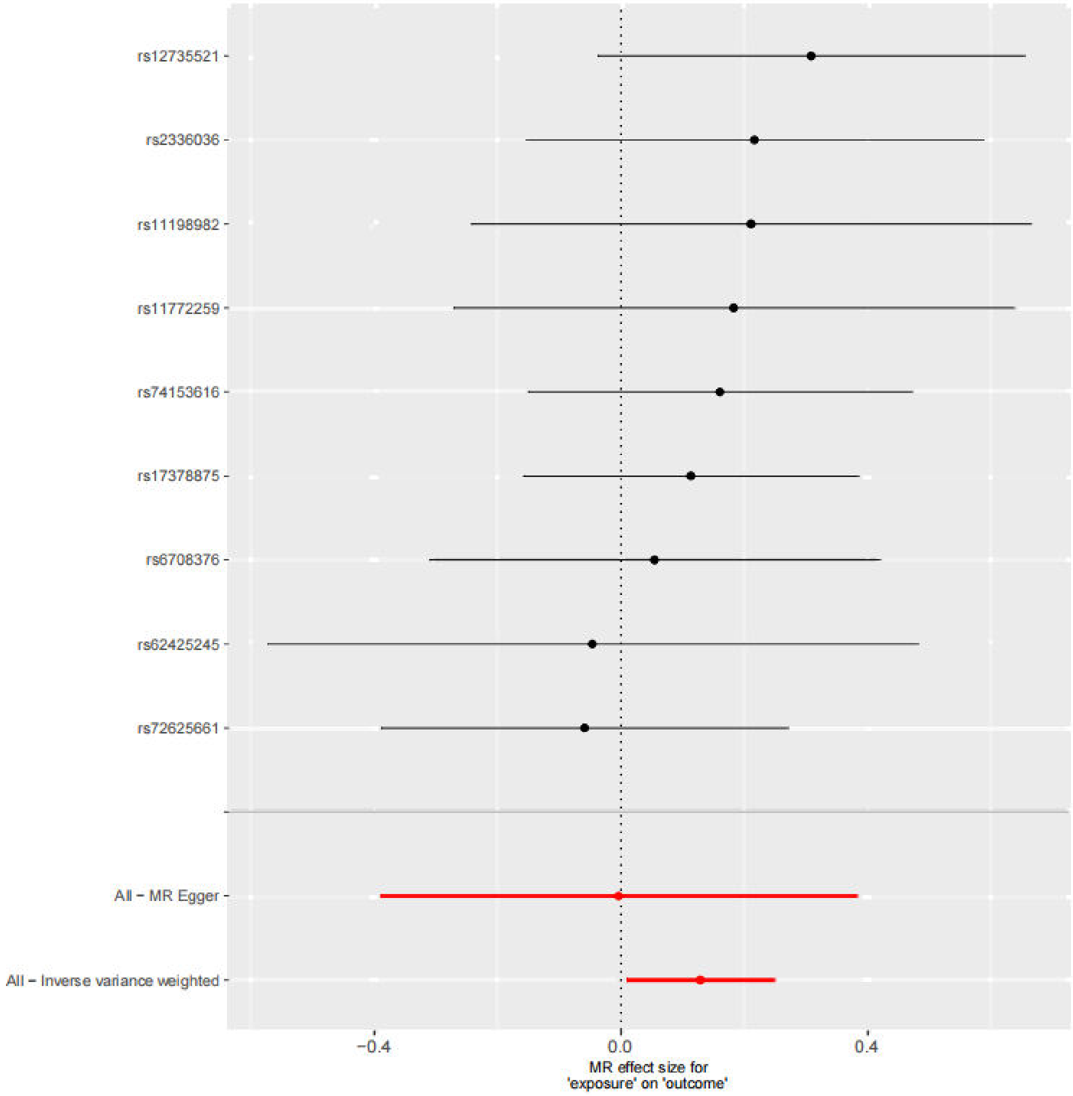
rs12735521
rs2336036
rs11198982
rs11772259
rs74153616
rs17378875
rs6708376
rs62425245
rs72625661
All − MR Egger
All − Inverse variance weighted
−0.4
0.0
0.4
MR effect size for
'exposure' on 'outcome'

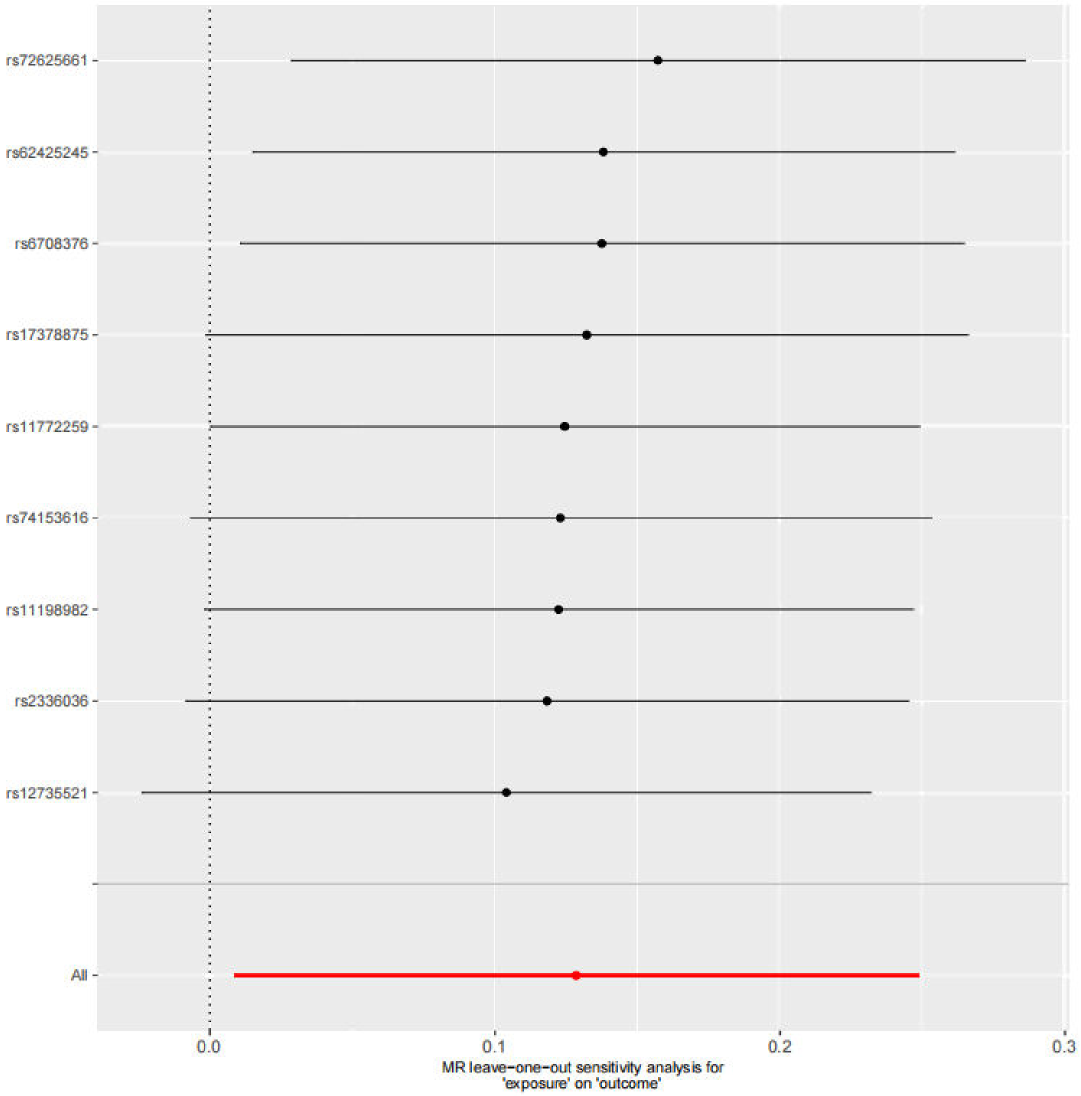
rs72625661
rs62425245
rs6708376
rs17378875
rs11772259
rs74153616
rs11198982
rs2336036
rs12735521
All
0.0
0.1
0.2
0.3
MR leave−one−out sensitivity analysis for
'exposure' on 'outcome'

GCST90257051

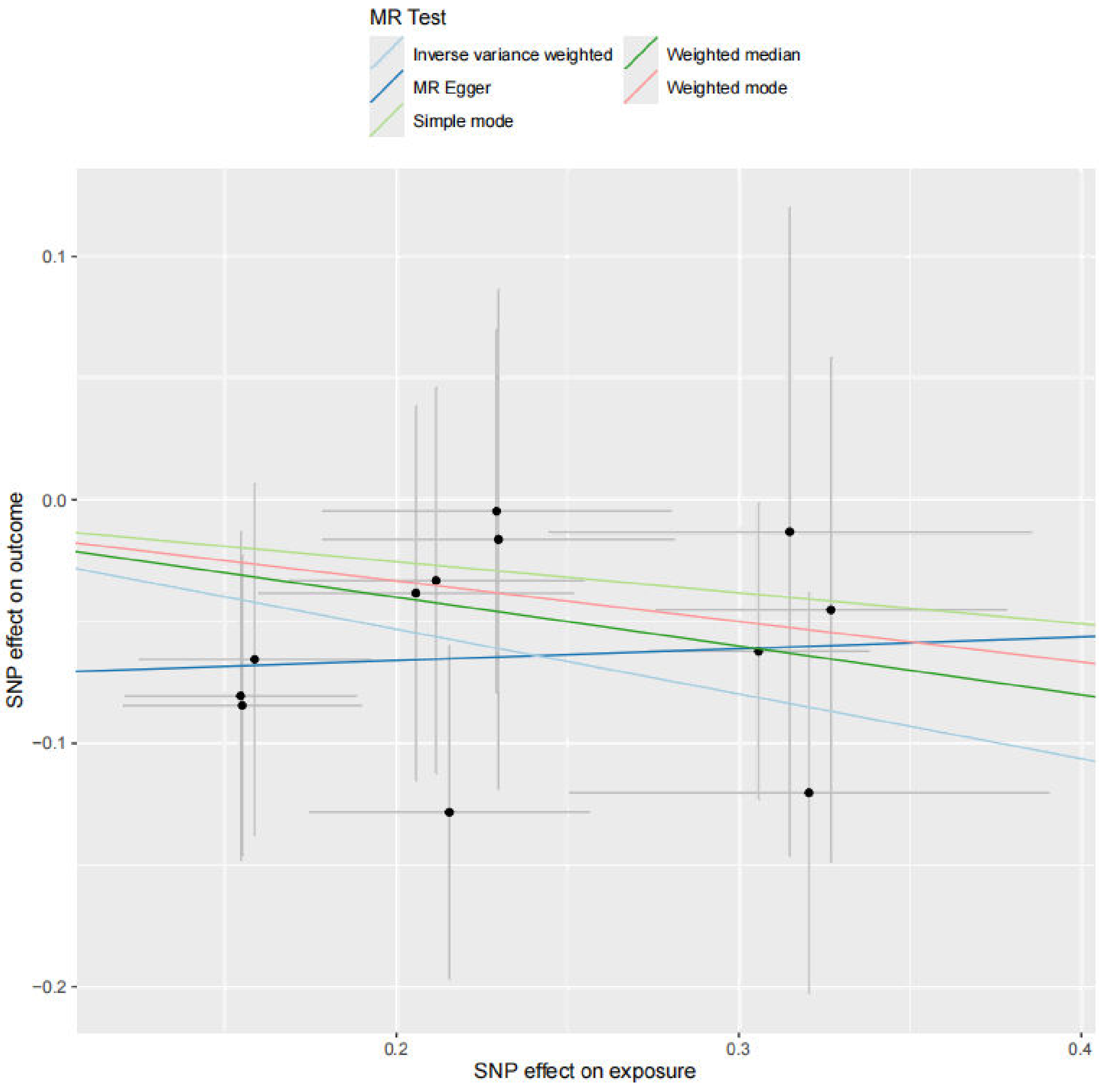

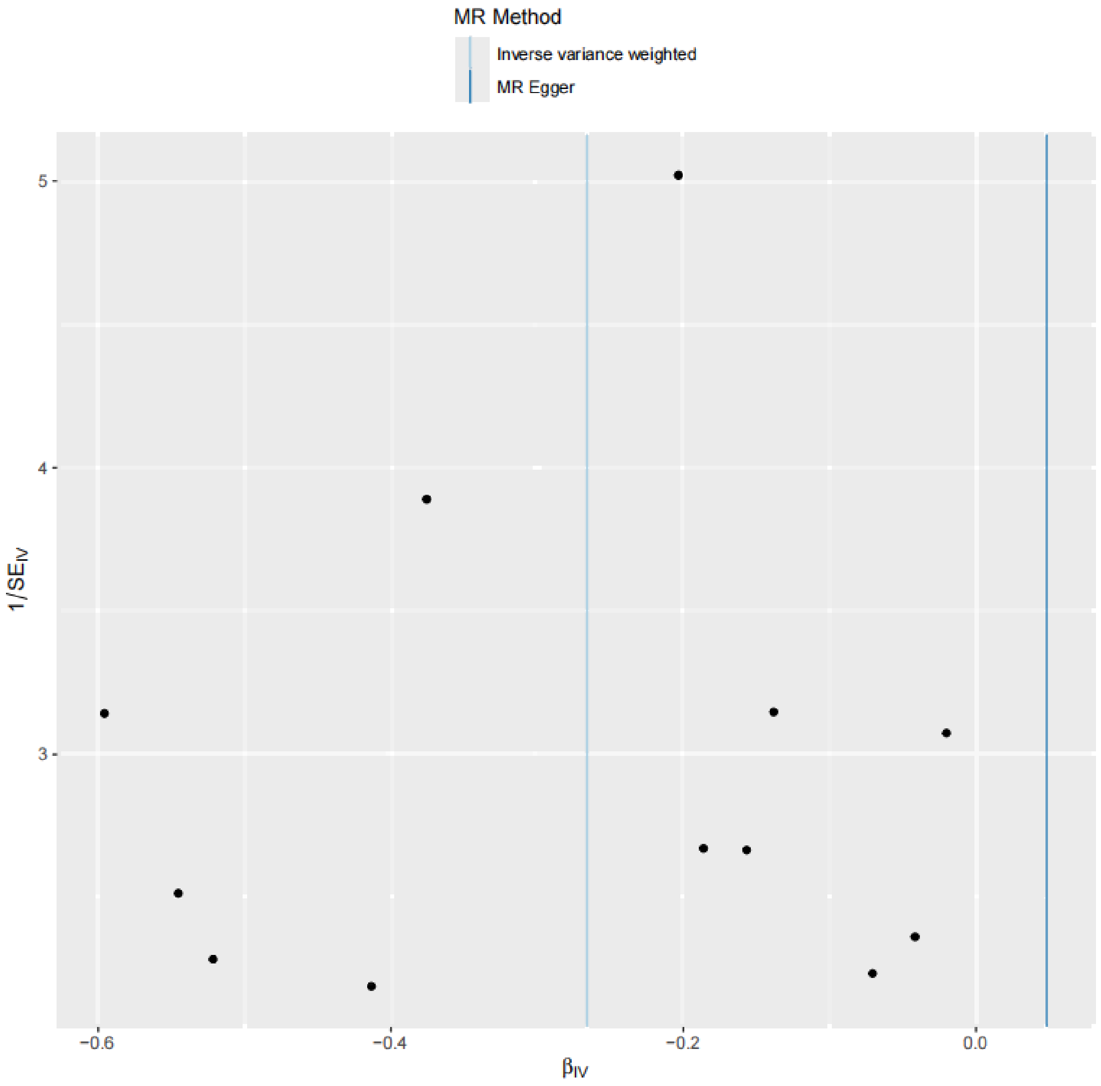

MR Method
Inverse variance weighted
MR Egger
5
4
3
$1/SE_{IV}$
−0.6
−0.4
−0.2
0.0
β_{IV}

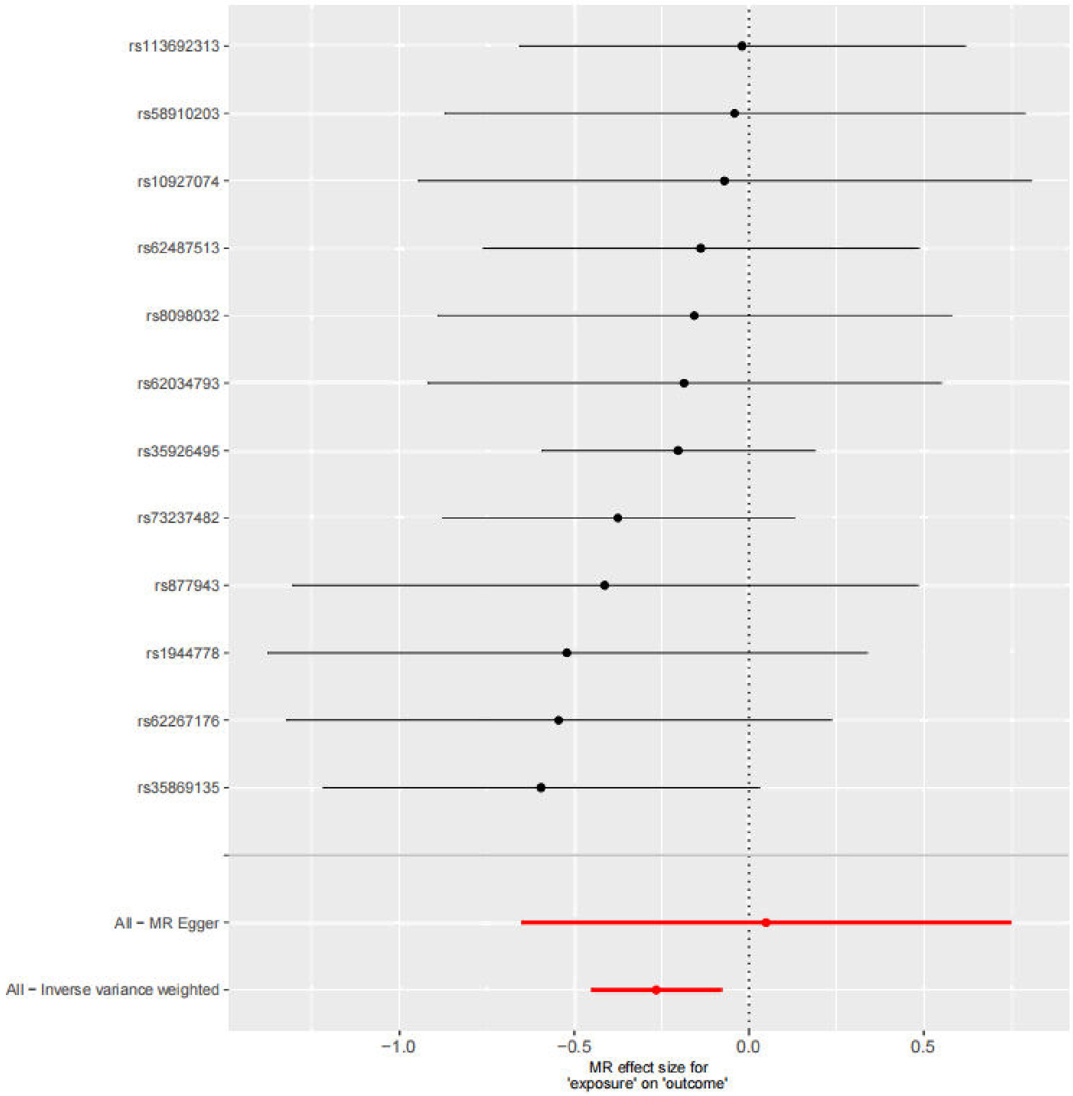
rs113692313
rs58910203
rs10927074
rs62487513
rs8098032
rs62034793
rs35926495
rs73237482
rs877943
rs1944778
rs62267176
rs35869135
All − MR Egger
All − Inverse variance weighted
−1.0
−0.5
0.0
0.5
MR effect size for
'exposure' on 'outcome'

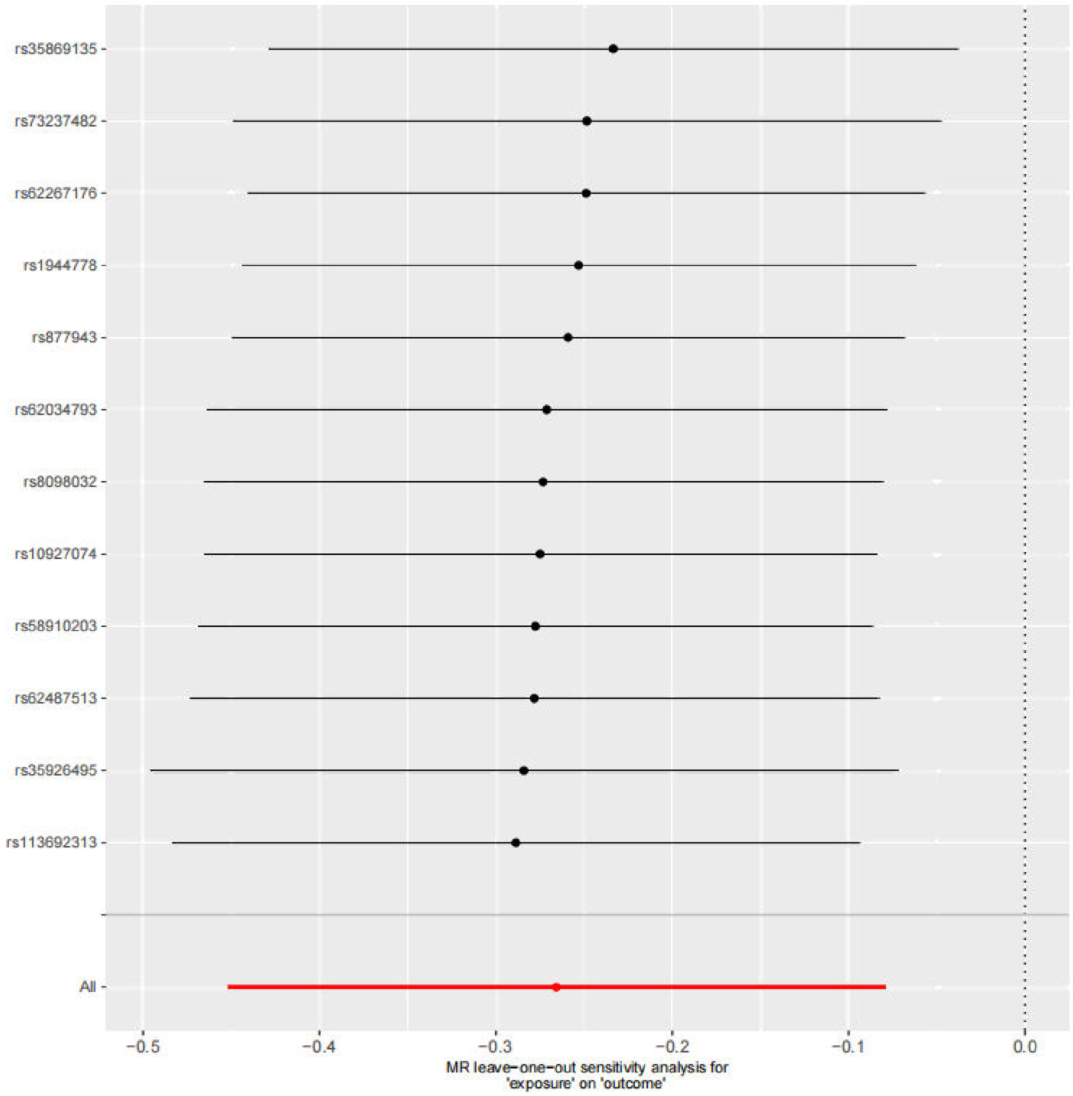

rs35869135
rs73237482
rs62267176
rs1944778
rs877943
rs62034793
rs8098032
rs10927074
rs58910203
rs62487513
rs35926495
rs113692313
All
−0.5
−0.4
−0.3
−0.2
−0.1
0.0
MR leave−one−out sensitivity analysis for
'exposure' on 'outcome'

GCST90257057

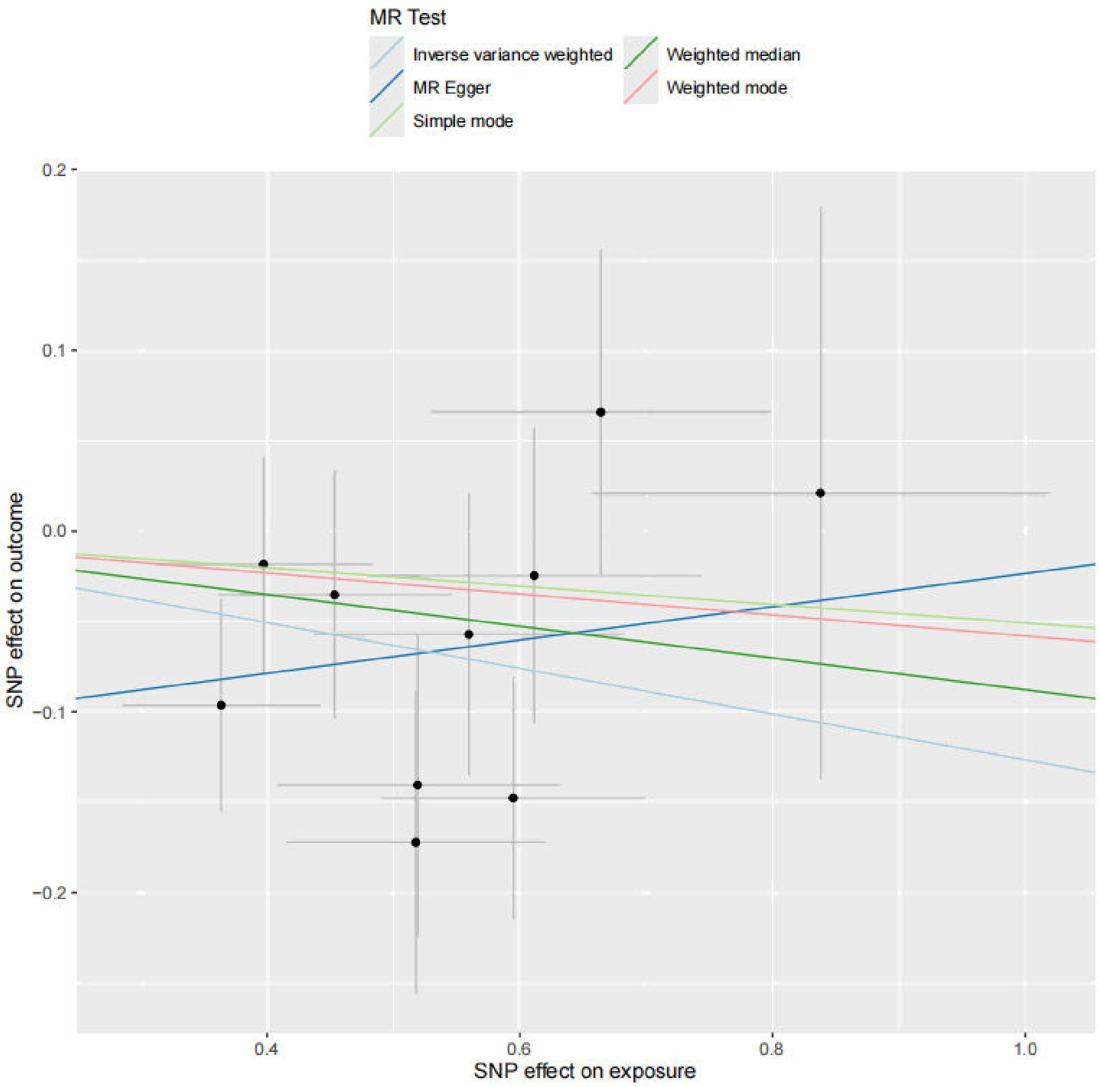

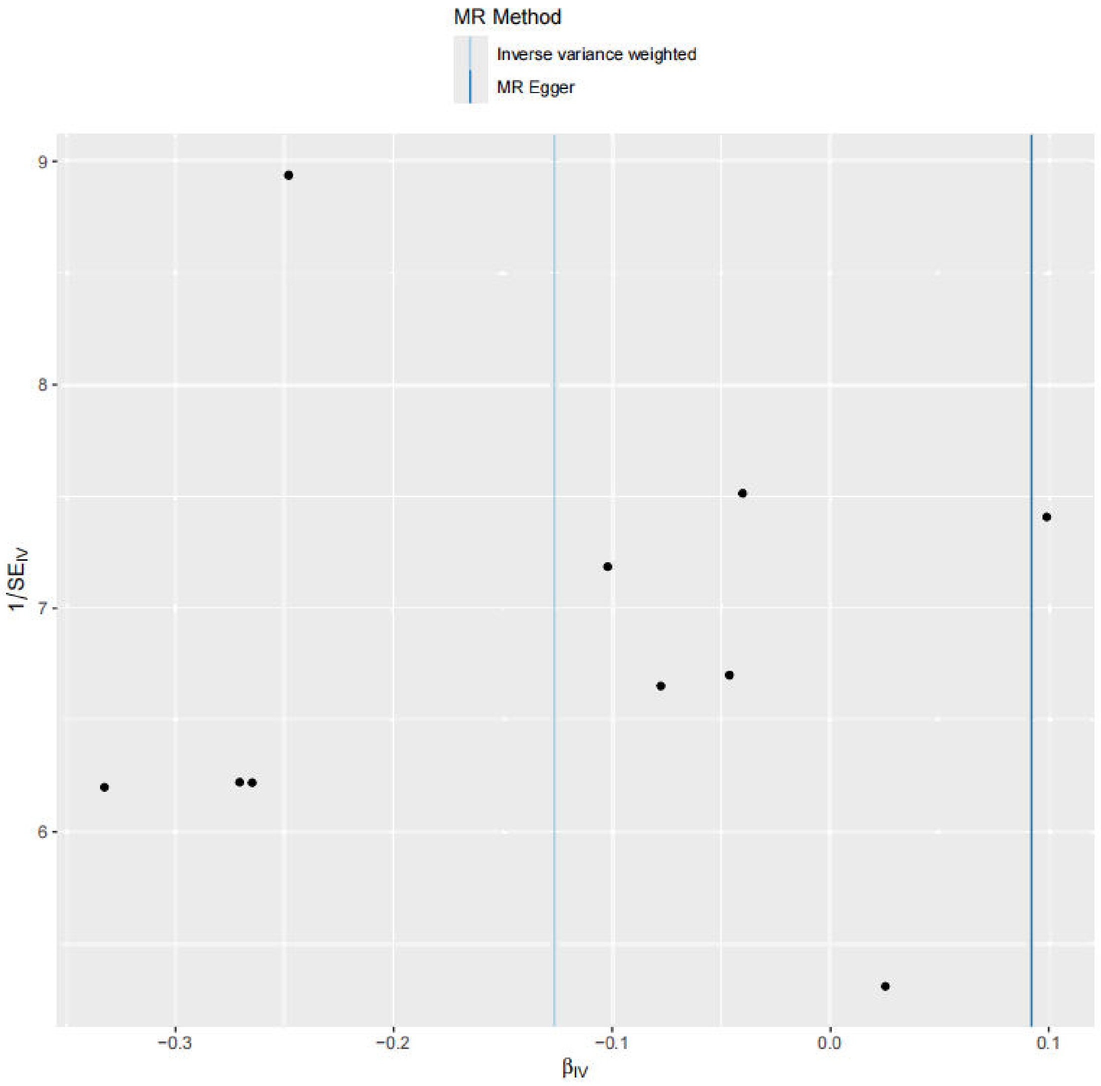

MR Method
Inverse variance weighted
MR Egger
9
8
7
6
1/SE_IV
-0.3
-0.2
-0.1
0.0
0.1
β_IV

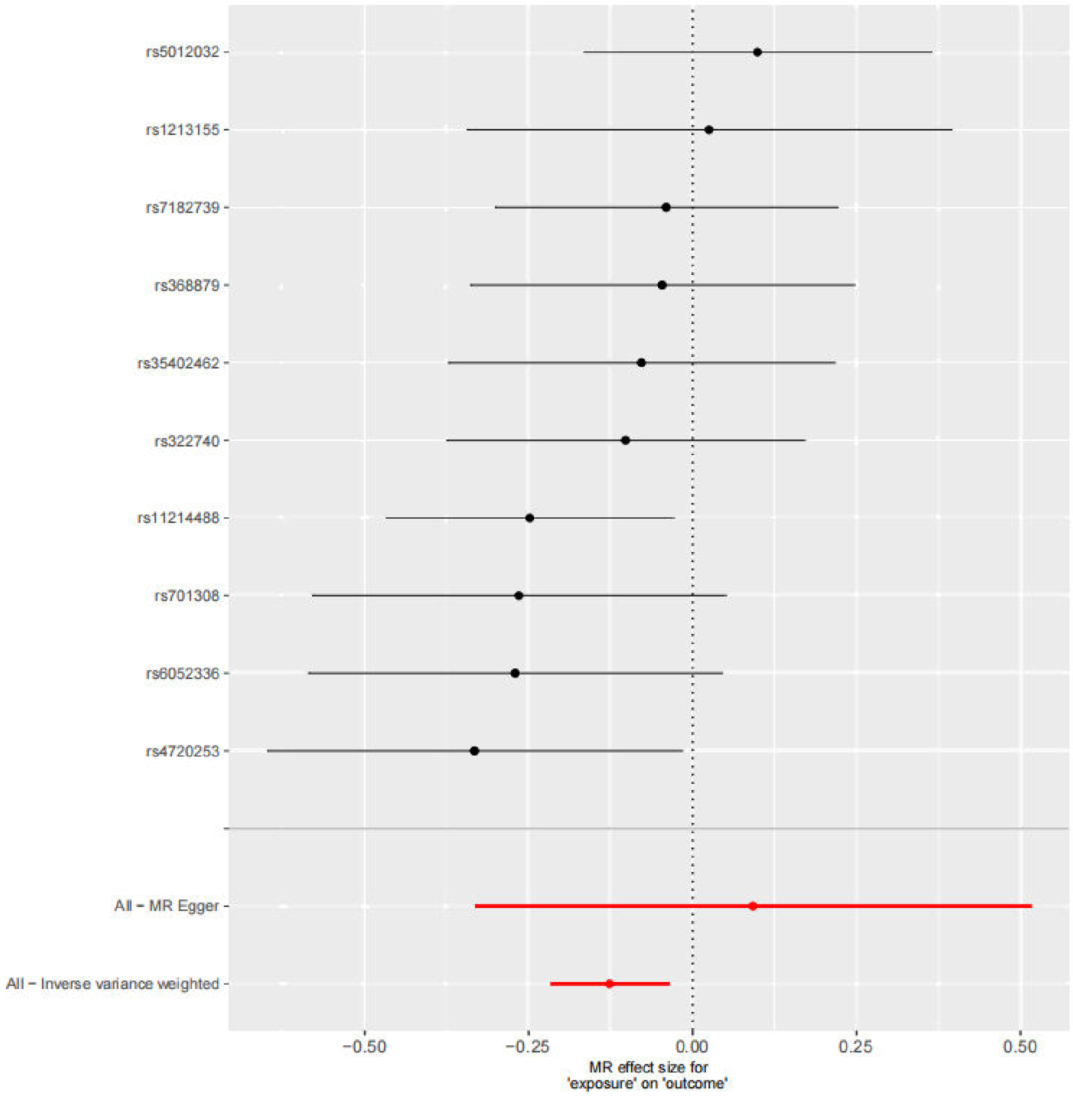
rs5012032
rs1213155
rs7182739
rs368879
rs35402462
rs322740
rs11214488
rs701308
rs6052336
rs4720253
All − MR Egger
All − Inverse variance weighted
−0.50
−0.25
0.00
0.25
0.50
MR effect size for
'exposure' on 'outcome'

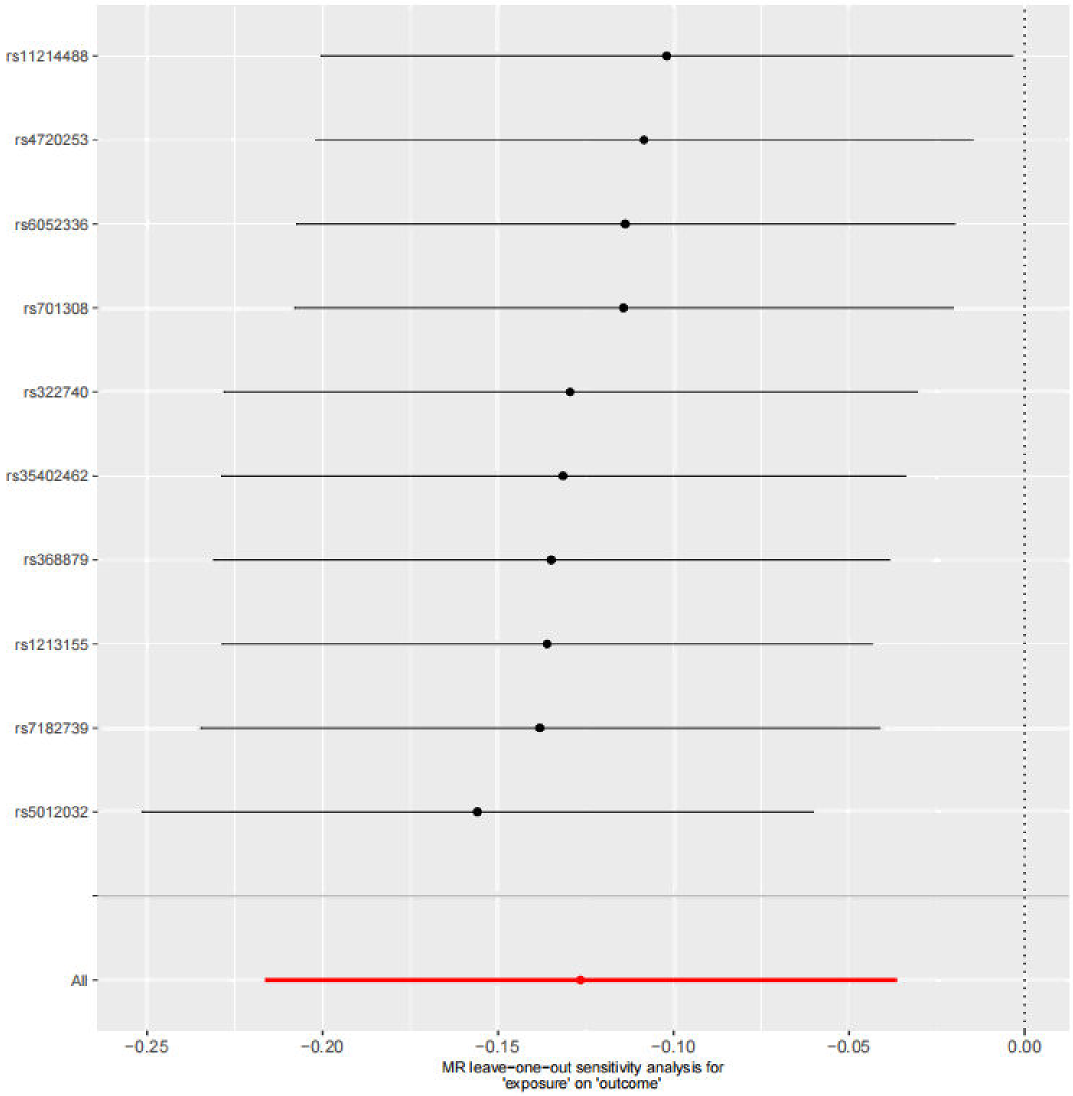
rs11214488
rs4720253
rs6052336
rs701308
rs322740
rs35402462
rs368879
rs1213155
rs7182739
rs5012032
All
−0.25
−0.20
−0.15
−0.10
−0.05
0.00
MR leave−one−out sensitivity analysis for
'exposure' on 'outcome'

GCST90257058

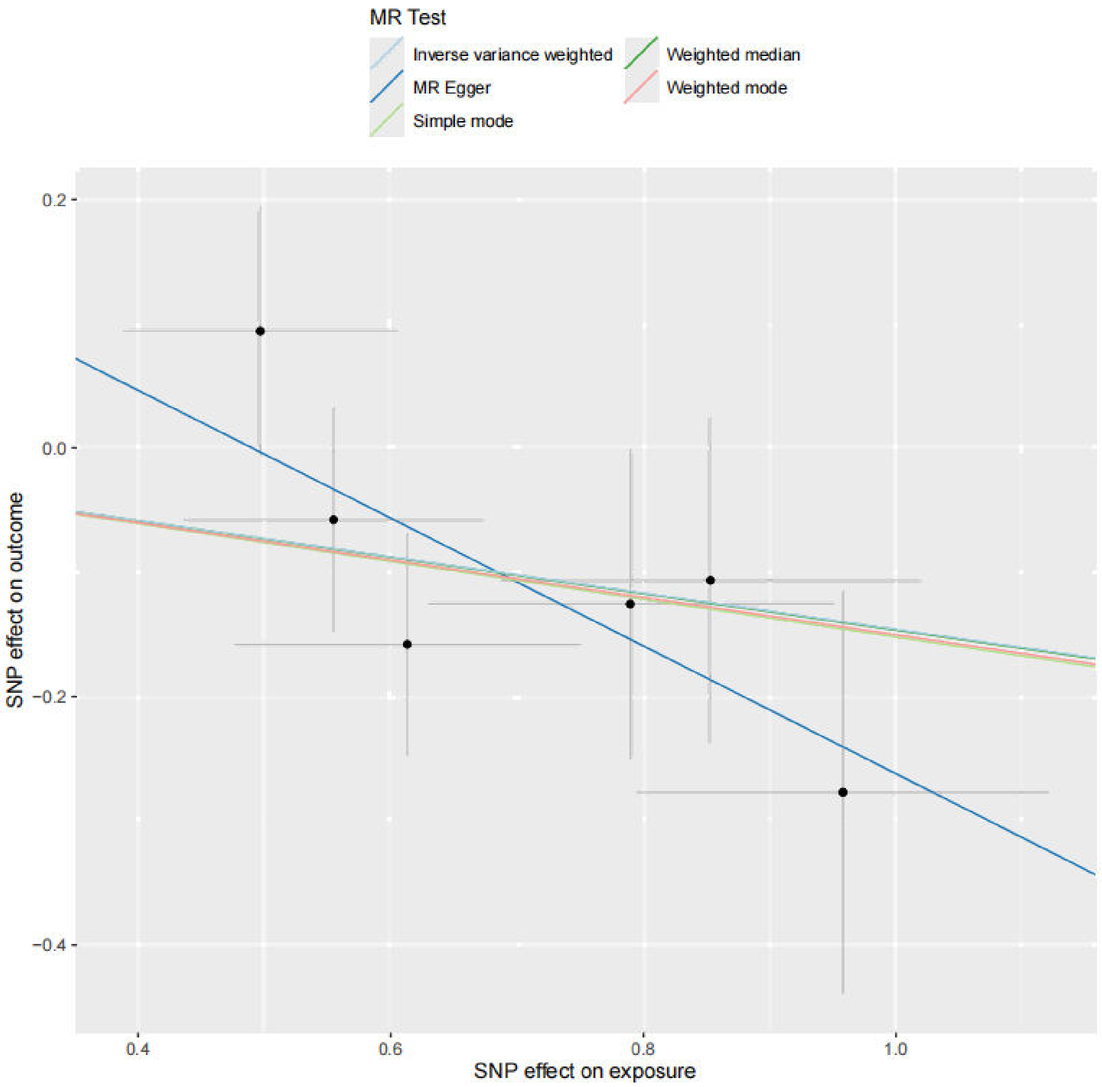

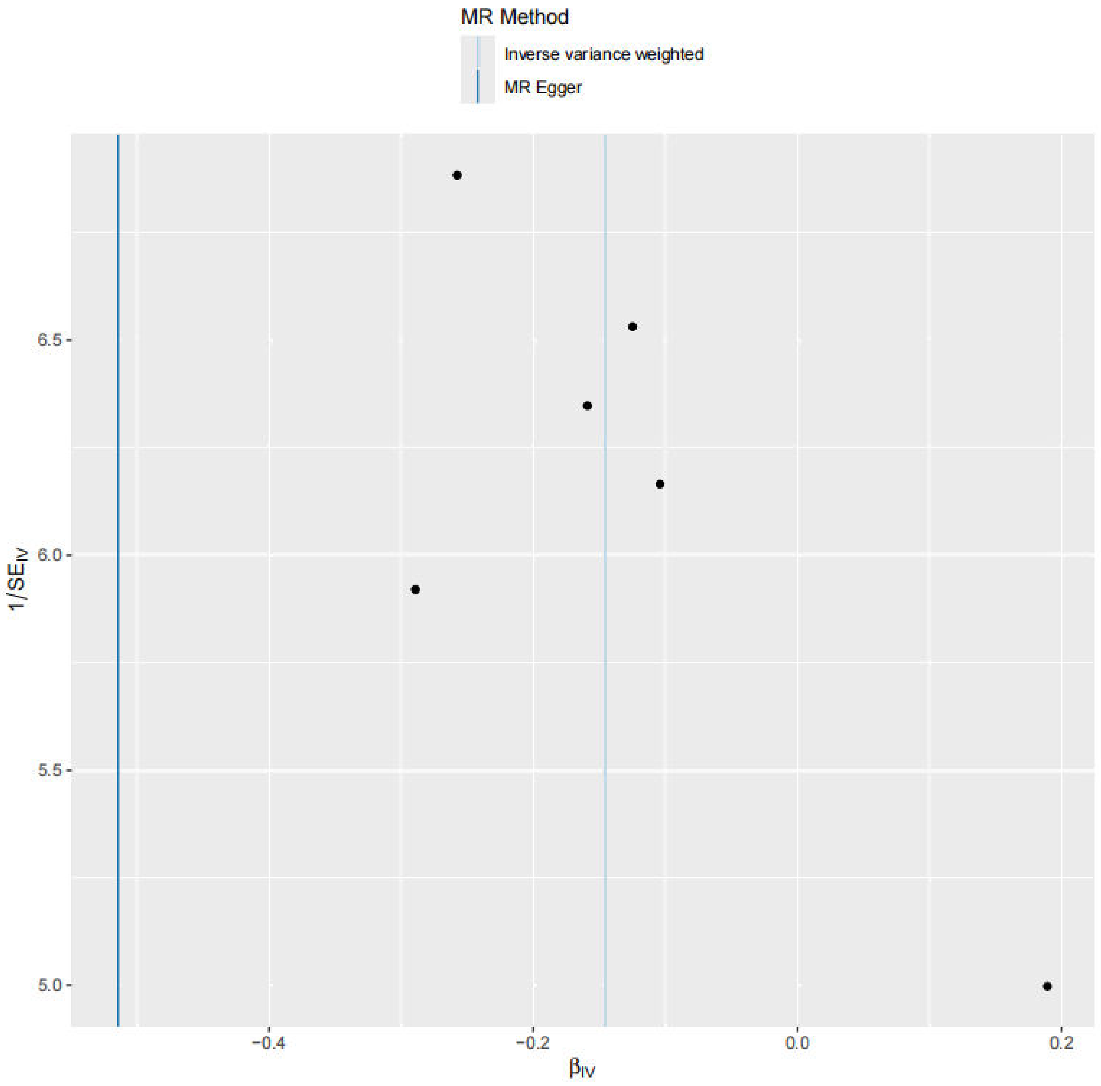

MR Method
Inverse variance weighted
MR Egger
6.5
6.0
5.5
5.0
1/SE_IV
−0.4
−0.2
0.0
0.2
β_IV

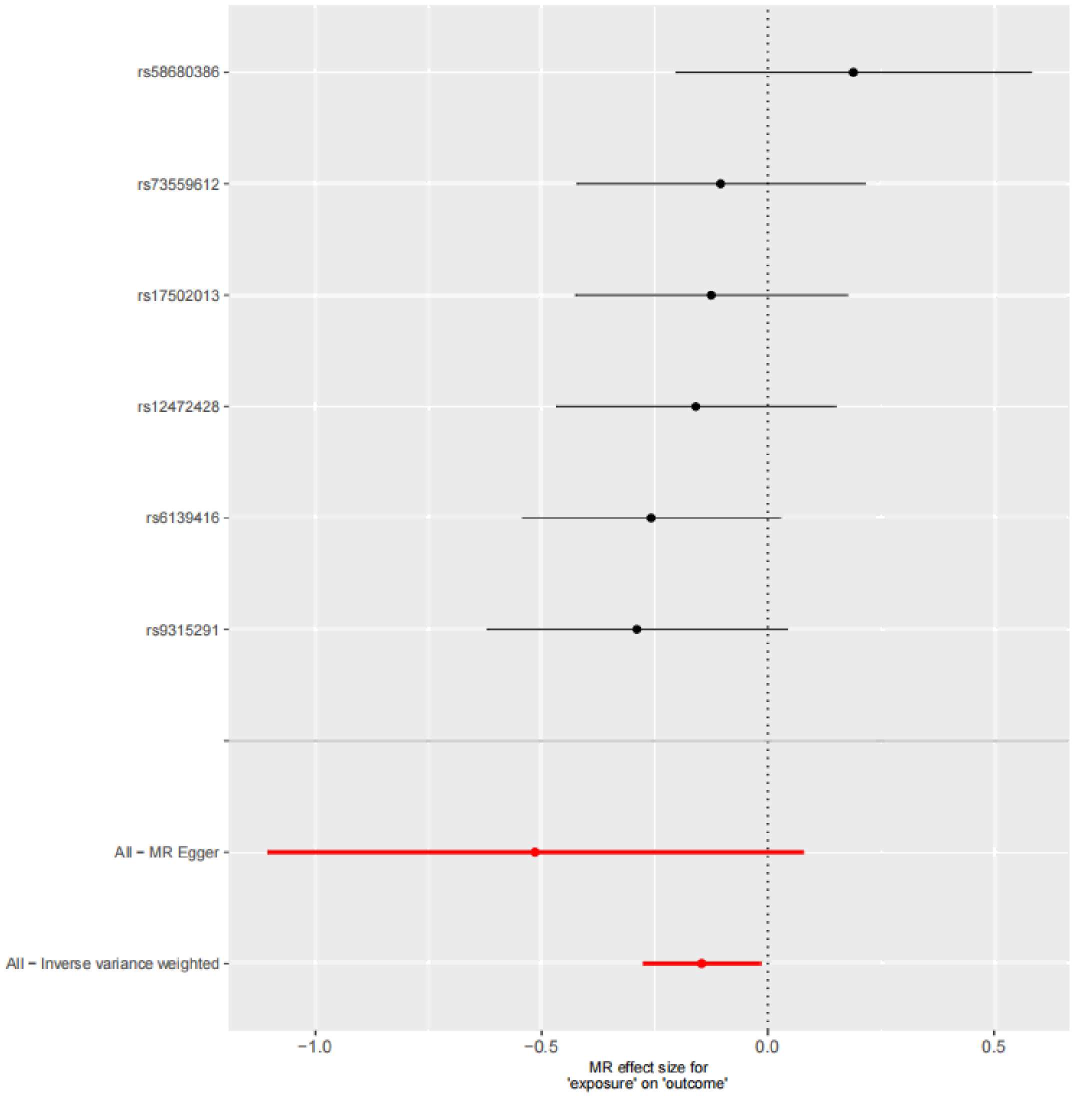
rs58680386
rs73559612
rs17502013
rs12472428
rs6139416
rs9315291
All − MR Egger
All − Inverse variance weighted
−1.0
−0.5
0.0
0.5
MR effect size for
'exposure' on 'outcome'

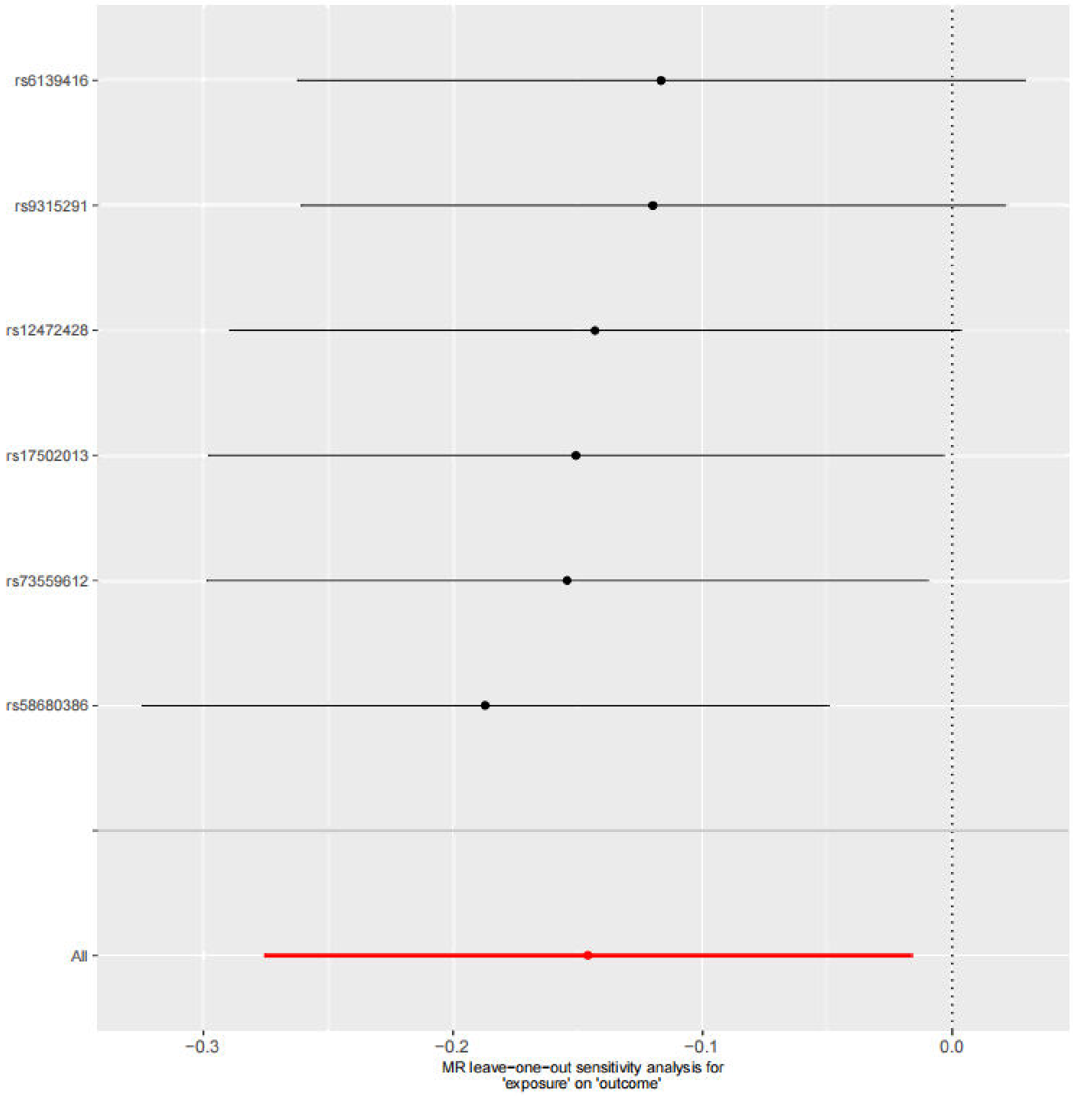

rs6139416
rs9315291
rs12472428
rs17502013
rs73559612
rs58680386
All
−0.3
−0.2
−0.1
0.0
MR leave−one−out sensitivity analysis for
'exposure' on 'outcome'

GCST90257079

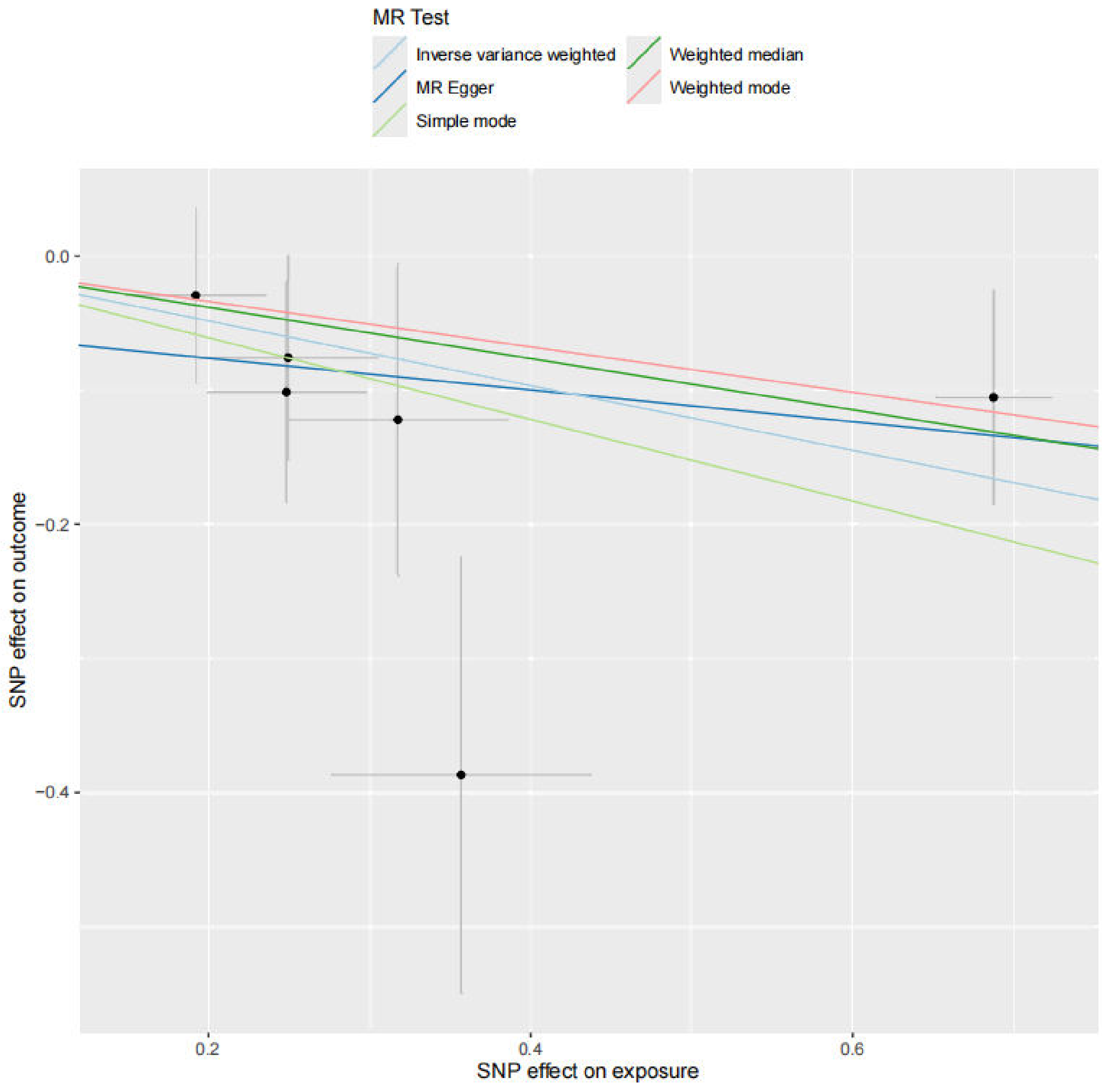

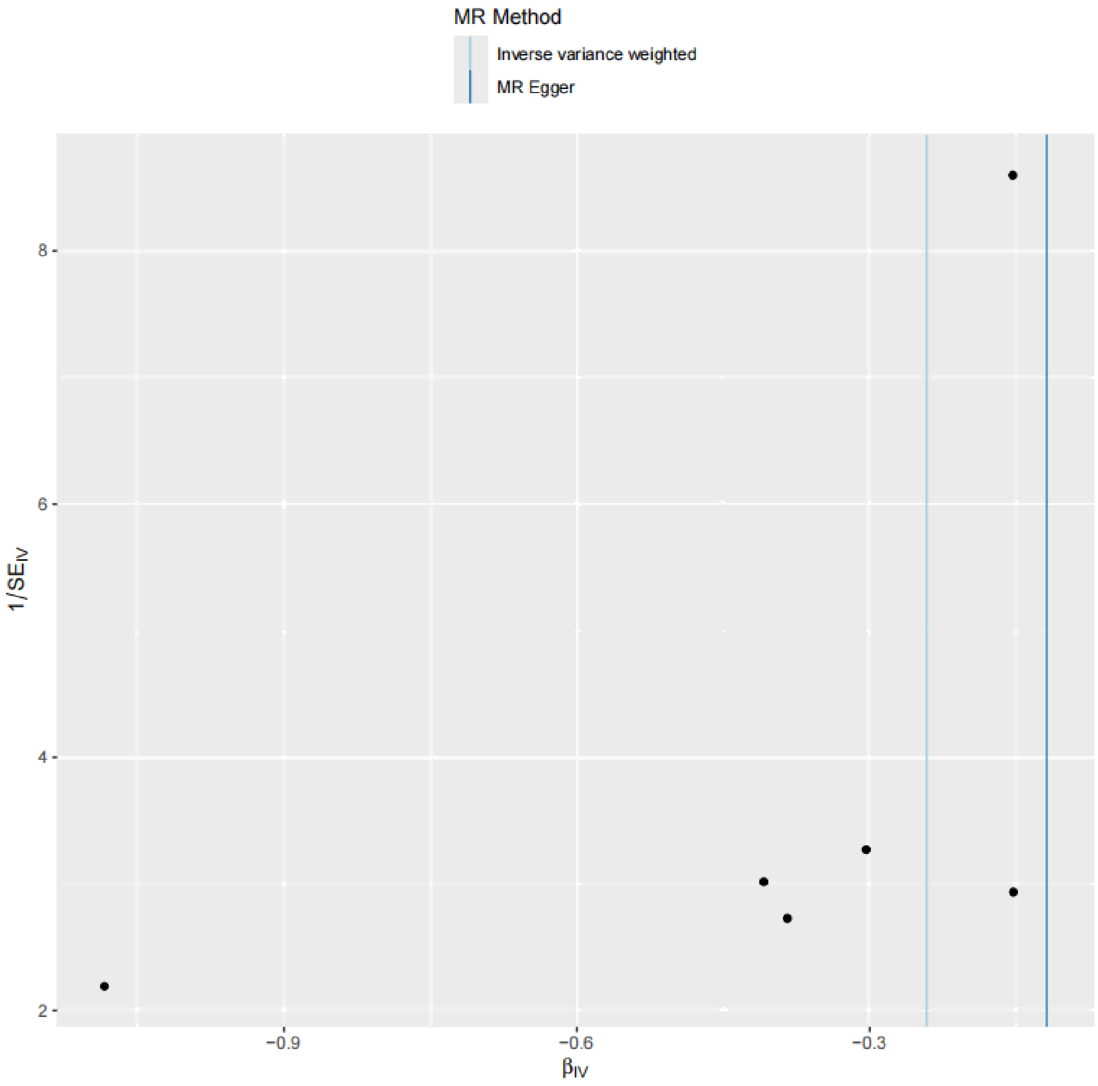
MR Method
Inverse variance weighted
MR Egger
8
6
4
2
1/SE_IV
−0.9
−0.6
−0.3
β_IV

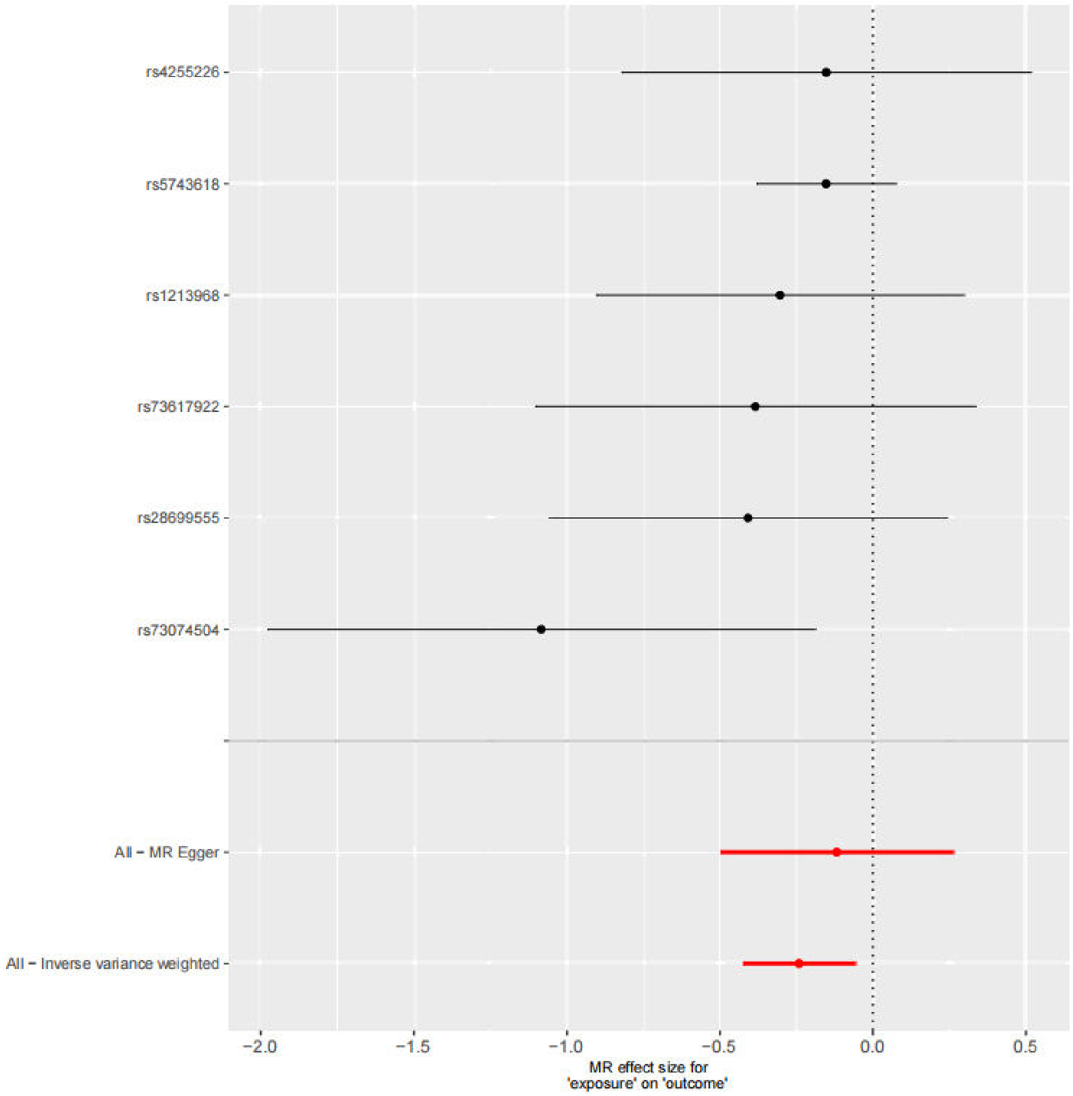

rs4255226
rs5743618
rs1213968
rs73617922
rs28699555
rs73074504
All − MR Egger
All − Inverse variance weighted
−2.0
−1.5
−1.0
−0.5
0.0
0.5
MR effect size for
'exposure' on 'outcome'

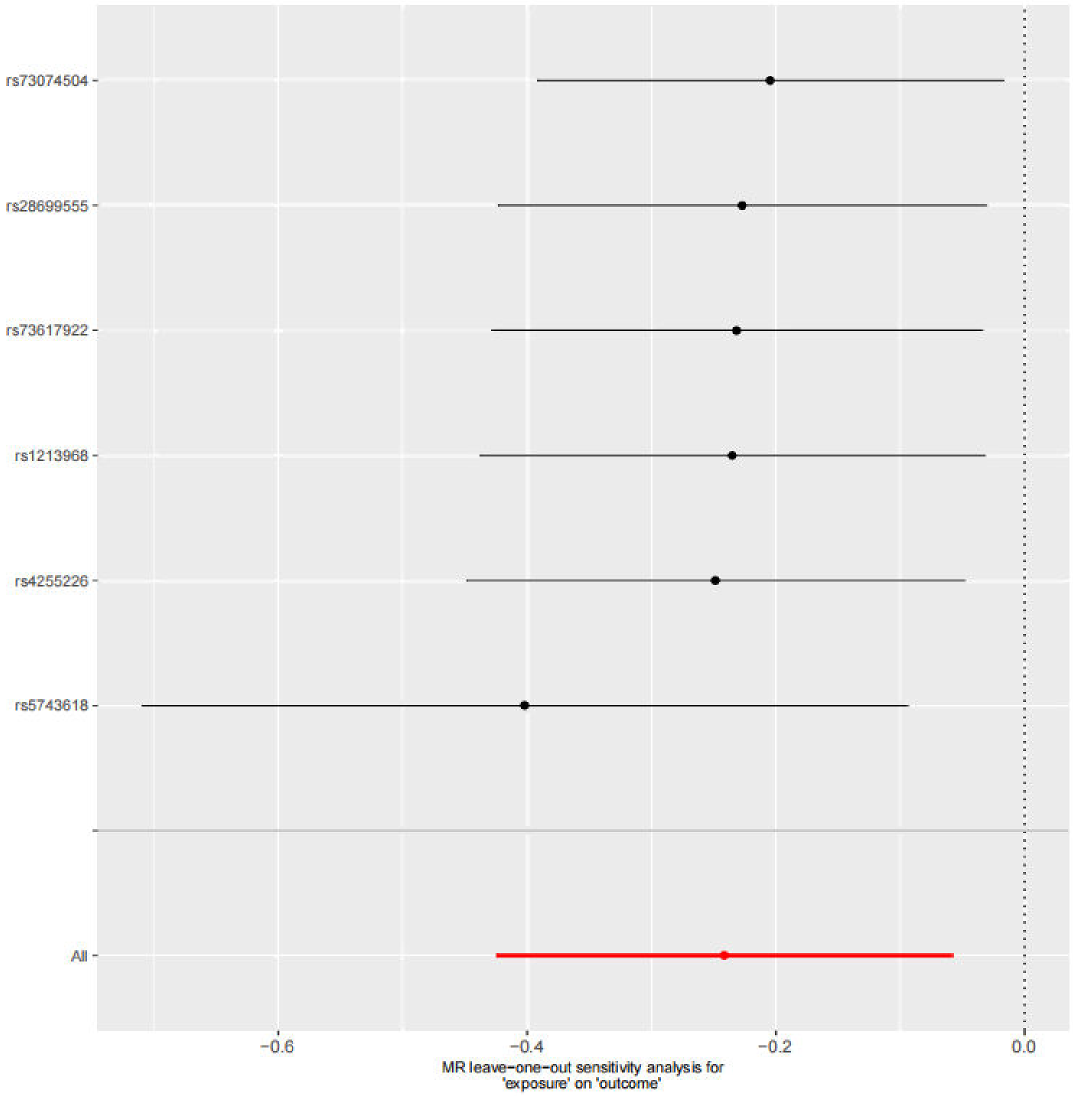

rs73074504
rs28699555
rs73617922
rs1213968
rs4255226
rs5743618
All
−0.6
−0.4
−0.2
0.0
MR leave−one−out sensitivity analysis for
'exposure' on 'outcome'

GCST90257081

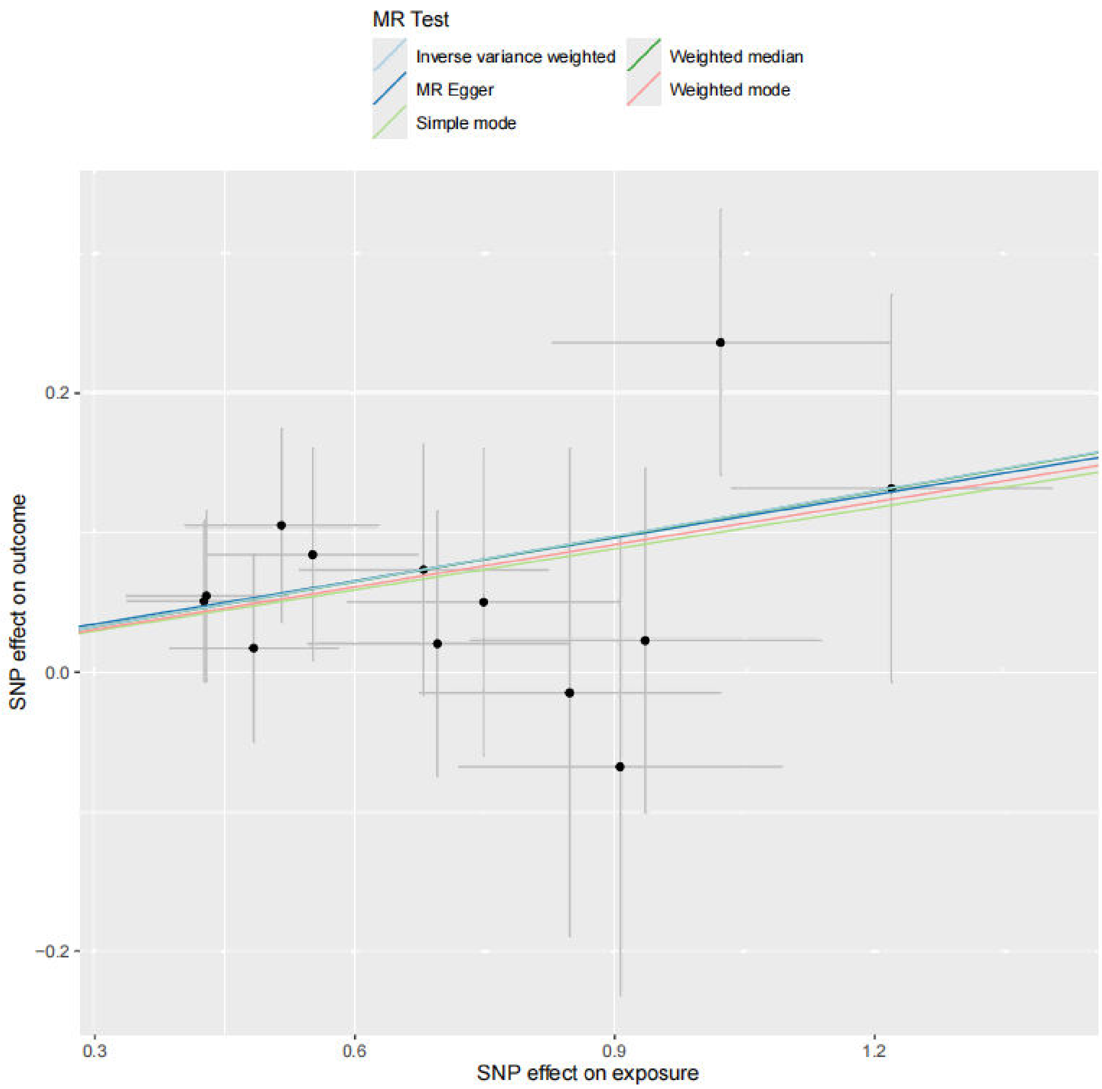

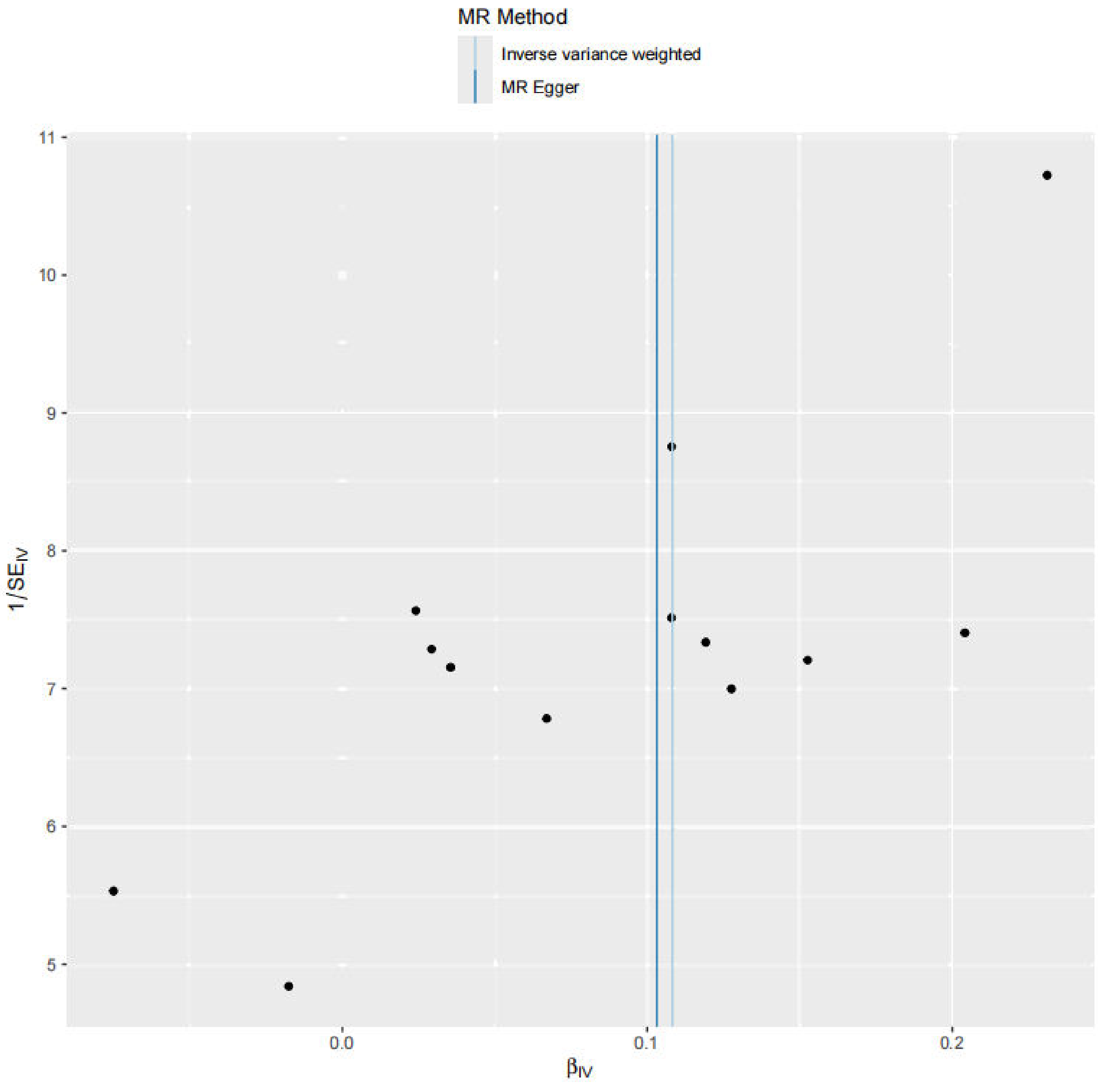

MR Method
Inverse variance weighted
MR Egger
11
10
9
8
7
6
5
1/SE_IV
0.0
0.1
0.2
β_IV

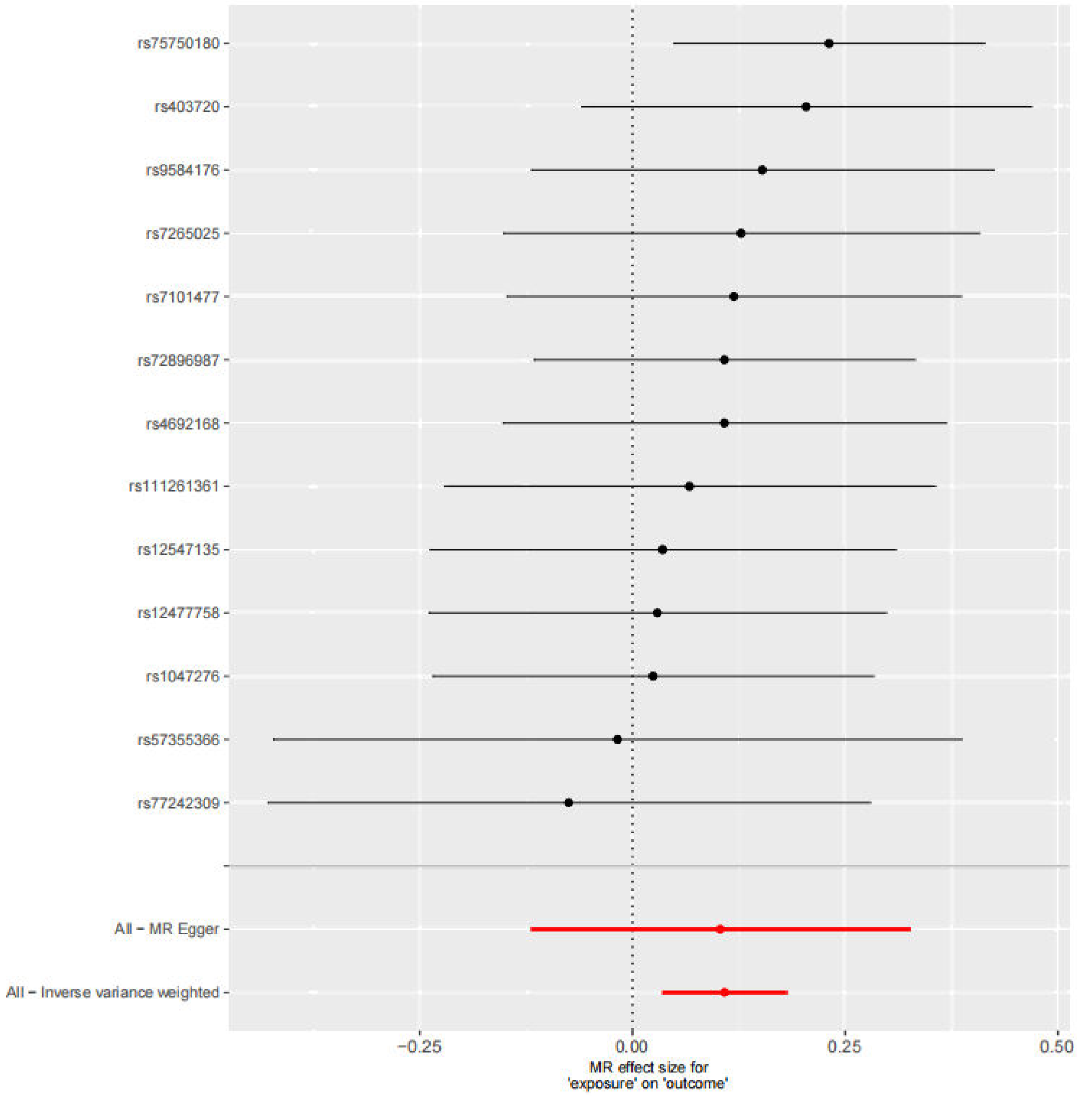
rs75750180
rs403720
rs9584176
rs7265025
rs7101477
rs72896987
rs4692168
rs111261361
rs12547135
rs12477758
rs1047276
rs57355366
rs77242309
All − MR Egger
All − Inverse variance weighted
−0.25
0.00
0.25
0.50
MR effect size for
'exposure' on 'outcome'

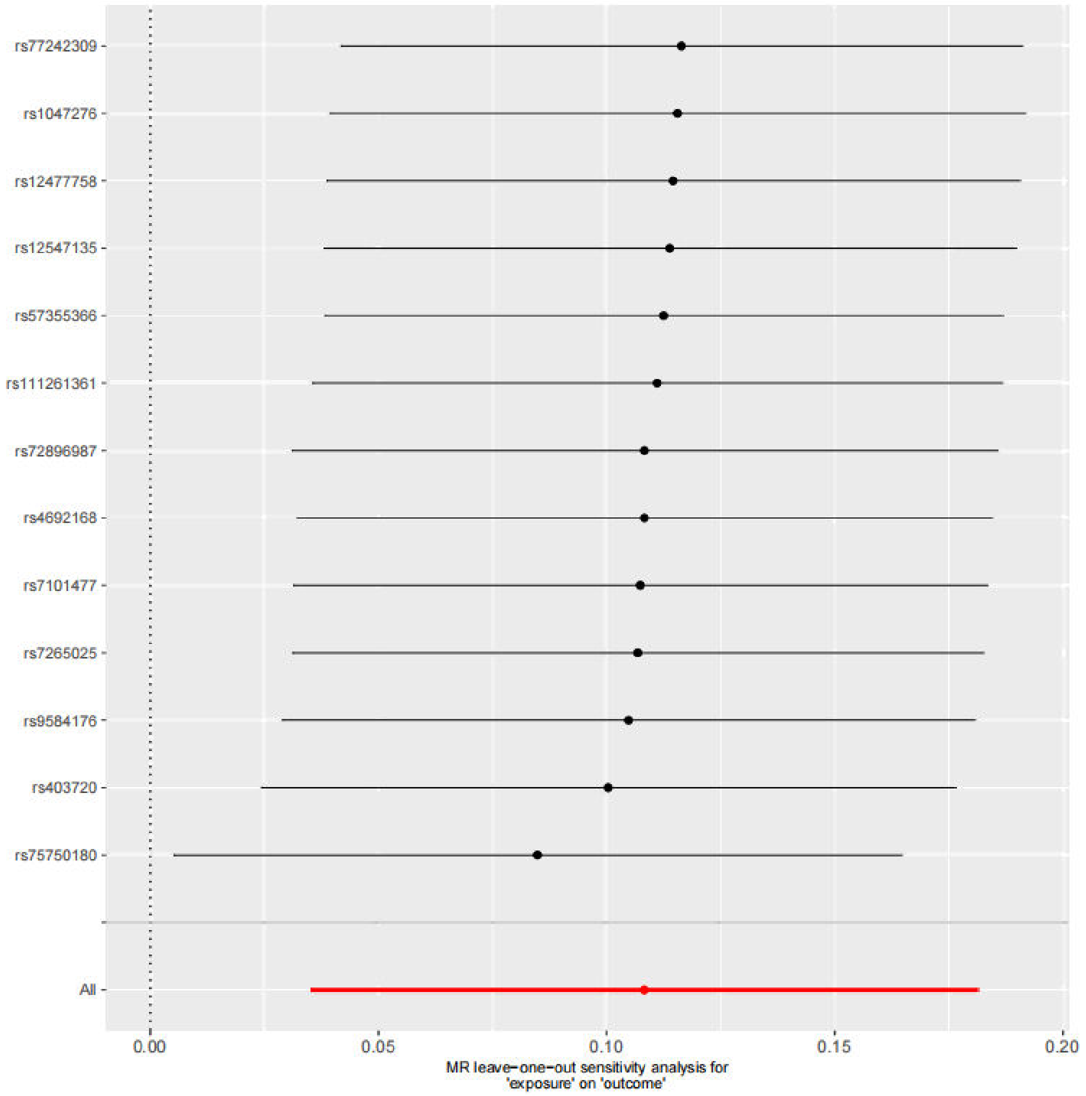

rs77242309
rs1047276
rs12477758
rs12547135
rs57355366
rs111261361
rs72896987
rs4692168
rs7101477
rs7265025
rs9584176
rs403720
rs75750180
All
0.00
0.05
0.10
0.15
0.20
MR leave−one−out sensitivity analysis for
'exposure' on 'outcome'

GCST90257035

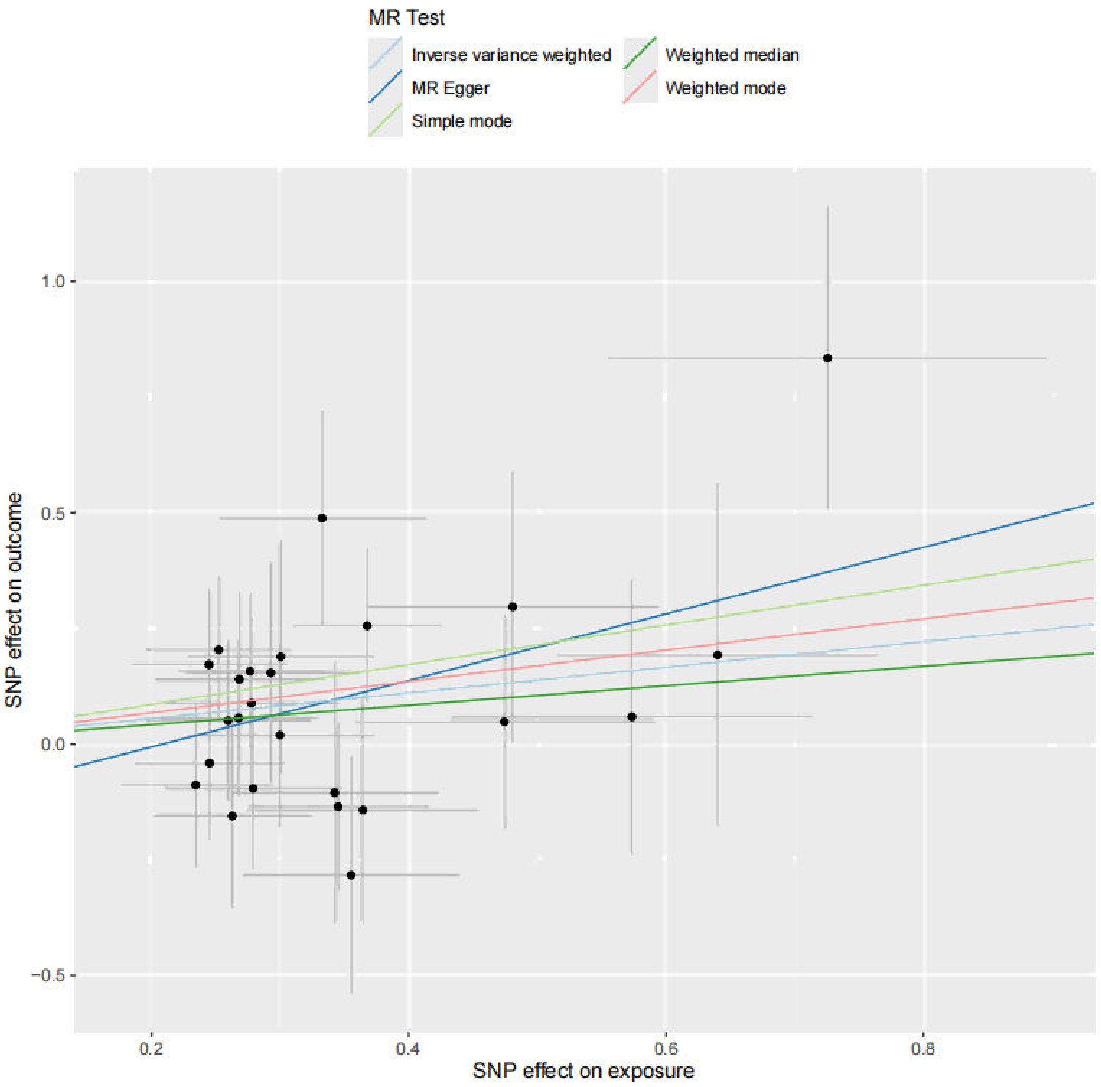

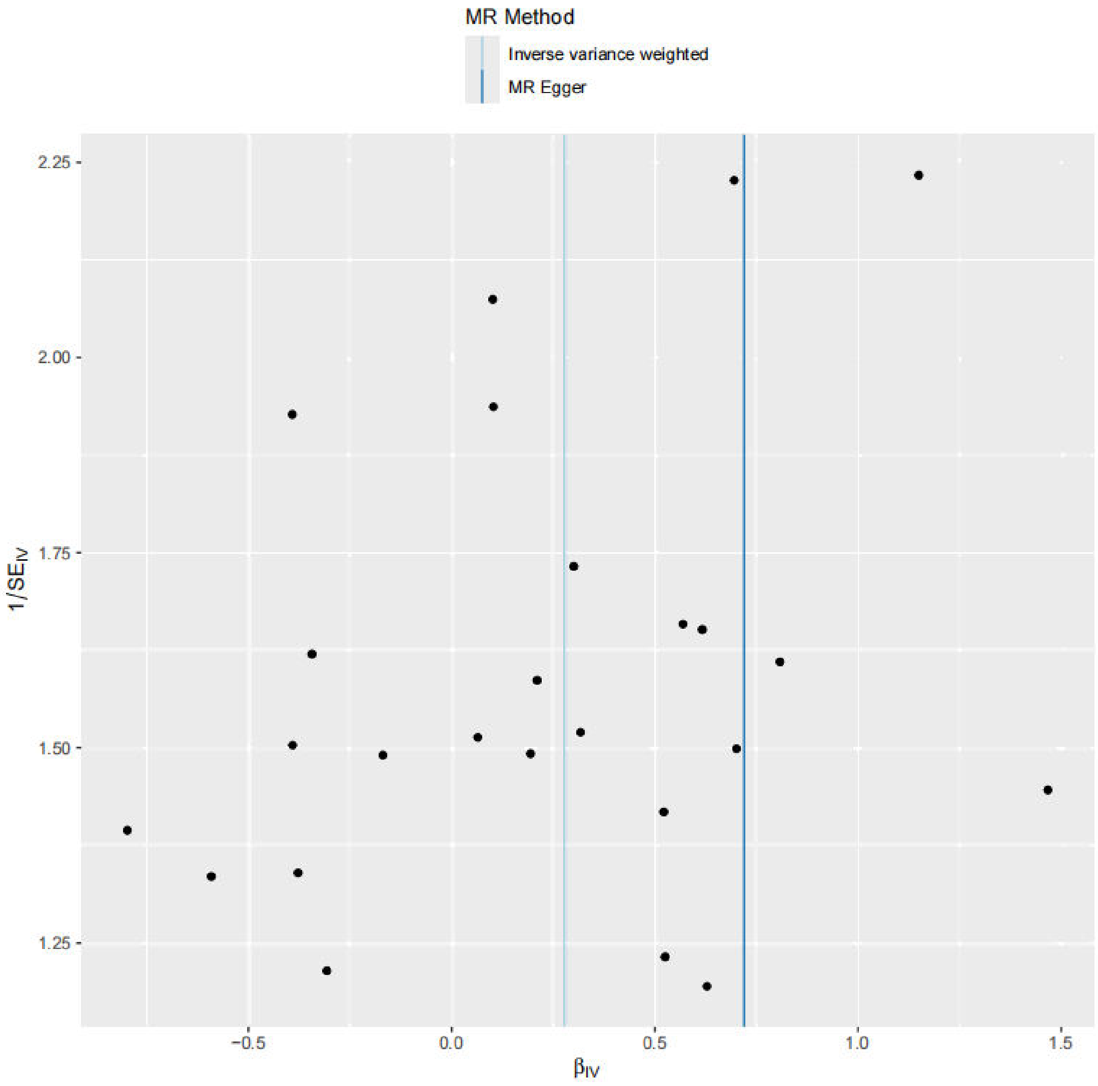

MR Method
Inverse variance weighted
MR Egger
2.25
2.00
1.75
1.50
1.25
1/SE$_{IV}$
−0.5
0.0
0.5
1.0
1.5
β$_{IV}$

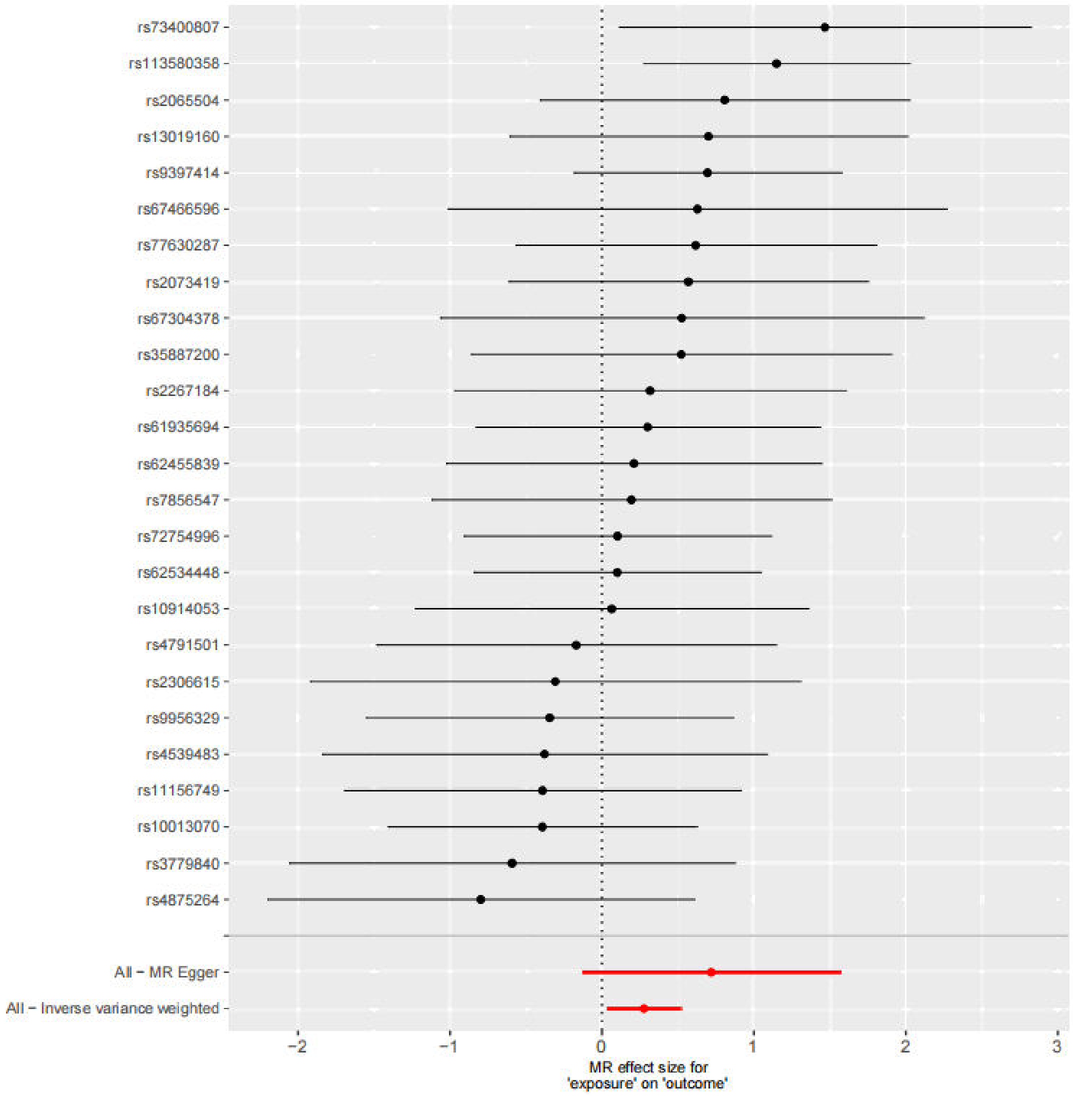
rs73400807
rs113580358
rs2065504
rs13019160
rs9397414
rs67466596
rs77630287
rs2073419
rs67304378
rs35887200
rs2267184
rs61935694
rs62455839
rs7856547
rs72754996
rs62534448
rs10914053
rs4791501
rs2306615
rs9956329
rs4539483
rs11156749
rs10013070
rs3779840
rs4875264
All − MR Egger
All − Inverse variance weighted
−2
−1
0
1
2
3
MR effect size for
'exposure' on 'outcome'

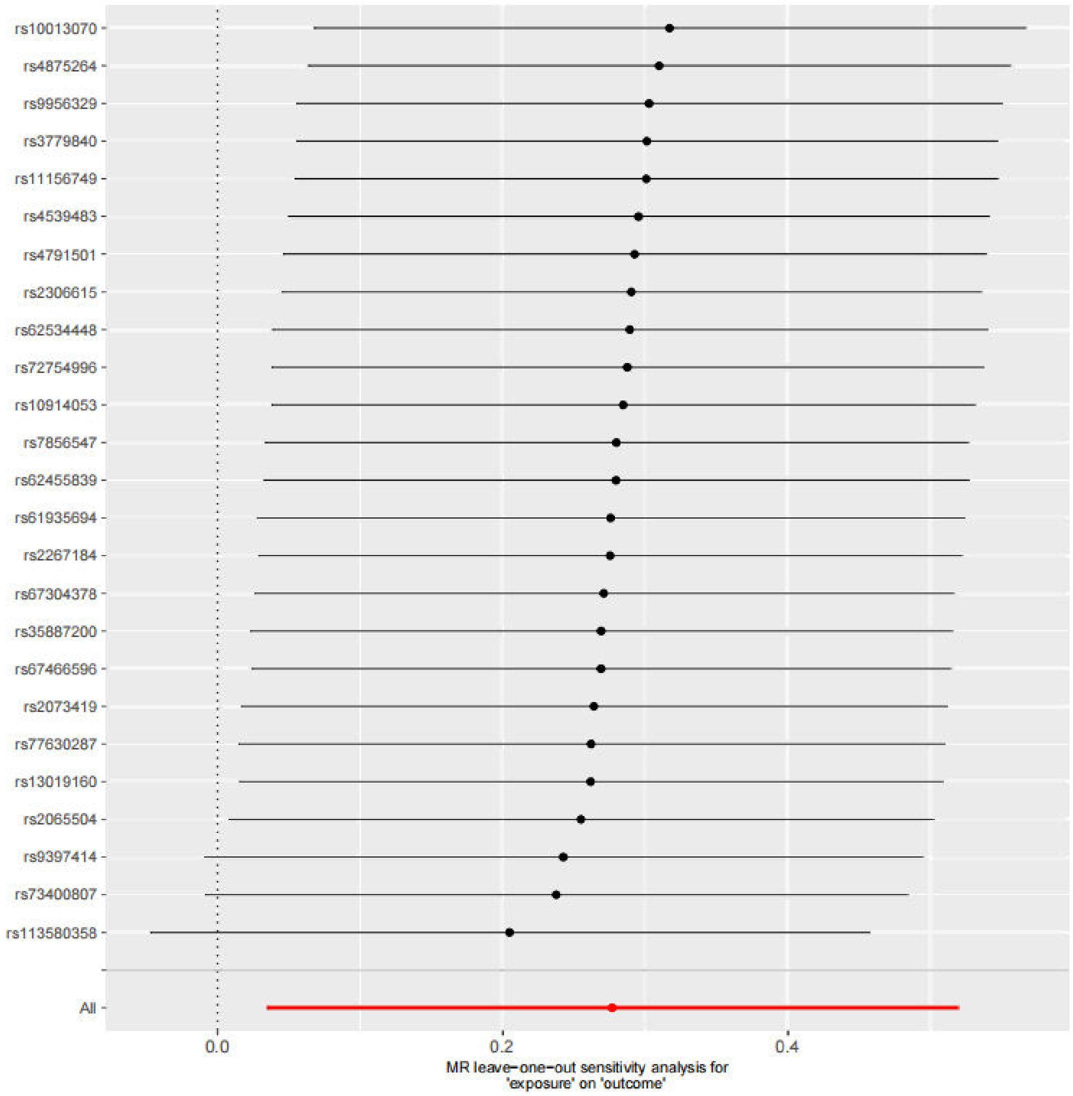
rs10013070
rs4875264
rs9956329
rs3779840
rs11156749
rs4539483
rs4791501
rs2306615
rs62534448
rs72754996
rs10914053
rs7856547
rs62455839
rs61935694
rs2267184
rs67304378
rs35887200
rs67466596
rs2073419
rs77630287
rs13019160
rs2065504
rs9397414
rs73400807
rs113580358
All
0.0
0.2
0.4
MR leave−one−out sensitivity analysis for
'exposure' on 'outcome'

GCST90257019

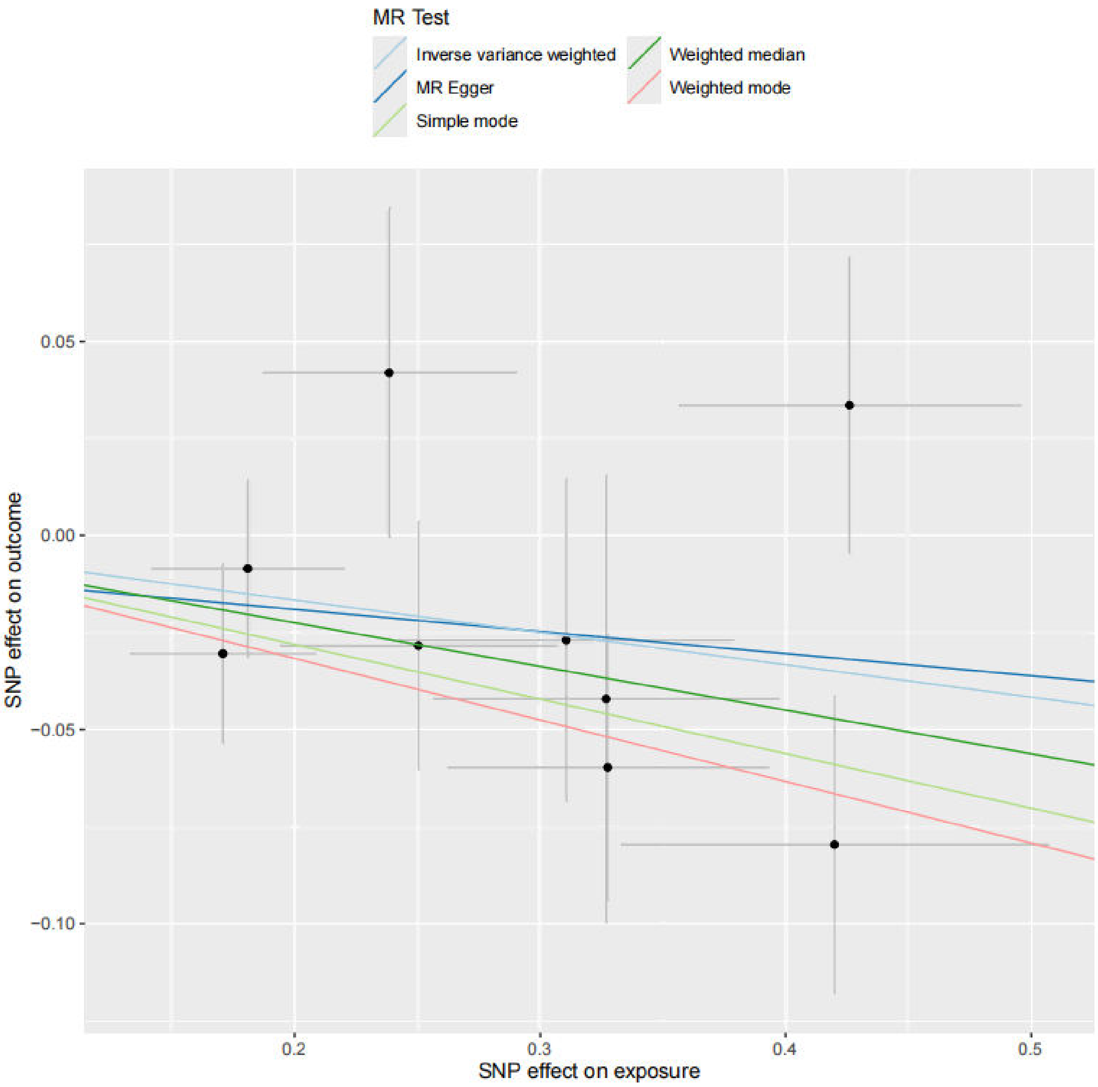

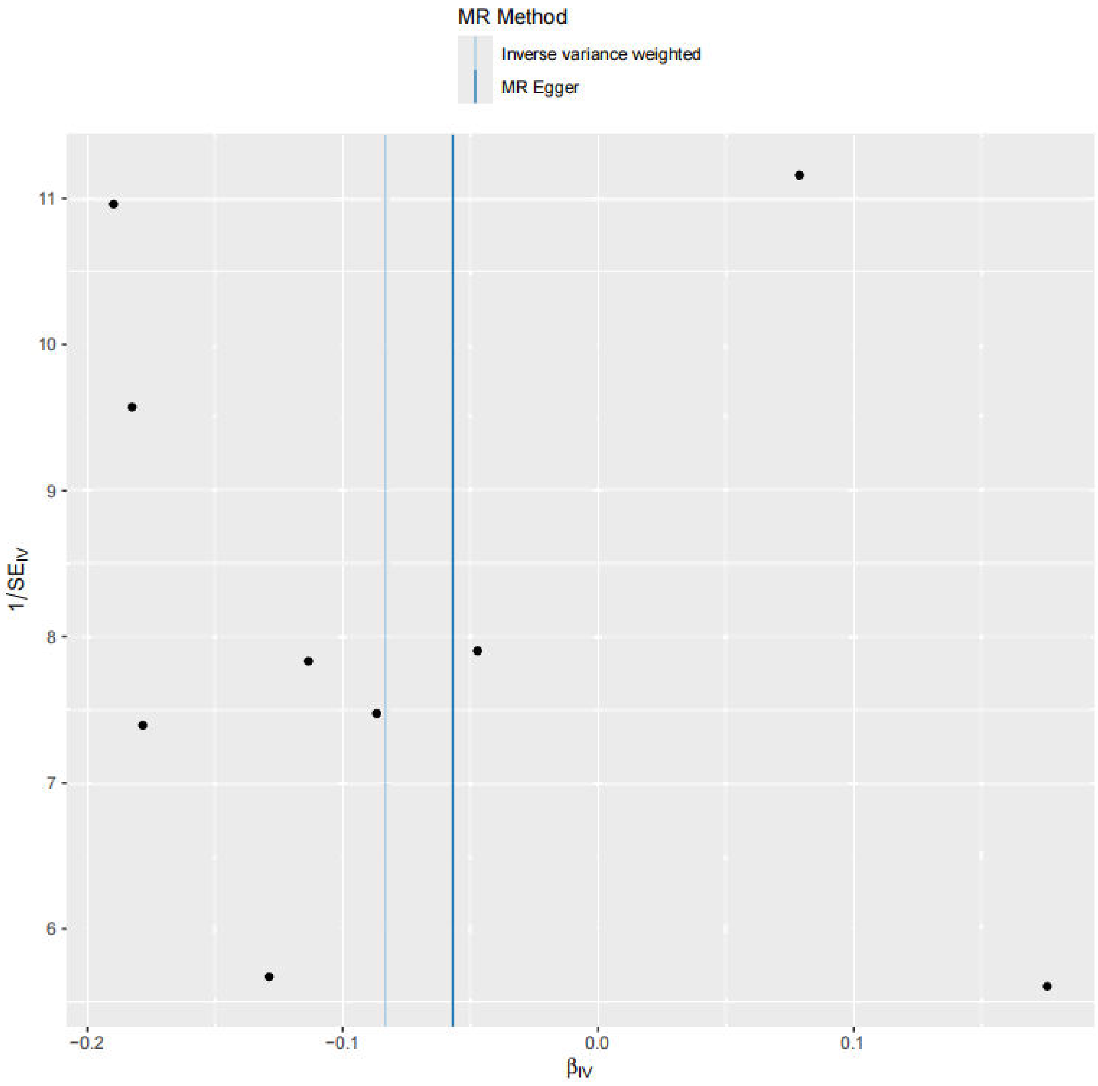

MR Method
Inverse variance weighted
MR Egger
11
10
9
8
7
6
1/SE$_{IV}$
−0.2
−0.1
0.0
0.1
β$_{IV}$

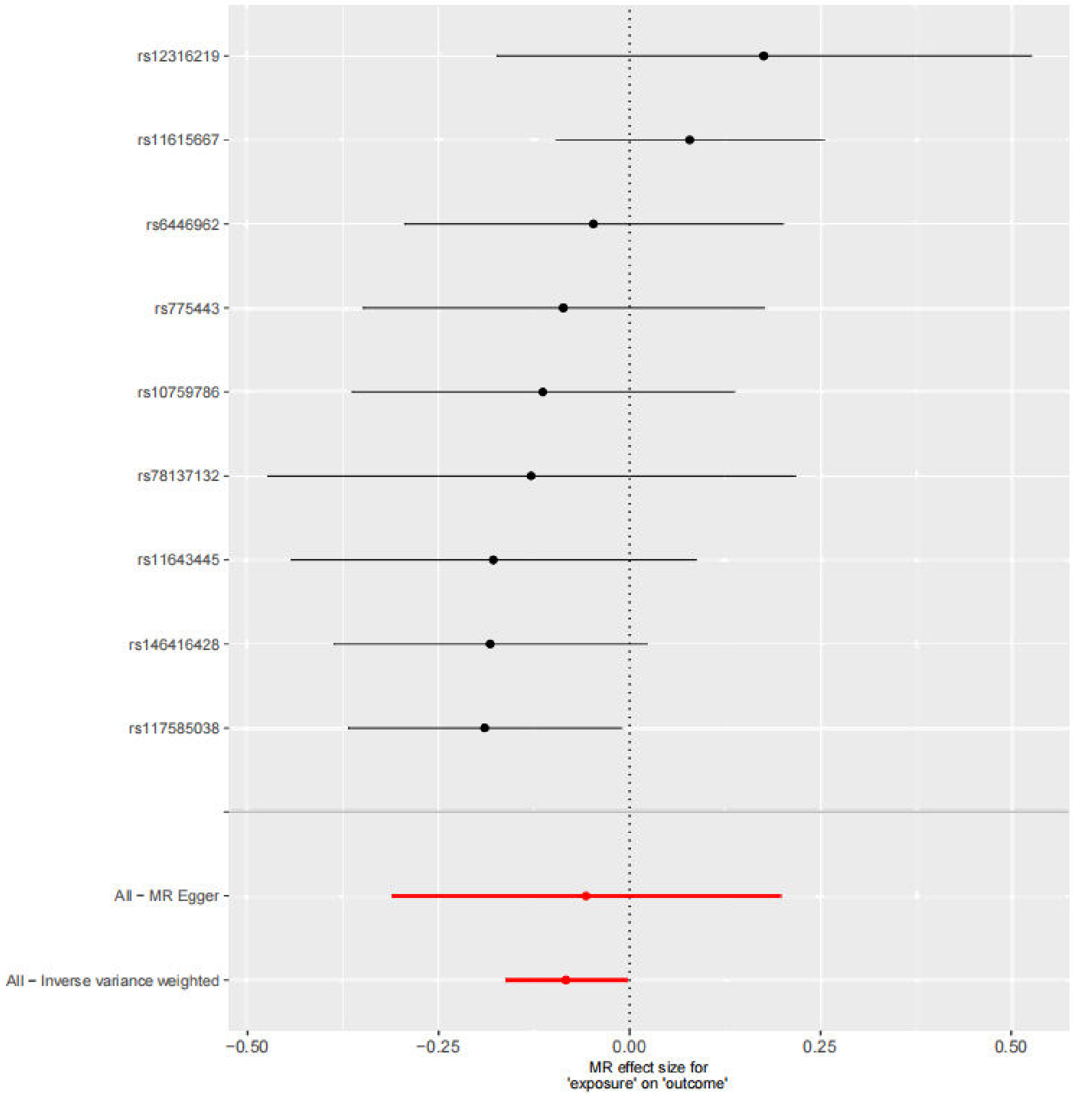

rs12316219
rs11615667
rs6446962
rs775443
rs10759786
rs78137132
rs11643445
rs146416428
rs117585038
All − MR Egger
All − Inverse variance weighted
−0.50
−0.25
0.00
0.25
0.50
MR effect size for
'exposure' on 'outcome'

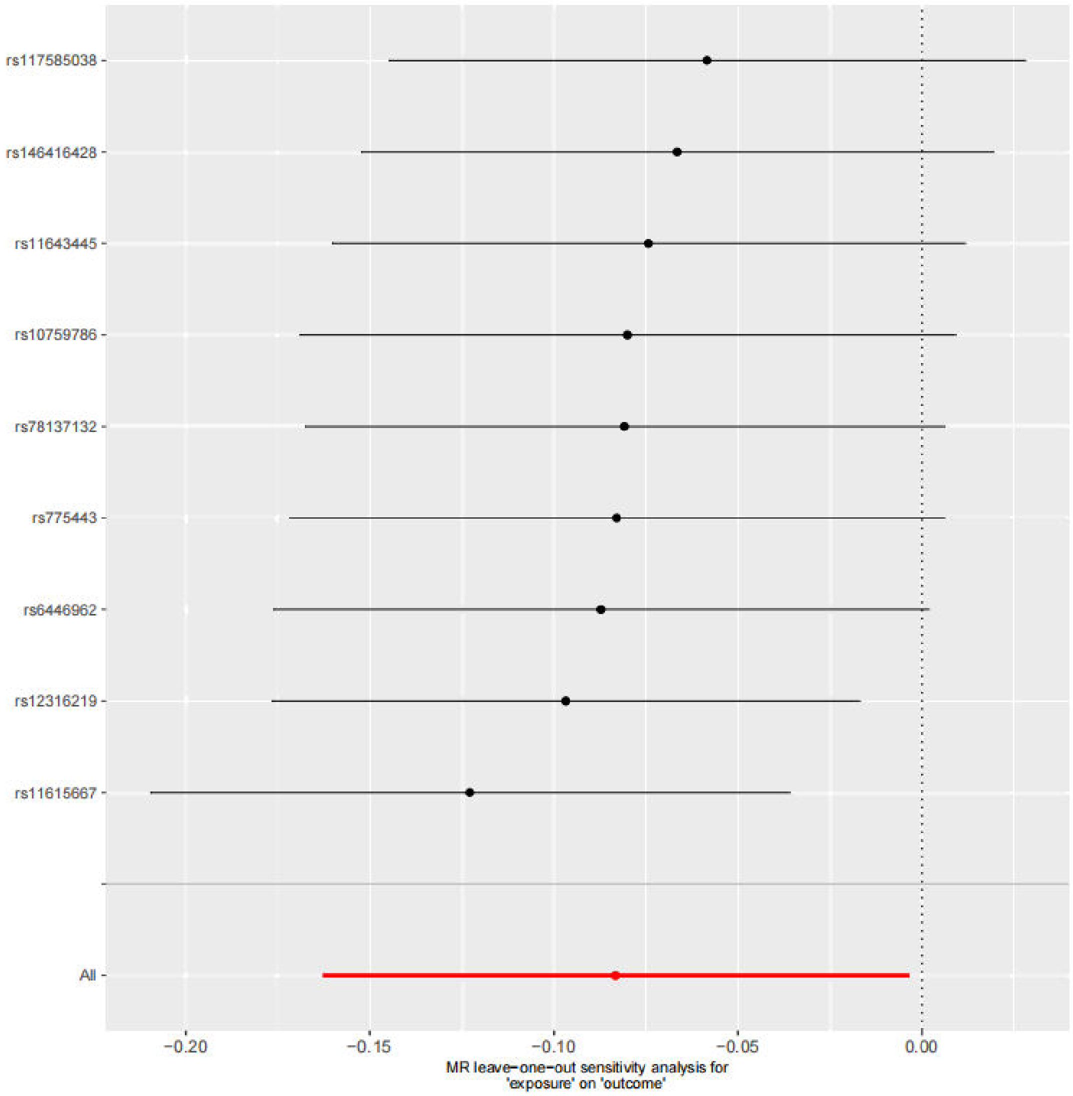

rs117585038
rs146416428
rs11643445
rs10759786
rs78137132
rs775443
rs6446962
rs12316219
rs11615667
All
−0.20
−0.15
−0.10
−0.05
0.00
MR leave−one−out sensitivity analysis for
'exposure' on 'outcome'

GCST90257024

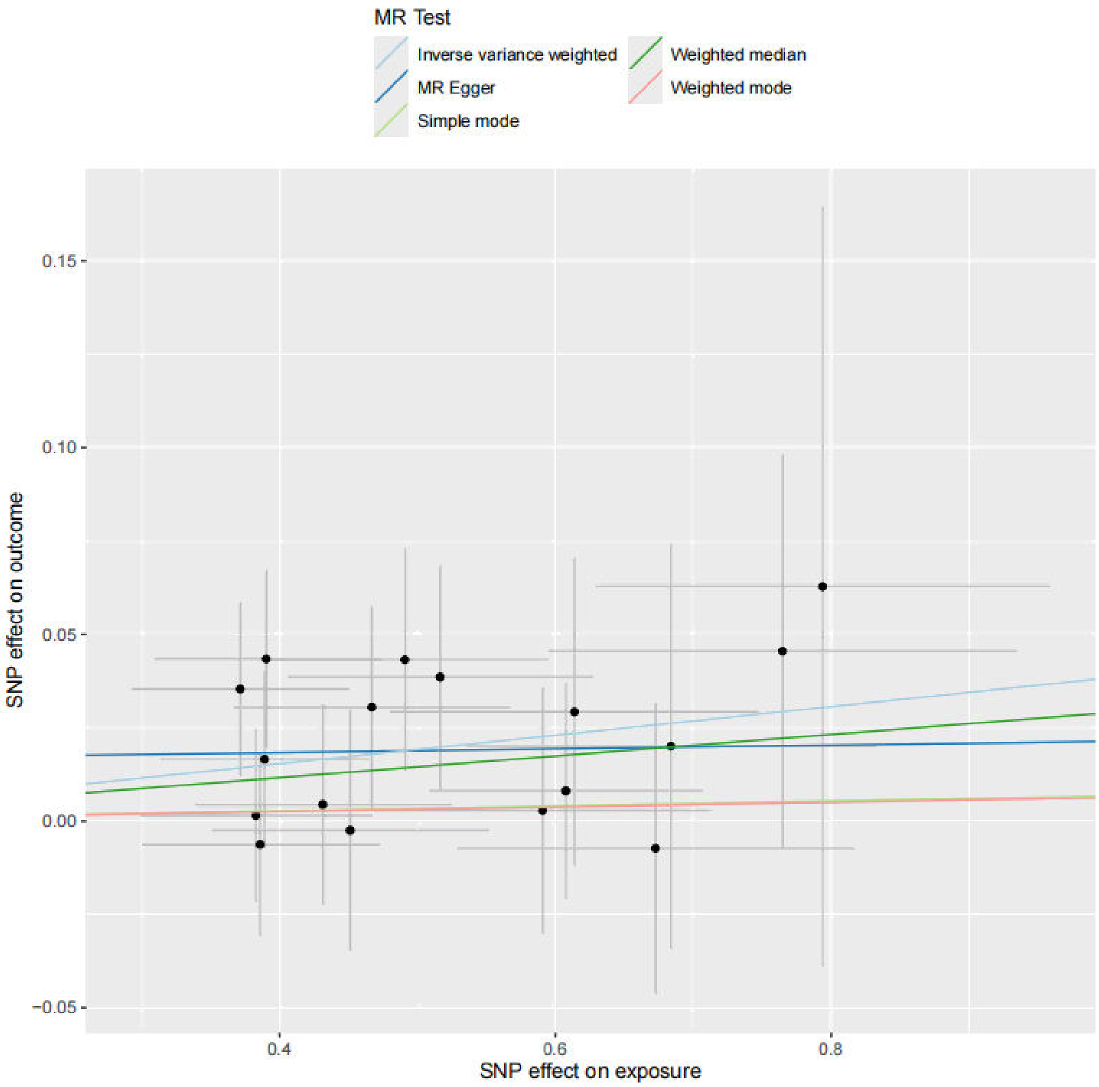

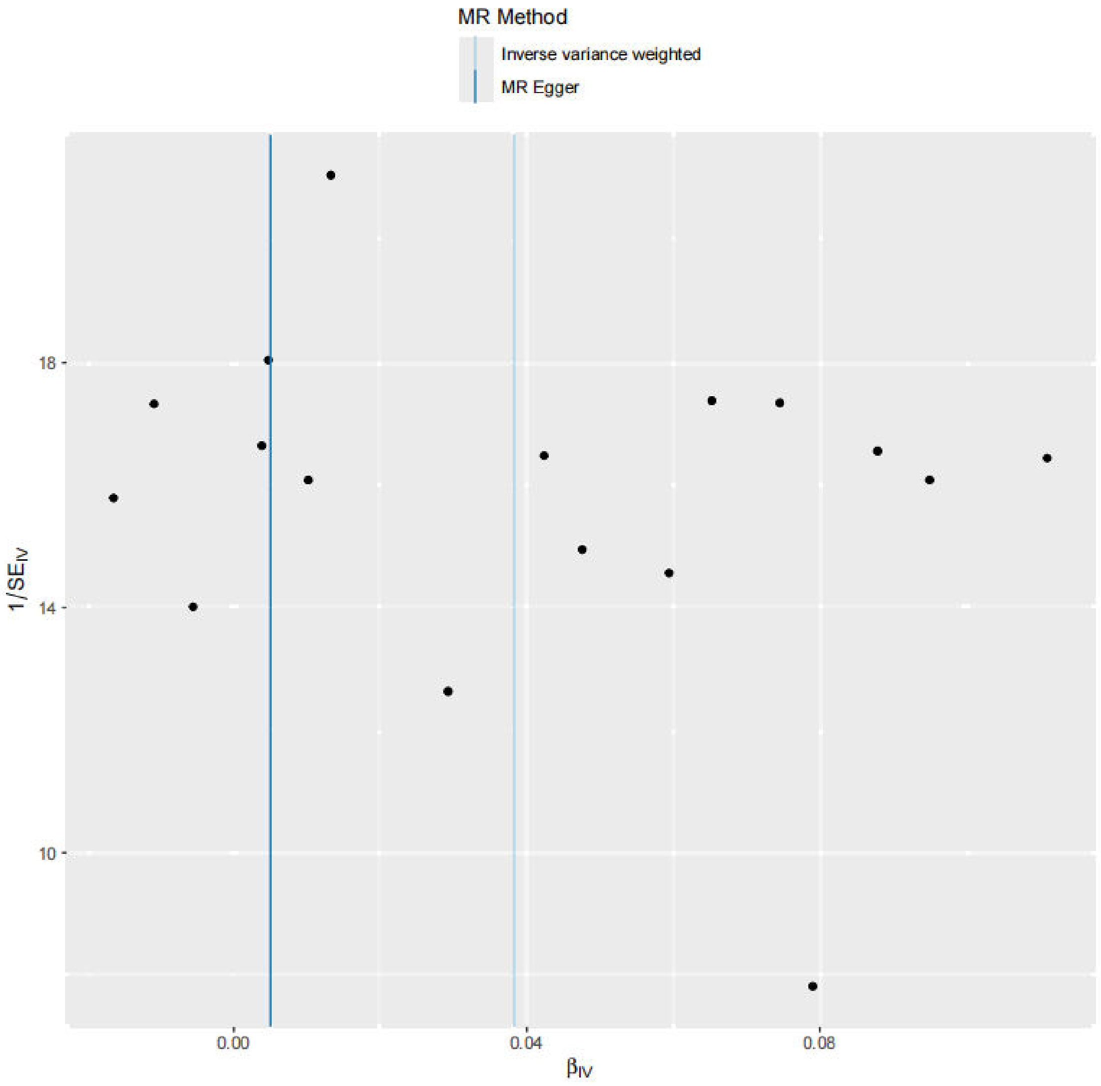

MR Method
Inverse variance weighted
MR Egger
18
14
10
1/SEIV
0.00
0.04
0.08
βIV

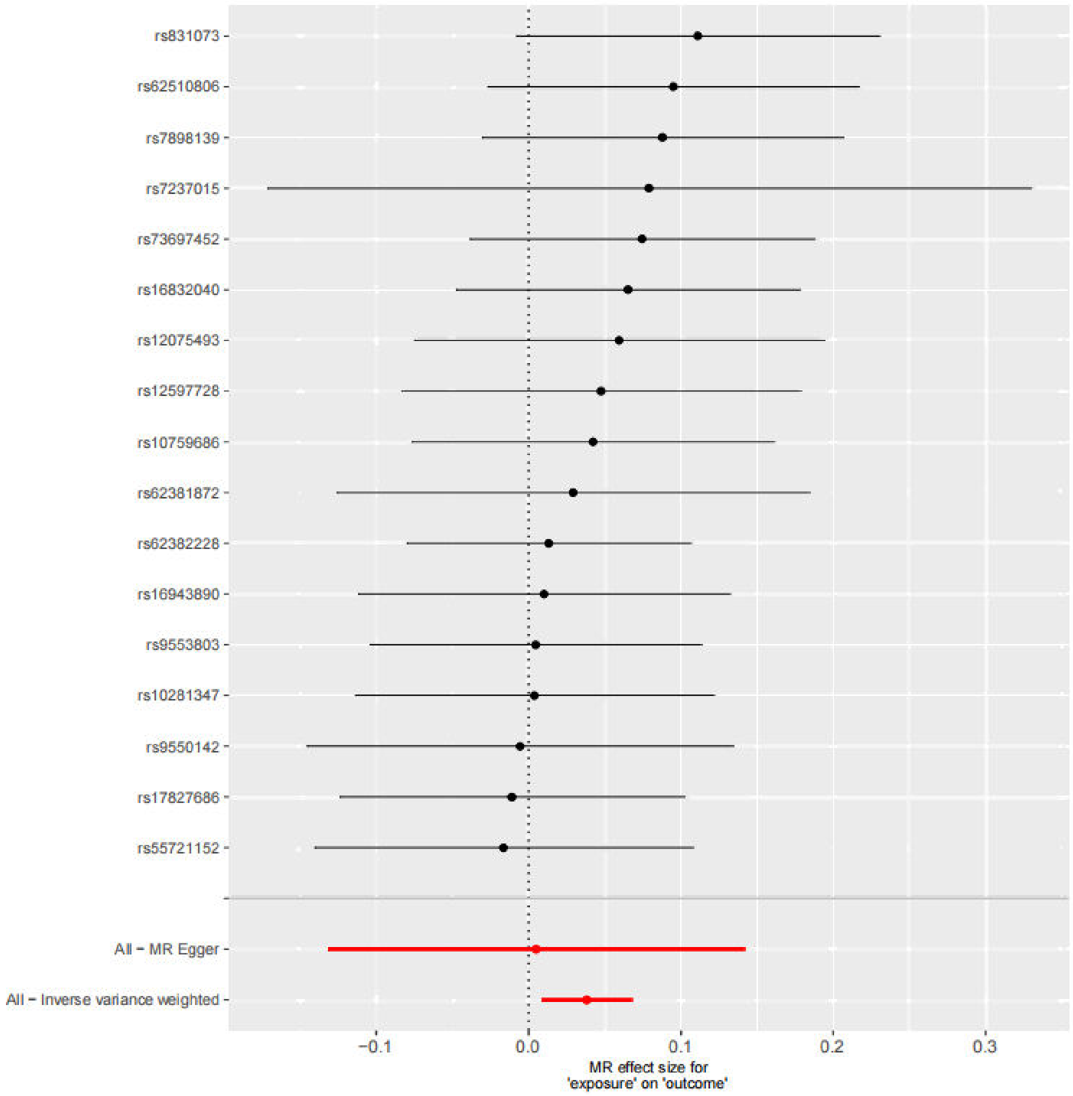
rs831073
rs62510806
rs7898139
rs7237015
rs73697452
rs16832040
rs12075493
rs12597728
rs10759686
rs62381872
rs62382228
rs16943890
rs9553803
rs10281347
rs9550142
rs17827686
rs55721152
All − MR Egger
All − Inverse variance weighted
−0.1
0.0
0.1
0.2
0.3
MR effect size for
'exposure' on 'outcome'

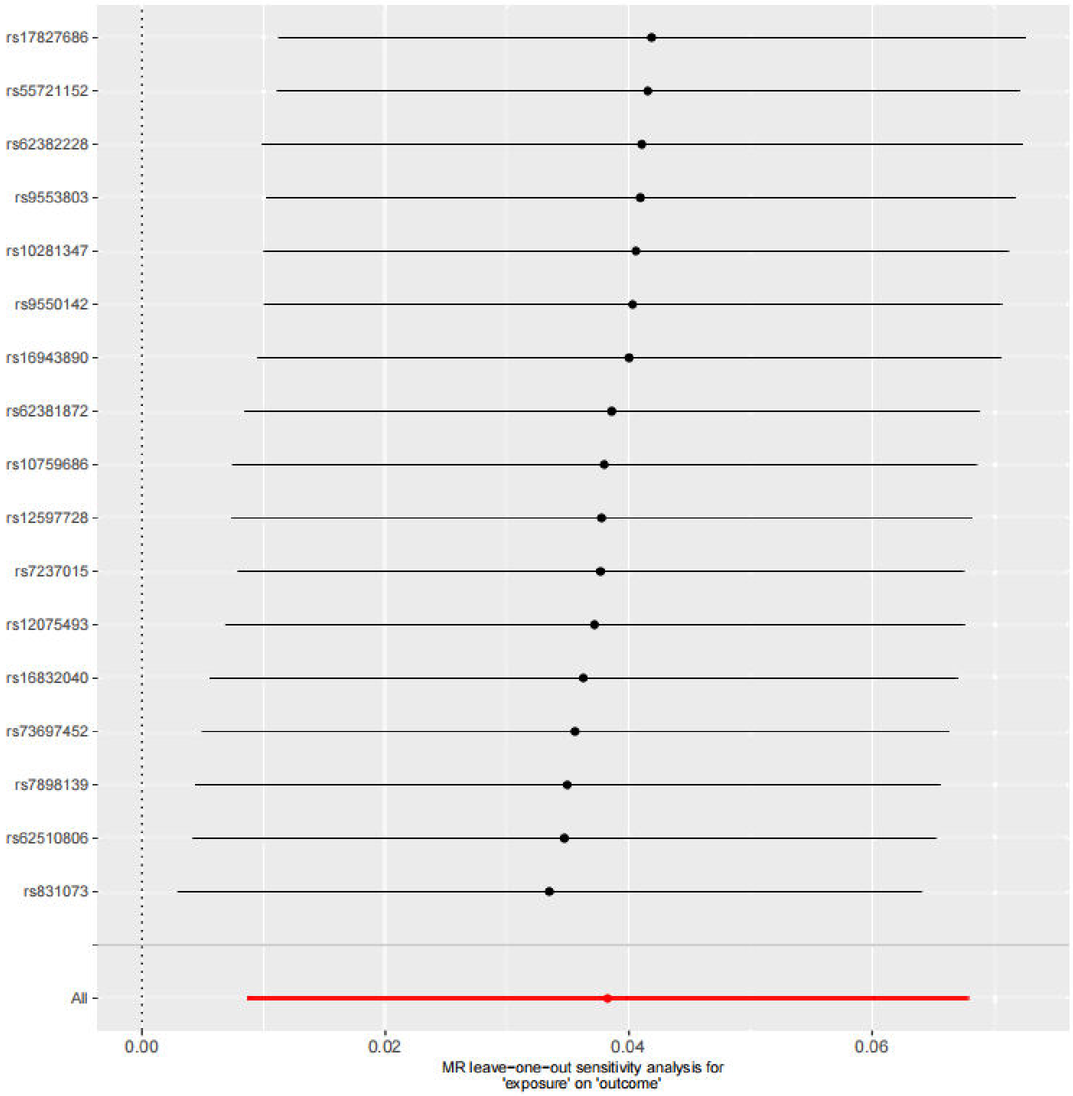
rs17827686
rs55721152
rs62382228
rs9553803
rs10281347
rs9550142
rs16943890
rs62381872
rs10759686
rs12597728
rs7237015
rs12075493
rs16832040
rs73697452
rs7898139
rs62510806
rs831073
All
0.00
0.02
0.04
0.06
MR leave−one−out sensitivity analysis for
'exposure' on 'outcome'

GCST90257030

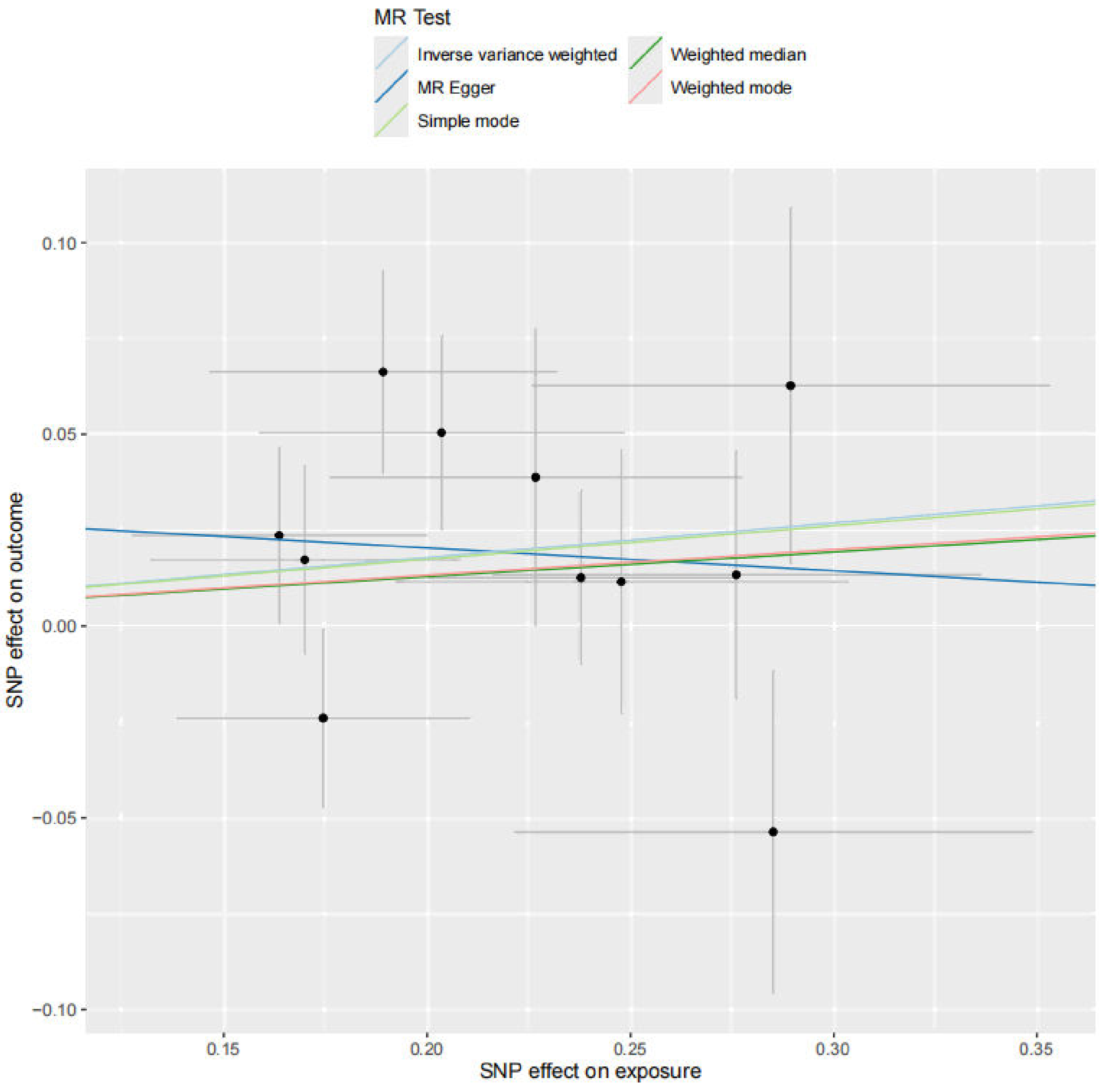

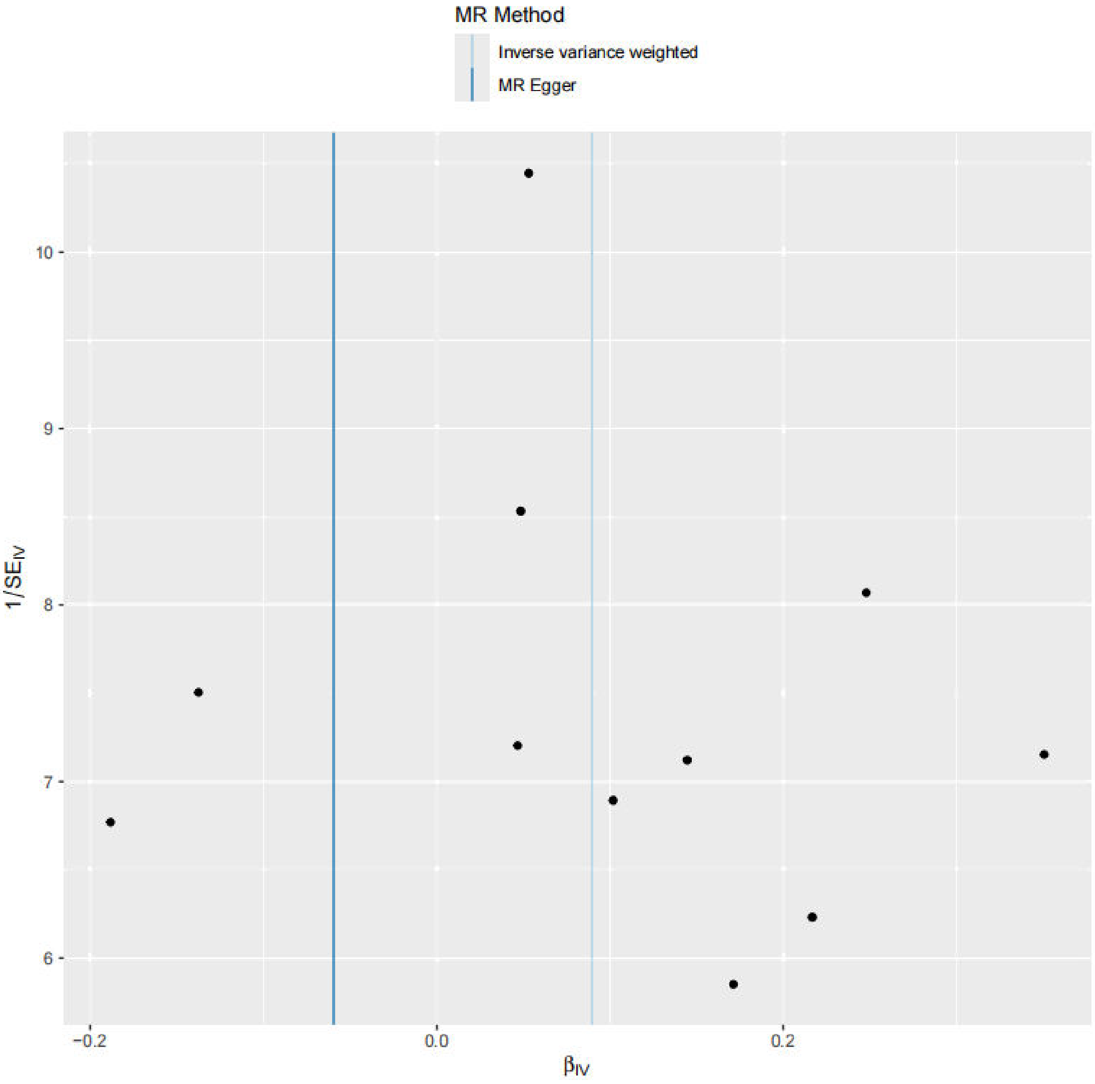

MR Method
Inverse variance weighted
MR Egger
10
9
8
7
6
1/SE_{IV}
−0.2
0.0
0.2
$β_{IV}$

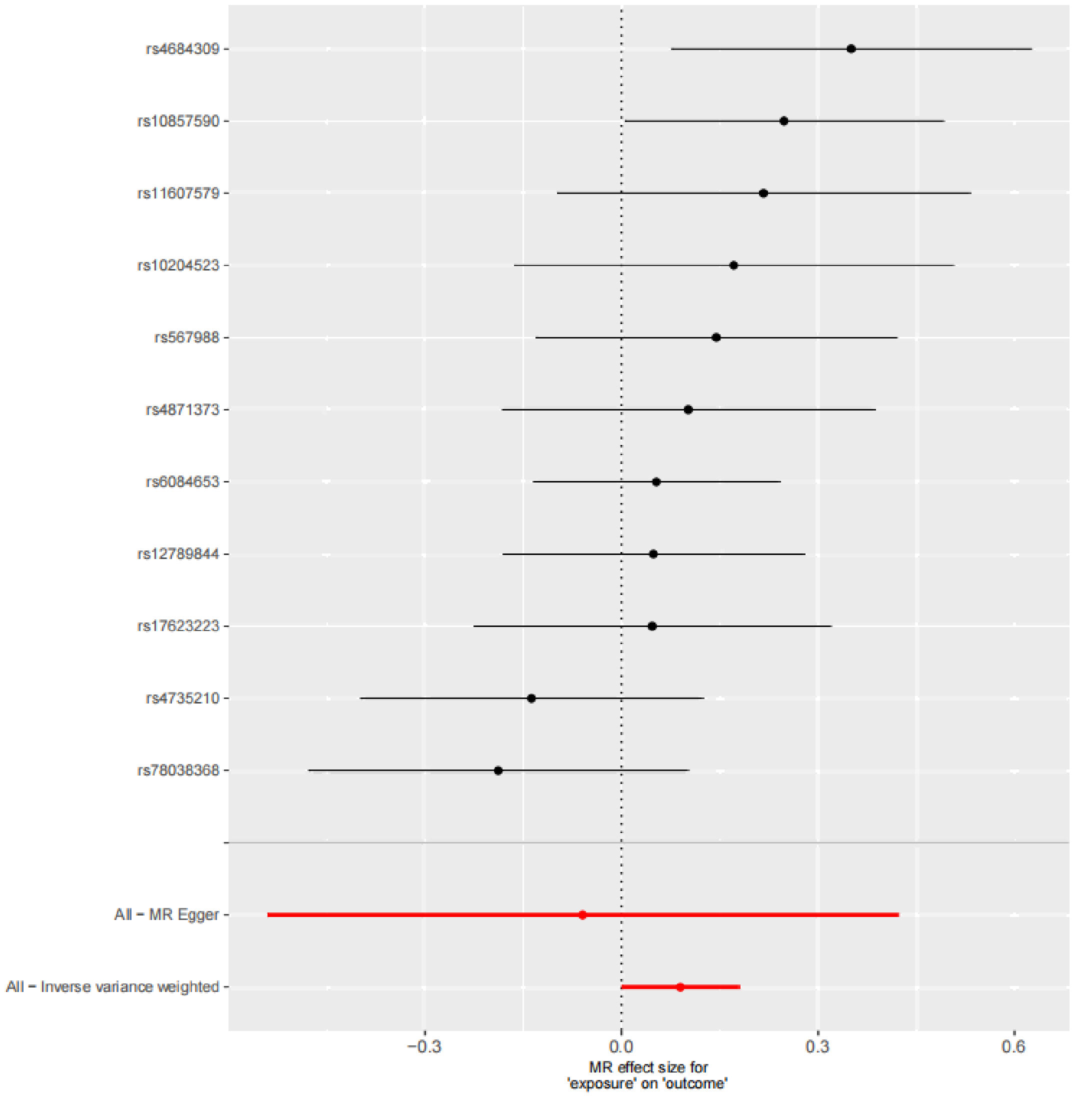

rs4684309
rs10857590
rs11607579
rs10204523
rs567988
rs4871373
rs6084653
rs12789844
rs17623223
rs4735210
rs78038368
All − MR Egger
All − Inverse variance weighted
−0.3
0.0
0.3
0.6
MR effect size for
'exposure' on 'outcome'

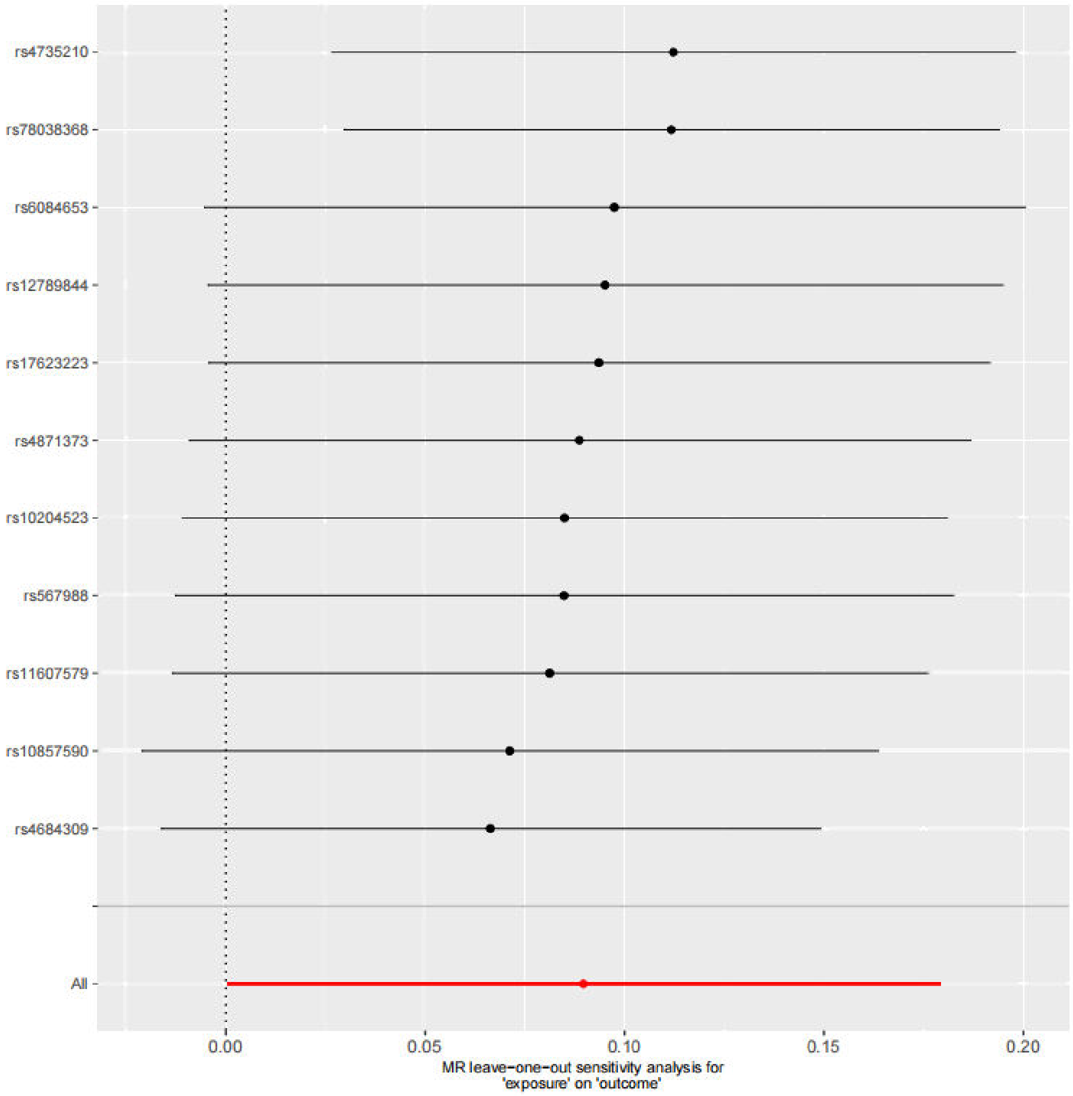
rs4735210
rs78038368
rs6084653
rs12789844
rs17623223
rs4871373
rs10204523
rs567988
rs11607579
rs10857590
rs4684309
All
0.00
0.05
0.10
0.15
0.20
MR leave−one−out sensitivity analysis for
'exposure' on 'outcome'

GCST90257040

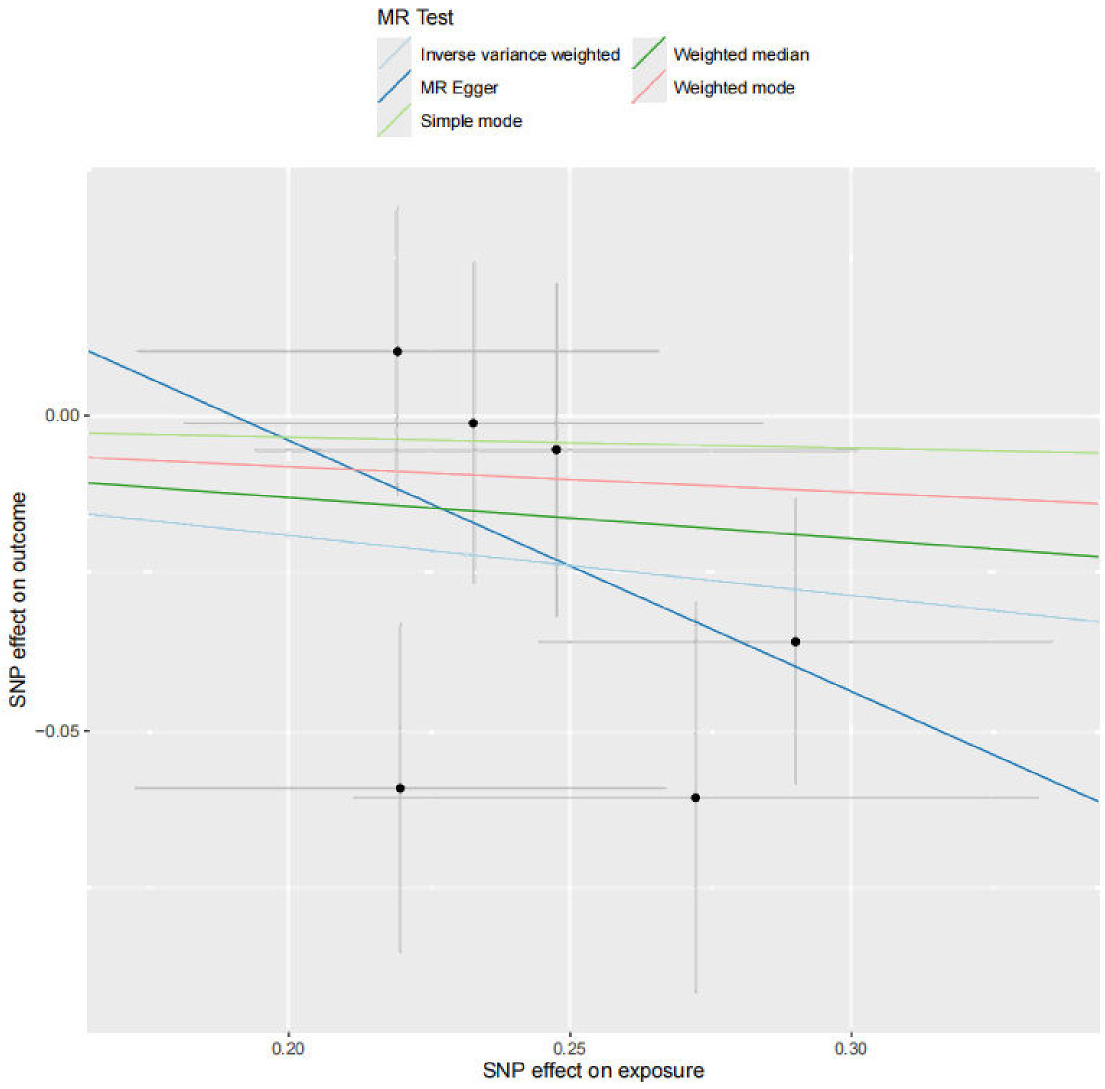

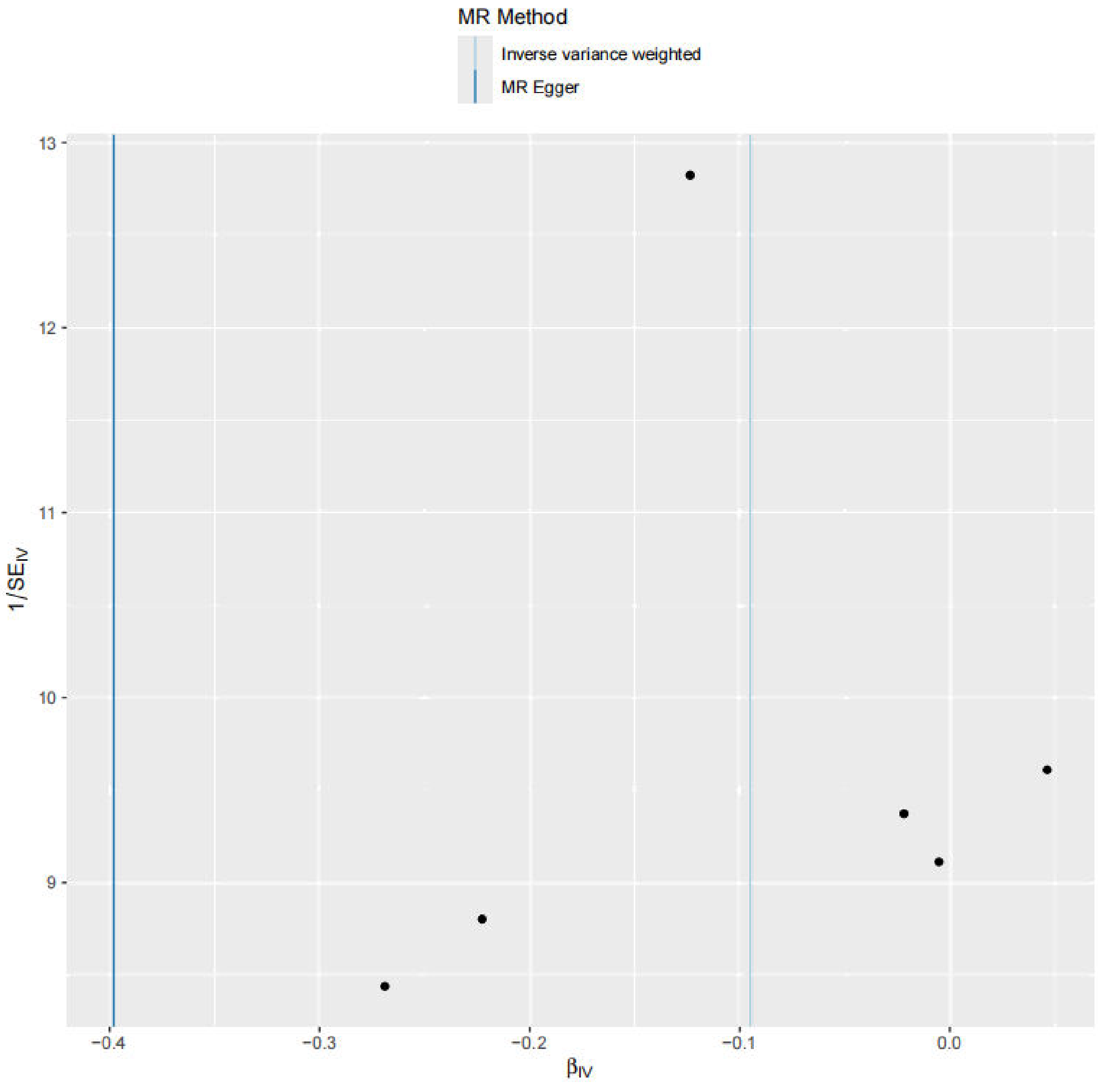

MR Method
Inverse variance weighted
MR Egger
13
12
11
10
9
1/SE$_{IV}$
−0.4
−0.3
−0.2
−0.1
0.0
β$_{IV}$

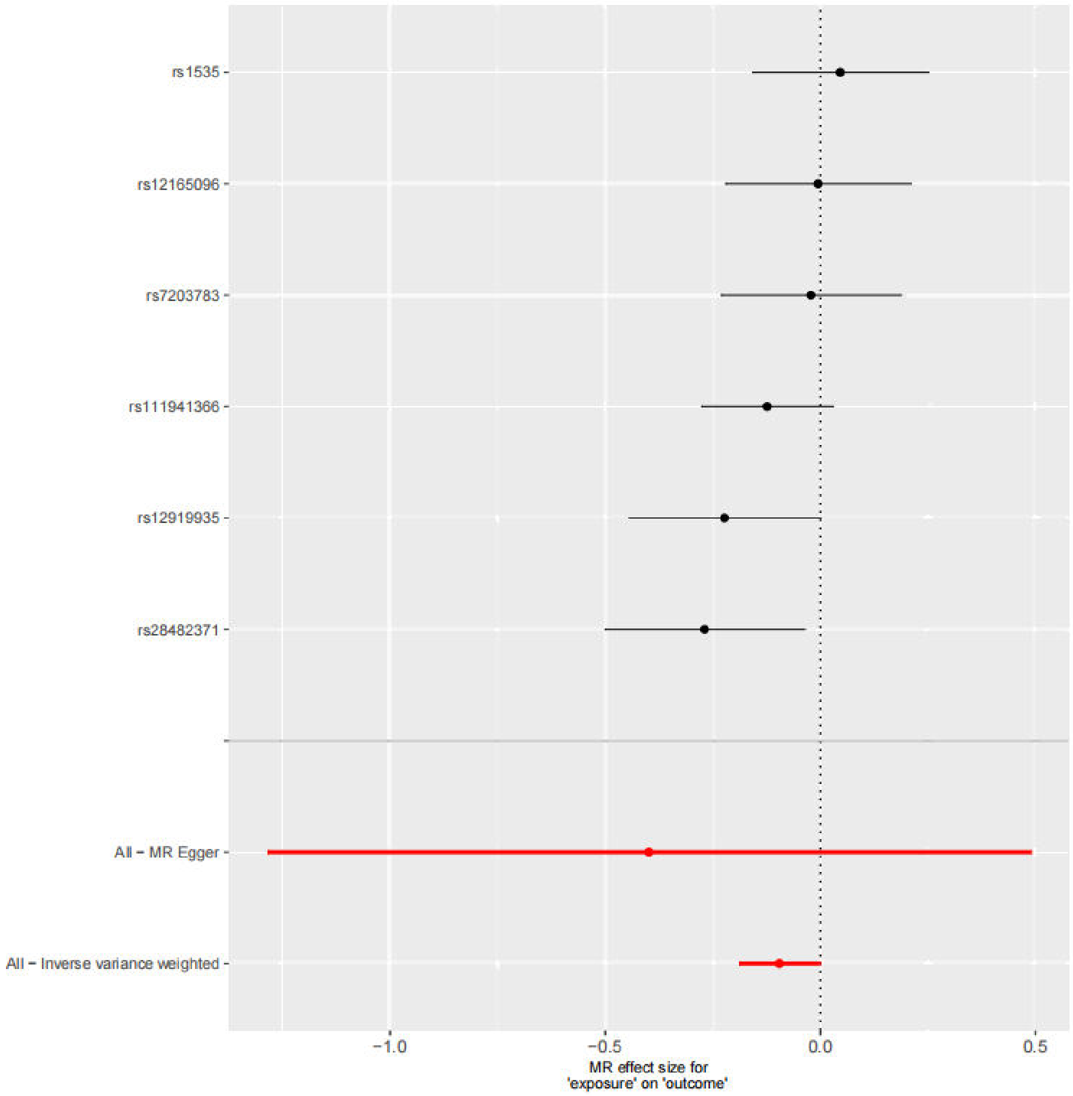

rs1535
rs12165096
rs7203783
rs111941366
rs12919935
rs28482371
All − MR Egger
All − Inverse variance weighted
−1.0
−0.5
0.0
0.5
MR effect size for
'exposure' on 'outcome'

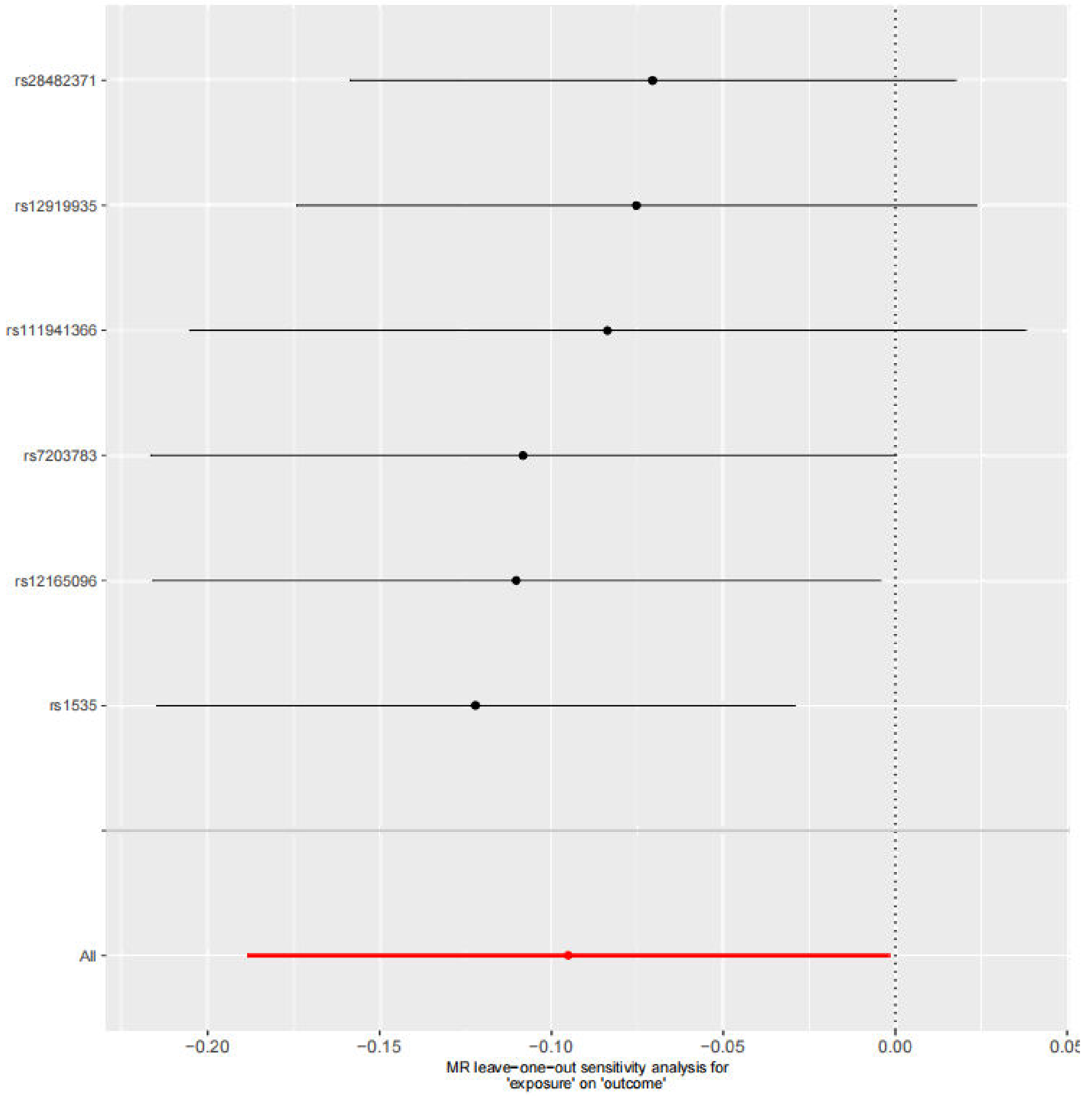
rs28482371
rs12919935
rs111941366
rs7203783
rs12165096
rs1535
All
−0.20
−0.15
−0.10
−0.05
0.00
0.05
MR leave−one−out sensitivity analysis for
'exposure' on 'outcome'

GCST90257053

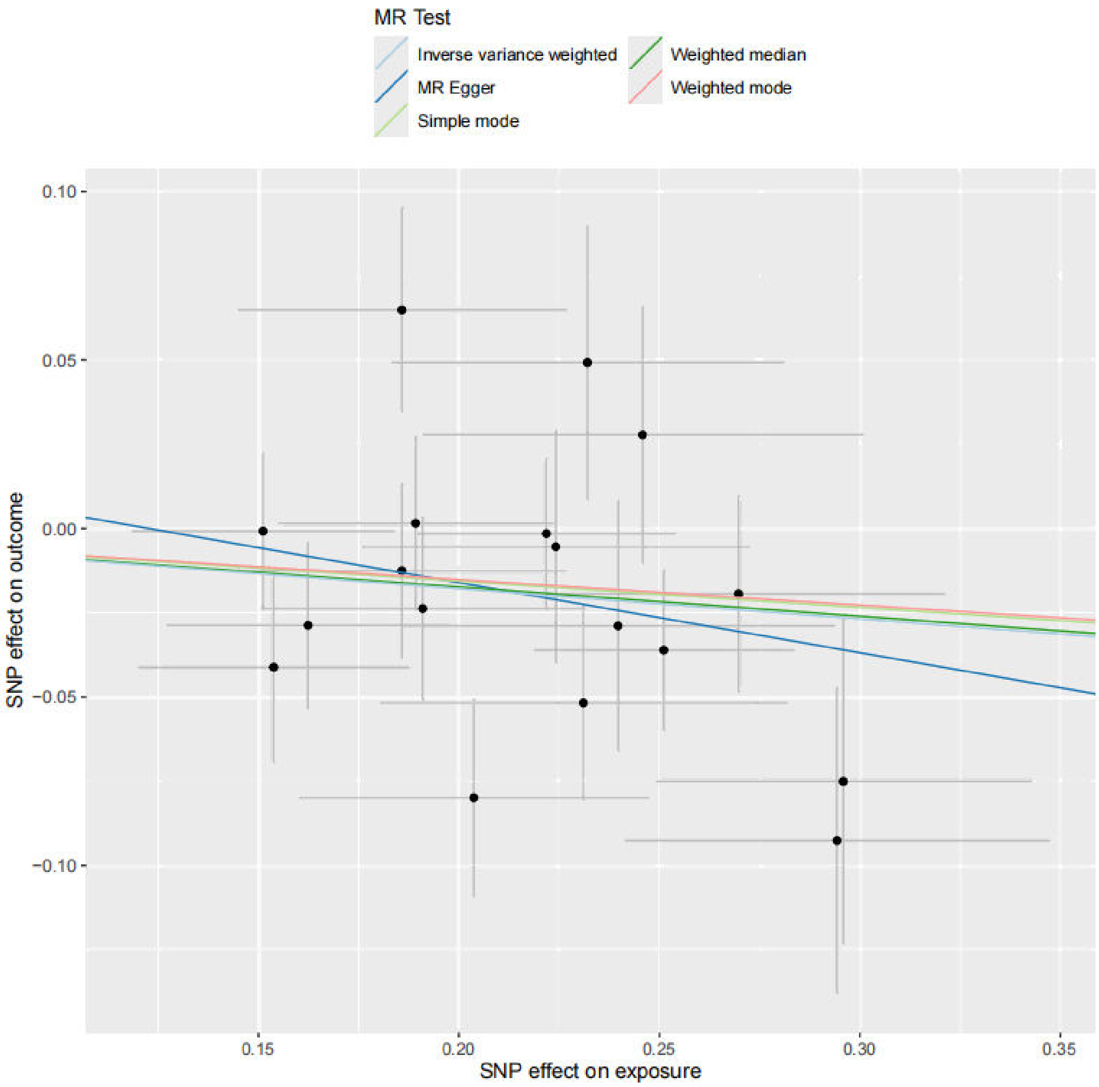

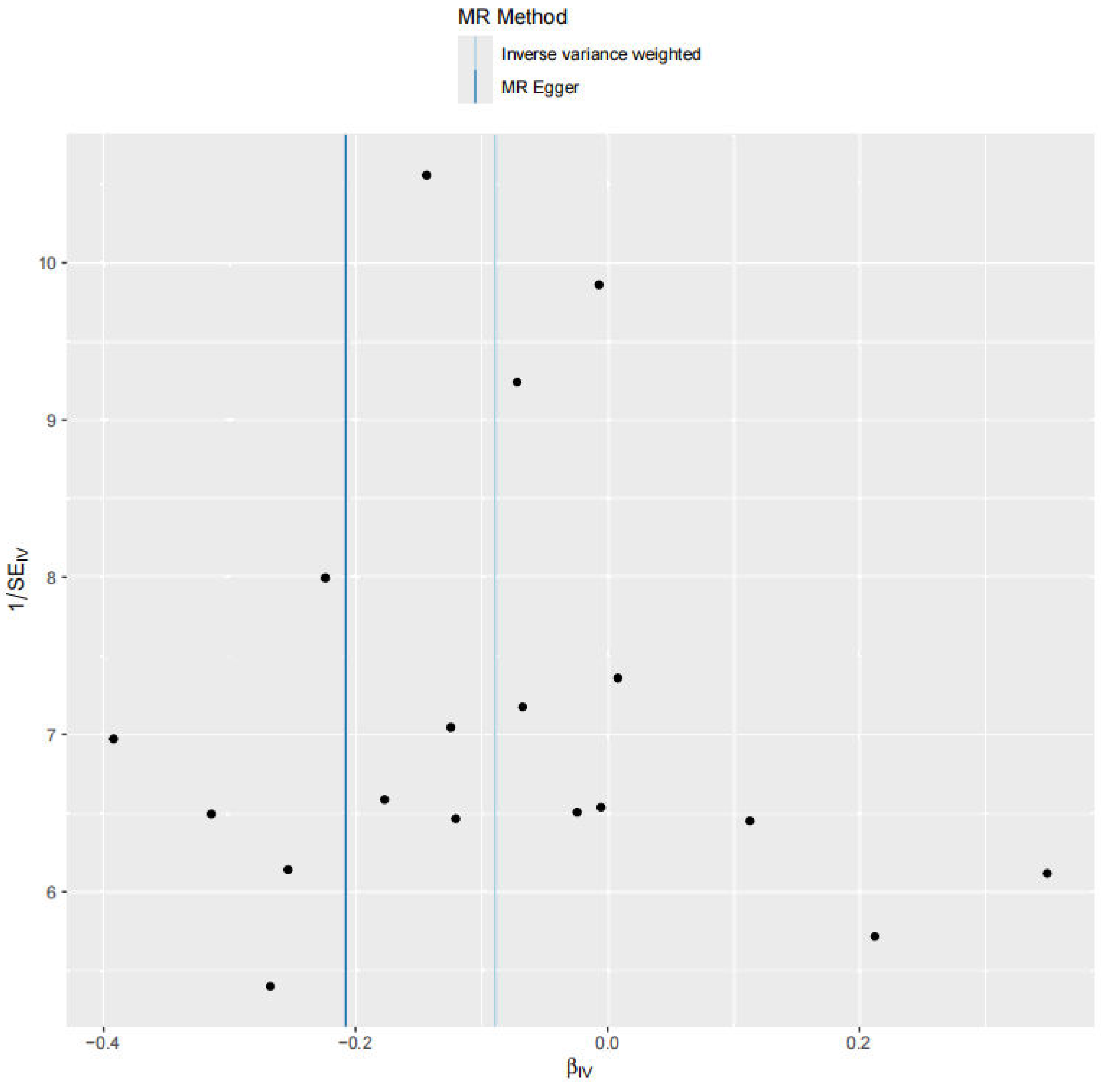

MR Method
Inverse variance weighted
MR Egger
10
9
8
7
6
1/SE$_{IV}$
−0.4
−0.2
0.0
0.2
β$_{IV}$

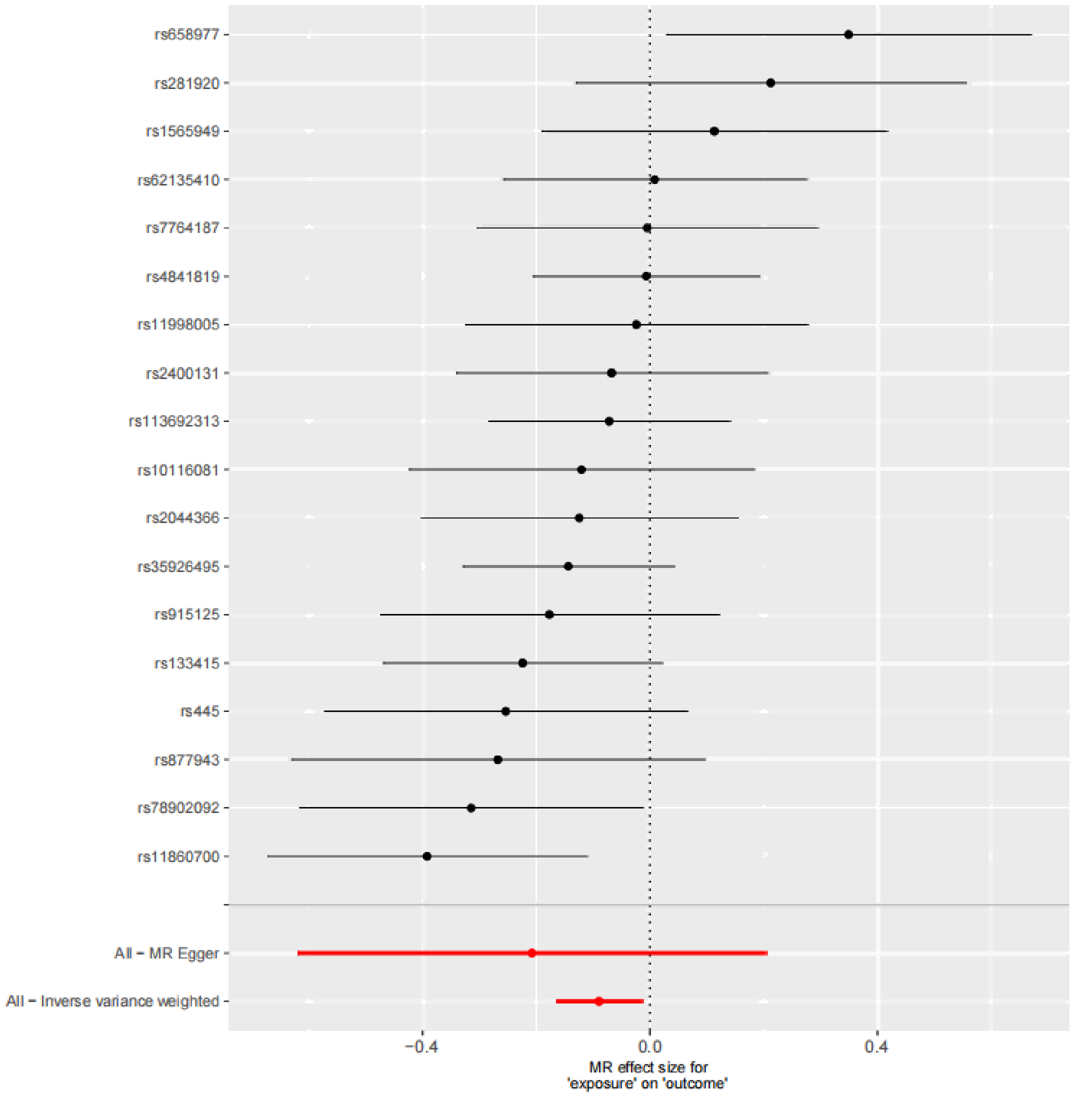
rs658977
rs281920
rs1565949
rs62135410
rs7764187
rs4841819
rs11998005
rs2400131
rs113692313
rs10116081
rs2044366
rs35926495
rs915125
rs133415
rs445
rs877943
rs78902092
rs11860700
All − MR Egger
All − Inverse variance weighted
−0.4
0.0
0.4
MR effect size for
'exposure' on 'outcome'

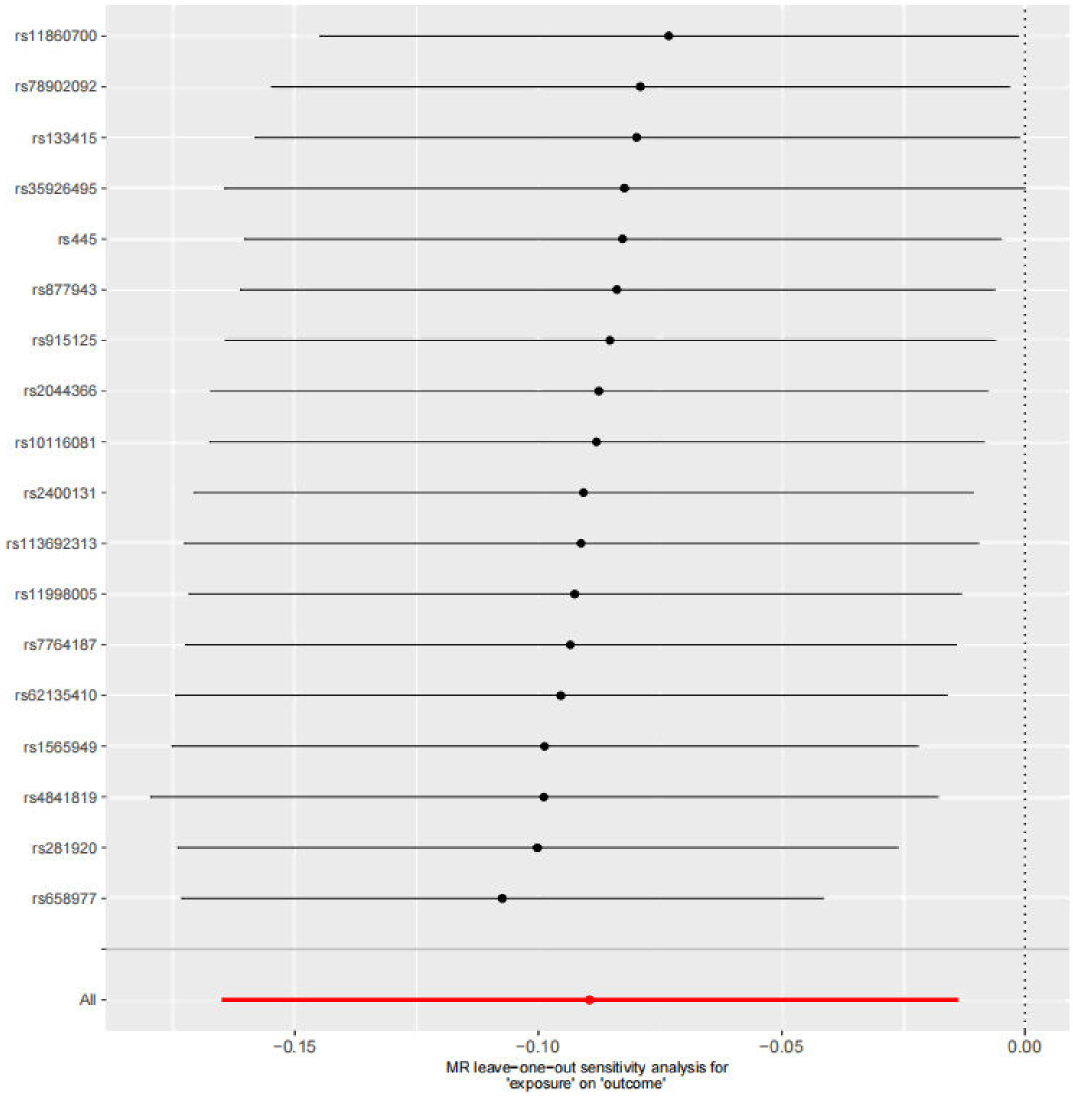

rs11860700
rs78902092
rs133415
rs35926495
rs445
rs877943
rs915125
rs2044366
rs10116081
rs2400131
rs113692313
rs11998005
rs7764187
rs62135410
rs1565949
rs4841819
rs281920
rs658977
All
−0.15
−0.10
−0.05
0.00
MR leave−one−out sensitivity analysis for
'exposure' on 'outcome'

GCST90257073

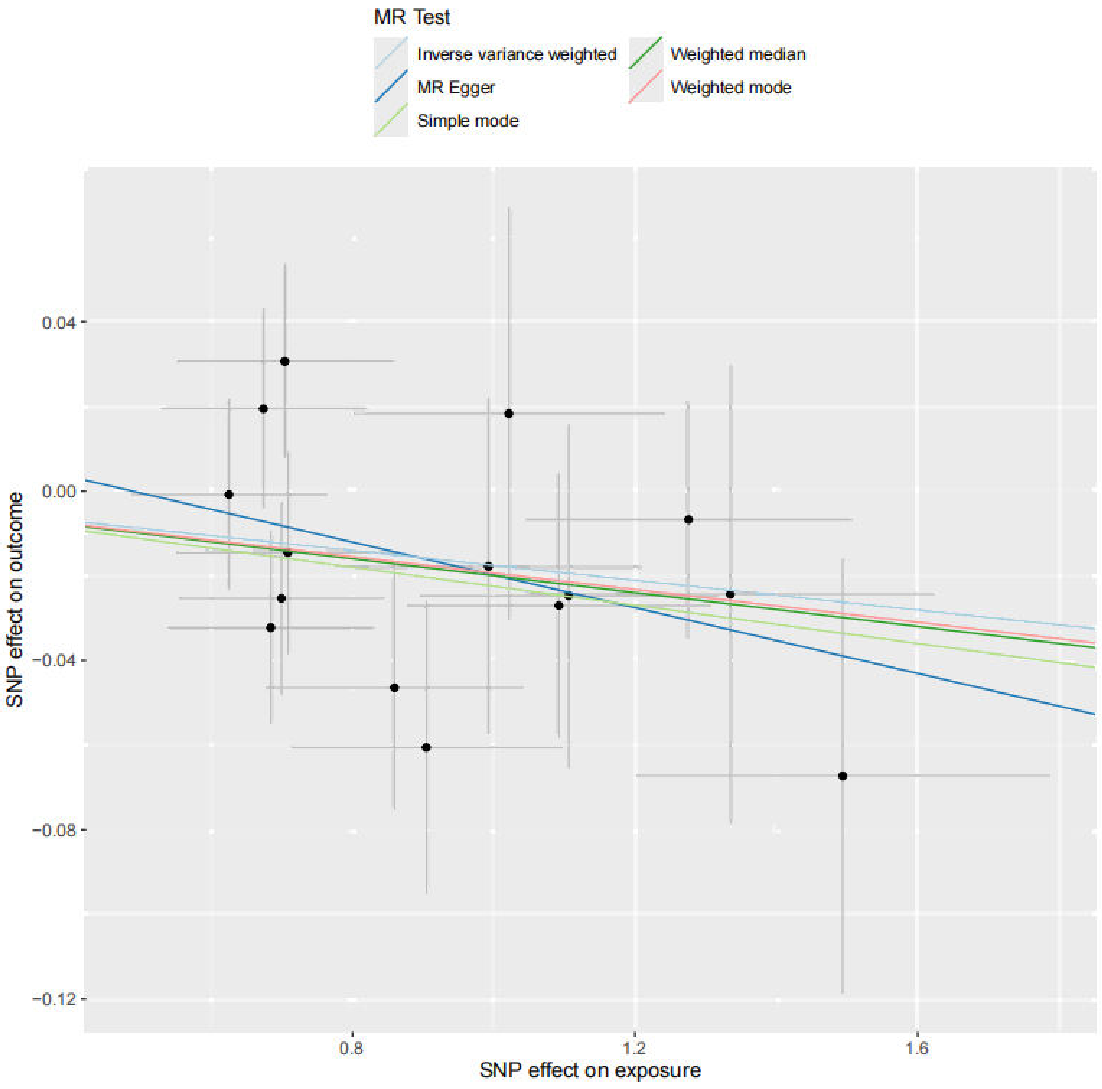

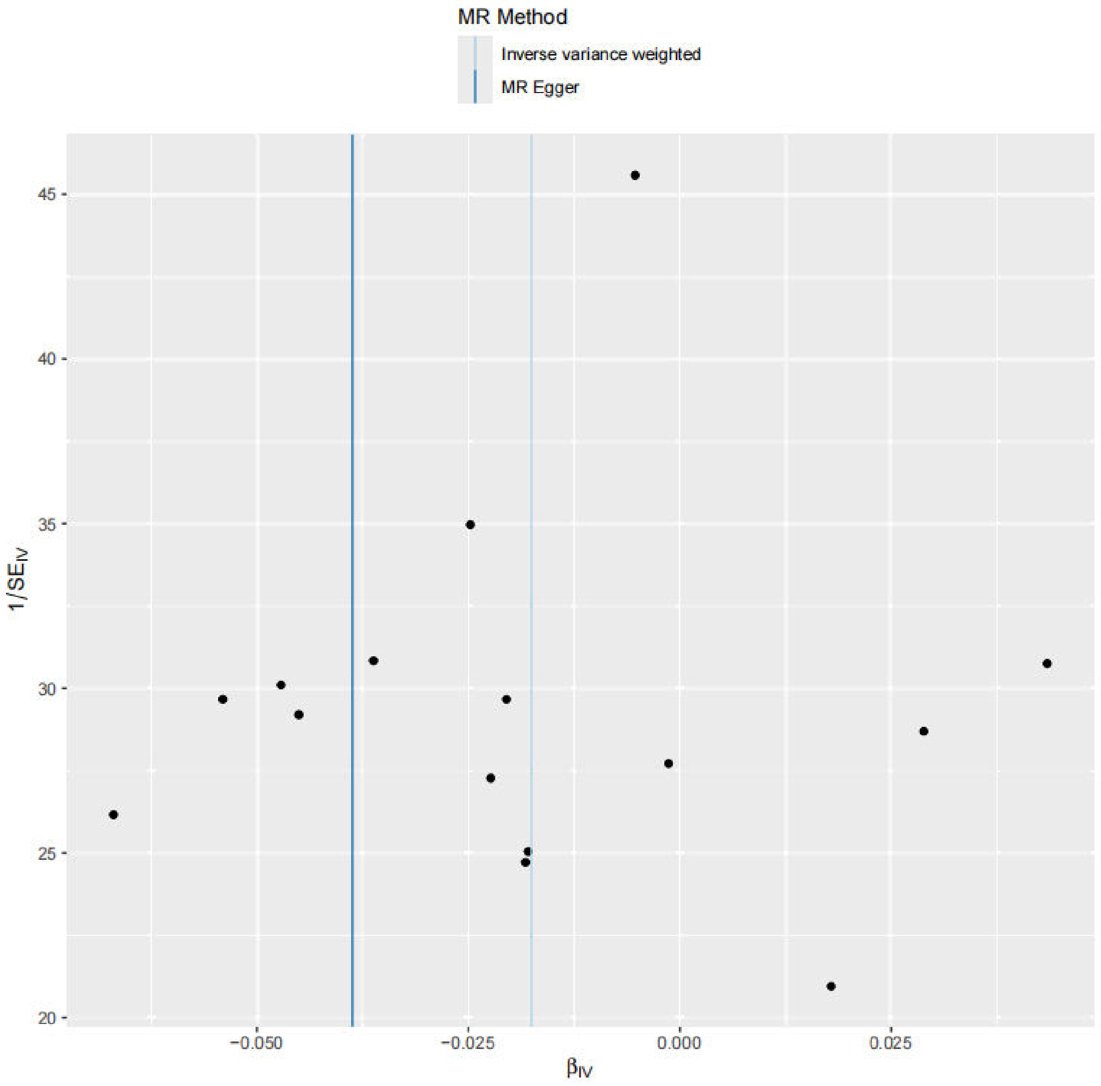

MR Method
Inverse variance weighted
MR Egger
45
40
35
30
25
20
1/SE_IV
−0.050
−0.025
0.000
0.025
β_IV

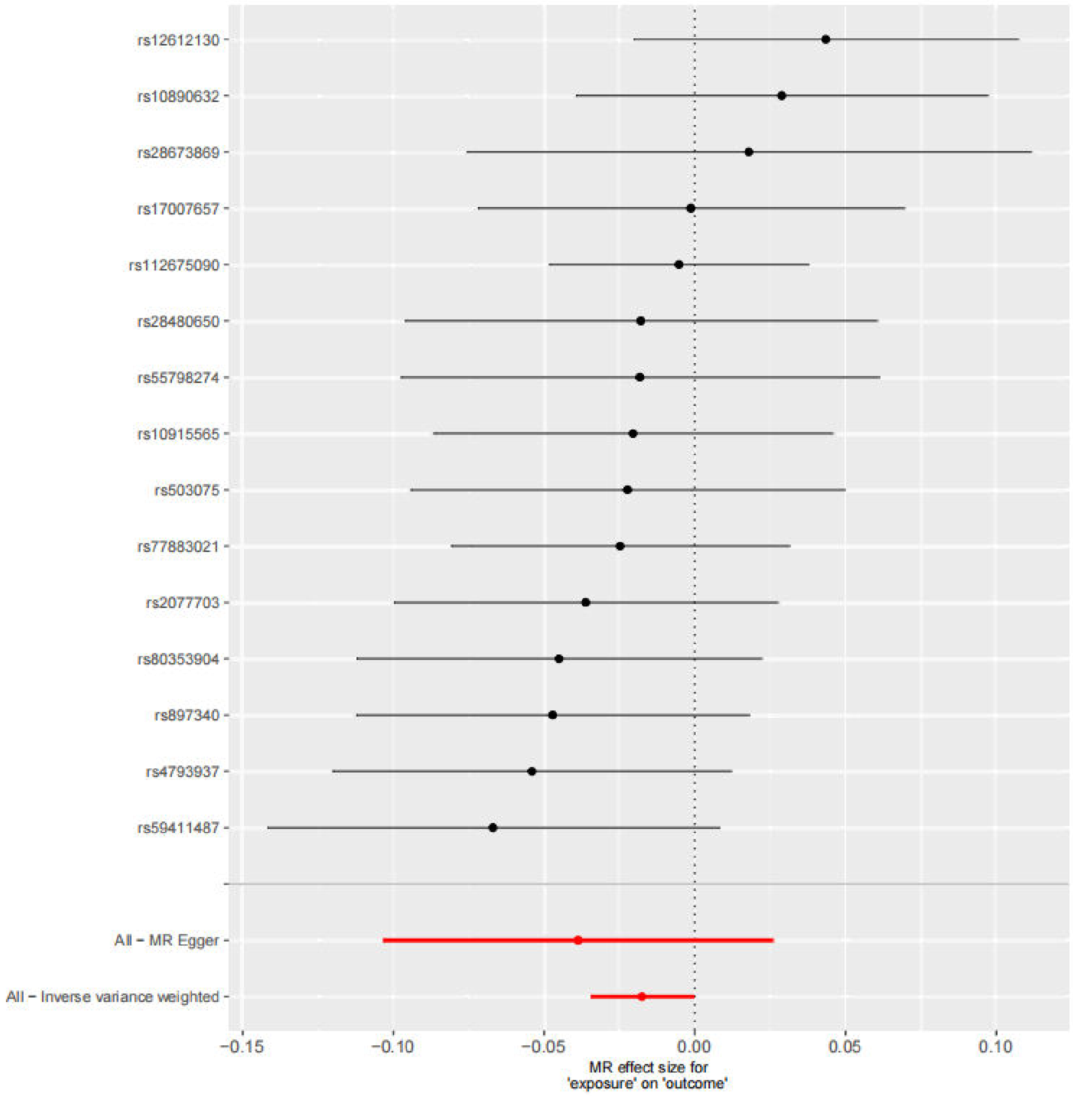

rs12612130
rs10890632
rs28673869
rs17007657
rs112675090
rs28480650
rs55798274
rs10915565
rs503075
rs77883021
rs2077703
rs80353904
rs897340
rs4793937
rs59411487
All − MR Egger
All − Inverse variance weighted
−0.15
−0.10
−0.05
0.00
0.05
0.10
MR effect size for
'exposure' on 'outcome'

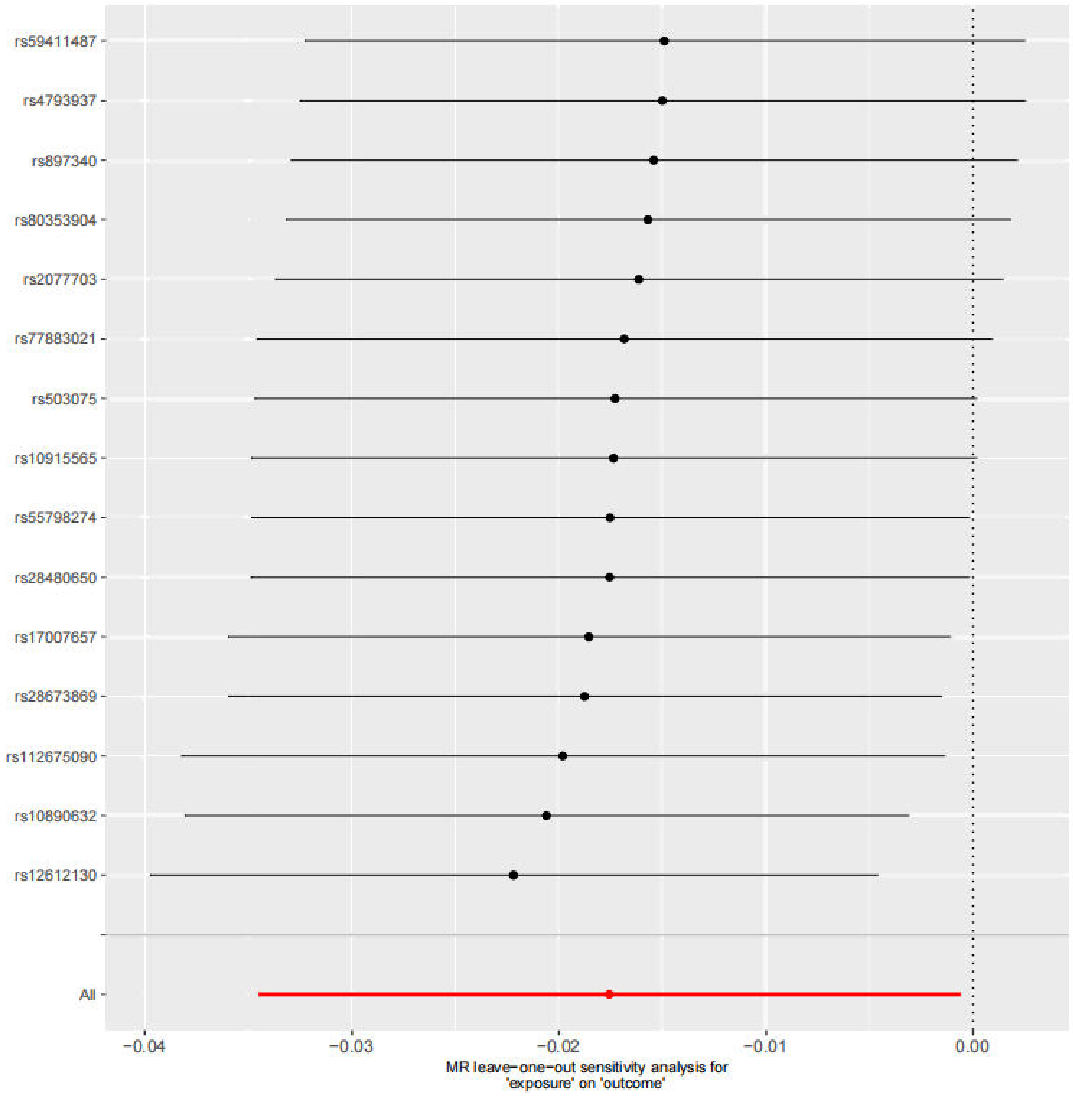

rs59411487
rs4793937
rs897340
rs80353904
rs2077703
rs77883021
rs503075
rs10915565
rs55798274
rs28480650
rs17007657
rs28673869
rs112675090
rs10890632
rs12612130
All
−0.04
−0.03
−0.02
−0.01
0.00
MR leave−one−out sensitivity analysis for
'exposure' on 'outcome'

GCST90257076

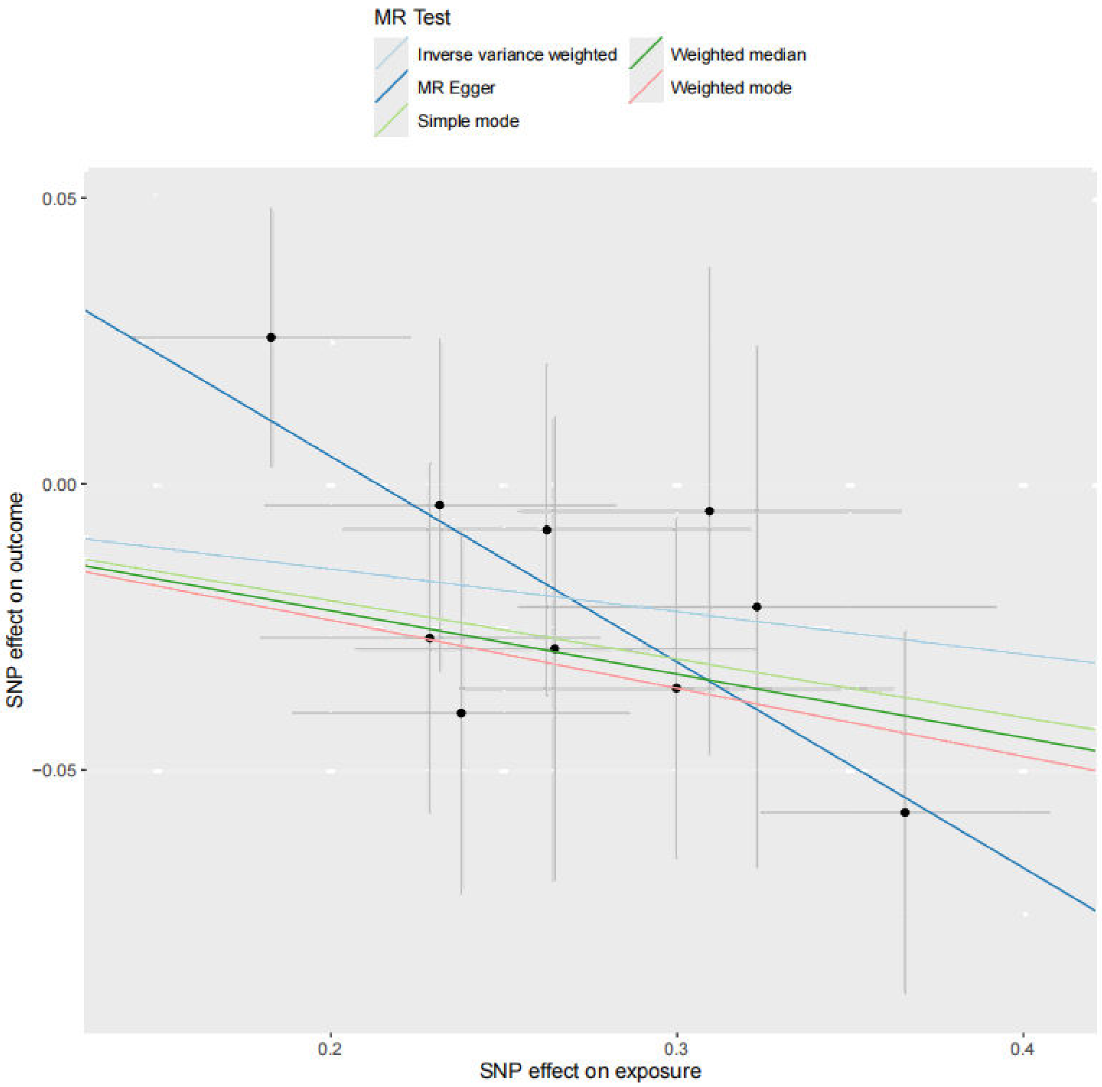

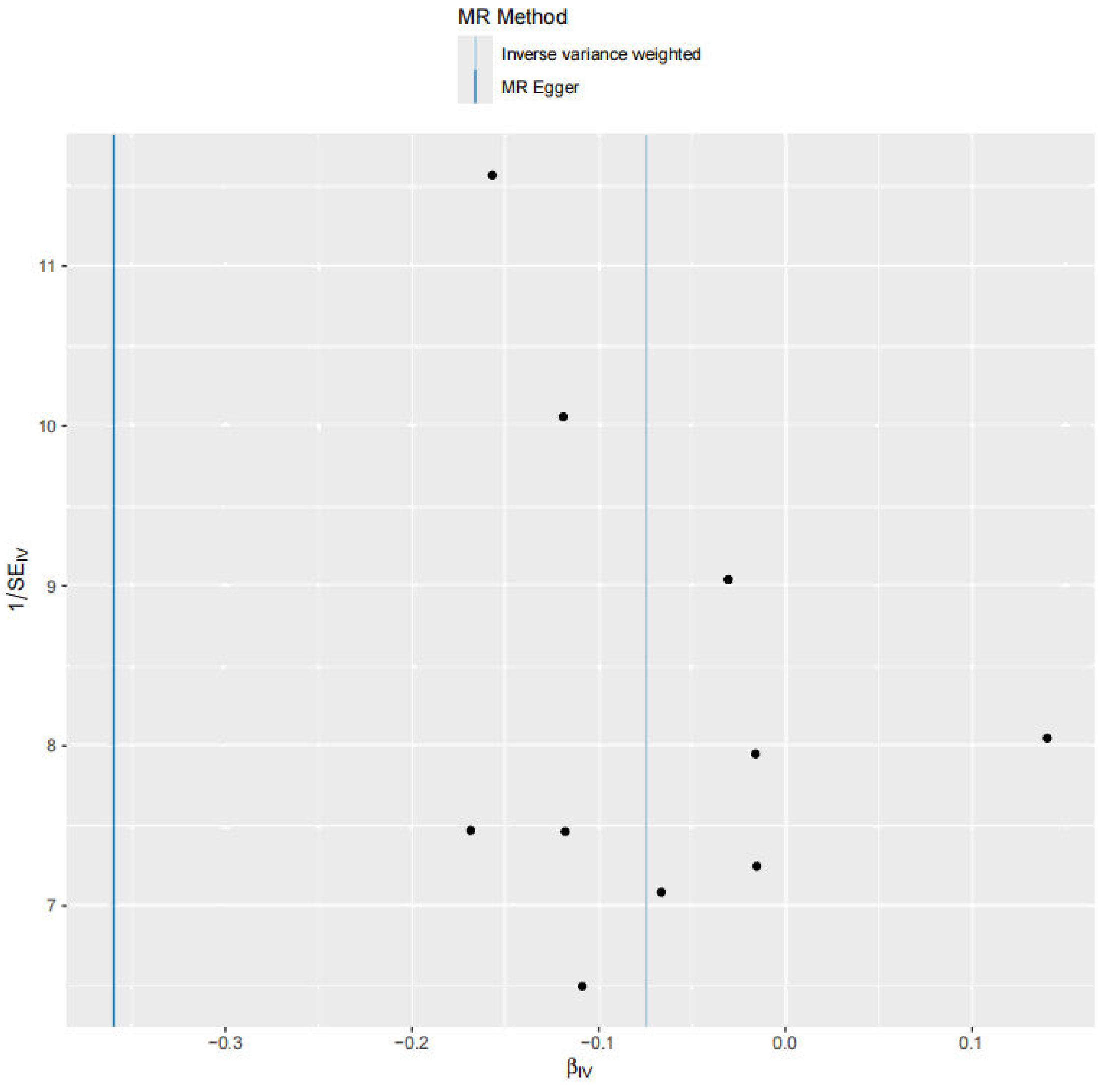

MR Method
Inverse variance weighted
MR Egger
11
10
9
8
7
1/SE_IV
−0.3
−0.2
−0.1
0.0
0.1
β_IV

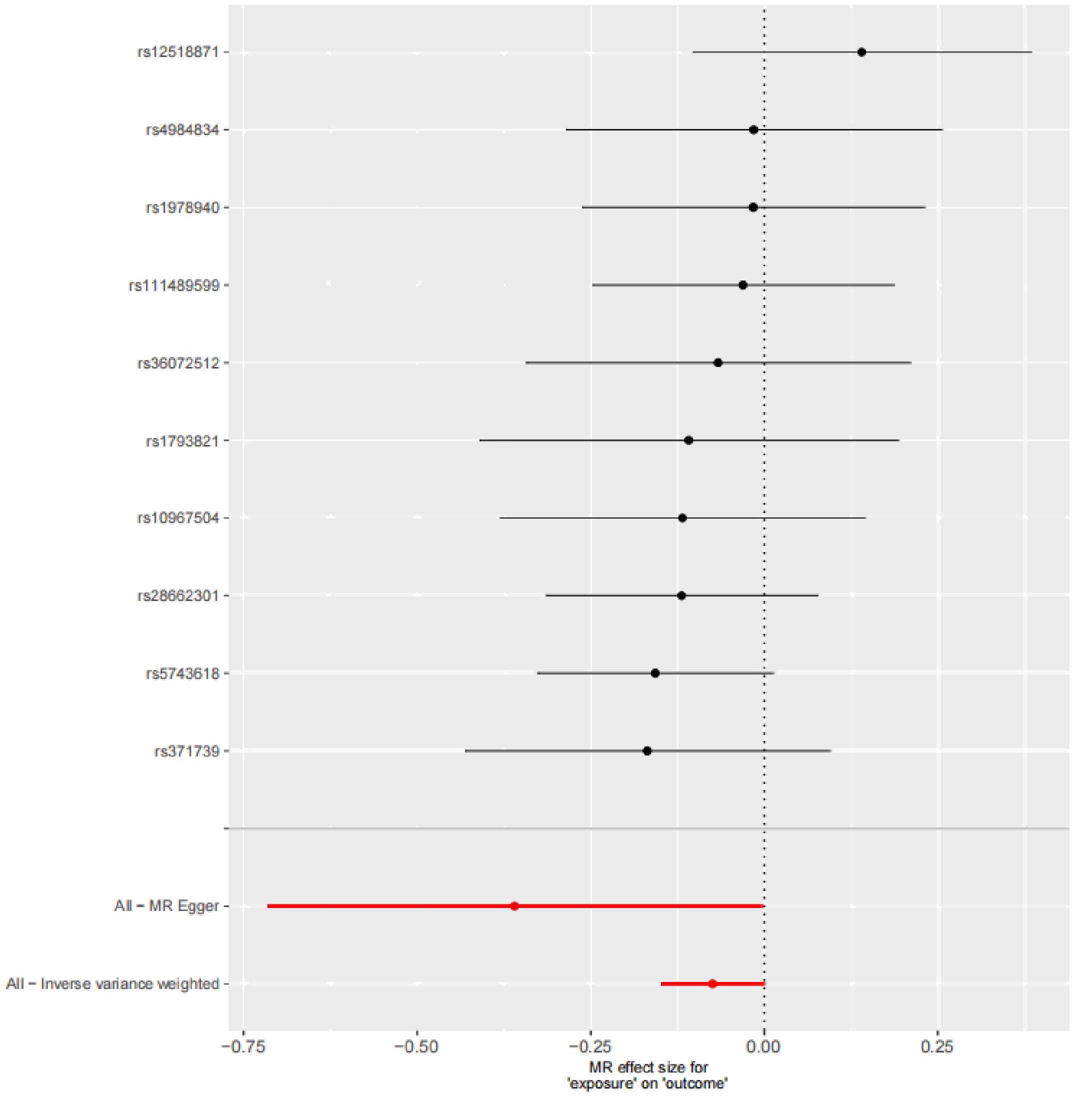
rs12518871
rs4984834
rs1978940
rs111489599
rs36072512
rs1793821
rs10967504
rs28662301
rs5743618
rs371739
All − MR Egger
All − Inverse variance weighted
−0.75
−0.50
−0.25
0.00
0.25
MR effect size for
'exposure' on 'outcome'

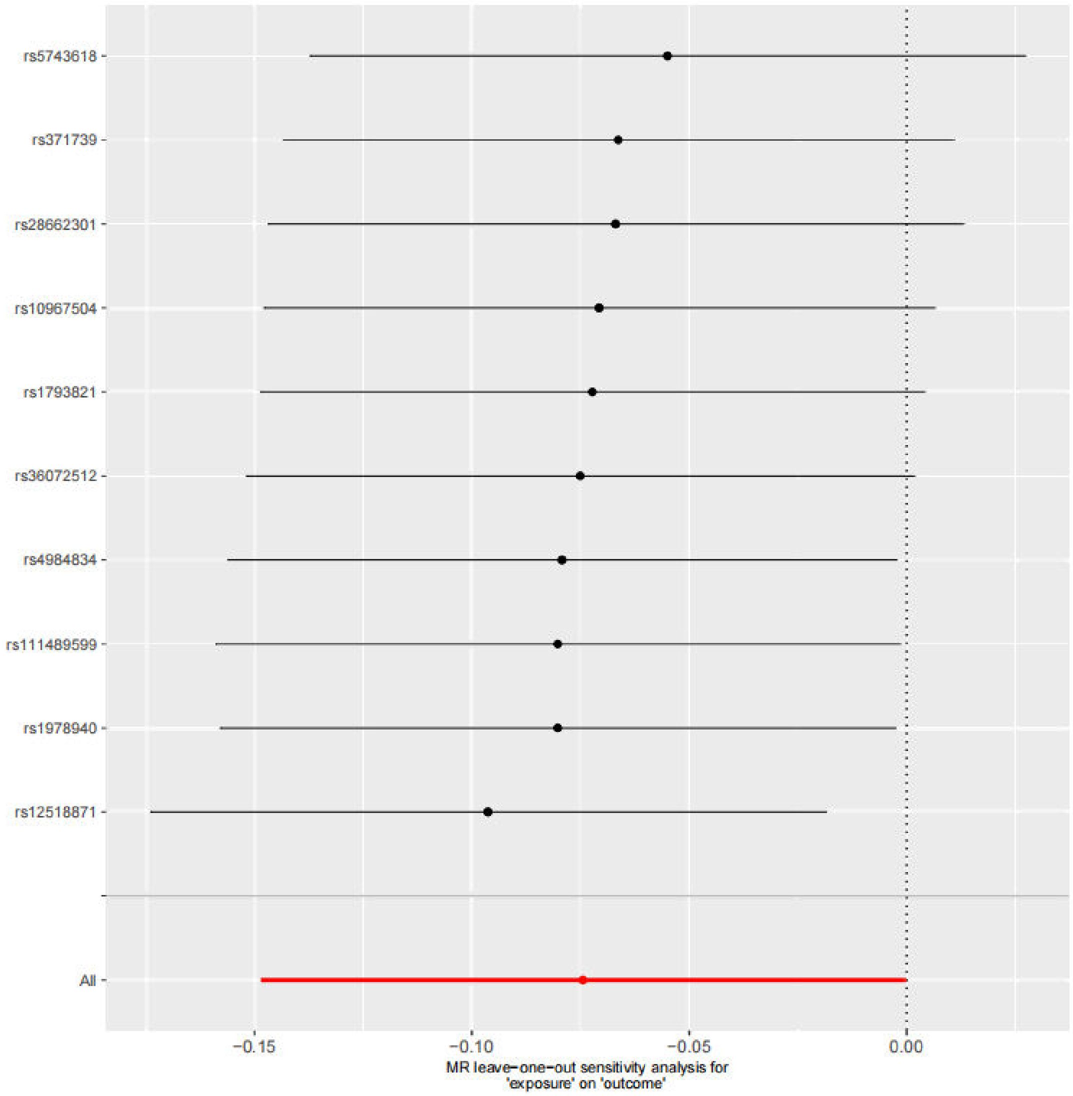

rs5743618
rs371739
rs28662301
rs10967504
rs1793821
rs36072512
rs4984834
rs111489599
rs1978940
rs12518871
All
−0.15
−0.10
−0.05
0.00
MR leave−one−out sensitivity analysis for
'exposure' on 'outcome'

GCST90257077

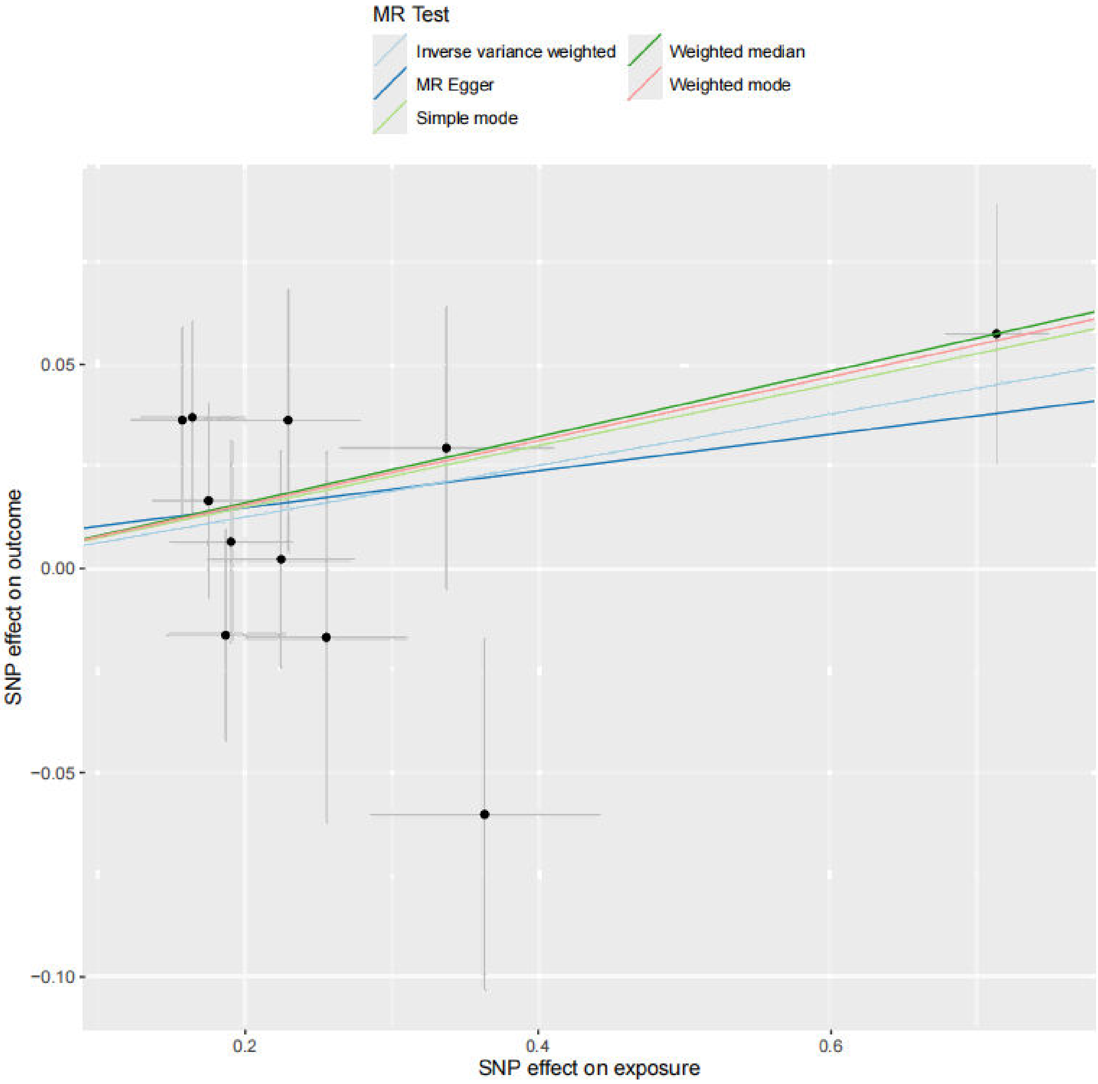

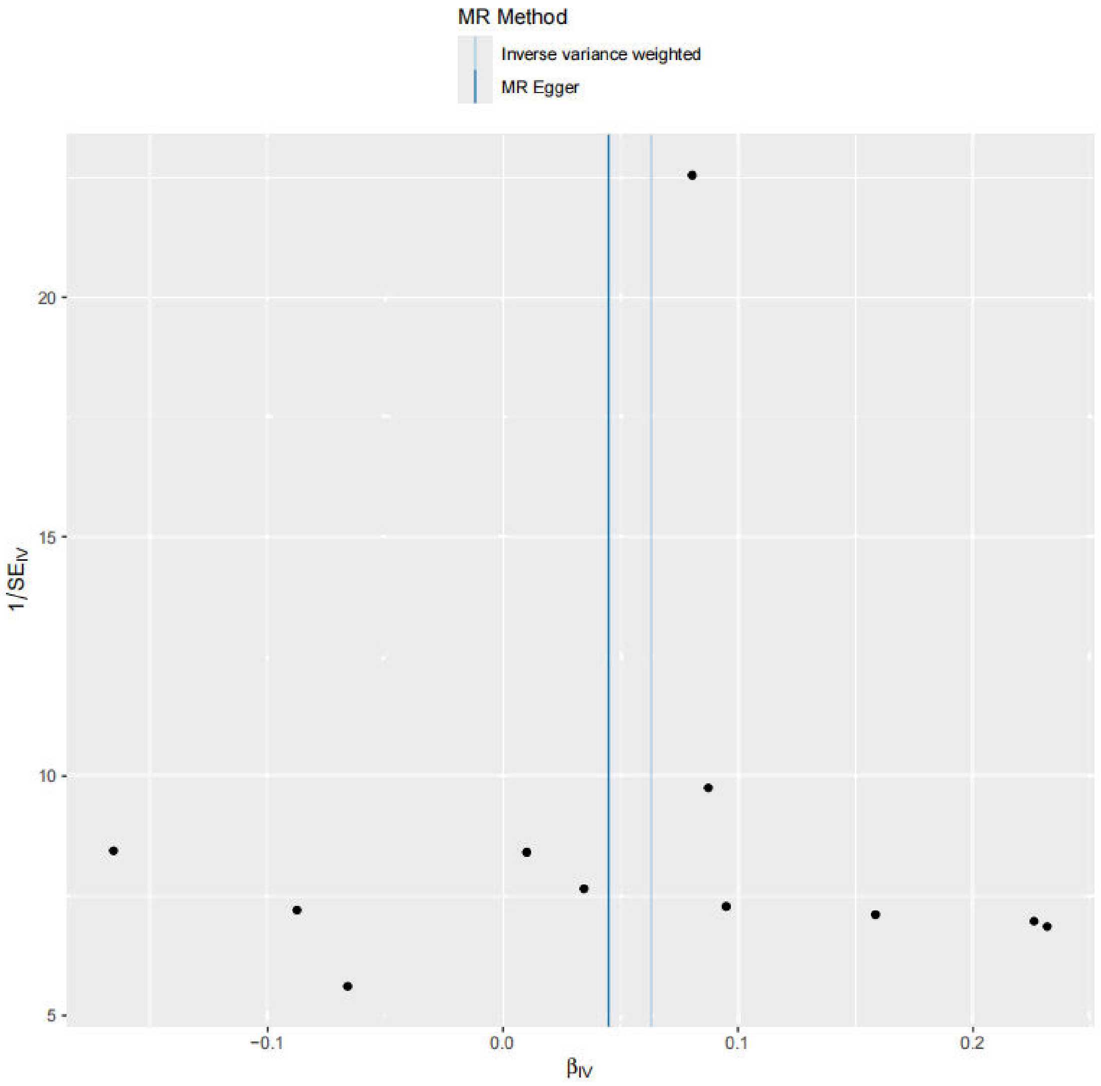
MR Method
Inverse variance weighted
MR Egger
1/SE$_{IV}$
20
15
10
5
−0.1
0.0
0.1
0.2
β$_{IV}$

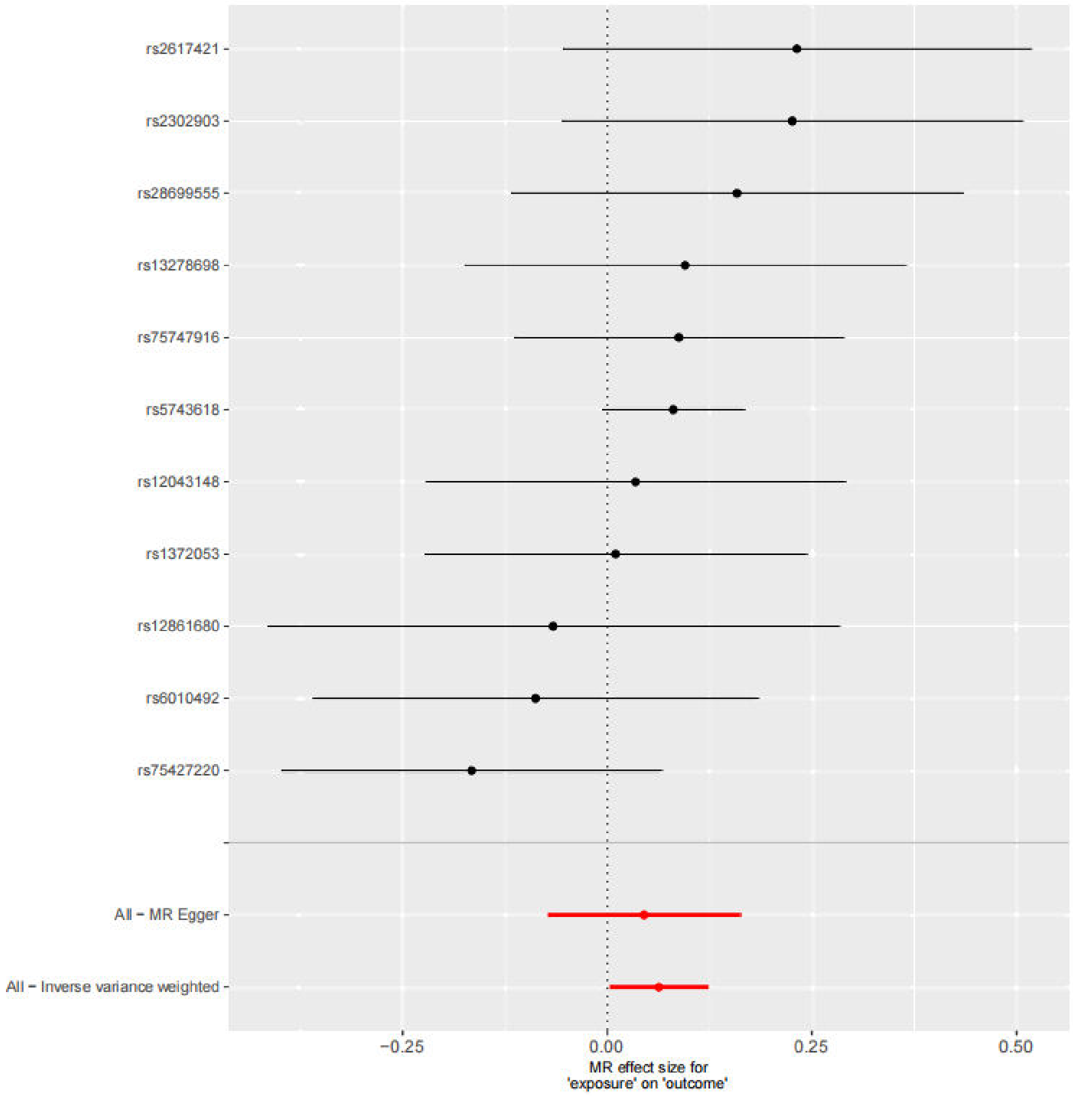

rs2617421
rs2302903
rs28699555
rs13278698
rs75747916
rs5743618
rs12043148
rs1372053
rs12861680
rs6010492
rs75427220
All − MR Egger
All − Inverse variance weighted
−0.25
0.00
0.25
0.50
MR effect size for
'exposure' on 'outcome'

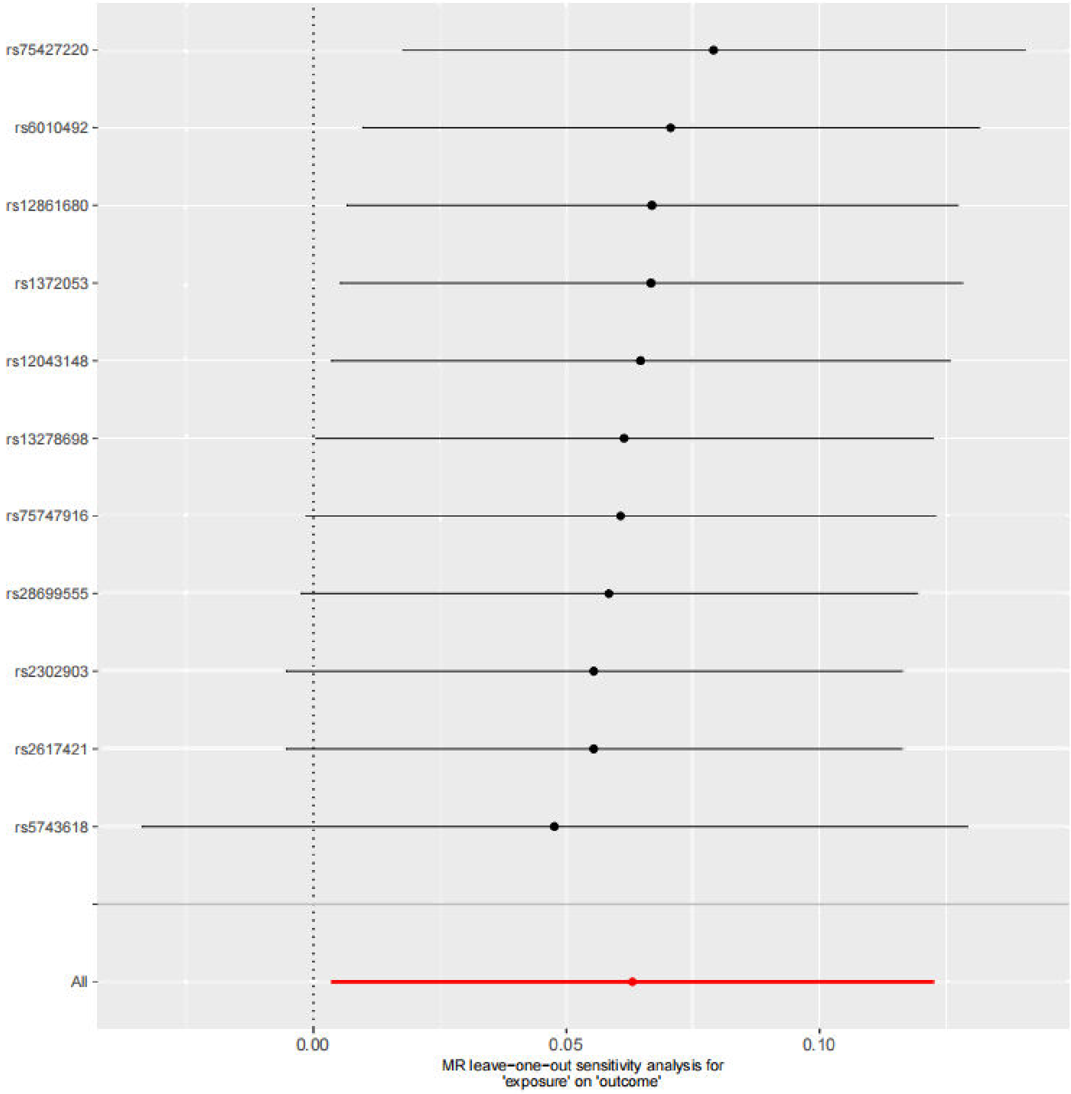
rs75427220
rs6010492
rs12861680
rs1372053
rs12043148
rs13278698
rs75747916
rs28699555
rs2302903
rs2617421
rs5743618
All
0.00
0.05
0.10
MR leave−one−out sensitivity analysis for
'exposure' on 'outcome'

GCST90257086

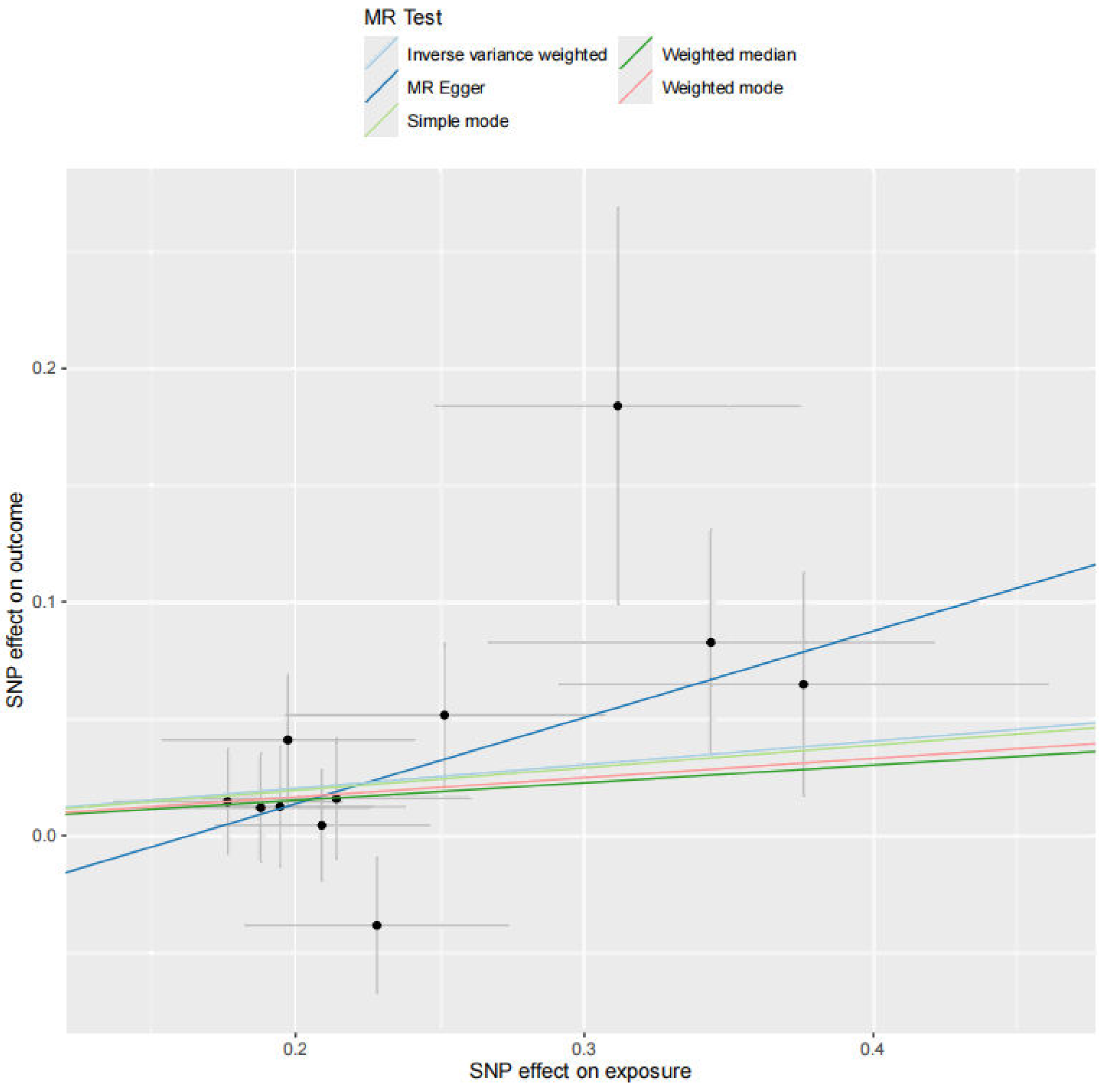

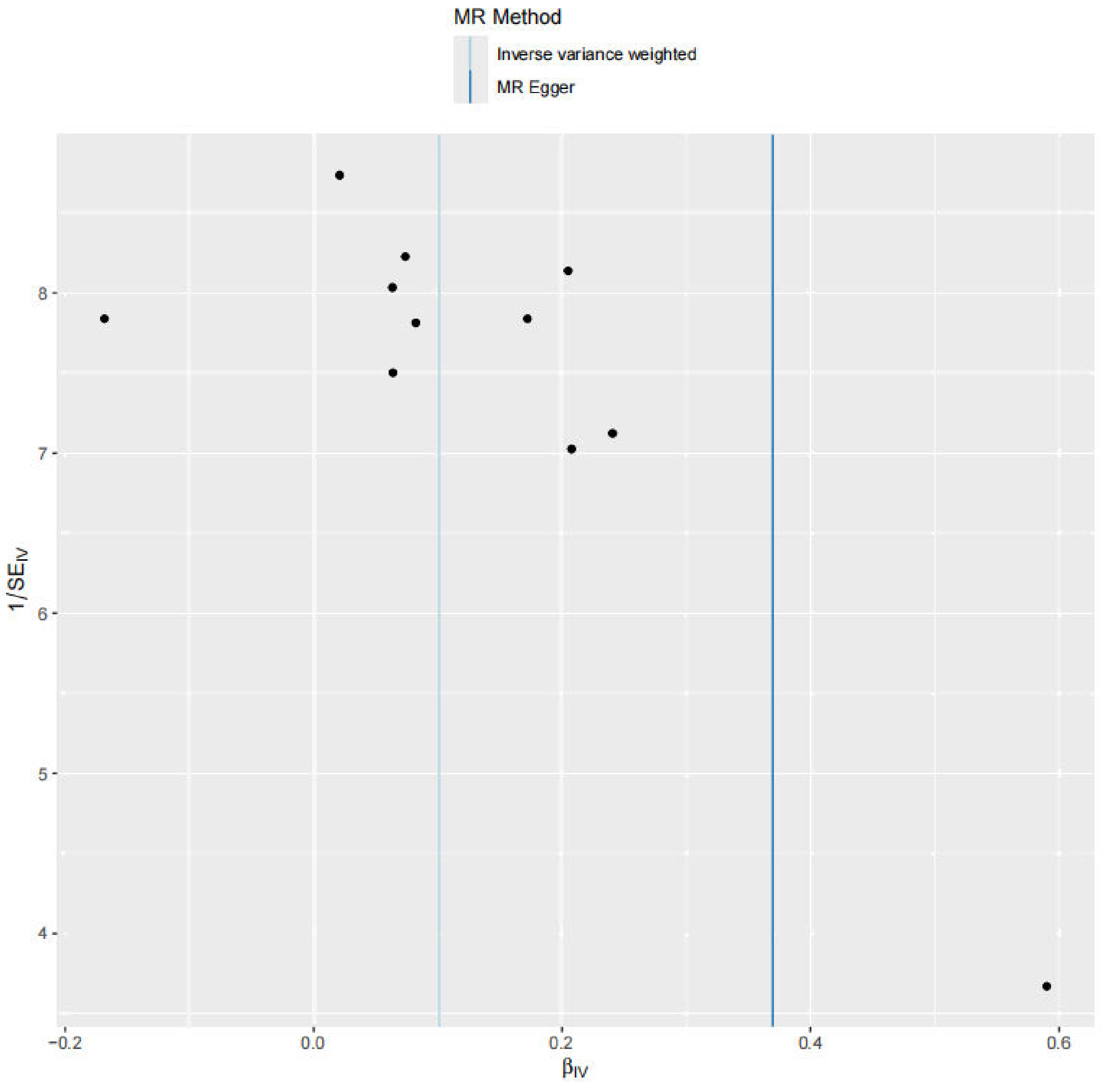

MR Method
Inverse variance weighted
MR Egger
8
7
6
5
4
1/SE$_{IV}$
-0.2
0.0
0.2
0.4
0.6
β$_{IV}$

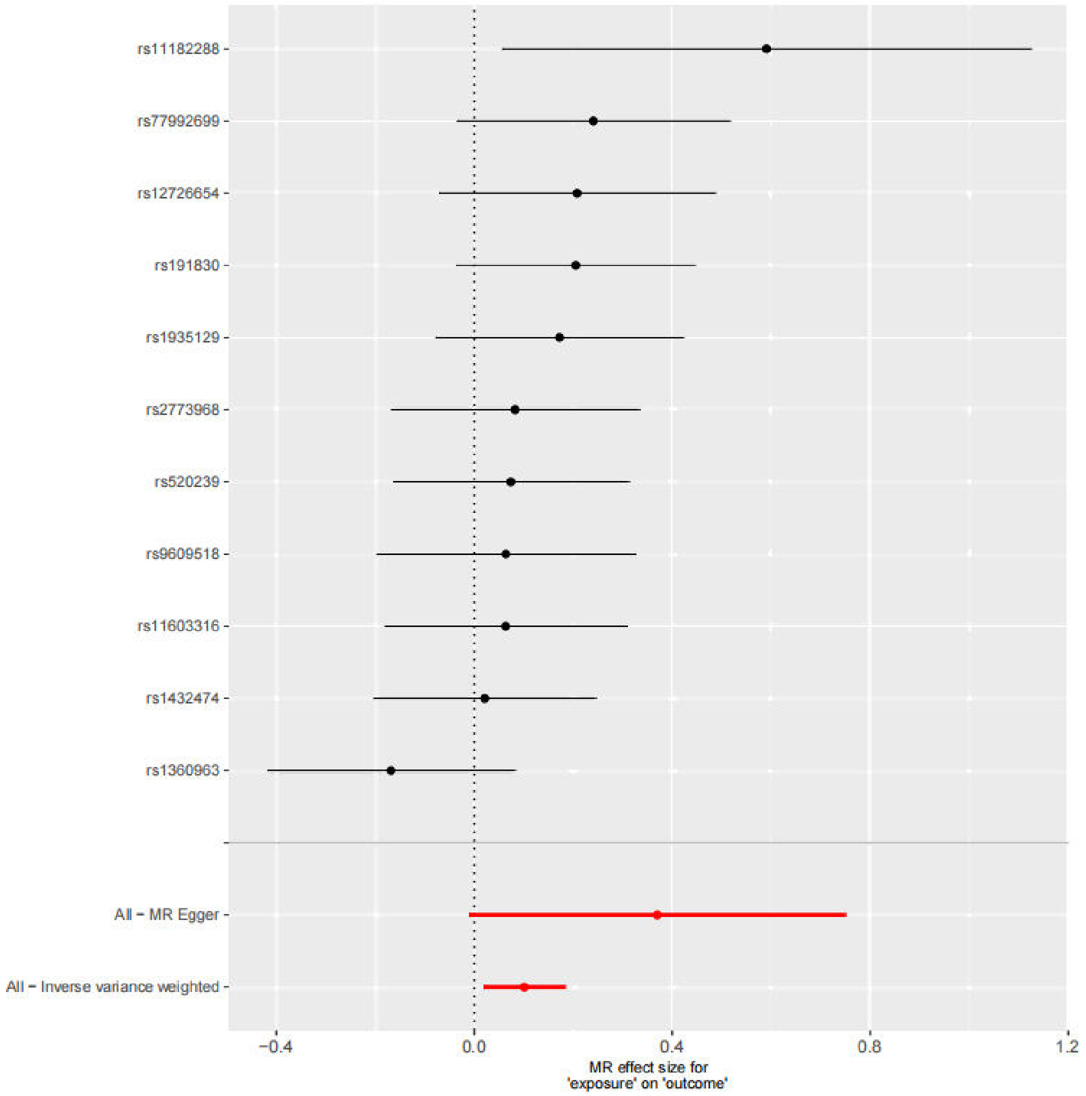
rs11182288
rs77992699
rs12726654
rs191830
rs1935129
rs2773968
rs520239
rs9609518
rs11603316
rs1432474
rs1360963
All − MR Egger
All − Inverse variance weighted
−0.4
0.0
0.4
0.8
1.2
MR effect size for
'exposure' on 'outcome'

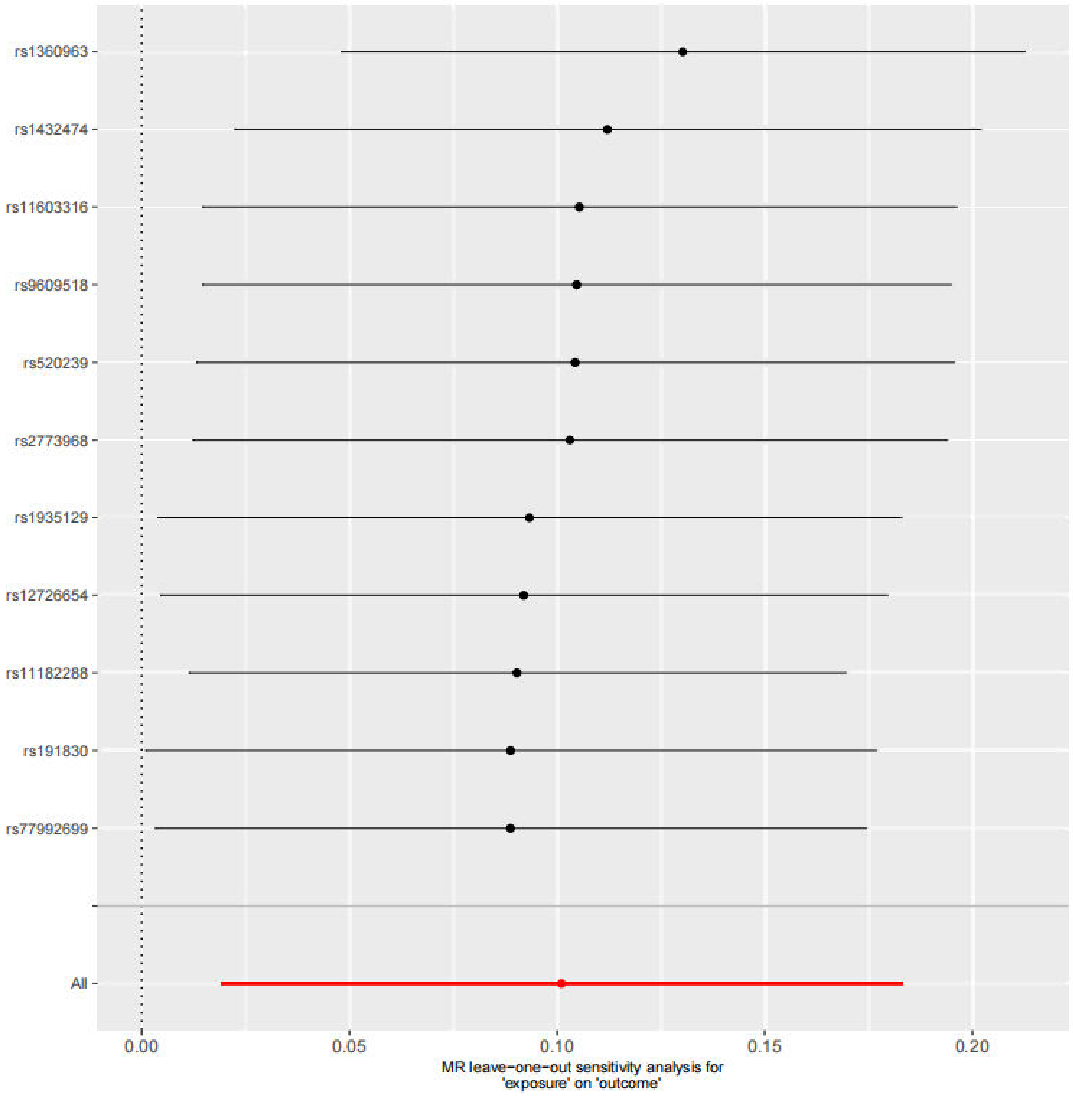

rs1360963
rs1432474
rs11603316
rs9609518
rs520239
rs2773968
rs1935129
rs12726654
rs11182288
rs191830
rs77992699
All
0.00
0.05
0.10
0.15
0.20
MR leave−one−out sensitivity analysis for
'exposure' on 'outcome'

GCST90257027

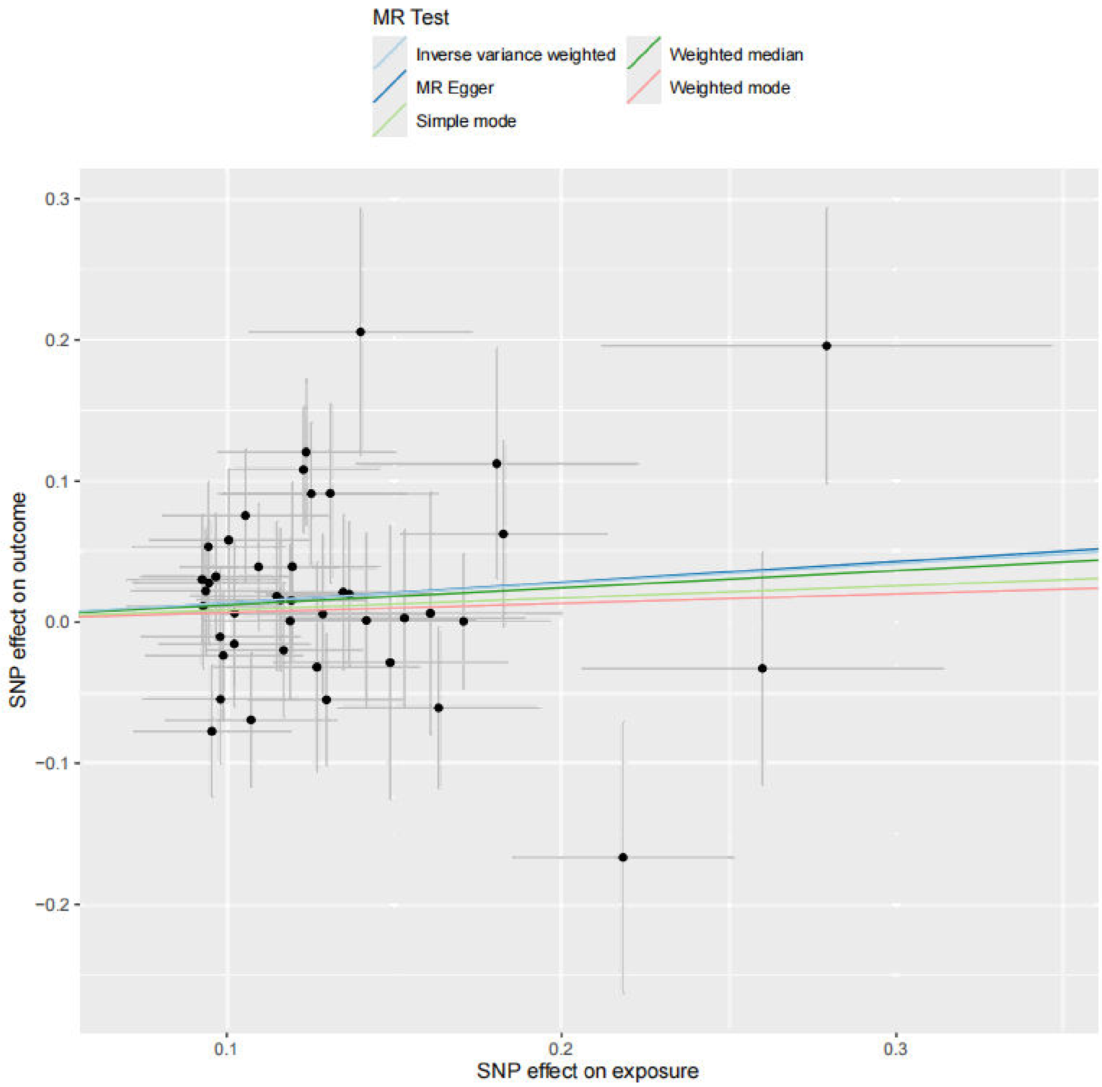

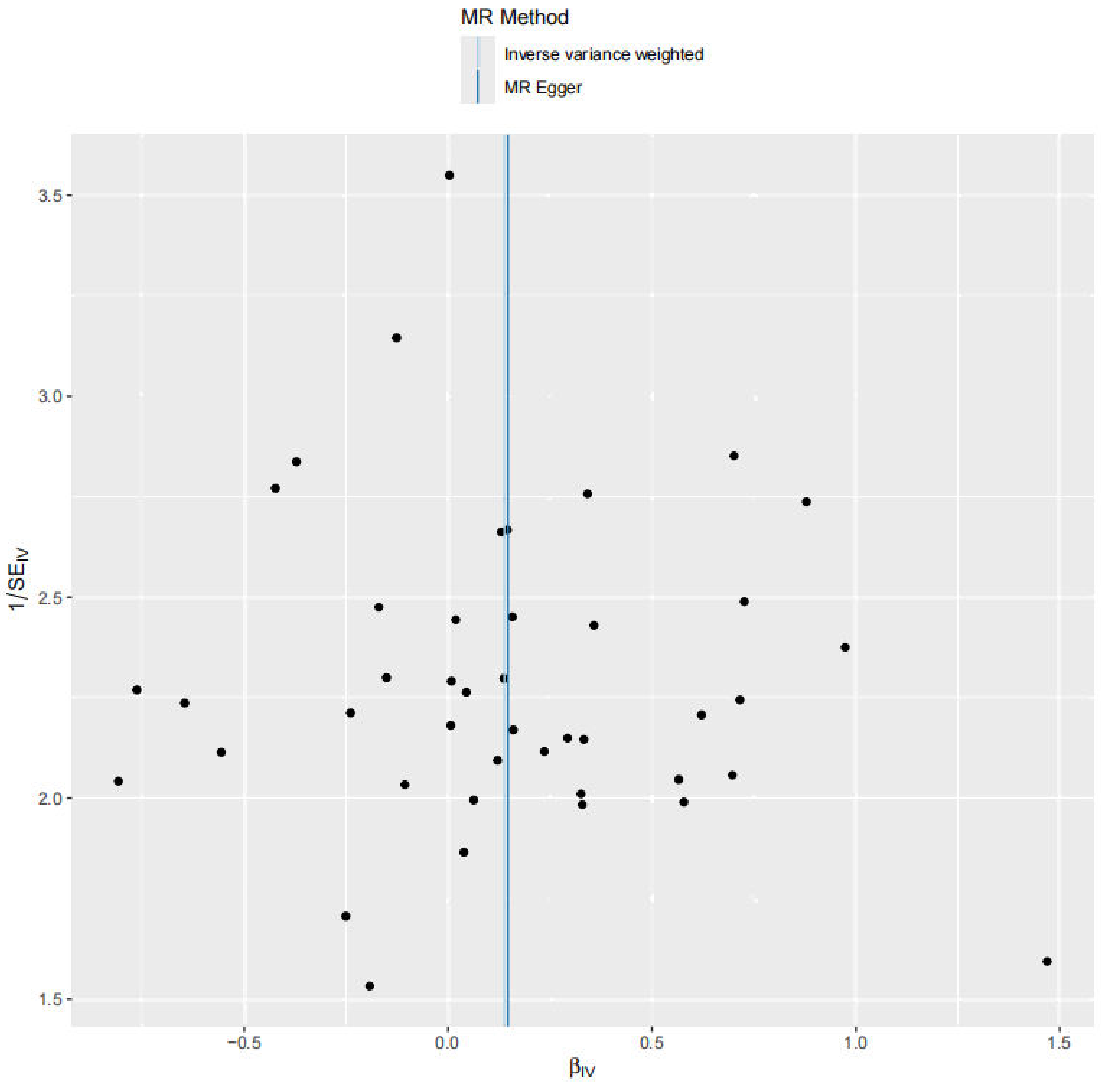
MR Method
Inverse variance weighted
MR Egger
3.5
3.0
2.5
2.0
1.5
1/SE_IV
−0.5
0.0
0.5
1.0
1.5
β_IV

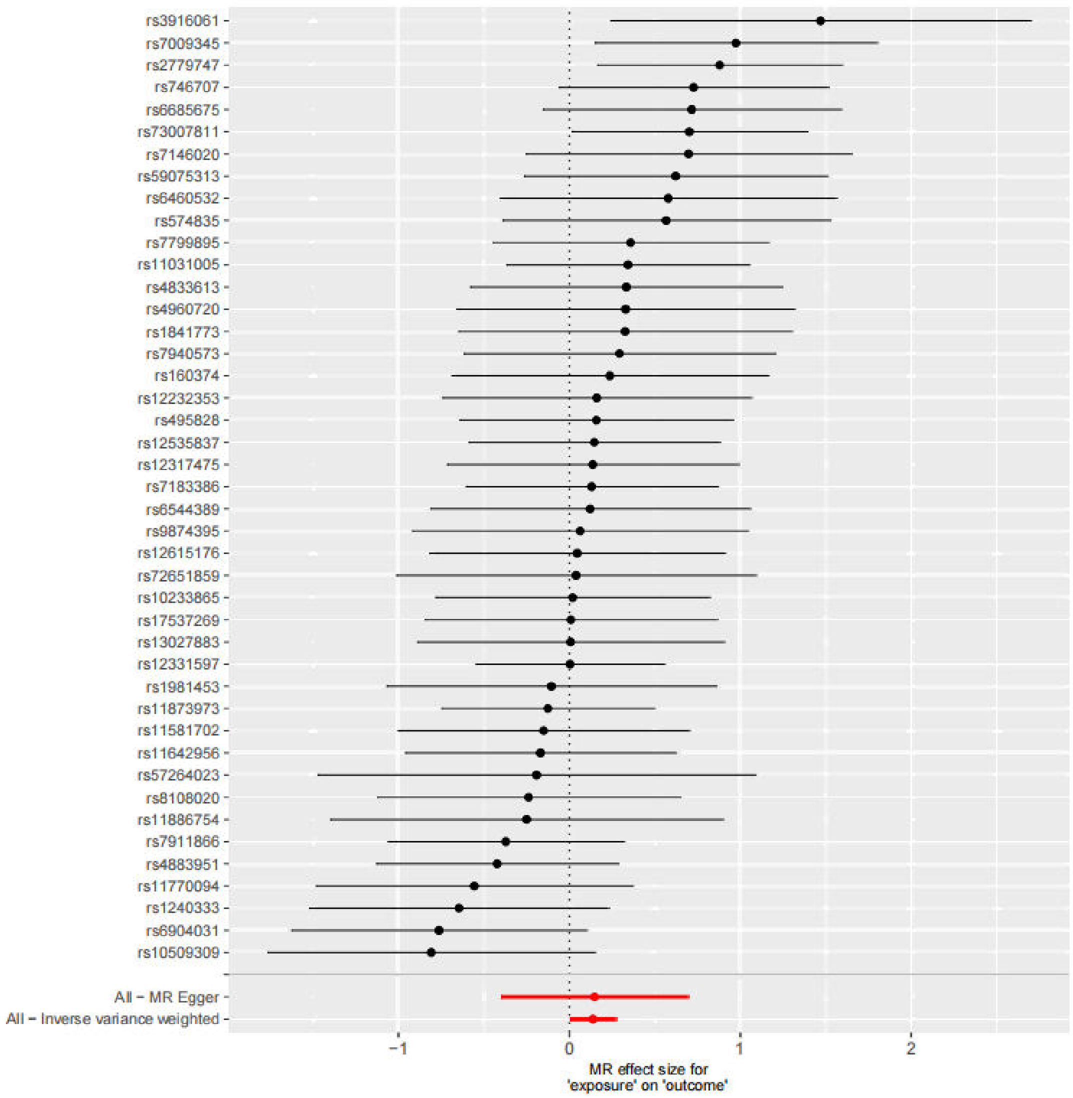
rs3916061
rs7009345
rs2779747
rs746707
rs6685675
rs73007811
rs7146020
rs59075313
rs6460532
rs574835
rs7799895
rs11031005
rs4833613
rs4960720
rs1841773
rs7940573
rs160374
rs12232353
rs495828
rs12535837
rs12317475
rs7183386
rs6544389
rs9874395
rs12615176
rs72651859
rs10233865
rs17537269
rs13027883
rs12331597
rs1981453
rs11873973
rs11581702
rs11642956
rs57264023
rs8108020
rs11886754
rs7911866
rs4883951
rs11770094
rs1240333
rs6904031
rs10509309
All − MR Egger
All − Inverse variance weighted
−1
0
1
2
MR effect size for
'exposure' on 'outcome'

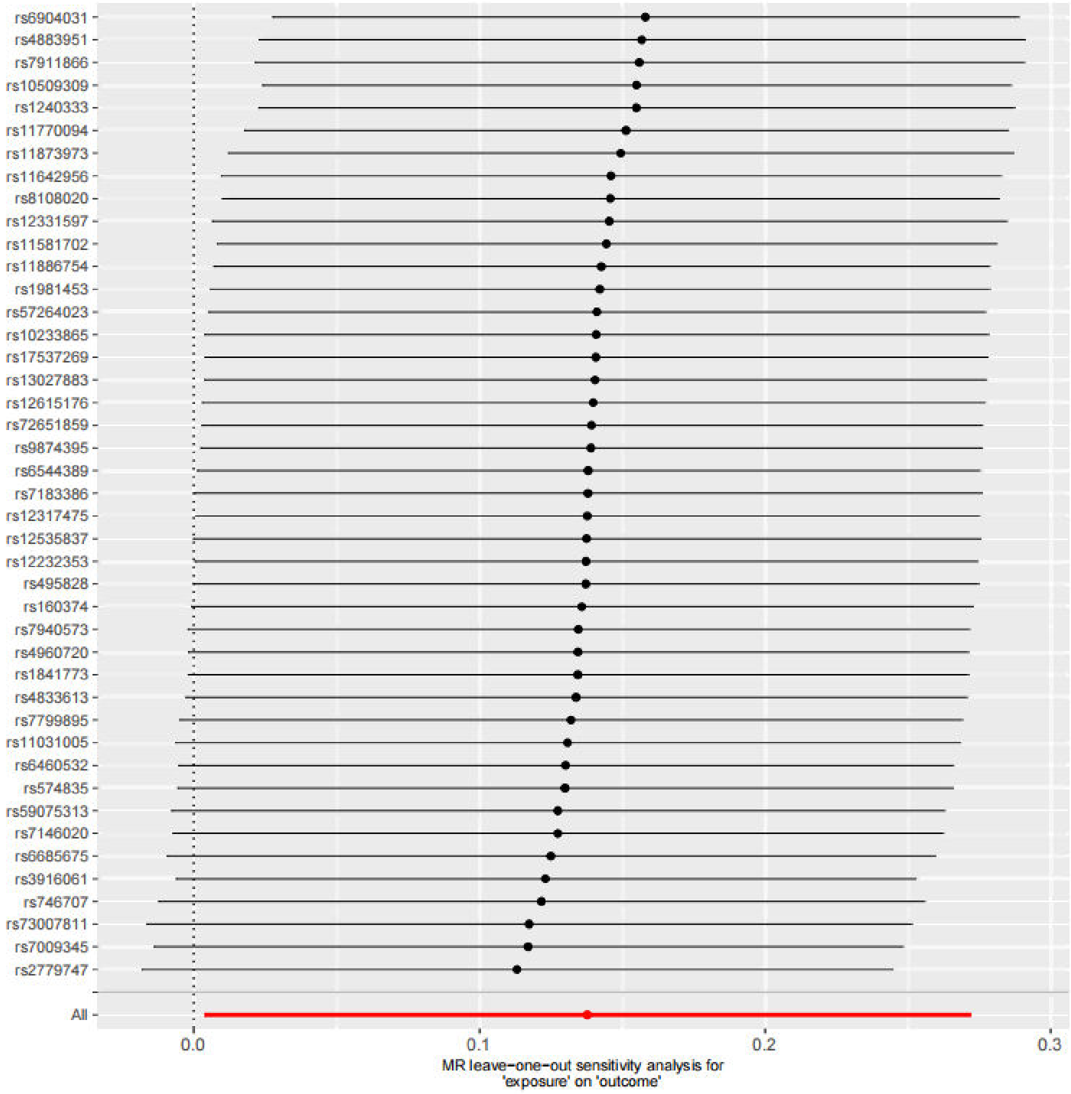
rs6904031
rs4883951
rs7911866
rs10509309
rs1240333
rs11770094
rs11873973
rs11642956
rs8108020
rs12331597
rs11581702
rs11886754
rs1981453
rs57264023
rs10233865
rs17537269
rs13027883
rs12615176
rs72651859
rs9874395
rs6544389
rs7183386
rs12317475
rs12535837
rs12232353
rs495828
rs160374
rs7940573
rs4960720
rs1841773
rs4833613
rs7799895
rs11031005
rs6460532
rs574835
rs59075313
rs7146020
rs6685675
rs3916061
rs746707
rs73007811
rs7009345
rs2779747
All
0.0
0.1
0.2
0.3
MR leave-one-out sensitivity analysis for
'exposure' on 'outcome'

GCST90257042

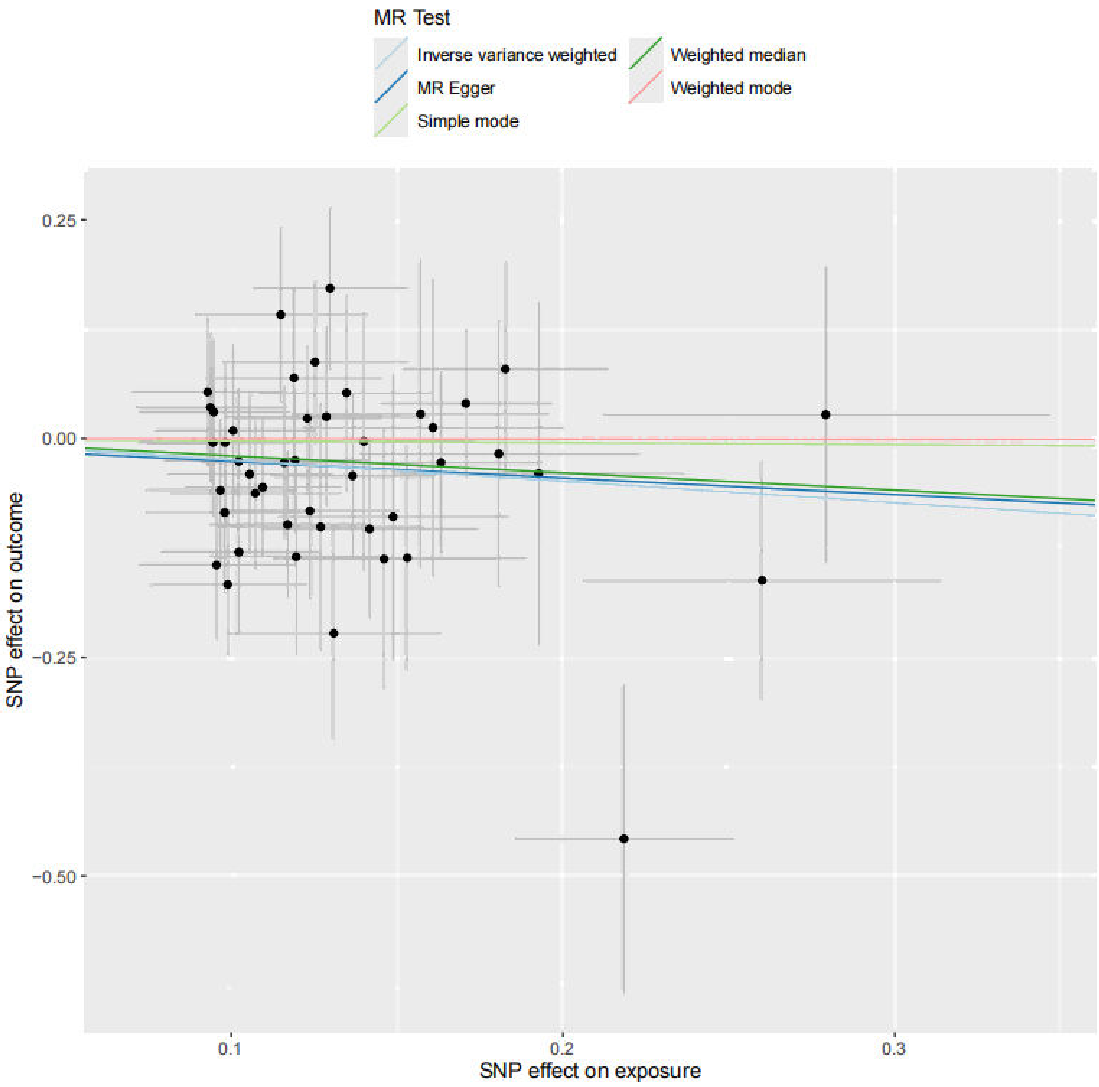

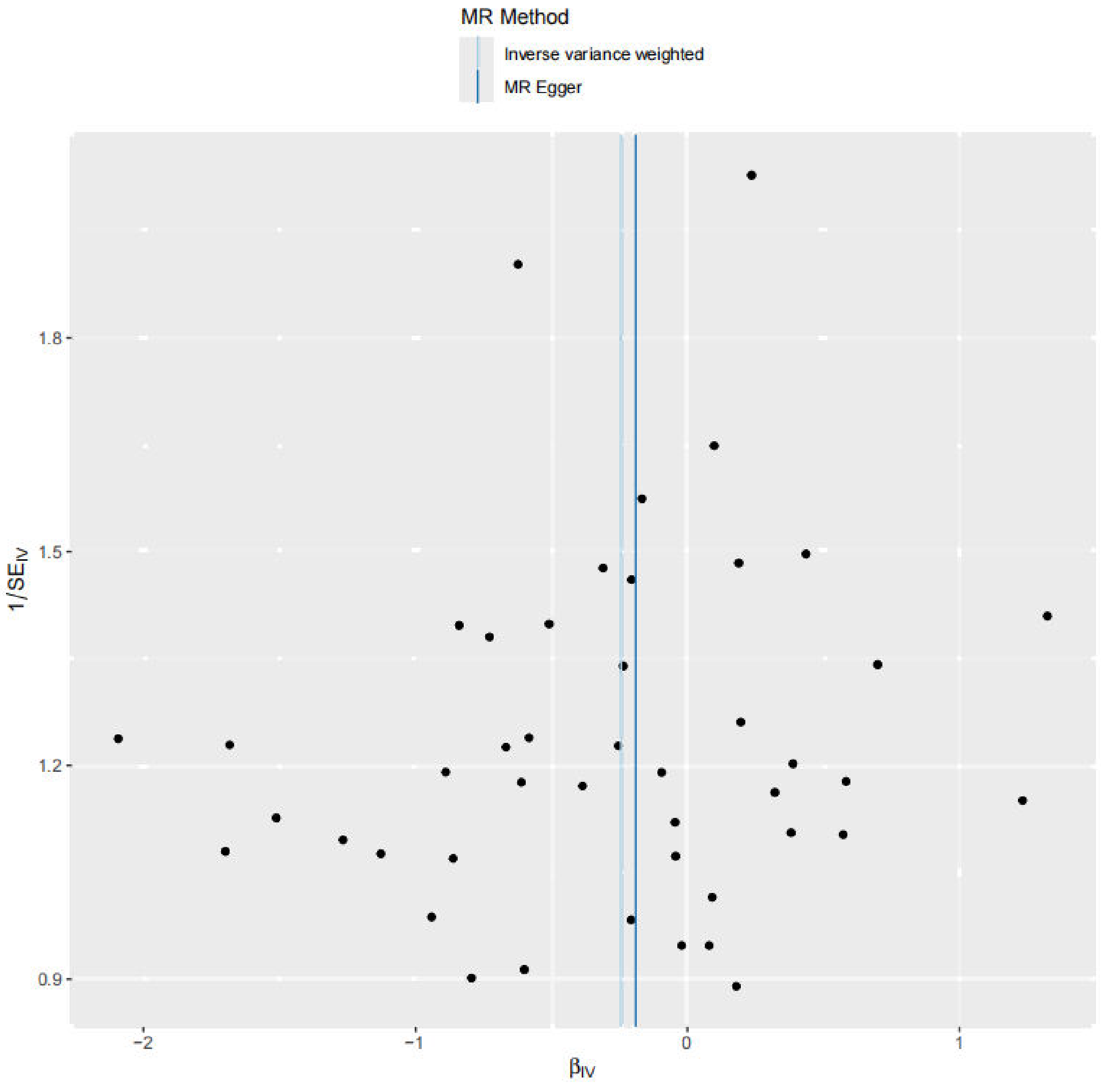
MR Method
Inverse variance weighted
MR Egger
1.8
1.5
1.2
0.9
$1/SE_{IV}$
−2
−1
0
1
β_{IV}

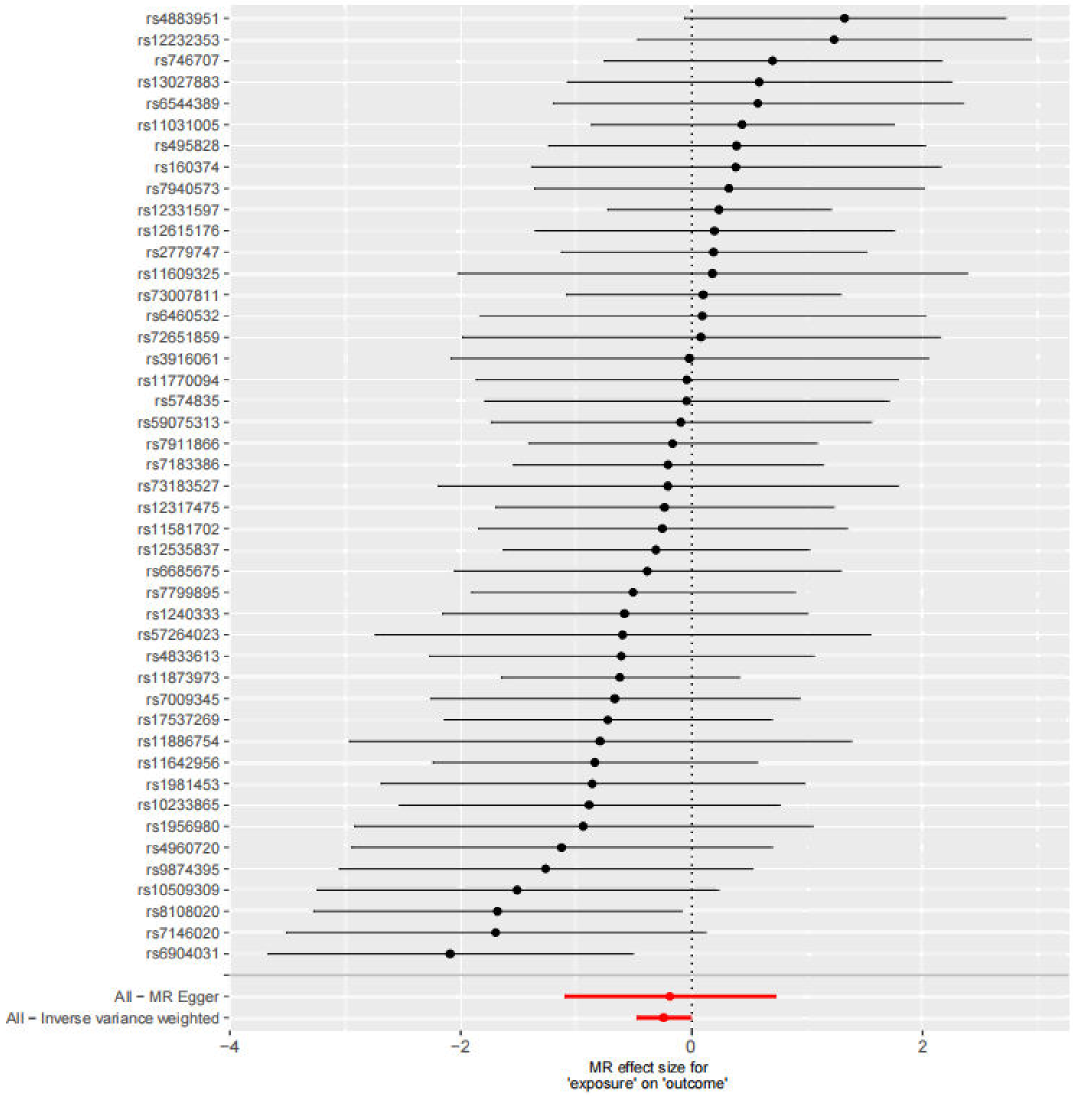

rs4883951
rs12232353
rs746707
rs13027883
rs6544389
rs11031005
rs495828
rs160374
rs7940573
rs12331597
rs12615176
rs2779747
rs11609325
rs73007811
rs6460532
rs72651859
rs3916061
rs11770094
rs574835
rs59075313
rs7911866
rs7183386
rs73183527
rs12317475
rs11581702
rs12535837
rs6685675
rs7799895
rs1240333
rs57264023
rs4833613
rs11873973
rs7009345
rs17537269
rs11886754
rs11642956
rs1981453
rs10233865
rs1956980
rs4960720
rs9874395
rs10509309
rs8108020
rs7146020
rs6904031
All − MR Egger
All − Inverse variance weighted
−4
−2
0
2
MR effect size for
'exposure' on 'outcome'

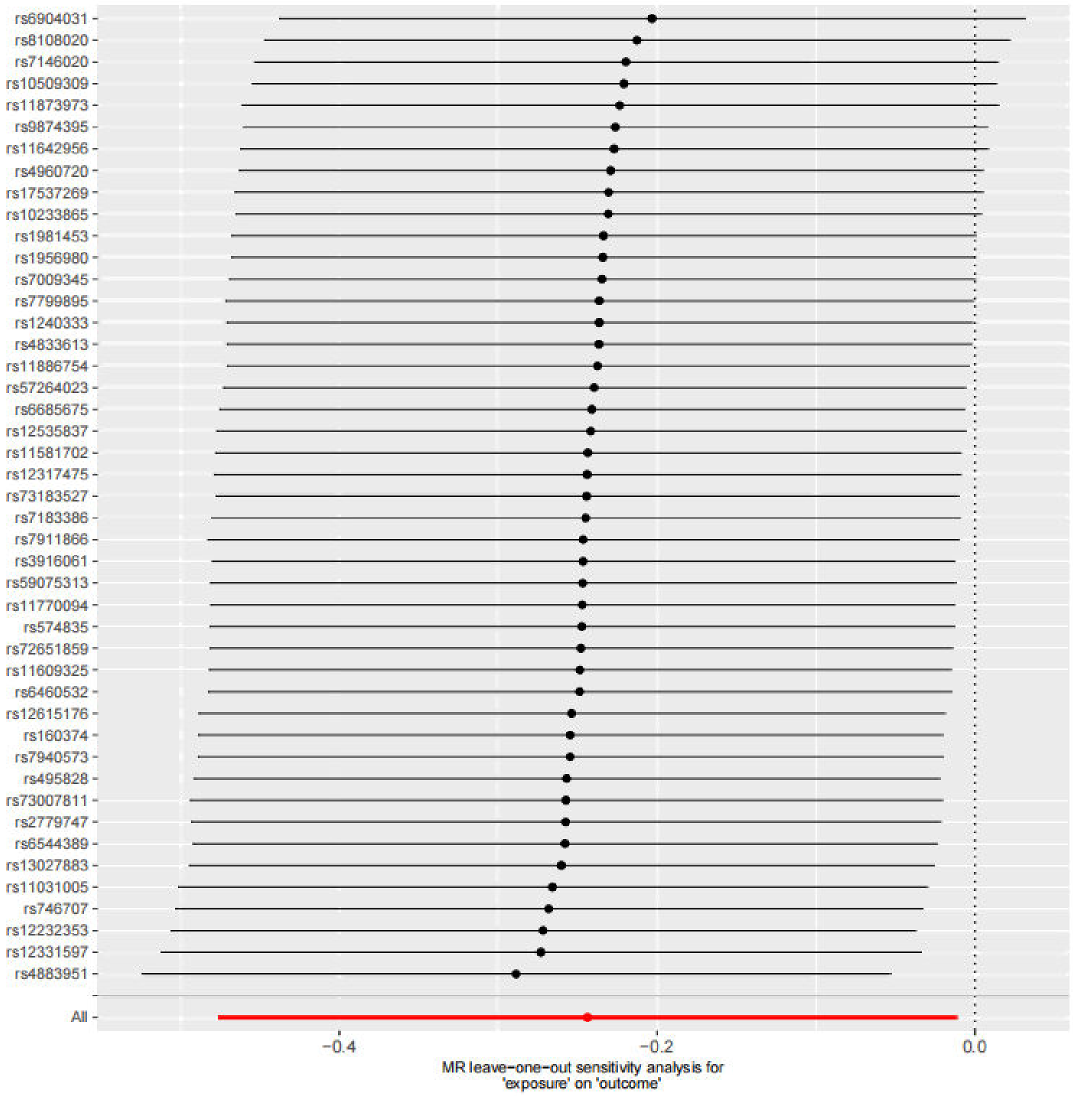
rs6904031
rs8108020
rs7146020
rs10509309
rs11873973
rs9874395
rs11642956
rs4960720
rs17537269
rs10233865
rs1981453
rs1956980
rs7009345
rs7799895
rs1240333
rs4833613
rs11886754
rs57264023
rs6685675
rs12535837
rs11581702
rs12317475
rs73183527
rs7183386
rs7911866
rs3916061
rs59075313
rs11770094
rs574835
rs72651859
rs11609325
rs6460532
rs12615176
rs160374
rs7940573
rs495828
rs73007811
rs2779747
rs6544389
rs13027883
rs11031005
rs746707
rs12232353
rs12331597
rs4883951
All
−0.4
−0.2
0.0
MR leave−one−out sensitivity analysis for
'exposure' on 'outcome'

GCST90257043

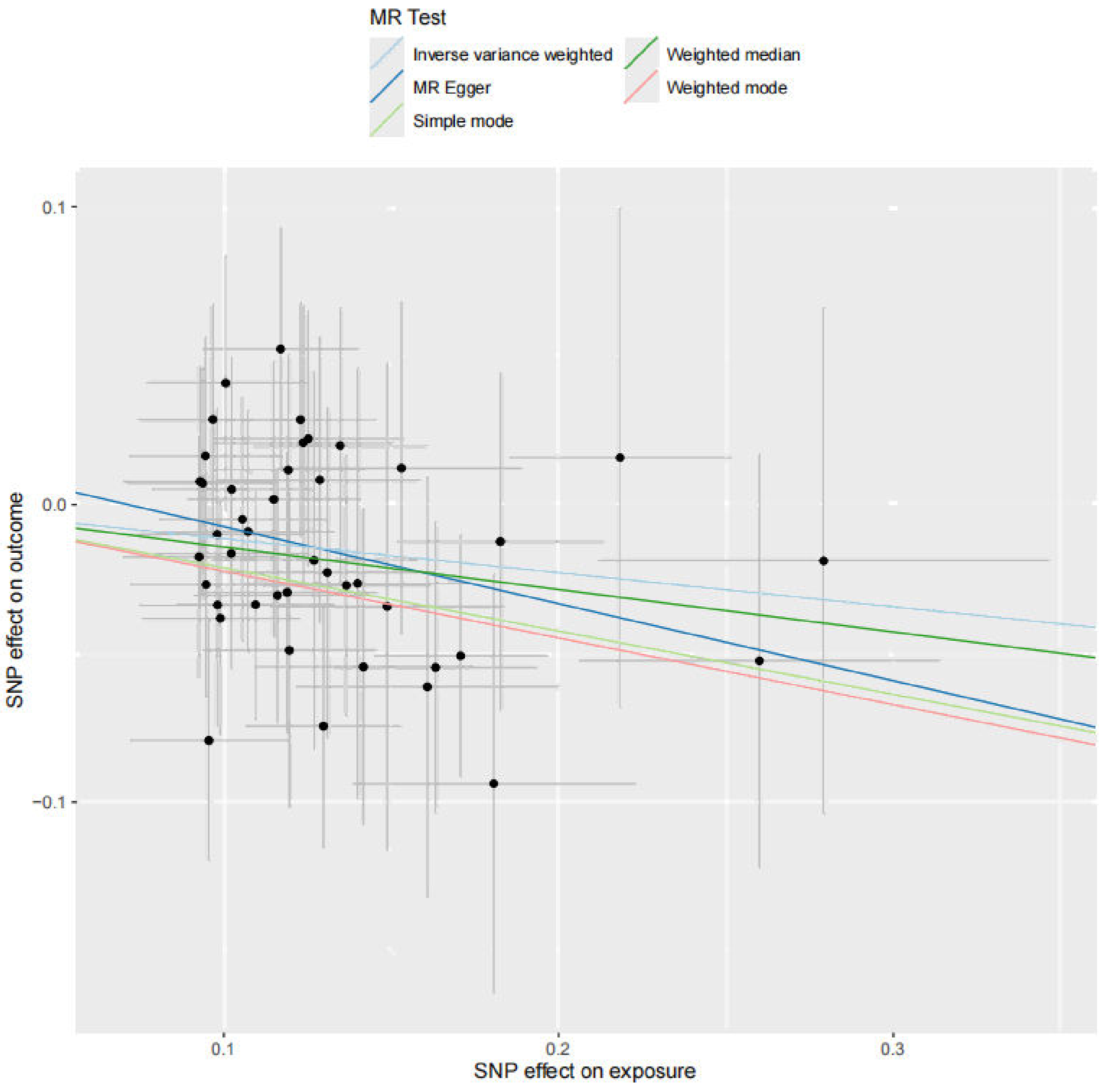

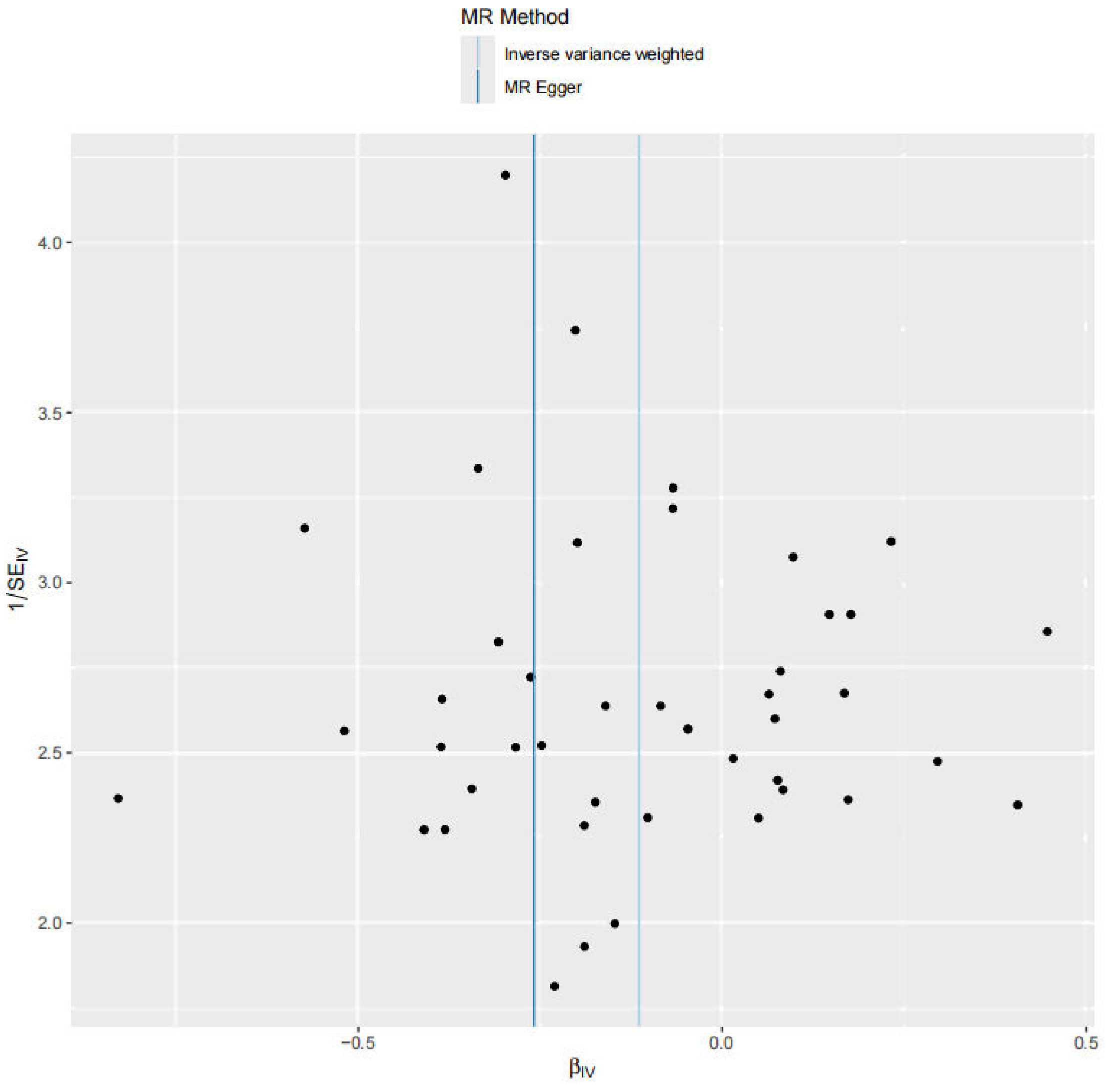
MR Method
Inverse variance weighted
MR Egger
4.0
3.5
3.0
2.5
2.0
1/SE_IV
−0.5
0.0
0.5
β_IV

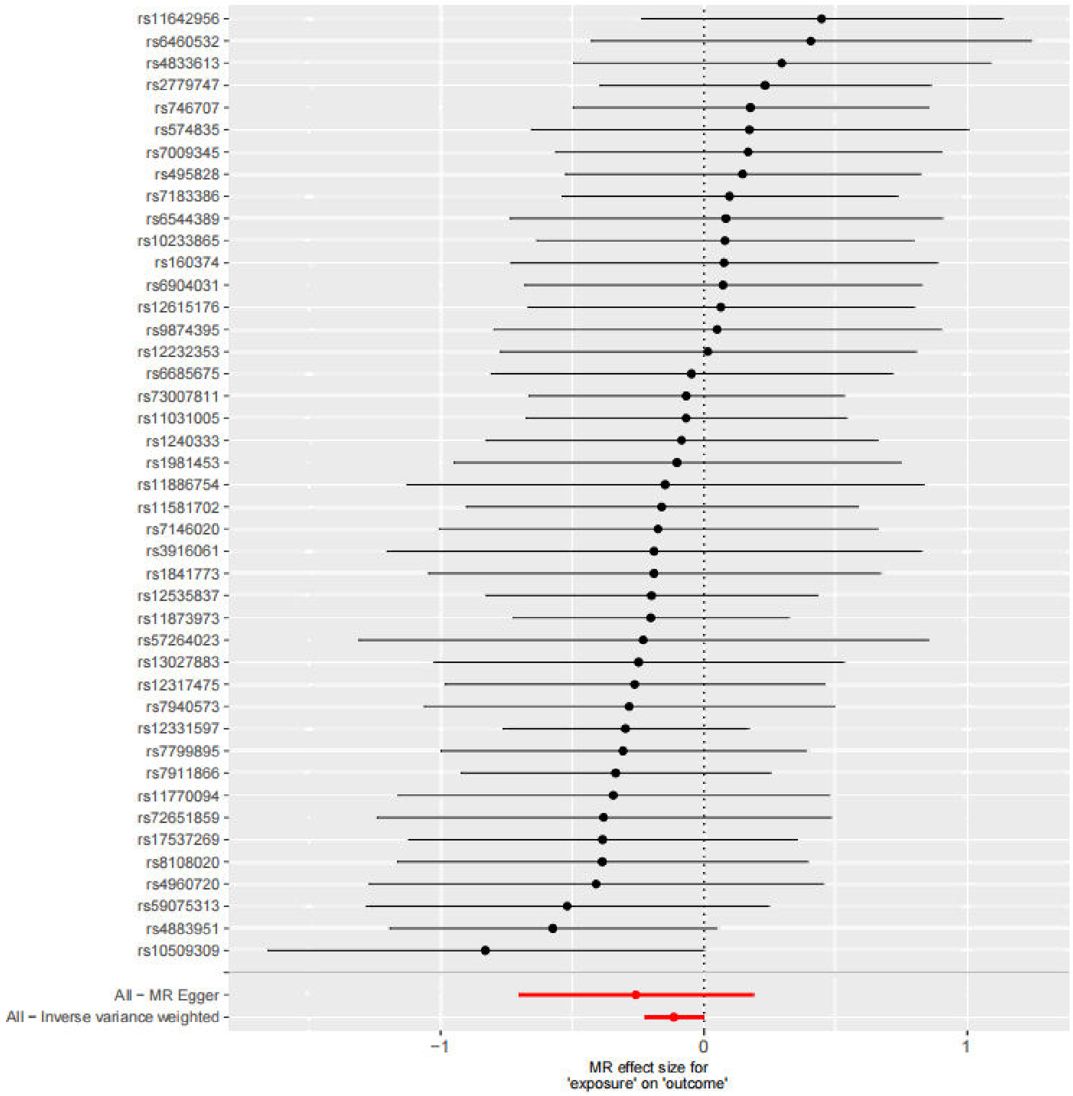
rs11642956
rs6460532
rs4833613
rs2779747
rs746707
rs574835
rs7009345
rs495828
rs7183386
rs6544389
rs10233865
rs160374
rs6904031
rs12615176
rs9874395
rs12232353
rs6685675
rs73007811
rs11031005
rs1240333
rs1981453
rs11886754
rs11581702
rs7146020
rs3916061
rs1841773
rs12535837
rs11873973
rs57264023
rs13027883
rs12317475
rs7940573
rs12331597
rs7799895
rs7911866
rs11770094
rs72651859
rs17537269
rs8108020
rs4960720
rs59075313
rs4883951
rs10509309
All − MR Egger
All − Inverse variance weighted
−1
0
1
MR effect size for
'exposure' on 'outcome'

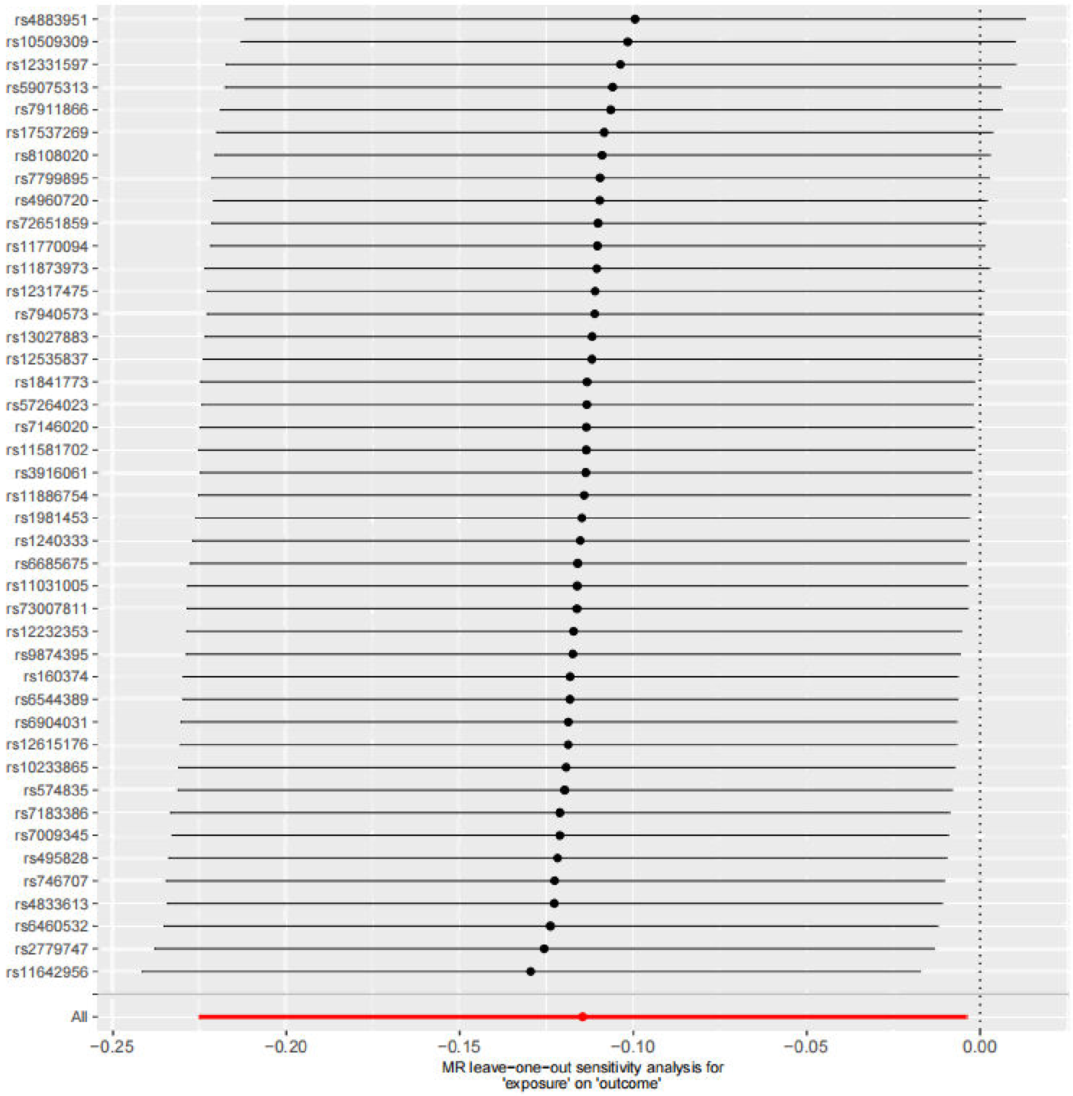
rs4883951
rs10509309
rs12331597
rs59075313
rs7911866
rs17537269
rs8108020
rs7799895
rs4960720
rs72651859
rs11770094
rs11873973
rs12317475
rs7940573
rs13027883
rs12535837
rs1841773
rs57264023
rs7146020
rs11581702
rs3916061
rs11886754
rs1981453
rs1240333
rs6685675
rs11031005
rs73007811
rs12232353
rs9874395
rs160374
rs6544389
rs6904031
rs12615176
rs10233865
rs574835
rs7183386
rs7009345
rs495828
rs746707
rs4833613
rs6460532
rs2779747
rs11642956
All
−0.25
−0.20
−0.15
−0.10
−0.05
0.00
MR leave−one−out sensitivity analysis for
'exposure' on 'outcome'

GCST90257071

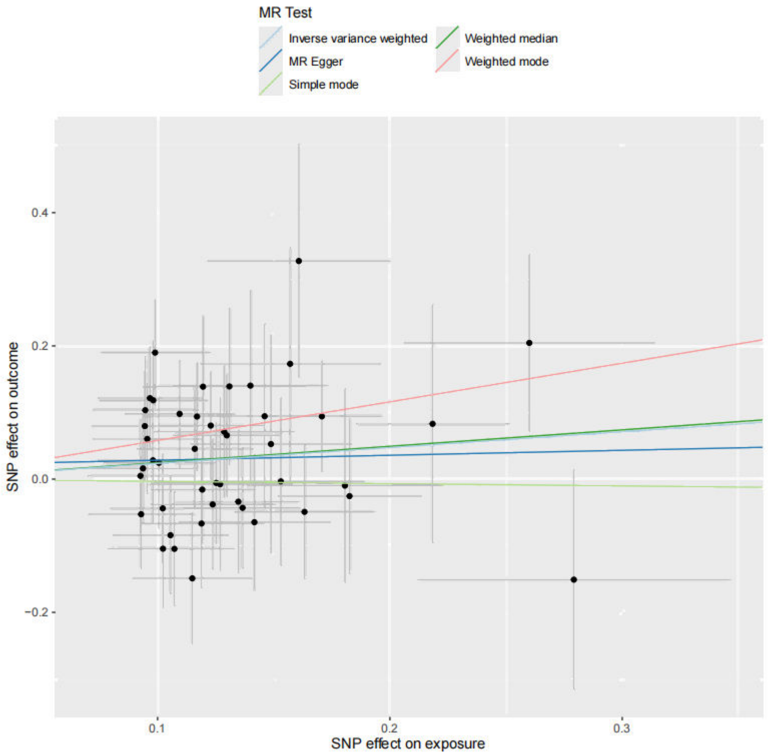

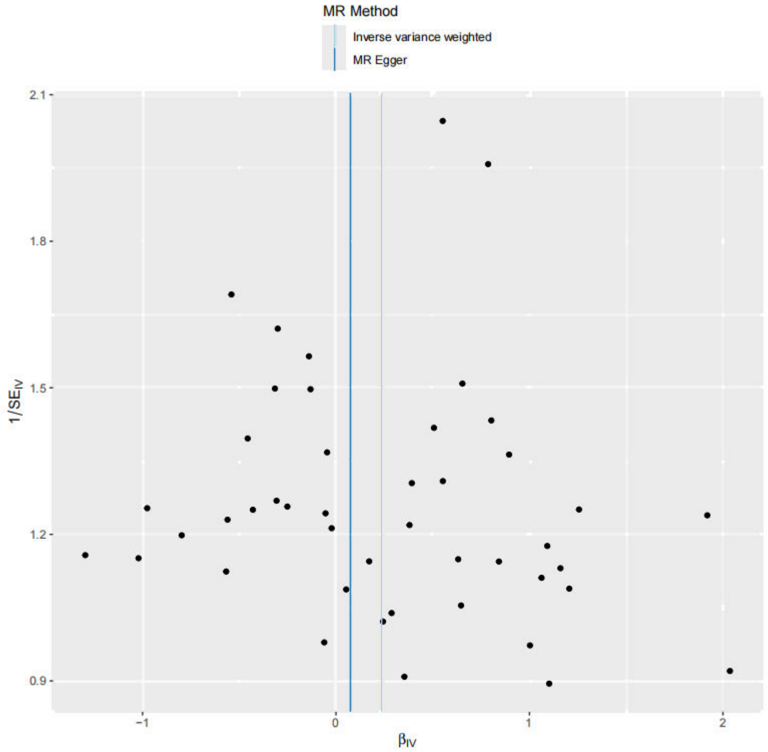

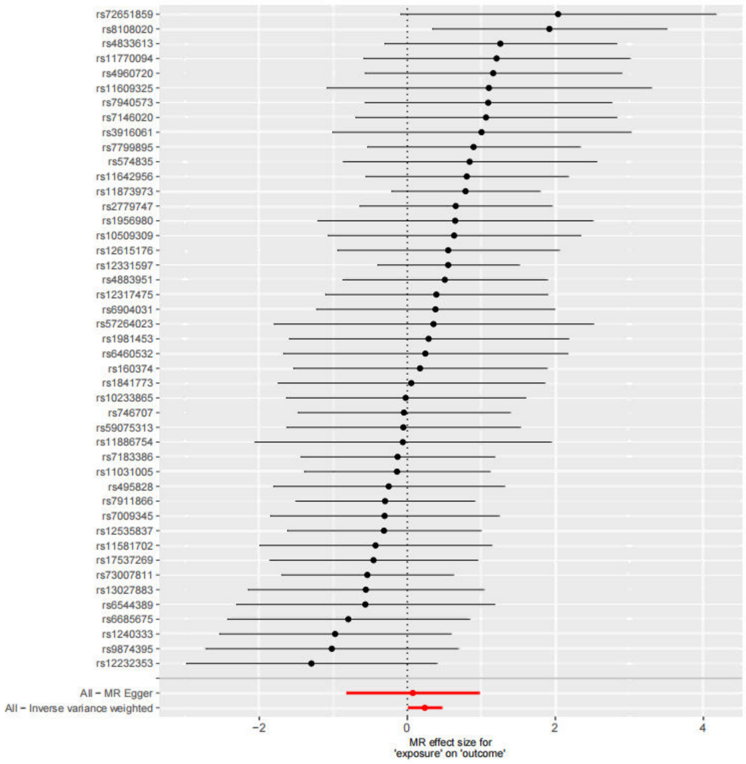

rs72651859
rs8108020
rs4833613
rs11770094
rs4960720
rs11609325
rs7940573
rs7146020
rs3916061
rs7799895
rs574835
rs11642956
rs11873973
rs2779747
rs1956980
rs10509309
rs12615176
rs12331597
rs4883951
rs12317475
rs6904031
rs57264023
rs1981453
rs6460532
rs160374
rs1841773
rs10233865
rs746707
rs59075313
rs11886754
rs7183386
rs11031005
rs495828
rs7911866
rs7009345
rs12535837
rs11581702
rs17537269
rs73007811
rs13027883
rs6544389
rs6685675
rs1240333
rs9874395
rs12232353
All − MR Egger
All − Inverse variance weighted
−2
0
2
4
MR effect size for
'exposure' on 'outcome'

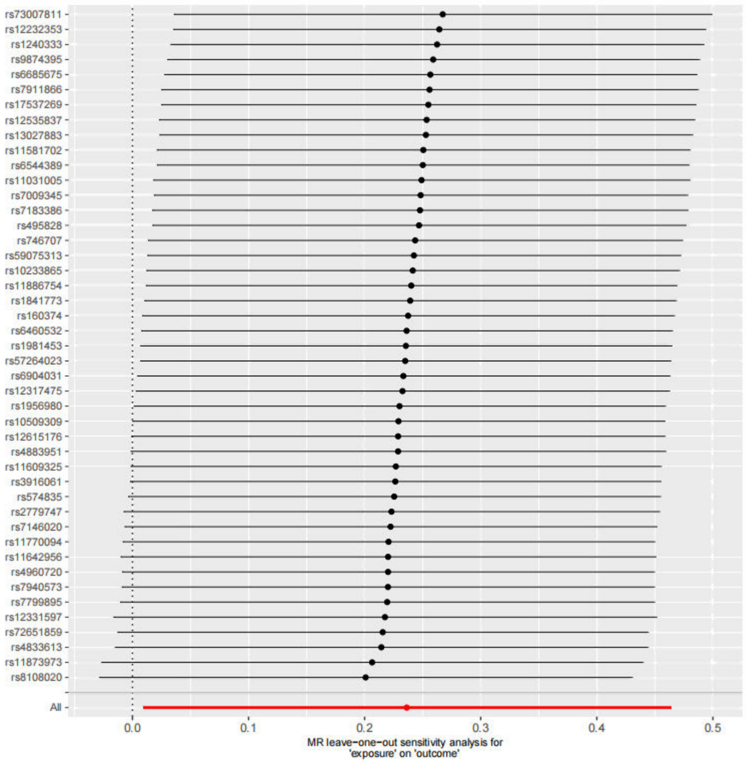

rs73007811
rs12232353
rs1240333
rs9874395
rs6685675
rs7911866
rs17537269
rs12535837
rs13027883
rs11581702
rs6544389
rs11031005
rs7009345
rs7183386
rs495828
rs746707
rs59075313
rs10233865
rs11886754
rs1841773
rs160374
rs6460532
rs1981453
rs57264023
rs6904031
rs12317475
rs1956980
rs10509309
rs12615176
rs4883951
rs11609325
rs3916061
rs574835
rs2779747
rs7146020
rs11770094
rs11642956
rs4960720
rs7940573
rs7799895
rs12331597
rs72651859
rs4833613
rs11873973
rs8108020
All
0.0
0.1
0.2
0.3
0.4
0.5
MR leave−one−out sensitivity analysis for
'exposure' on 'outcome'

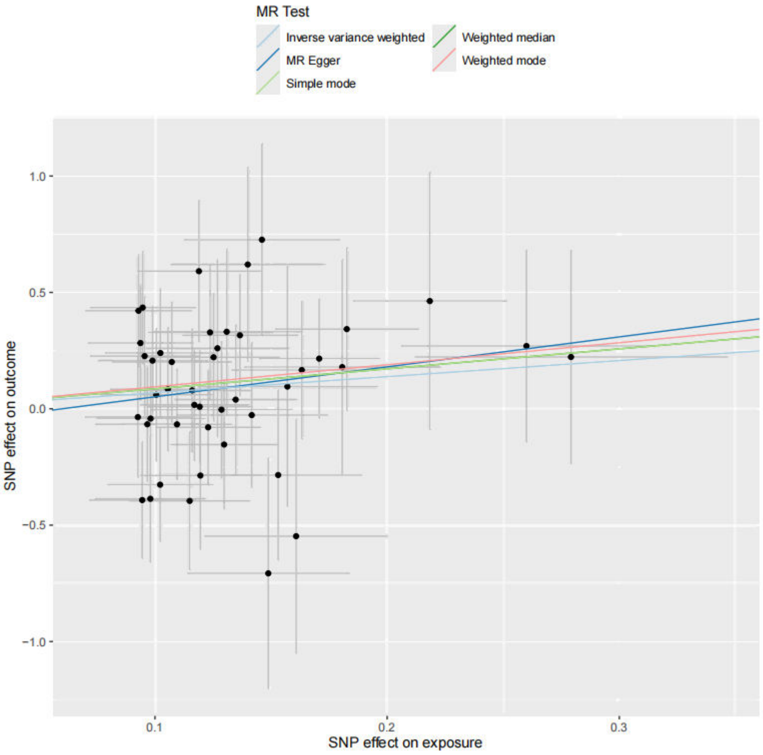

GCST90257100

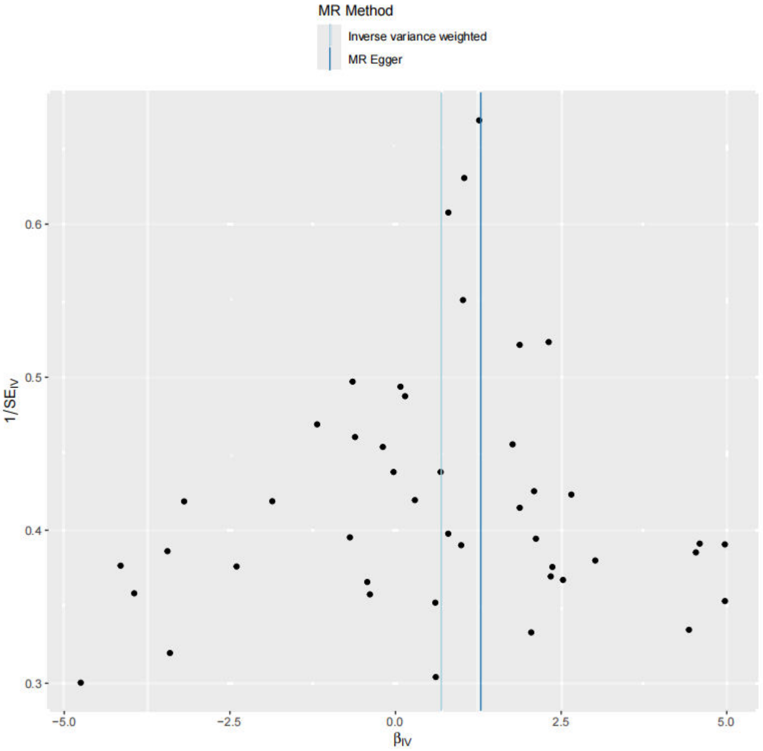

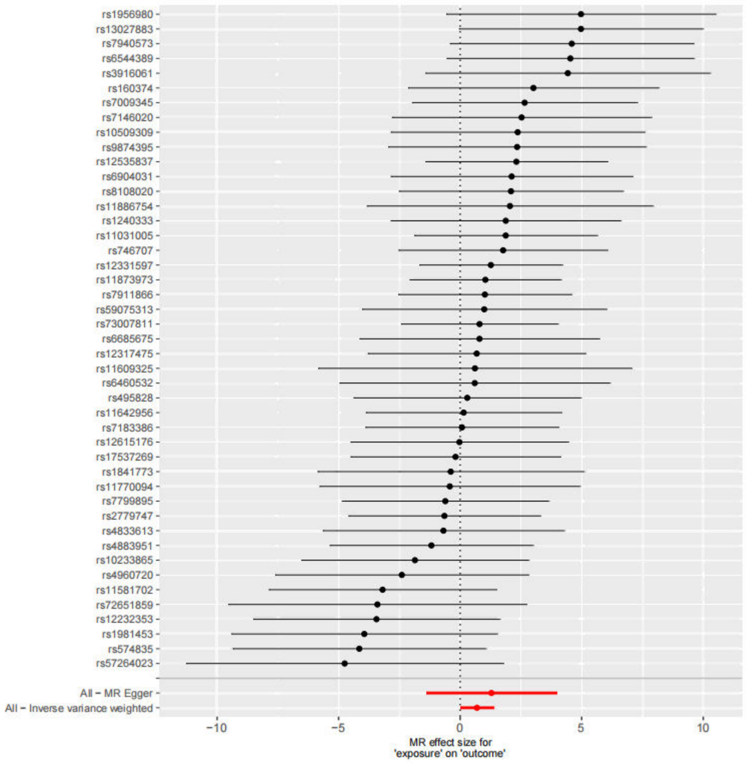
rs1956980
rs13027883
rs7940573
rs6544389
rs3916061
rs160374
rs7009345
rs7146020
rs10509309
rs9874395
rs12535837
rs6904031
rs8108020
rs11886754
rs1240333
rs11031005
rs746707
rs12331597
rs11873973
rs7911866
rs59075313
rs73007811
rs6685675
rs12317475
rs11609325
rs6460532
rs495828
rs11642956
rs7183386
rs12615176
rs17537269
rs1841773
rs11770094
rs7799895
rs2779747
rs4833613
rs4883951
rs10233865
rs4960720
rs11581702
rs72651859
rs12232353
rs1981453
rs574835
rs57264023
All − MR Egger
All − Inverse variance weighted
−10
−5
0
5
10
MR effect size for
'exposure' on 'outcome'

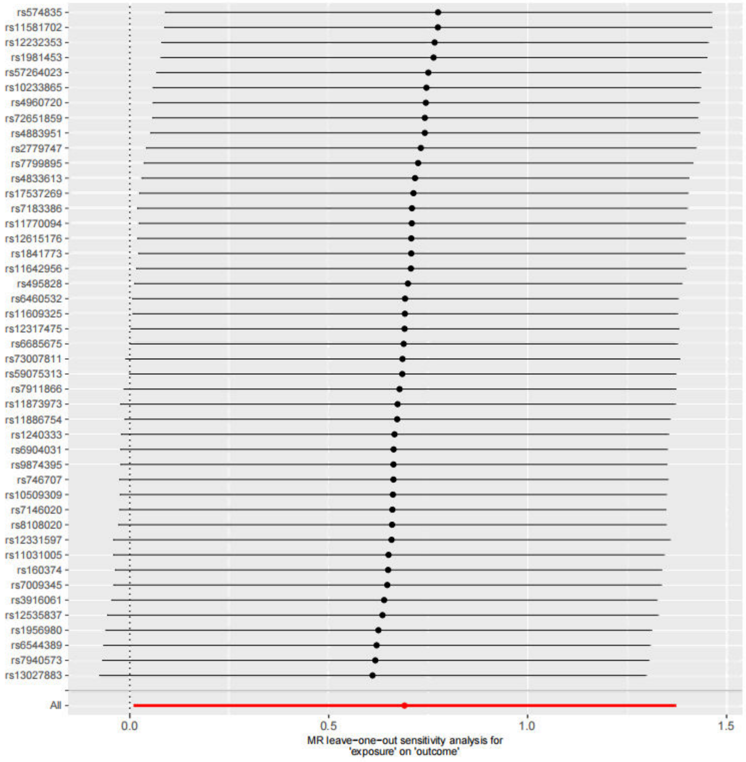
rs574835
rs11581702
rs12232353
rs1981453
rs57264023
rs10233865
rs4960720
rs72651859
rs4883951
rs2779747
rs7799895
rs4833613
rs17537269
rs7183386
rs11770094
rs12615176
rs1841773
rs11642956
rs495828
rs6460532
rs11609325
rs12317475
rs6685675
rs73007811
rs59075313
rs7911866
rs11873973
rs11886754
rs1240333
rs6904031
rs9874395
rs746707
rs10509309
rs7146020
rs8108020
rs12331597
rs11031005
rs160374
rs7009345
rs3916061
rs12535837
rs1956980
rs6544389
rs7940573
rs13027883
All
0.0
0.5
1.0
1.5
MR leave−one−out sensitivity analysis for
'exposure' on 'outcome'

GCST90257060

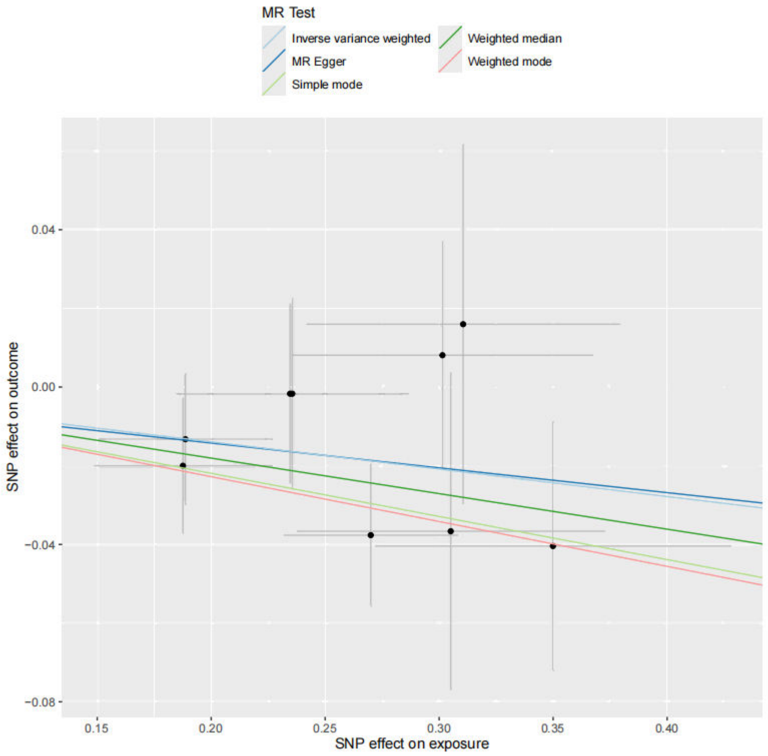

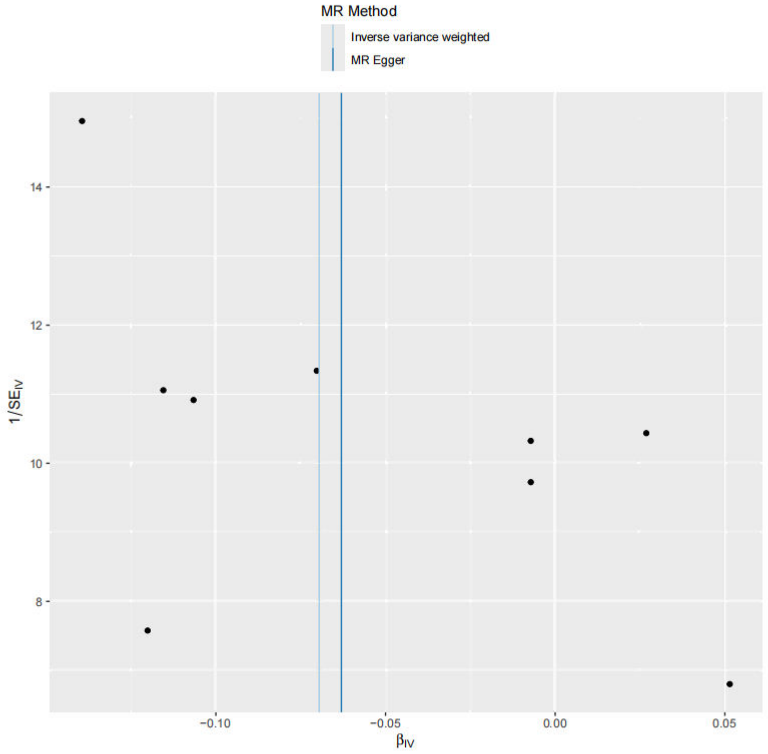

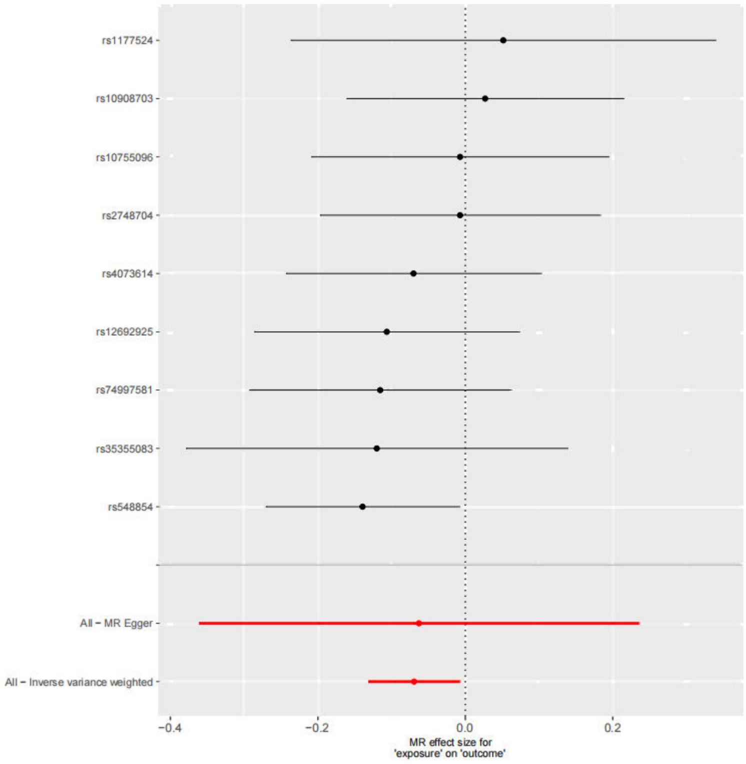
rs1177524
rs10908703
rs10755096
rs2748704
rs4073614
rs12692925
rs74997581
rs35355083
rs548854
All − MR Egger
All − Inverse variance weighted
−0.4
−0.2
0.0
0.2
MR effect size for
'exposure' on 'outcome'

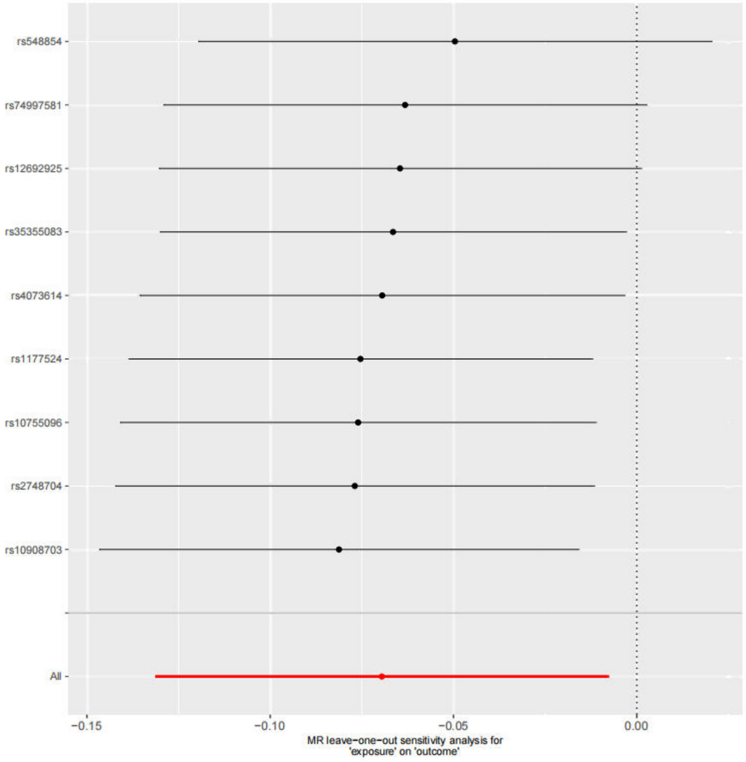
rs548854
rs74997581
rs12692925
rs35355083
rs4073614
rs1177524
rs10755096
rs2748704
rs10908703
All
−0.15
−0.10
−0.05
0.00
MR leave−one−out sensitivity analysis for
'exposure' on 'outcome'

GCST90257071

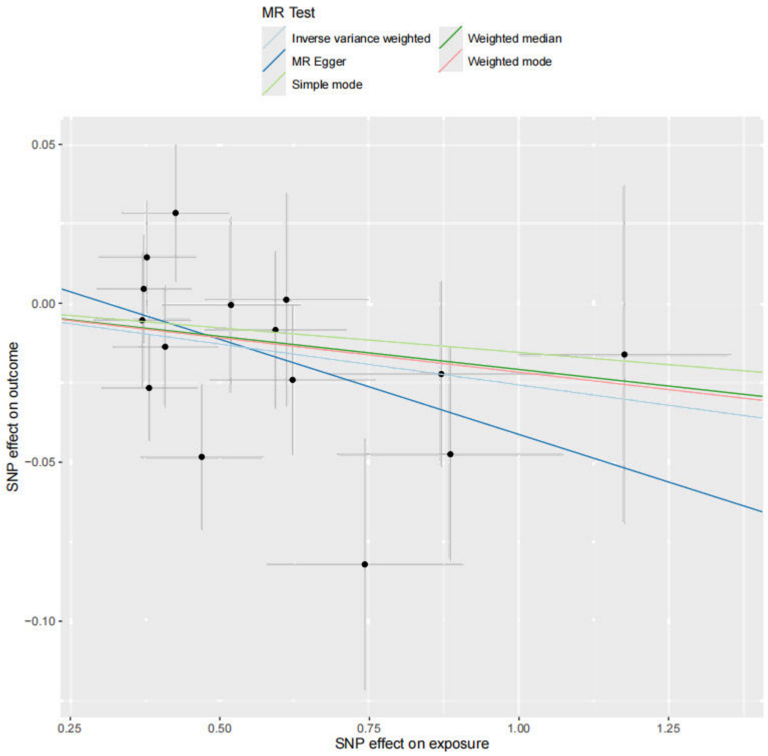

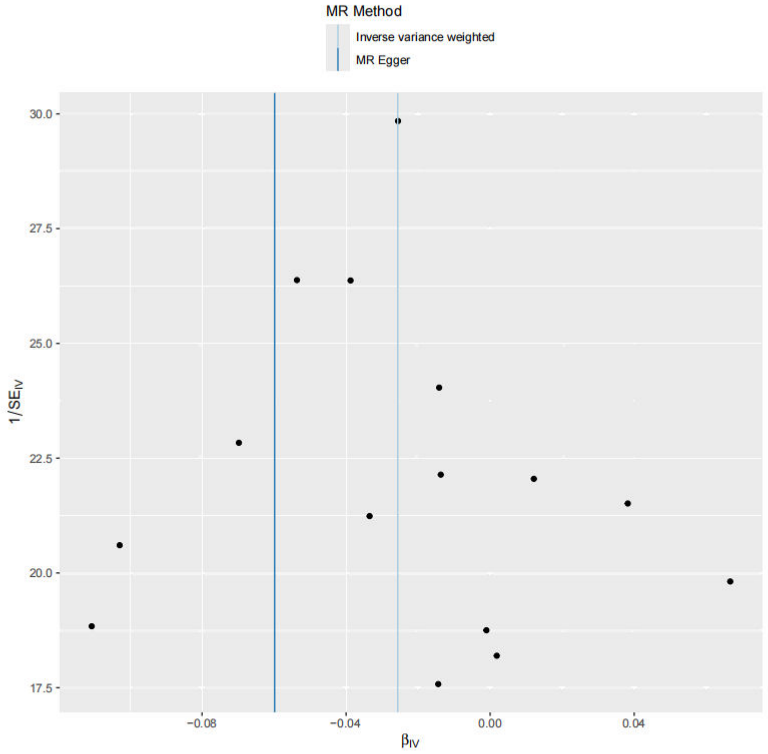

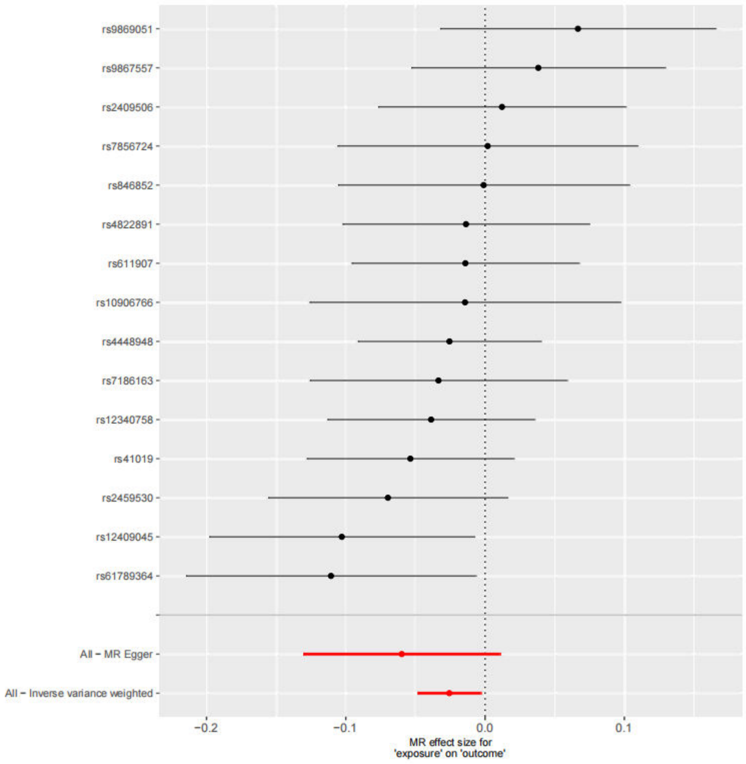
rs9869051
rs9867557
rs2409506
rs7856724
rs846852
rs4822891
rs611907
rs10906766
rs4448948
rs7186163
rs12340758
rs41019
rs2459530
rs12409045
rs61789364
All − MR Egger
All − Inverse variance weighted
−0.2
−0.1
0.0
0.1
MR effect size for
'exposure' on 'outcome'

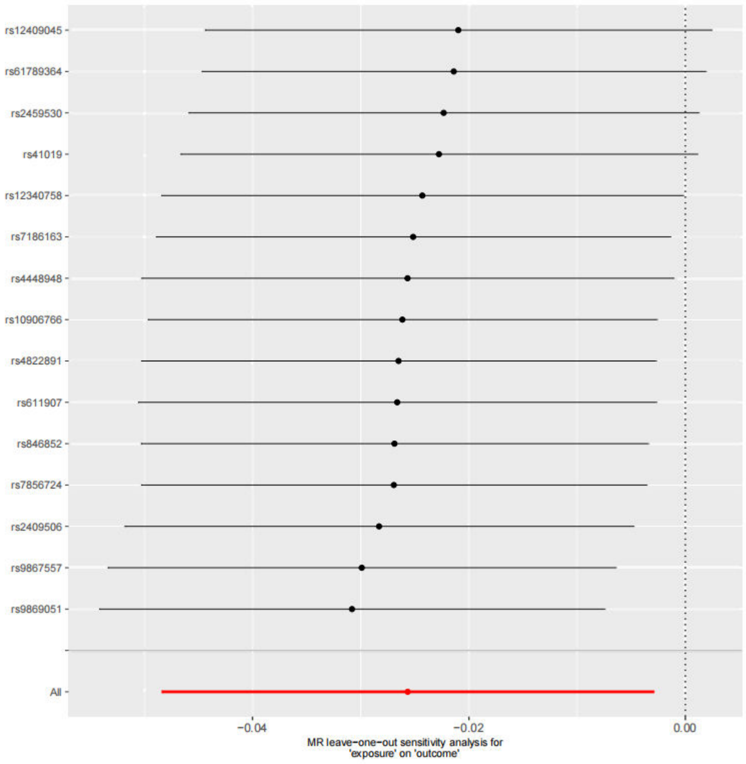
rs12409045
rs61789364
rs2459530
rs41019
rs12340758
rs7186163
rs4448948
rs10906766
rs4822891
rs611907
rs846852
rs7856724
rs2409506
rs9867557
rs9869051
All
−0.04
−0.02
0.00
MR leave−one−out sensitivity analysis for
'exposure' on 'outcome'

GCST90257077

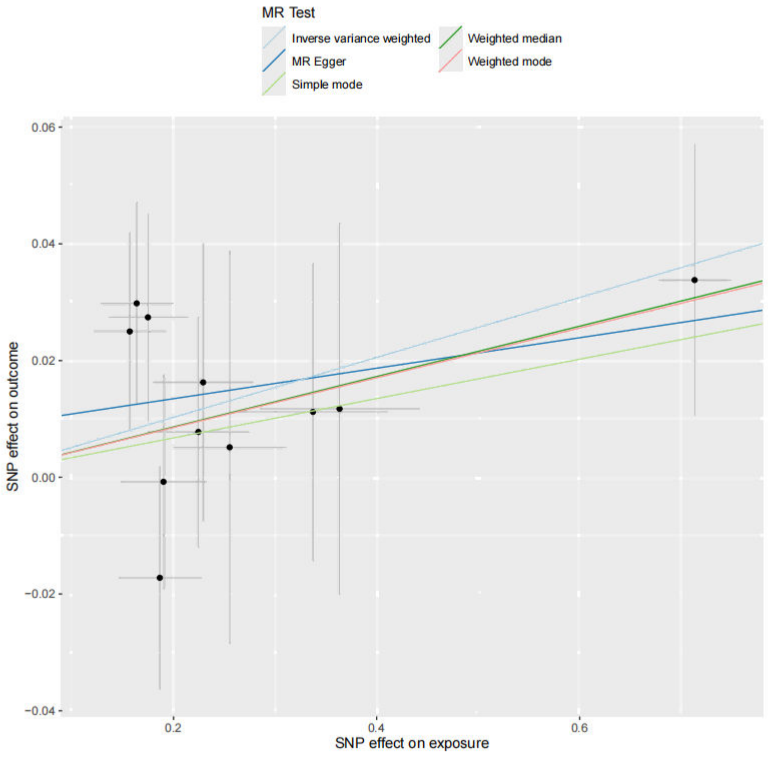

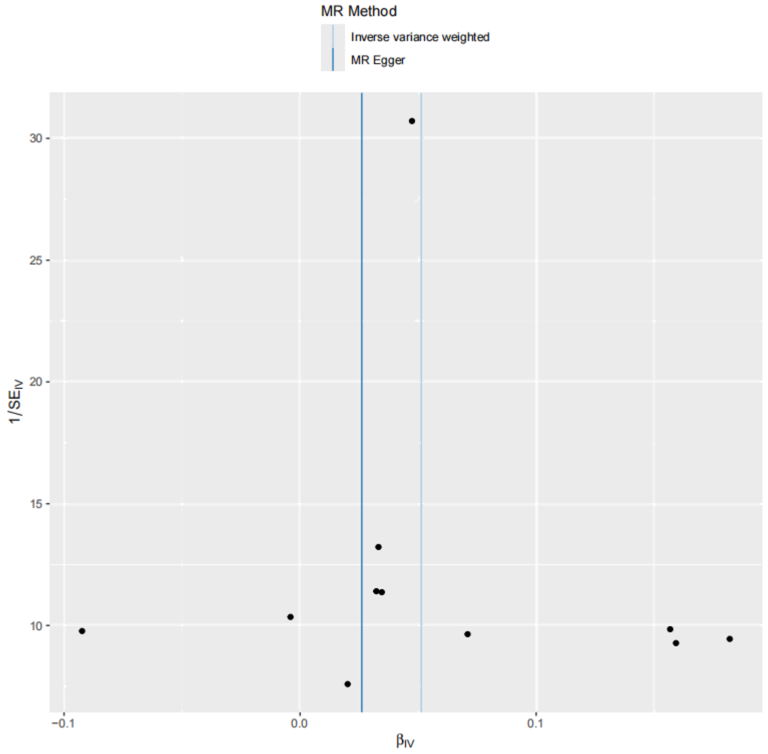

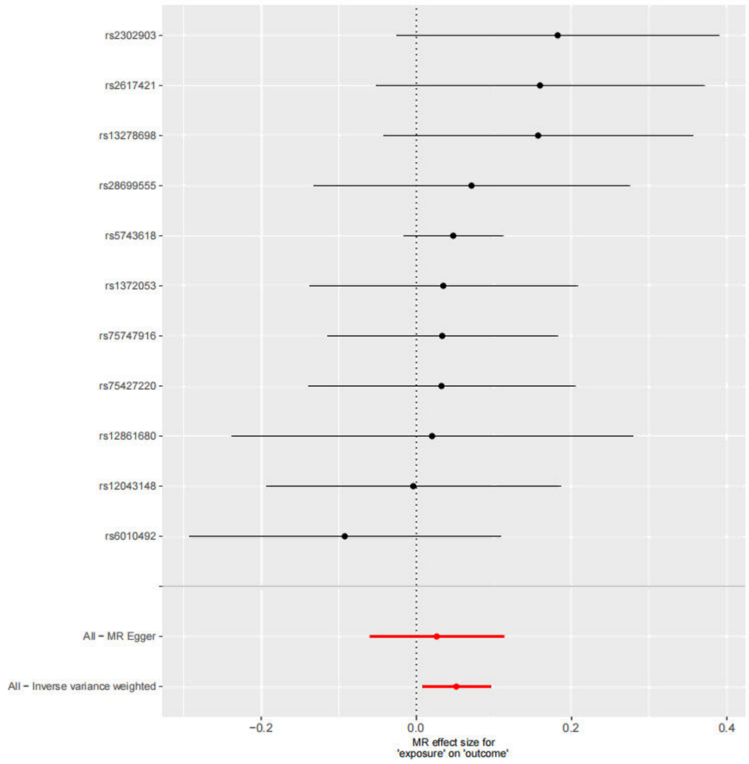

rs2302903
rs2617421
rs13278698
rs28699555
rs5743618
rs1372053
rs75747916
rs75427220
rs12861680
rs12043148
rs6010492
All − MR Egger
All − Inverse variance weighted
−0.2
0.0
0.2
0.4
MR effect size for
'exposure' on 'outcome'

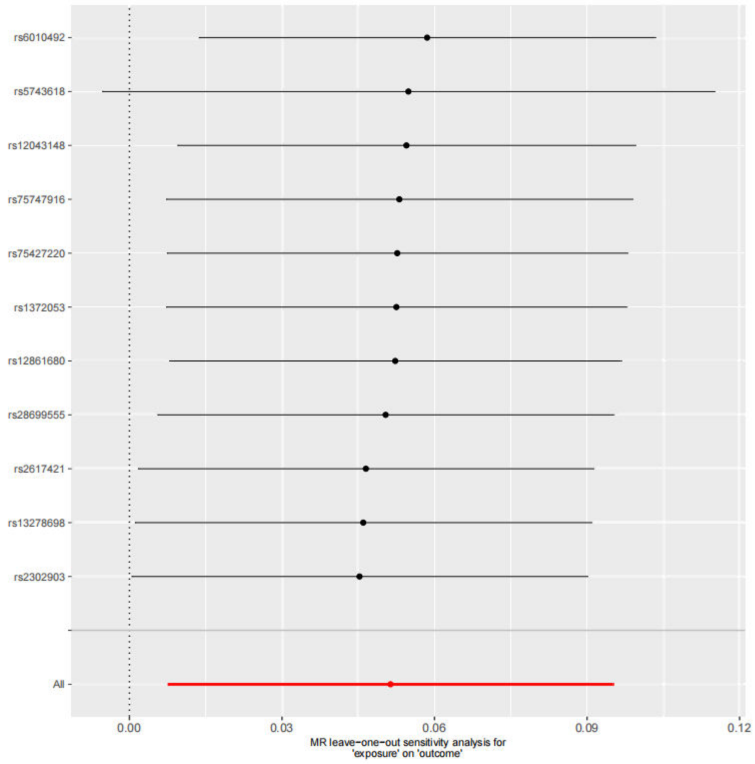

rs6010492
rs5743618
rs12043148
rs75747916
rs75427220
rs1372053
rs12861680
rs28699555
rs2617421
rs13278698
rs2302903
All
0.00
0.03
0.06
0.09
0.12
MR leave−one−out sensitivity analysis for
'exposure' on 'outcome'

GCST90257095

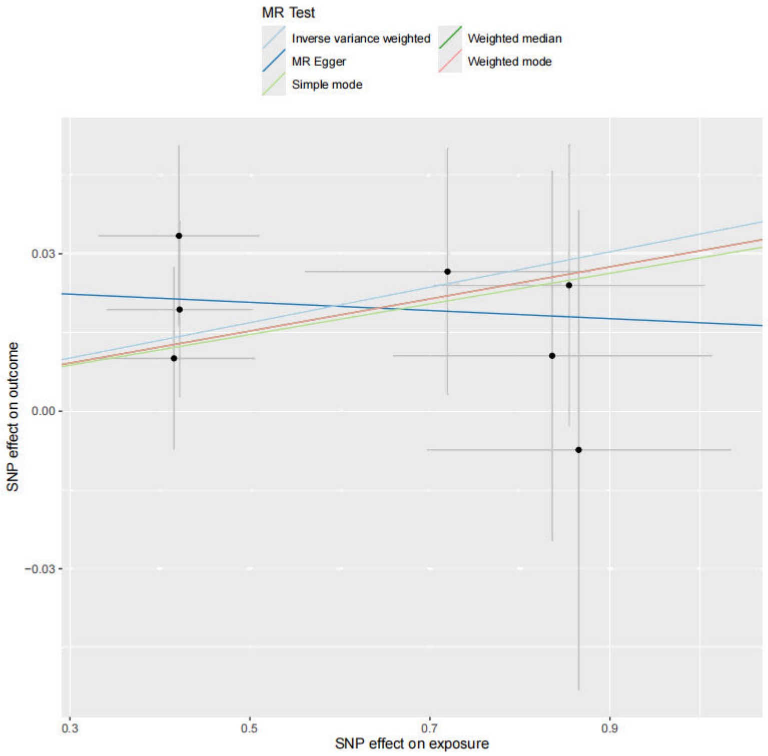

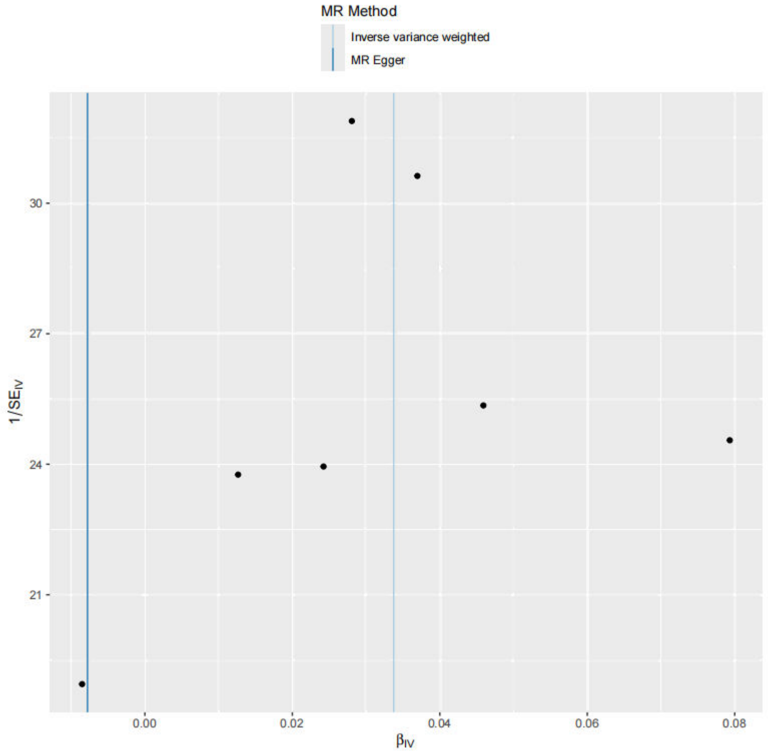

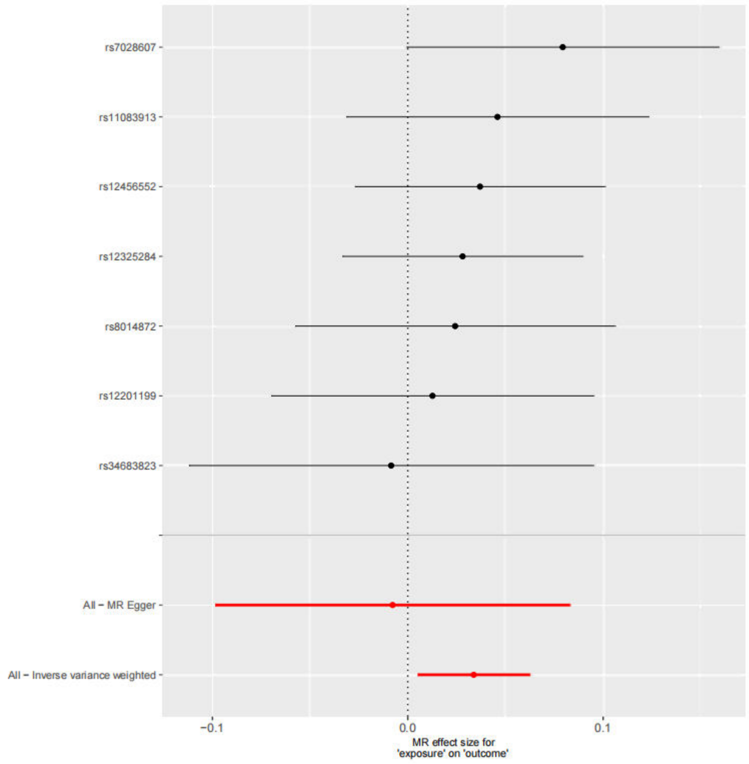

rs7028607
rs11083913
rs12456552
rs12325284
rs8014872
rs12201199
rs34683823
All − MR Egger
All − Inverse variance weighted
−0.1
0.0
0.1
MR effect size for
'exposure' on 'outcome'

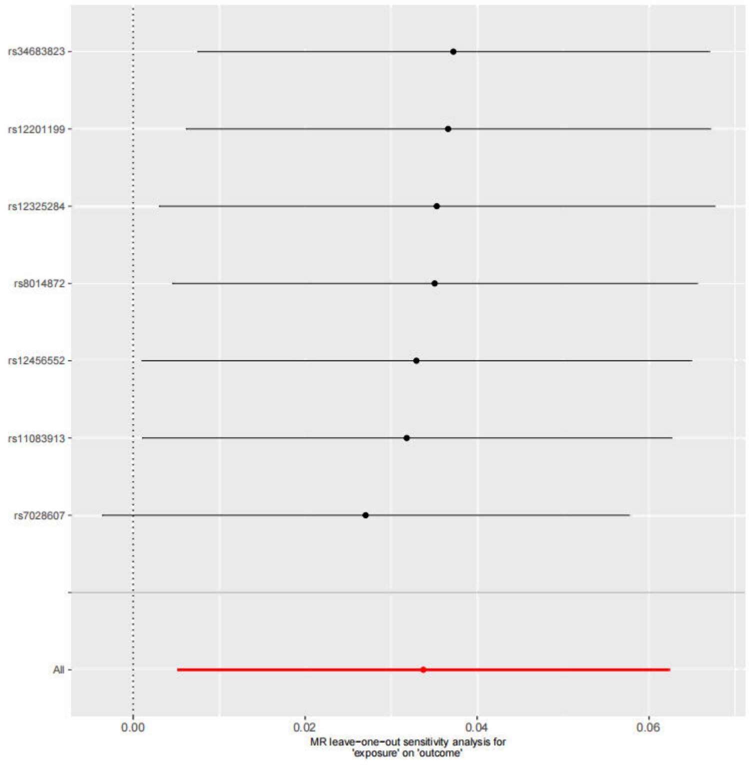

rs34683823
rs12201199
rs12325284
rs8014872
rs12456552
rs11083913
rs7028607
All
0.00
0.02
0.04
0.06
MR leave−one−out sensitivity analysis for
'exposure' on 'outcome'

GCST90257017

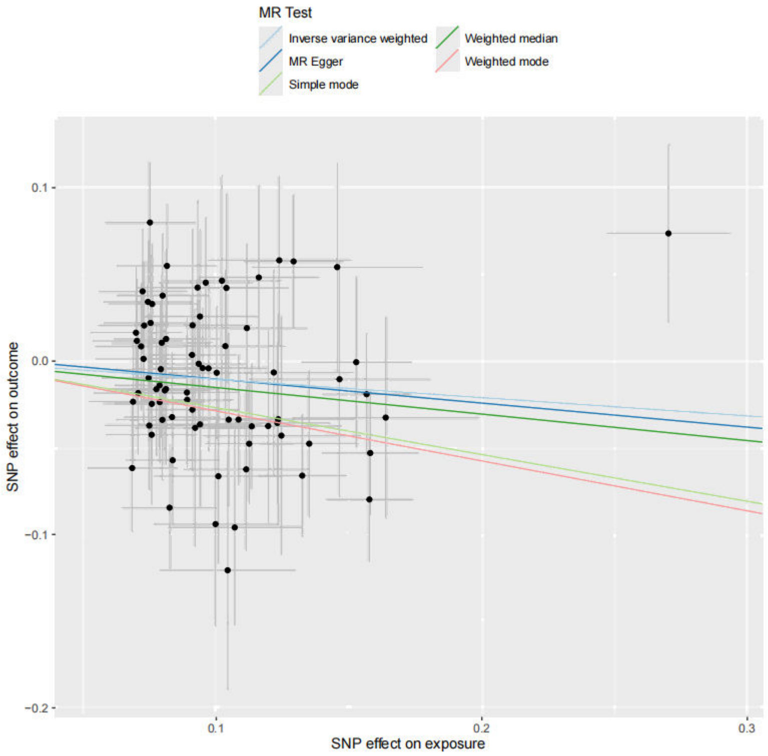

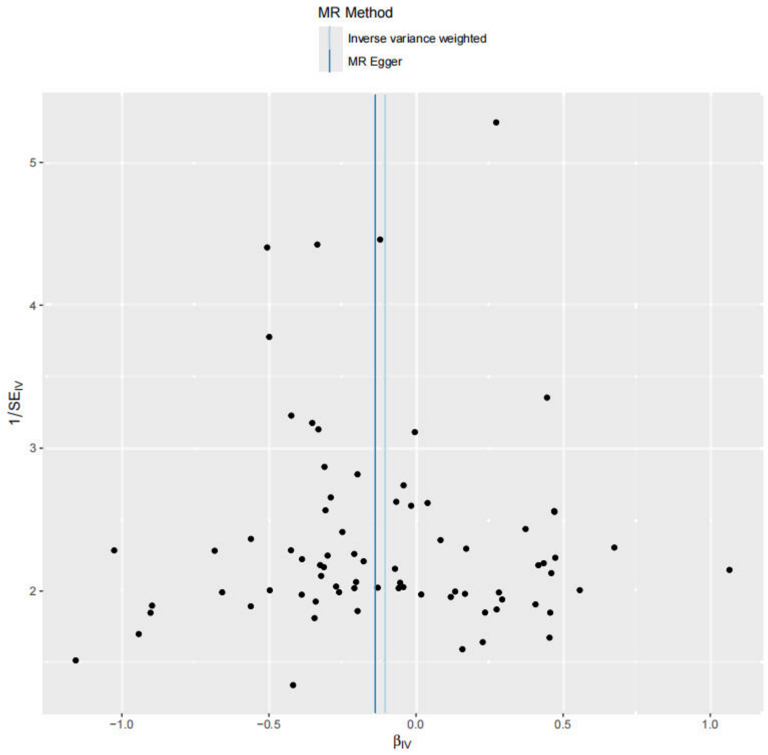

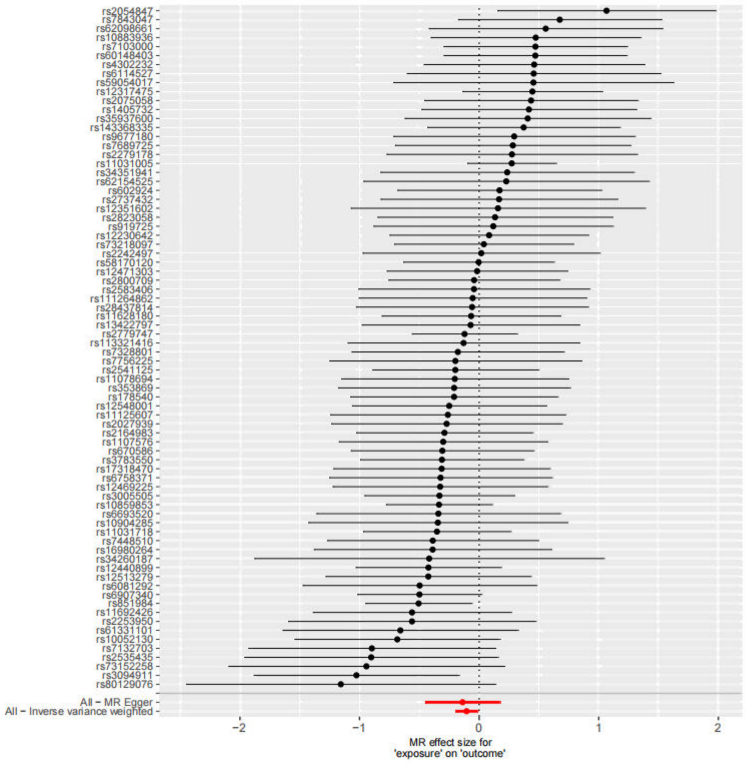
rs2054847
rs7843047
rs62098661
rs10883936
rs7103000
rs60148403
rs4302232
rs6114527
rs59054017
rs12317475
rs2075058
rs1405732
rs35937600
rs143368335
rs9677180
rs7689725
rs2279178
rs11031005
rs34351941
rs62154525
rs602924
rs2737432
rs12351602
rs2823058
rs919725
rs12230642
rs73218097
rs2242497
rs58170120
rs12471303
rs2800709
rs2583406
rs111264862
rs28437814
rs11628180
rs13422797
rs2779747
rs113321416
rs7328801
rs7756225
rs2541125
rs11078694
rs353869
rs178540
rs12548001
rs11125607
rs2027939
rs2164983
rs1107576
rs670586
rs3783550
rs17318470
rs6758371
rs12469225
rs3005505
rs10859853
rs6693520
rs10904285
rs11031718
rs7448510
rs16980264
rs34260187
rs12440899
rs12513279
rs6081292
rs6907340
rs851984
rs11692426
rs2253950
rs61331101
rs10052130
rs7132703
rs2535435
rs73152258
rs3094911
rs80129076
All − MR Egger
All − Inverse variance weighted
−2
−1
0
1
2
MR effect size for
'exposure' on 'outcome'

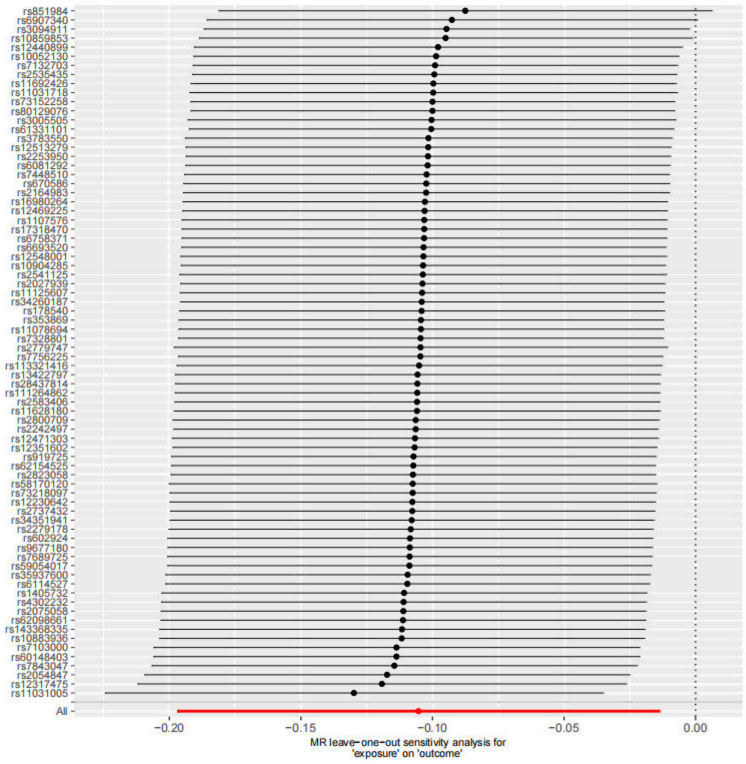
rs851984
rs6907340
rs3094911
rs10859853
rs12440899
rs10052130
rs7132703
rs2535435
rs11692426
rs11031718
rs73152258
rs80129076
rs3005505
rs61331101
rs3783550
rs12513279
rs2253950
rs6081292
rs7448510
rs670586
rs2164983
rs16980264
rs12469225
rs1107576
rs17318470
rs6758371
rs6693520
rs12548001
rs10904285
rs2541125
rs2027939
rs11125607
rs34260187
rs178540
rs353869
rs11078694
rs7328801
rs2779747
rs7756225
rs113321416
rs13422797
rs28437814
rs111264862
rs2583406
rs11628180
rs2800709
rs2242497
rs12471303
rs12351602
rs919725
rs62154525
rs2823058
rs58170120
rs73218097
rs12230642
rs2737432
rs34351941
rs2279178
rs602924
rs9677180
rs7689725
rs59054017
rs35937600
rs6114527
rs1405732
rs4302232
rs2075058
rs62098661
rs143368335
rs10883936
rs7103000
rs60148403
rs7843047
rs2054847
rs12317475
rs11031005
All
−0.20
−0.15
−0.10
−0.05
0.00
MR leave−one−out sensitivity analysis for
'exposure' on 'outcome'

GCST90257019

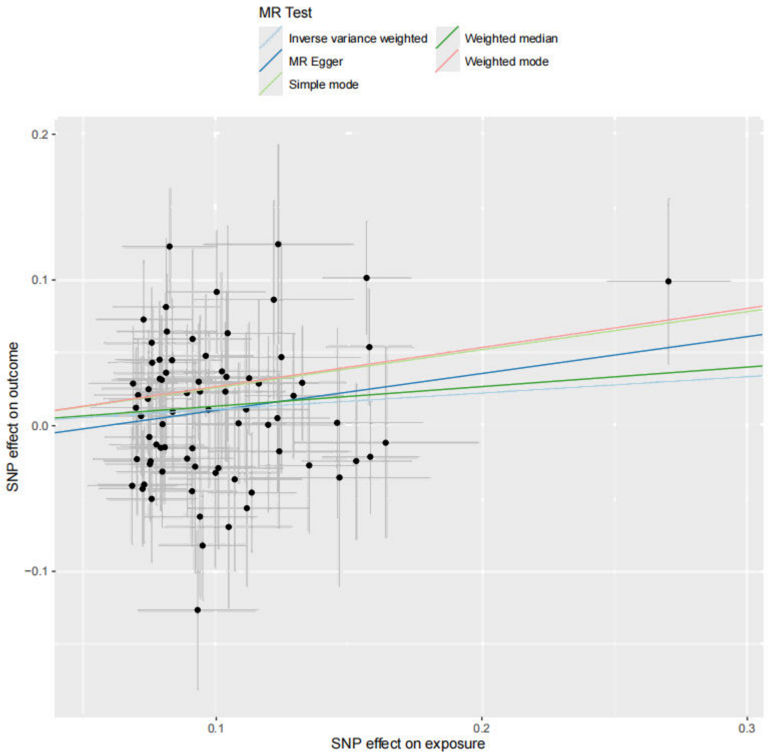

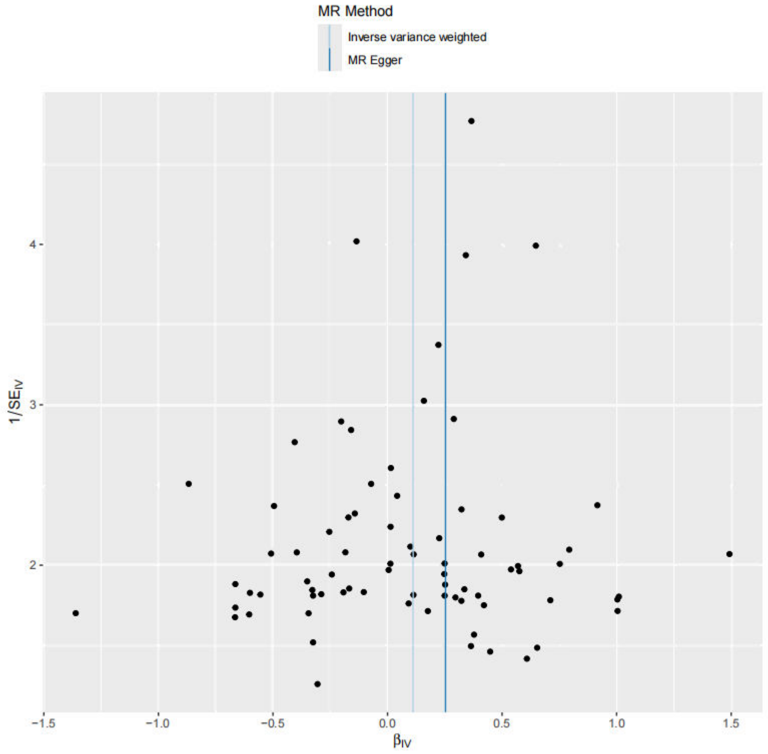

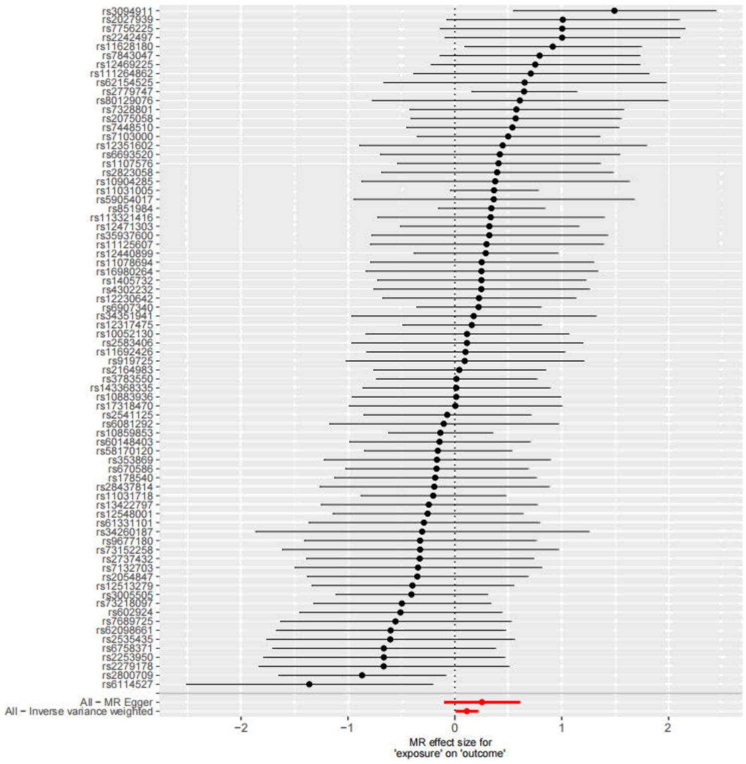
All − MR Egger
All − Inverse variance weighted
−2
−1
0
1
2
MR effect size for
'exposure' on 'outcome'

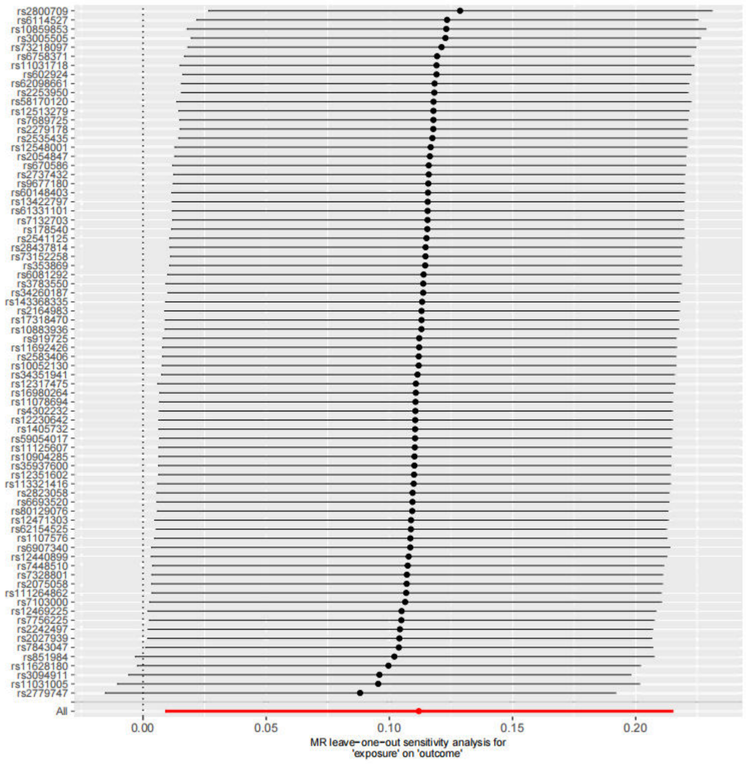
All
0.00
0.05
0.10
0.15
0.20
MR leave−one−out sensitivity analysis for
'exposure' on 'outcome'

GCST90257022

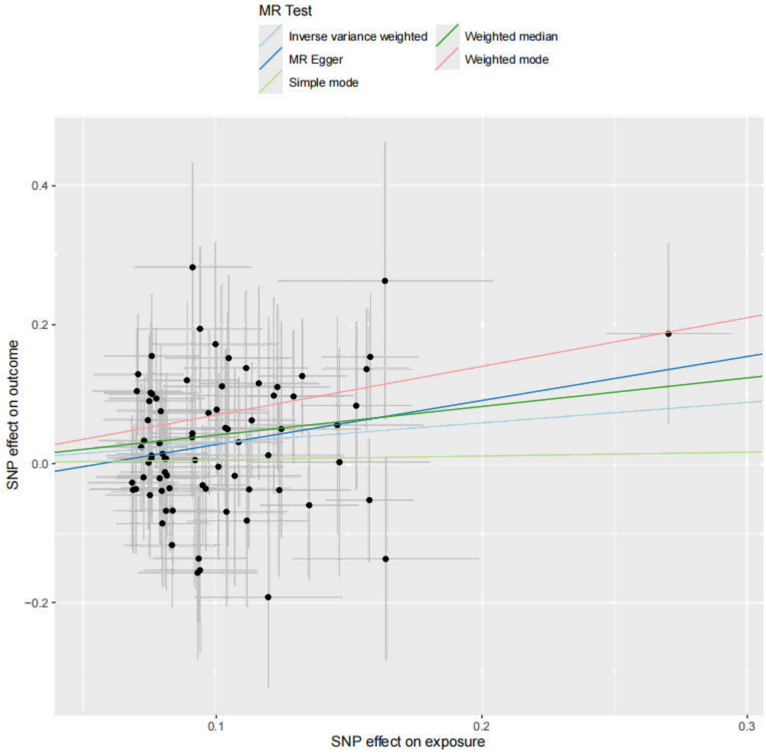

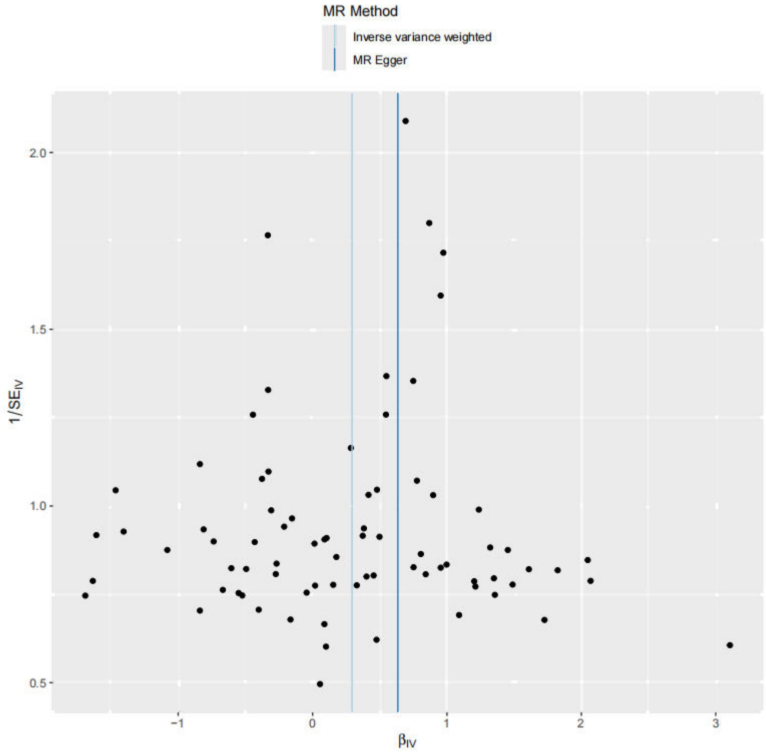

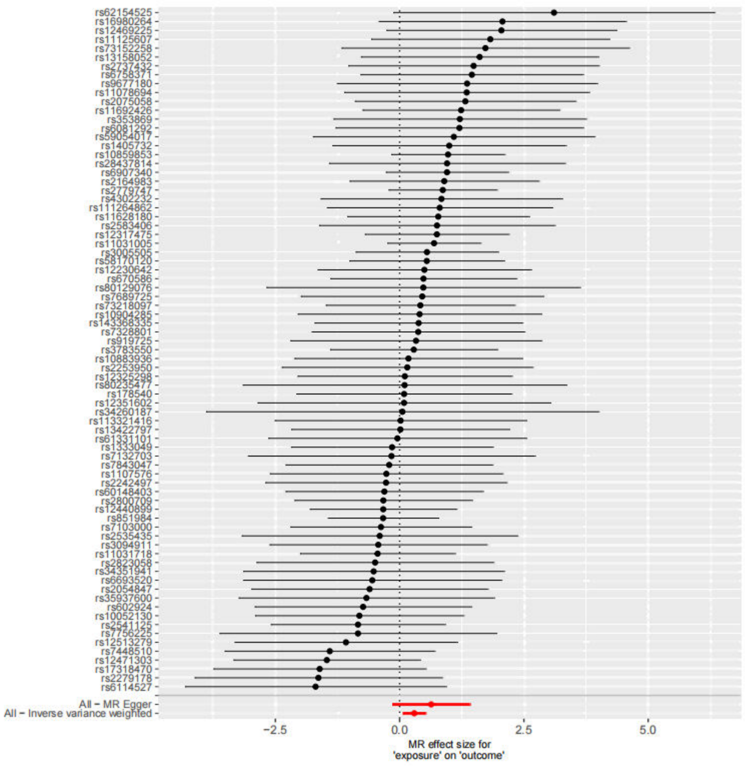

All − MR Egger
All − Inverse variance weighted
−2.5
0.0
2.5
5.0
MR effect size for
'exposure' on 'outcome'

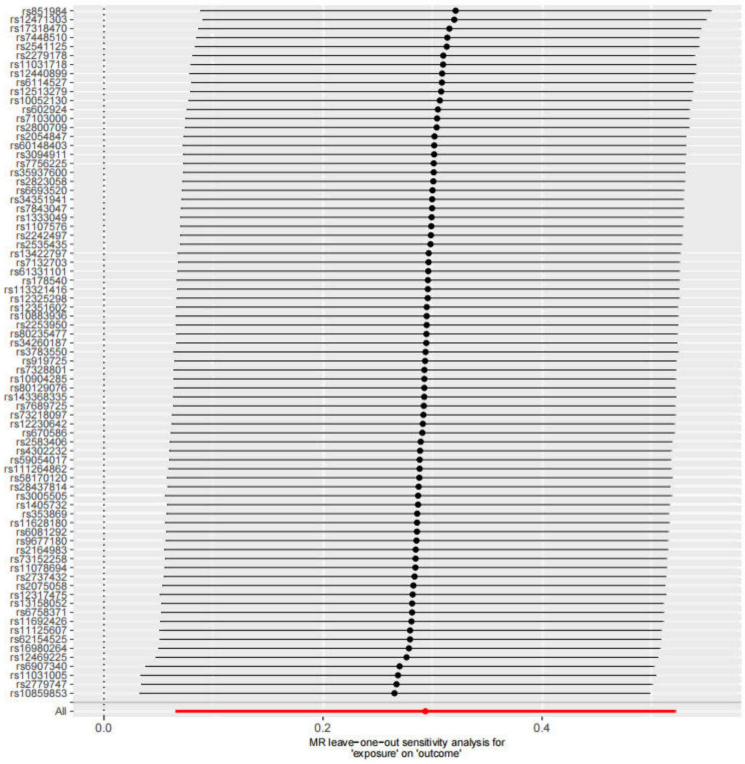

All
0.0
0.2
0.4
MR leave−one−out sensitivity analysis for
'exposure' on 'outcome'

GCST90257025

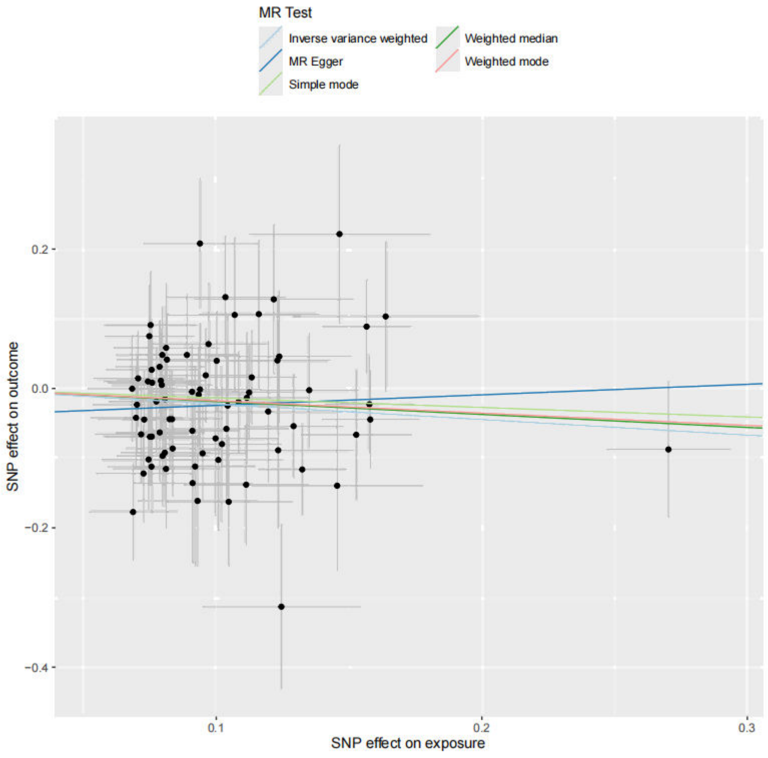

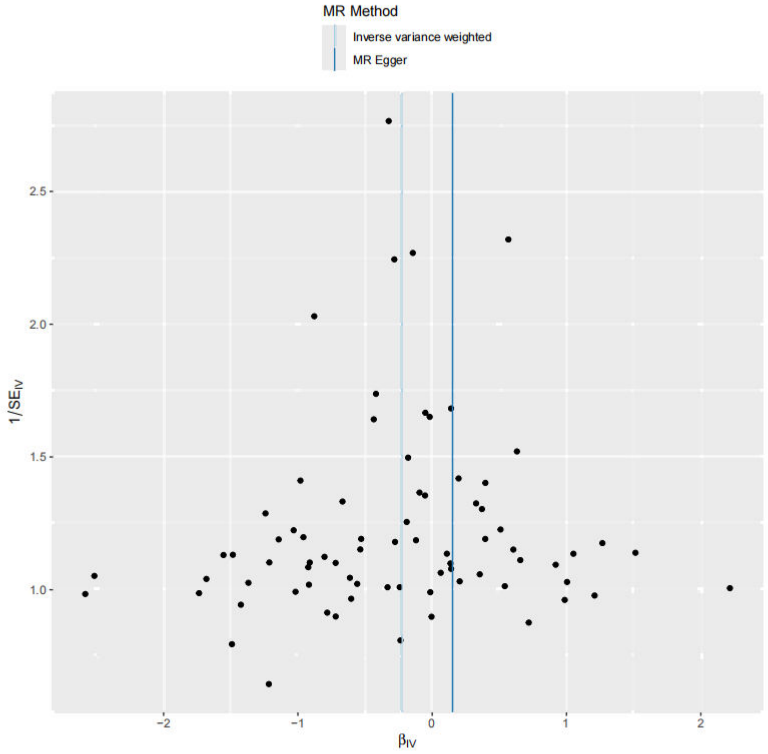

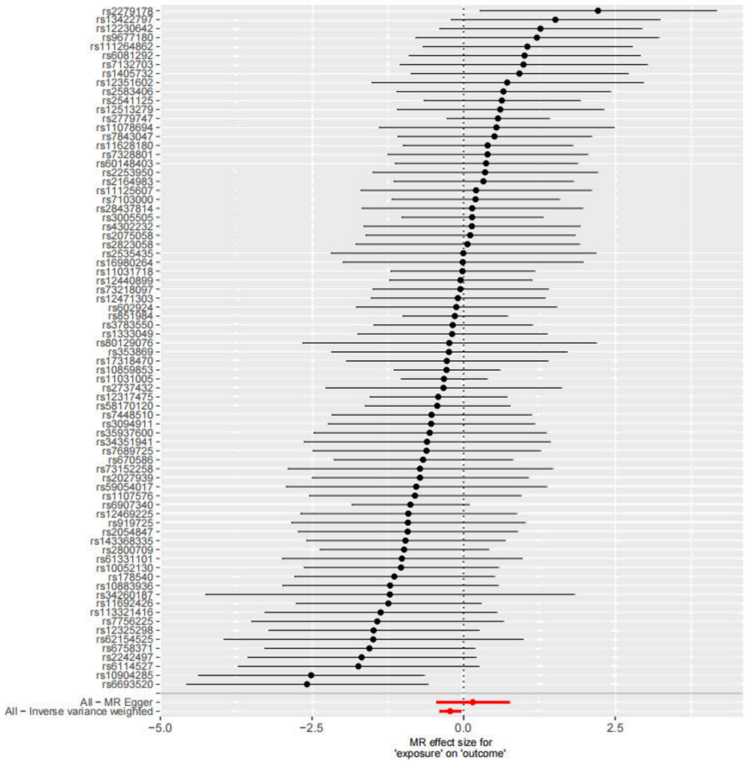
All − MR Egger
All − Inverse variance weighted
−5.0
−2.5
0.0
2.5
MR effect size for
'exposure' on 'outcome'

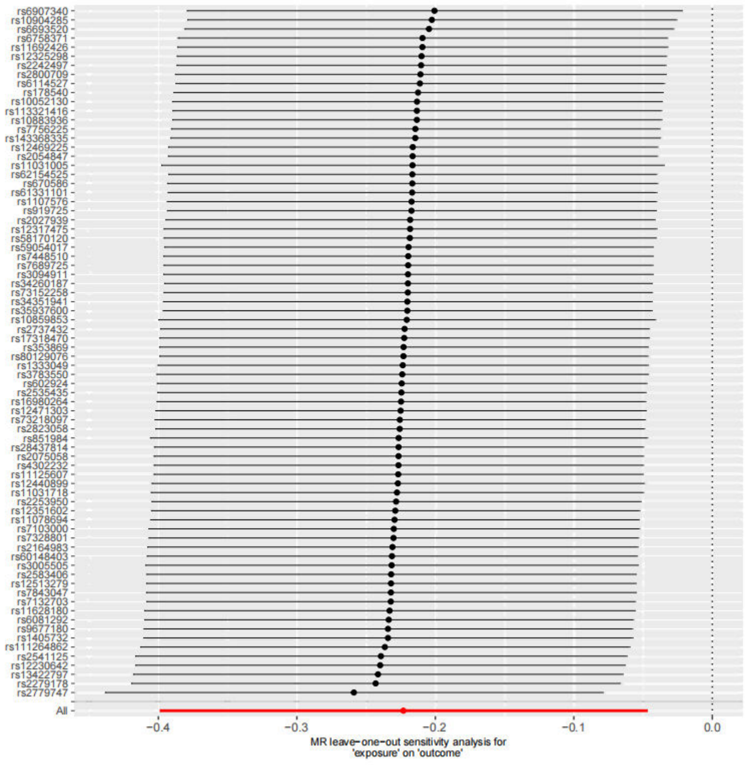
All
−0.4
−0.3
−0.2
−0.1
0.0
MR leave−one−out sensitivity analysis for
'exposure' on 'outcome'

GCST90257028

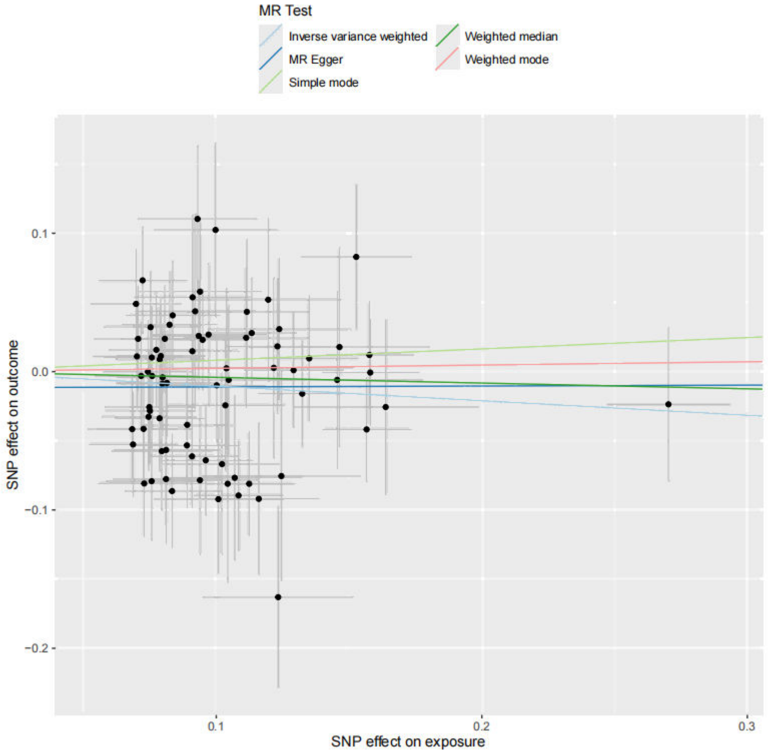

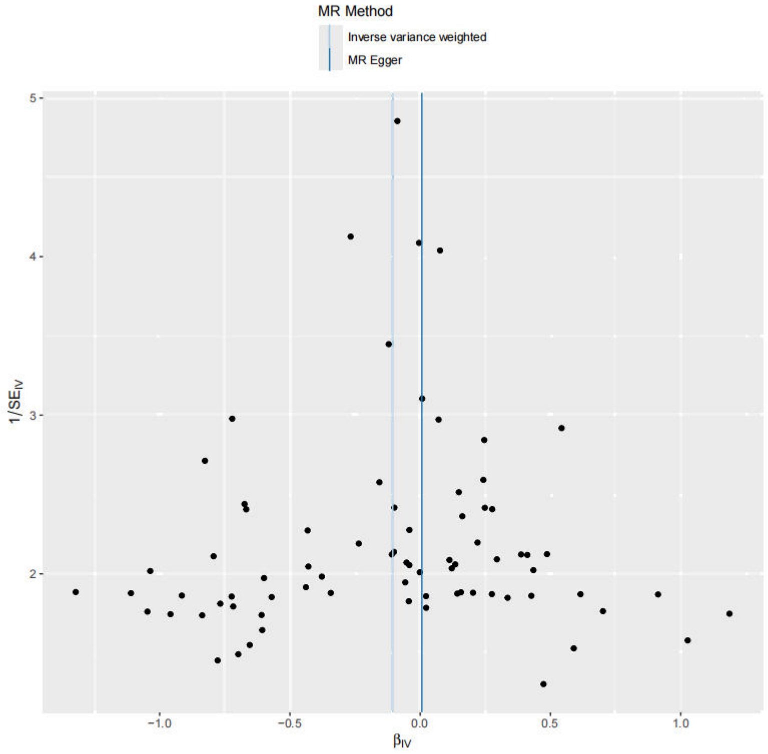

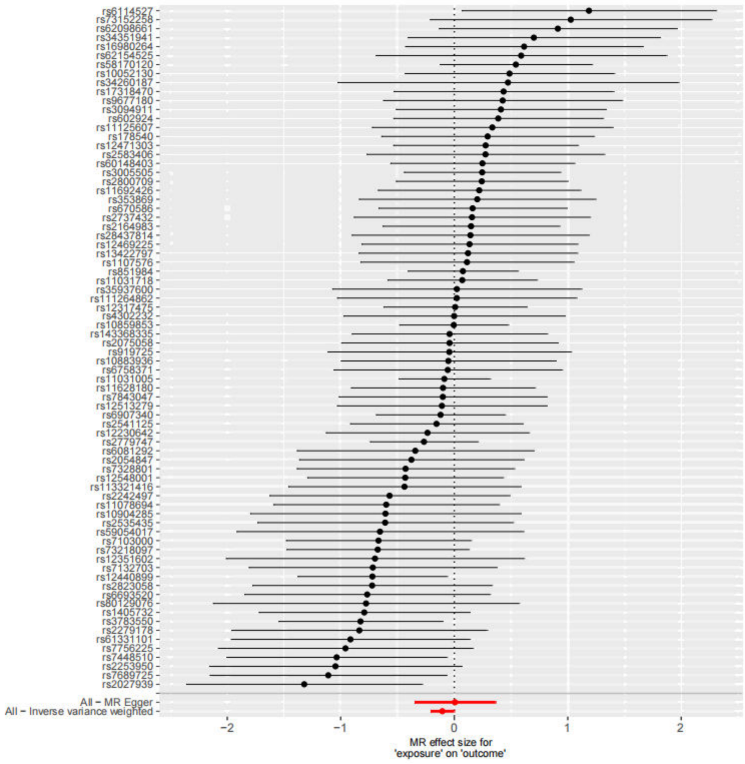
All − MR Egger
All − Inverse variance weighted
−2
−1
0
1
2
MR effect size for
'exposure' on 'outcome'

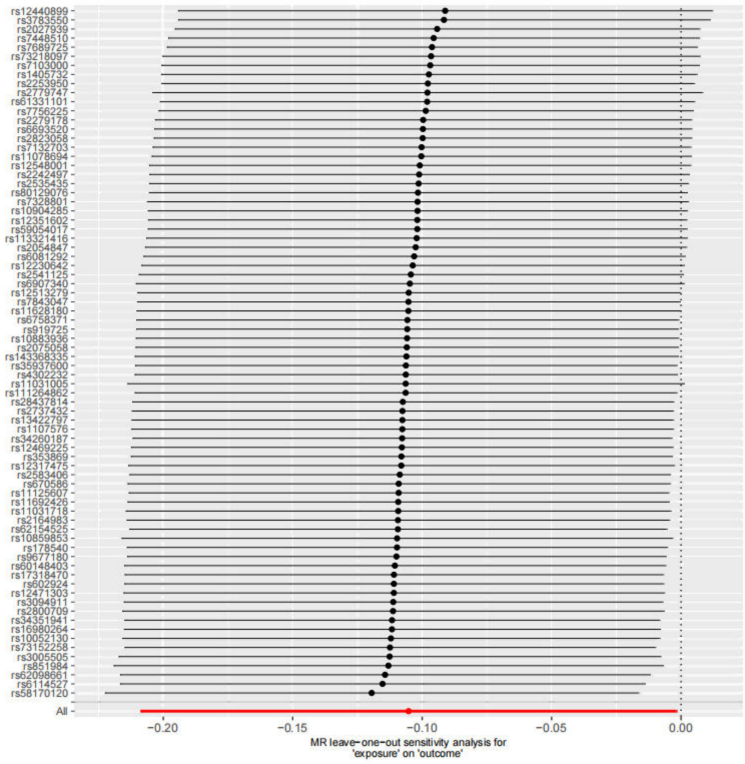
All
−0.20
−0.15
−0.10
−0.05
0.00
MR leave−one−out sensitivity analysis for
'exposure' on 'outcome'

GCST90257041

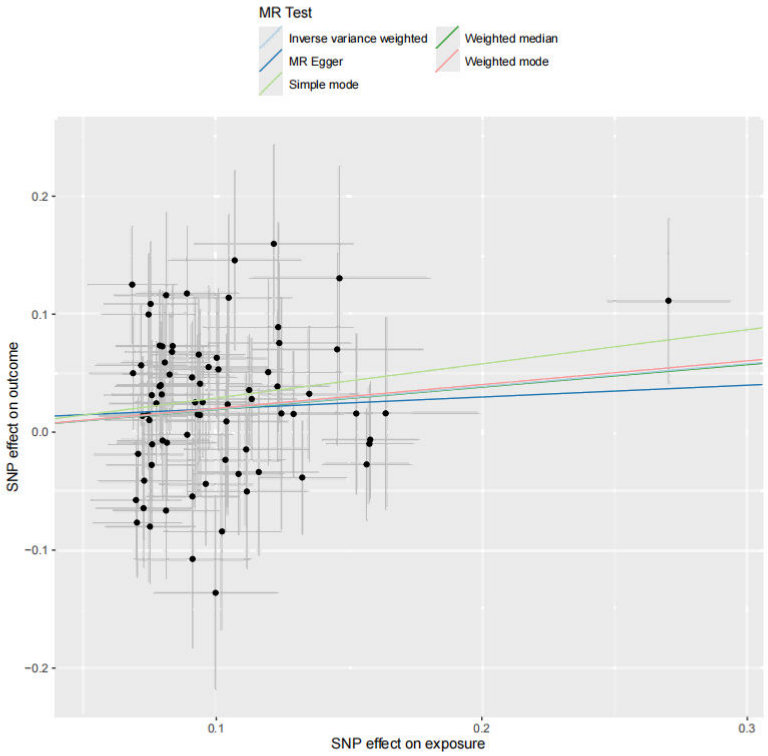

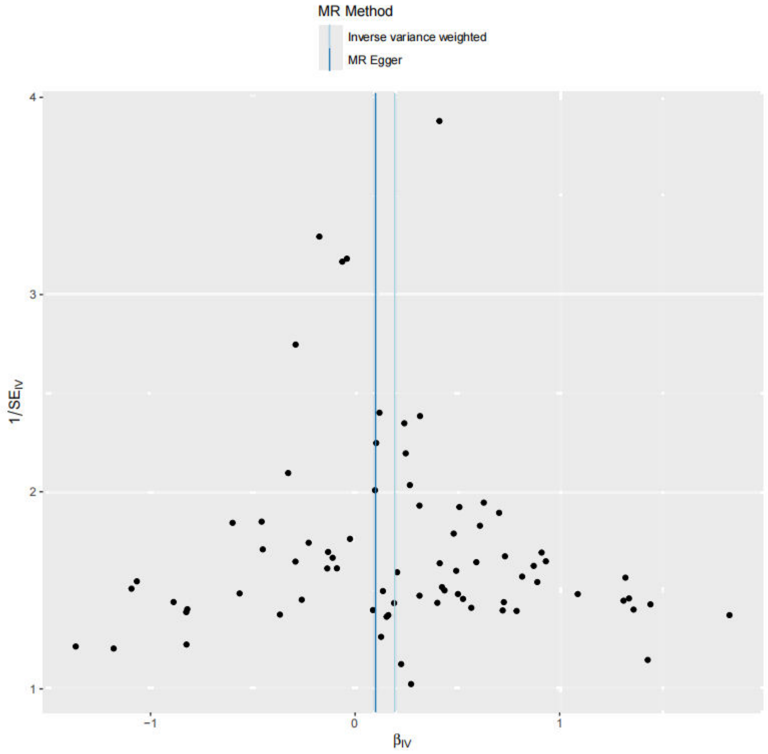

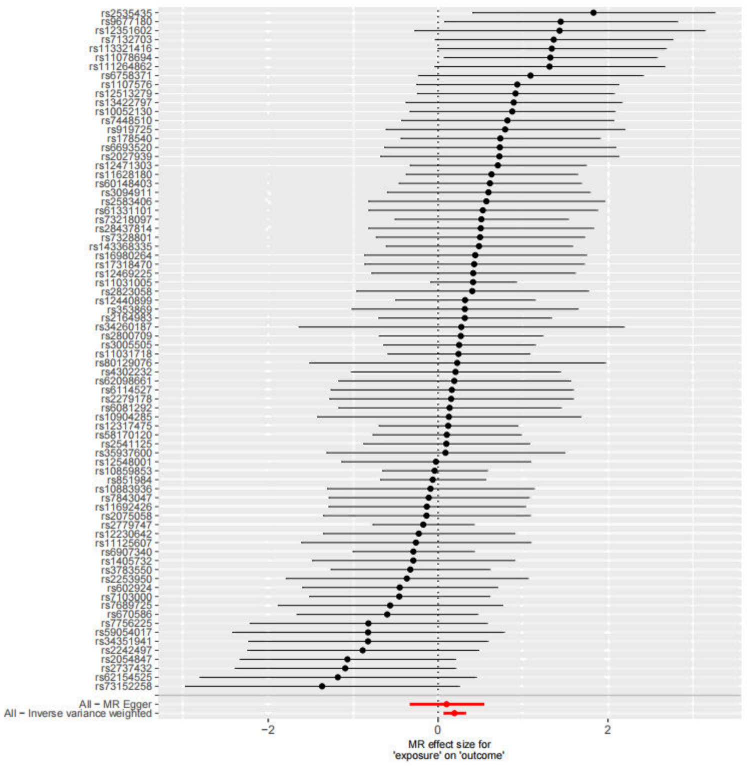
All - MR Egger
All - Inverse variance weighted
-2
0
2
MR effect size for
'exposure' on 'outcome'

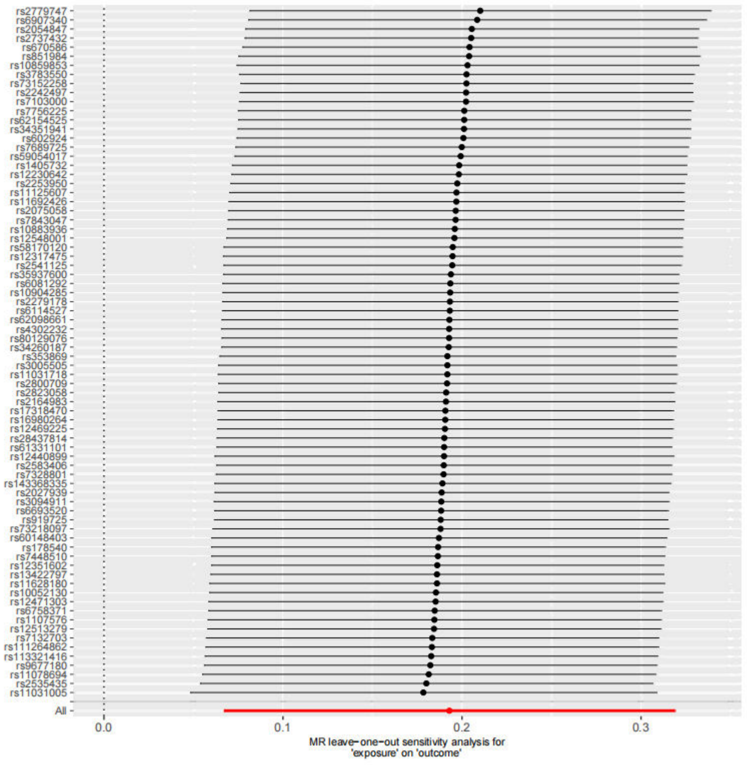
All
0.0
0.1
0.2
0.3
MR leave-one-out sensitivity analysis for
'exposure' on 'outcome'

GCST90257088

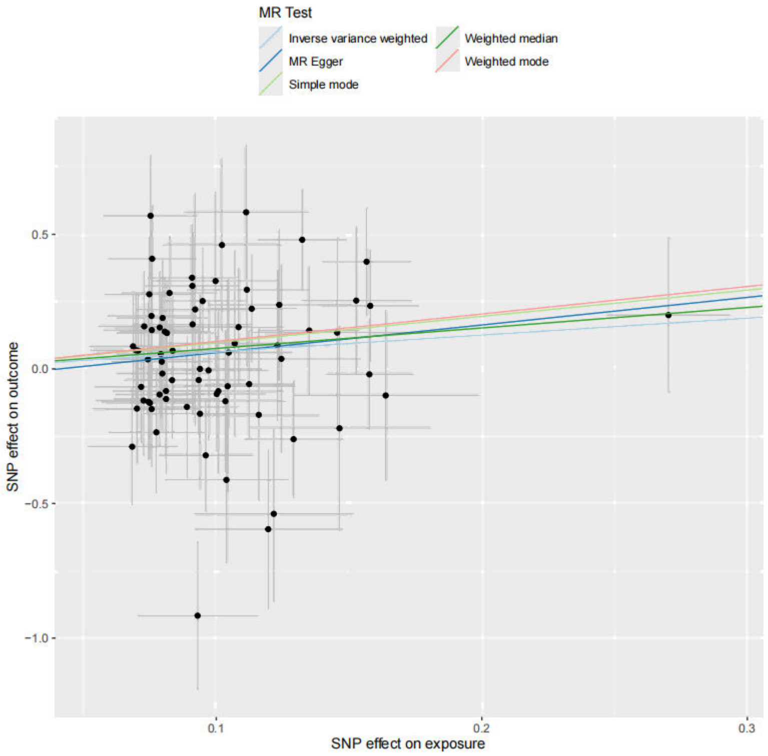

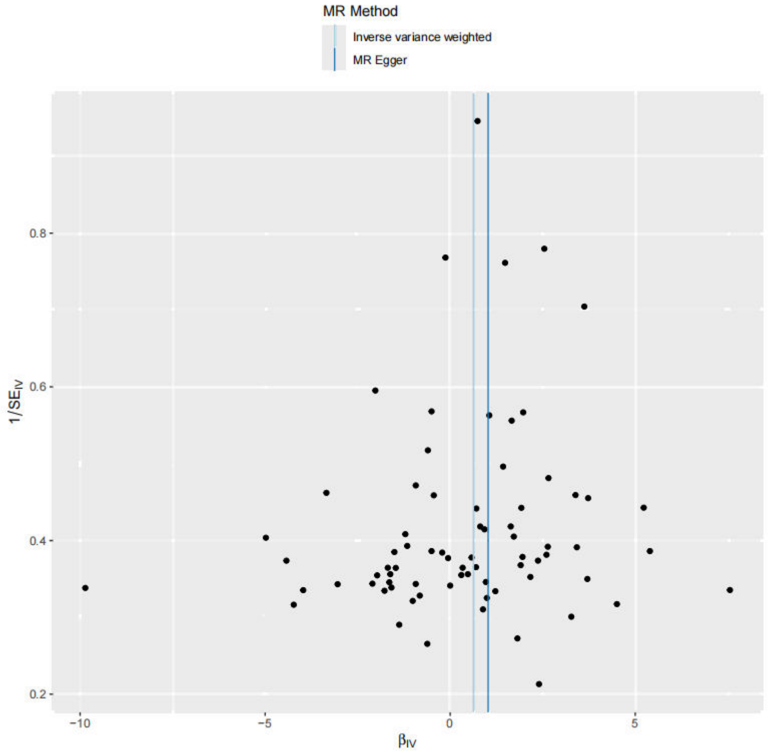

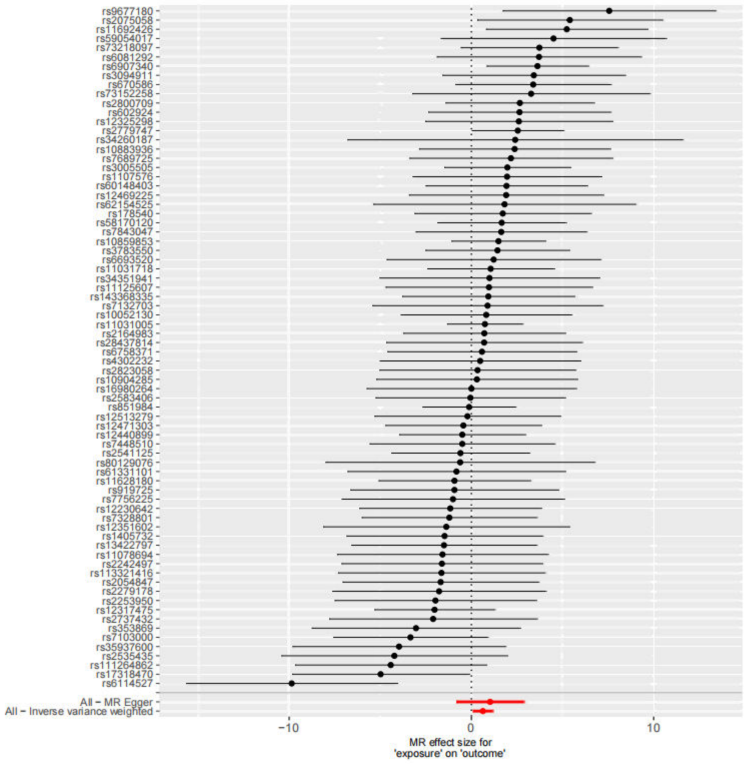
rs9677180
rs2075058
rs11692426
rs59054017
rs73218097
rs6081292
rs6907340
rs3094911
rs670586
rs73152258
rs2800709
rs602924
rs12325298
rs2779747
rs34260187
rs10883936
rs7689725
rs3005505
rs1107576
rs60148403
rs12469225
rs62154525
rs178540
rs58170120
rs7843047
rs10859853
rs3783550
rs6693520
rs11031718
rs34351941
rs11125607
rs143368335
rs7132703
rs10052130
rs11031005
rs2164983
rs28437814
rs6758371
rs4302232
rs2823058
rs10904285
rs16980264
rs2583406
rs851984
rs12513279
rs12471303
rs12440899
rs7448510
rs2541125
rs80129076
rs61331101
rs11628180
rs919725
rs7756225
rs12230642
rs7328801
rs12351602
rs1405732
rs13422797
rs11078694
rs2242497
rs113321416
rs2054847
rs2279178
rs2253950
rs12317475
rs2737432
rs353869
rs7103000
rs35937600
rs2535435
rs111264862
rs17318470
rs6114527
All − MR Egger
All − Inverse variance weighted
−10
0
10
MR effect size for
'exposure' on 'outcome'

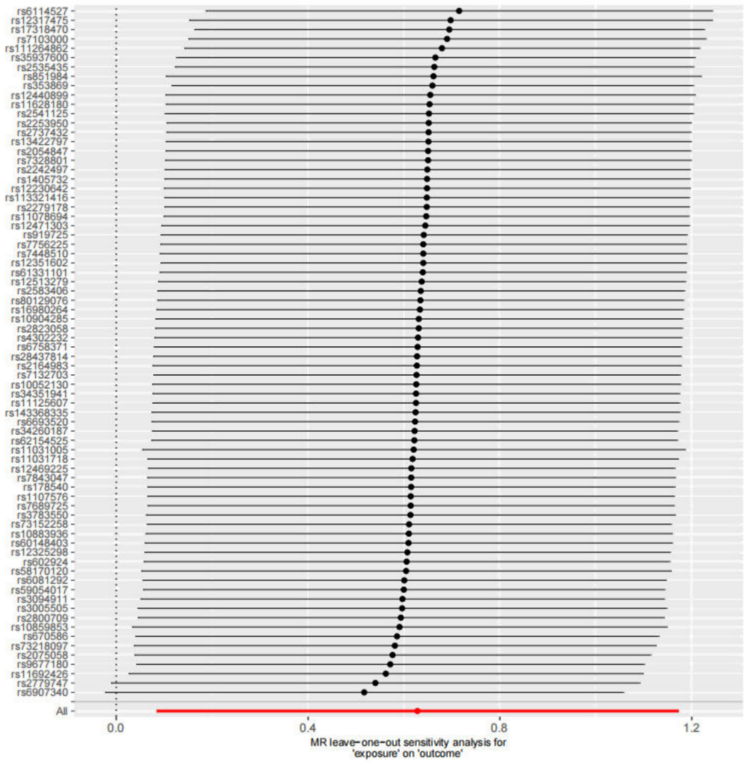
rs6114527
rs12317475
rs17318470
rs7103000
rs111264862
rs35937600
rs2535435
rs851984
rs353869
rs12440899
rs11628180
rs2541125
rs2253950
rs2737432
rs13422797
rs2054847
rs7328801
rs2242497
rs1405732
rs12230642
rs113321416
rs2279178
rs11078694
rs12471303
rs919725
rs7756225
rs7448510
rs12351602
rs61331101
rs12513279
rs2583406
rs80129076
rs16980264
rs10904285
rs2823058
rs4302232
rs6758371
rs28437814
rs2164983
rs7132703
rs10052130
rs34351941
rs11125607
rs143368335
rs6693520
rs34260187
rs62154525
rs11031005
rs11031718
rs12469225
rs7843047
rs178540
rs1107576
rs7689725
rs3783550
rs73152258
rs10883936
rs60148403
rs12325298
rs602924
rs58170120
rs6081292
rs59054017
rs3094911
rs3005505
rs2800709
rs10859853
rs670586
rs73218097
rs2075058
rs9677180
rs11692426
rs2779747
rs6907340
All
0.0
0.4
0.8
1.2
MR leave−one−out sensitivity analysis for
'exposure' on 'outcome'

GCST90257089

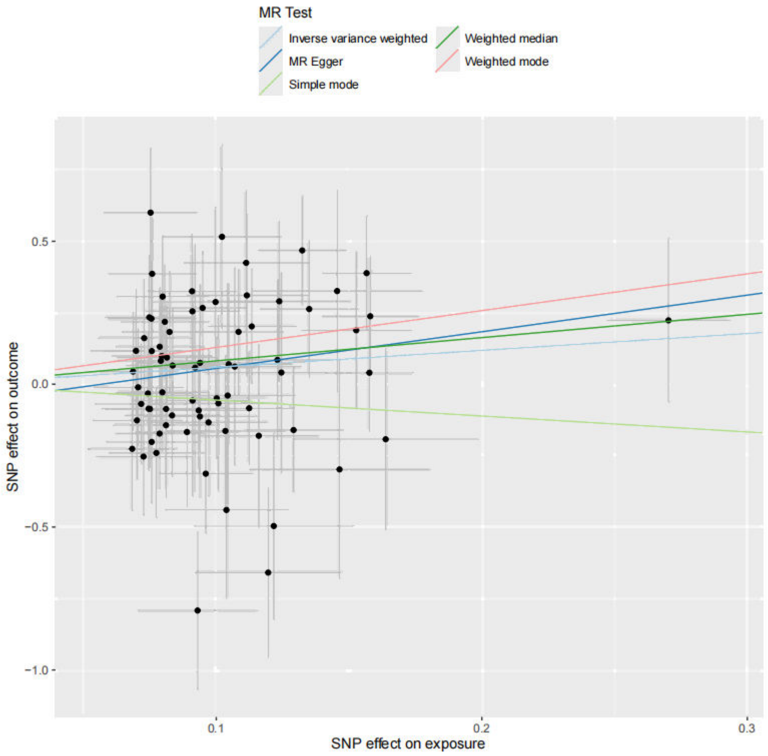

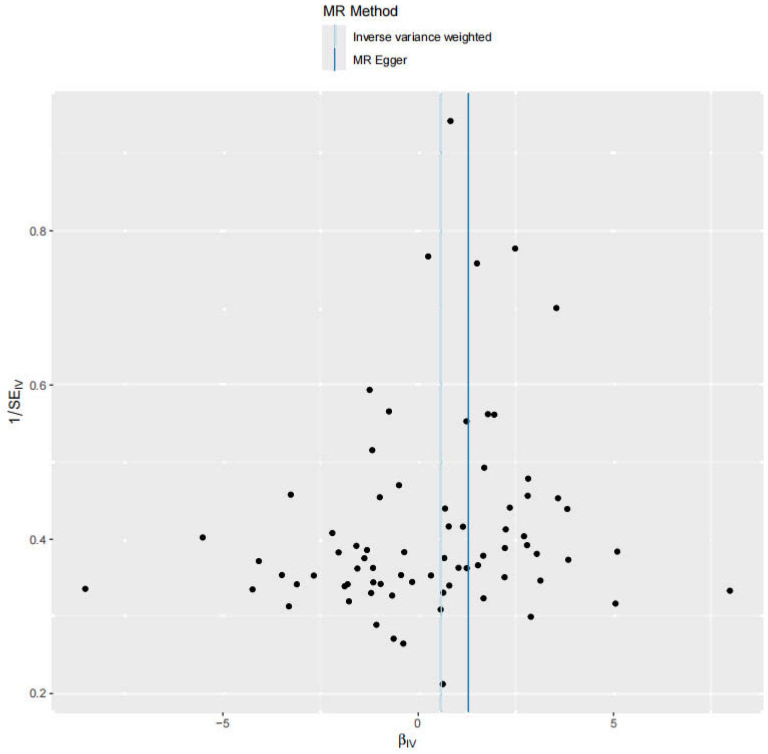

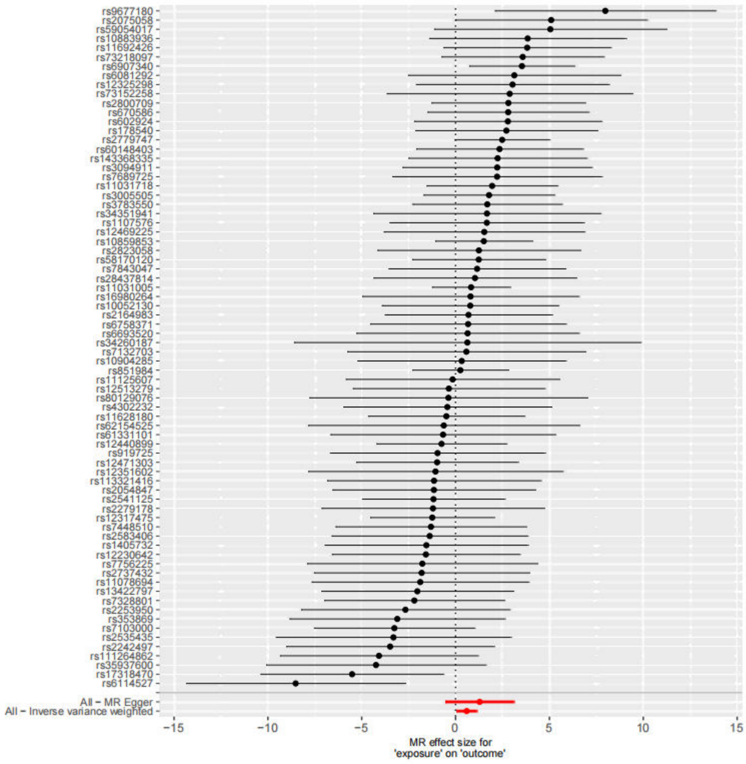
All − MR Egger
All − Inverse variance weighted
−15
−10
−5
0
5
10
15
MR effect size for
'exposure' on 'outcome'

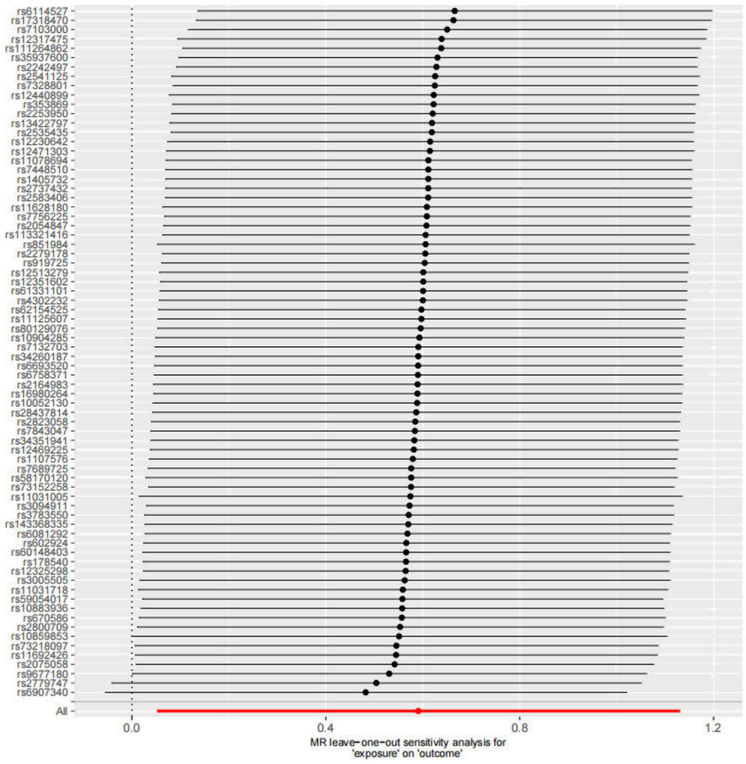
All
0.0
0.4
0.8
1.2
MR leave−one−out sensitivity analysis for
'exposure' on 'outcome'

GCST90257051

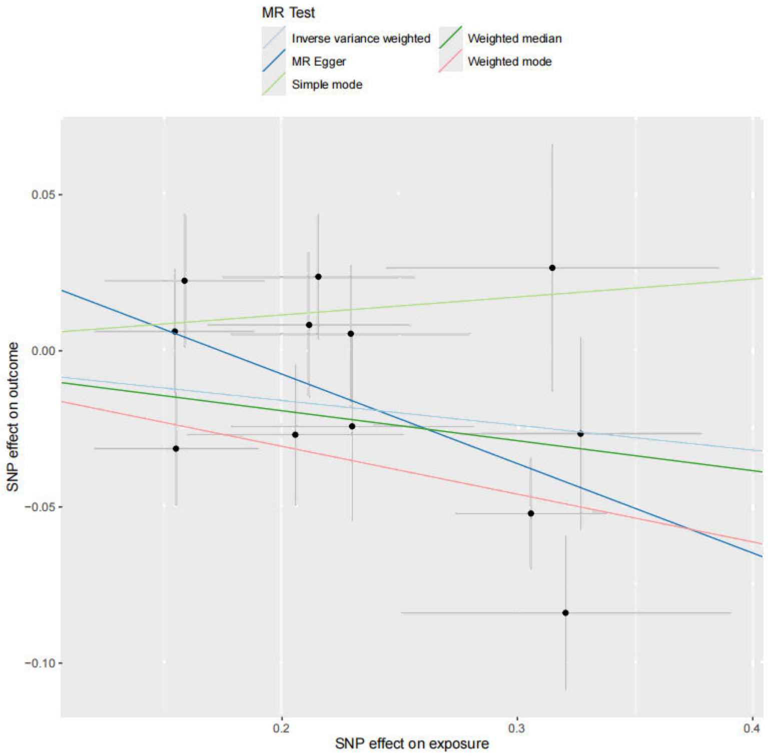

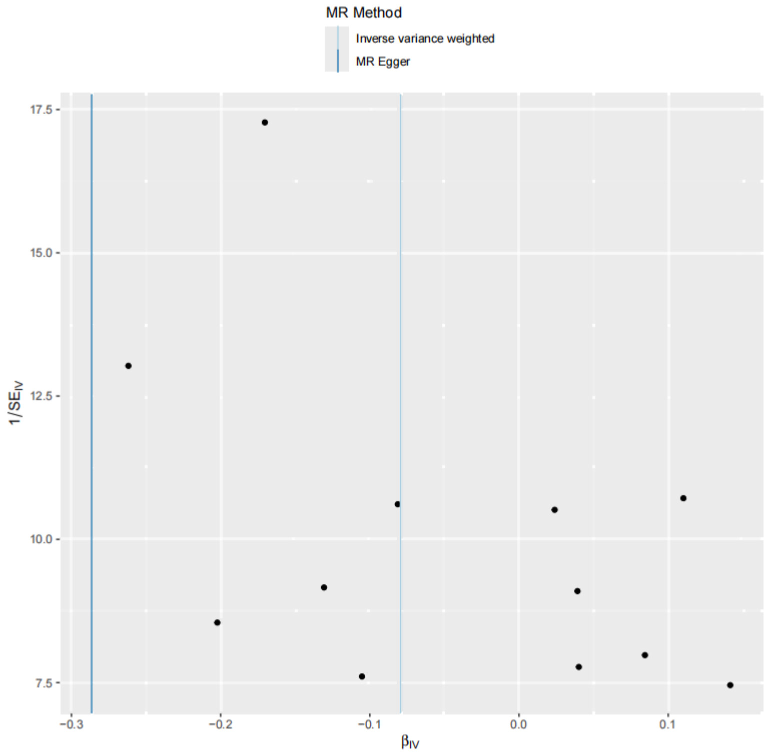

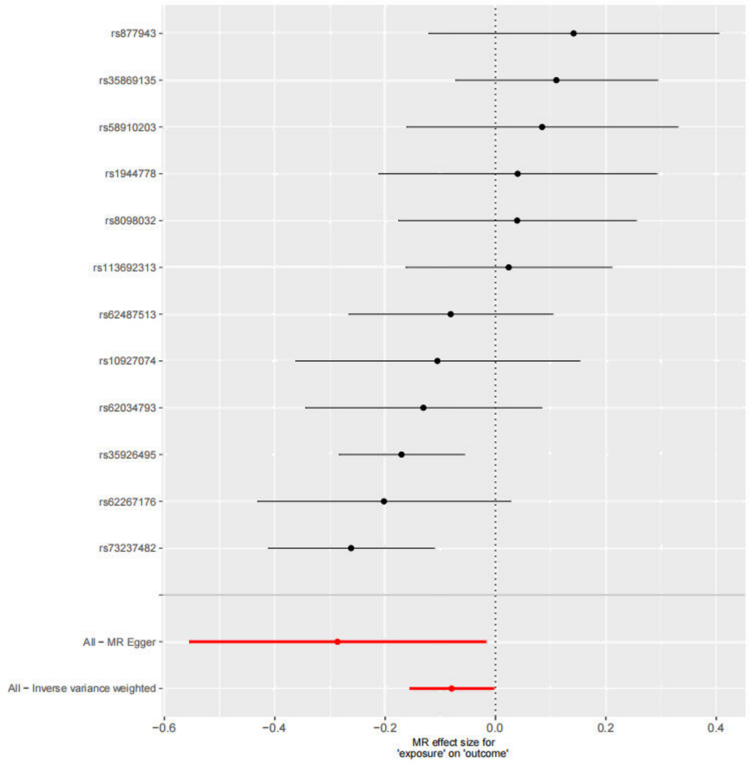

rs877943
rs35869135
rs58910203
rs1944778
rs8098032
rs113692313
rs62487513
rs10927074
rs62034793
rs35926495
rs62267176
rs73237482
All − MR Egger
All − Inverse variance weighted
−0.6
−0.4
−0.2
0.0
0.2
0.4
MR effect size for
'exposure' on 'outcome'

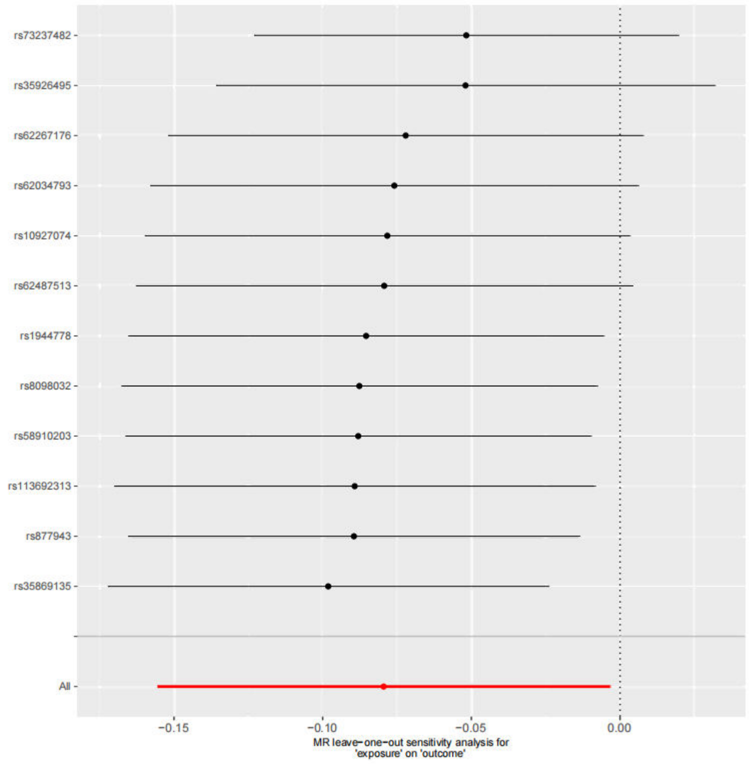

rs73237482
rs35926495
rs62267176
rs62034793
rs10927074
rs62487513
rs1944778
rs8098032
rs58910203
rs113692313
rs877943
rs35869135
All
−0.15
−0.10
−0.05
0.00
MR leave−one−out sensitivity analysis for
'exposure' on 'outcome'

GCST90257054

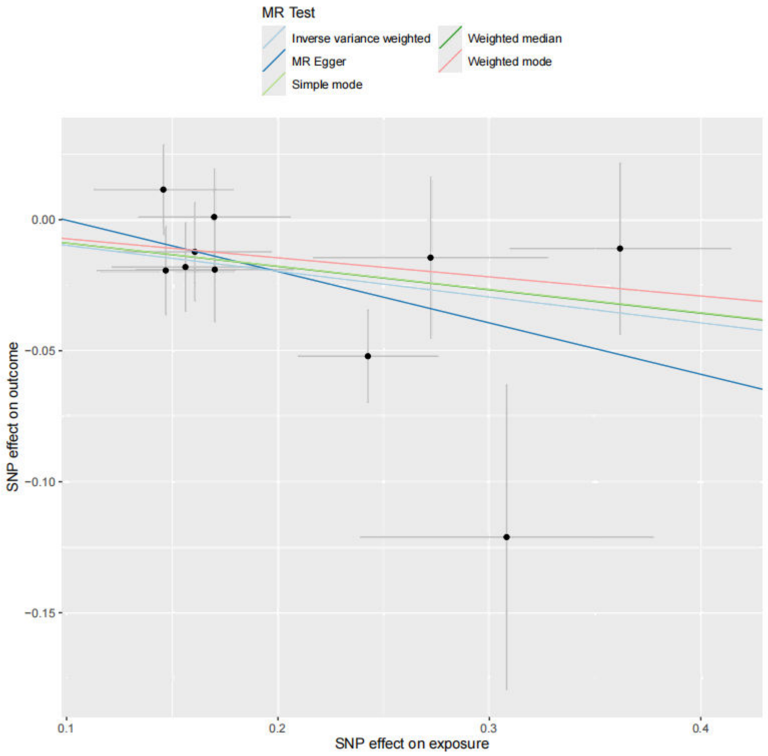

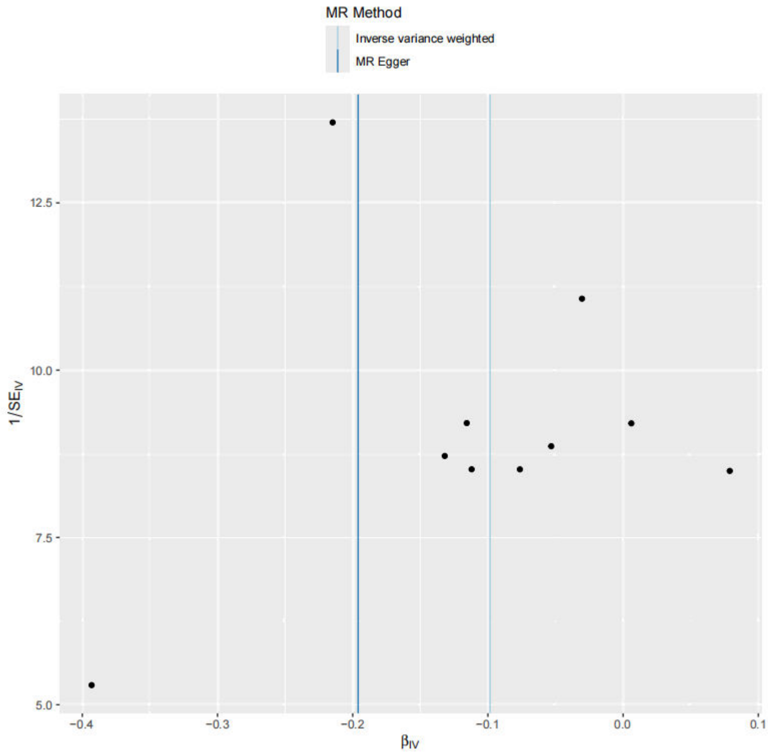

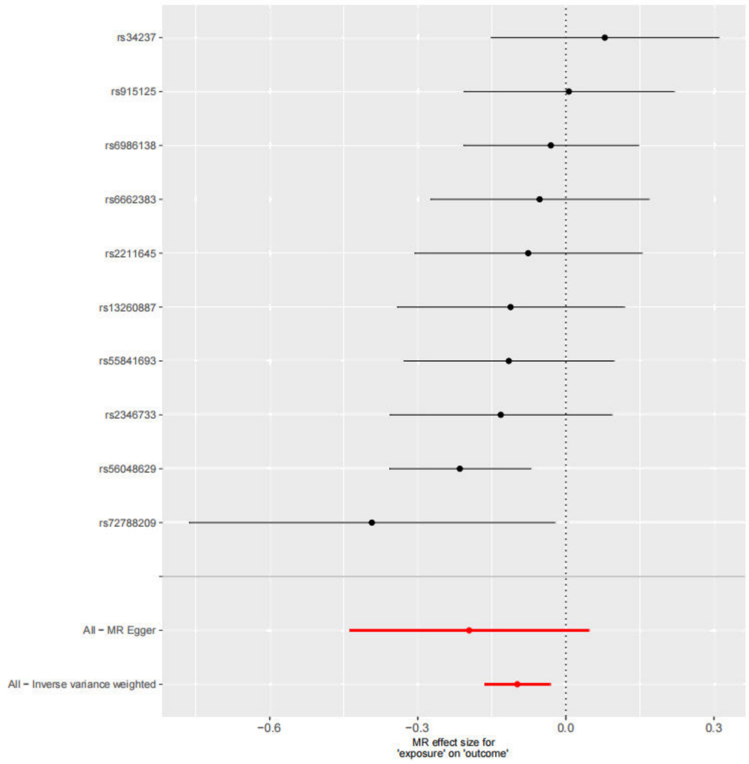
rs34237
rs915125
rs6986138
rs6662383
rs2211645
rs13260887
rs55841693
rs2346733
rs56048629
rs72788209
All − MR Egger
All − Inverse variance weighted
−0.6
−0.3
0.0
0.3
MR effect size for
'exposure' on 'outcome'

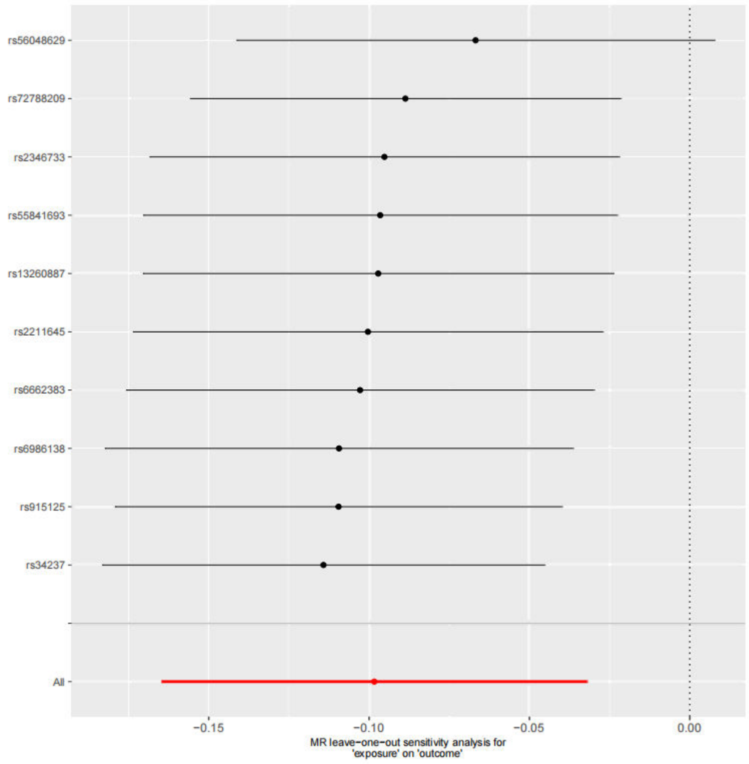
rs56048629
rs72788209
rs2346733
rs55841693
rs13260887
rs2211645
rs6662383
rs6986138
rs915125
rs34237
All
−0.15
−0.10
−0.05
0.00
MR leave−one−out sensitivity analysis for
'exposure' on 'outcome'

GCST90257092

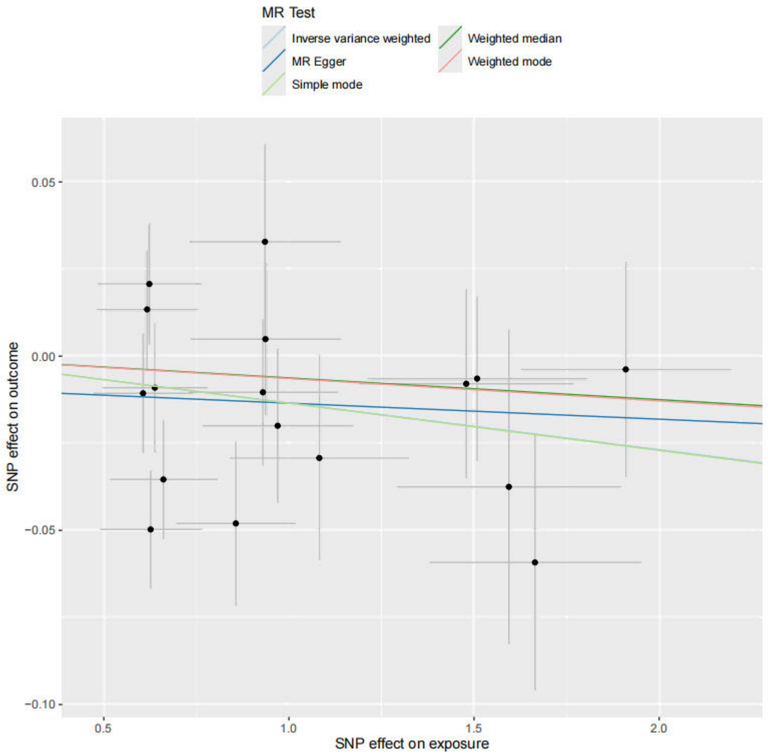

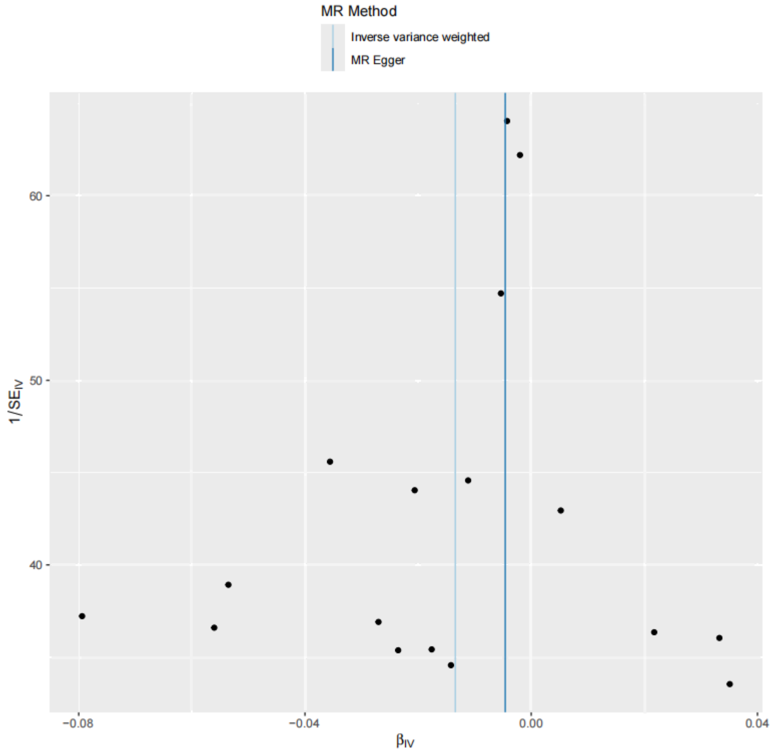

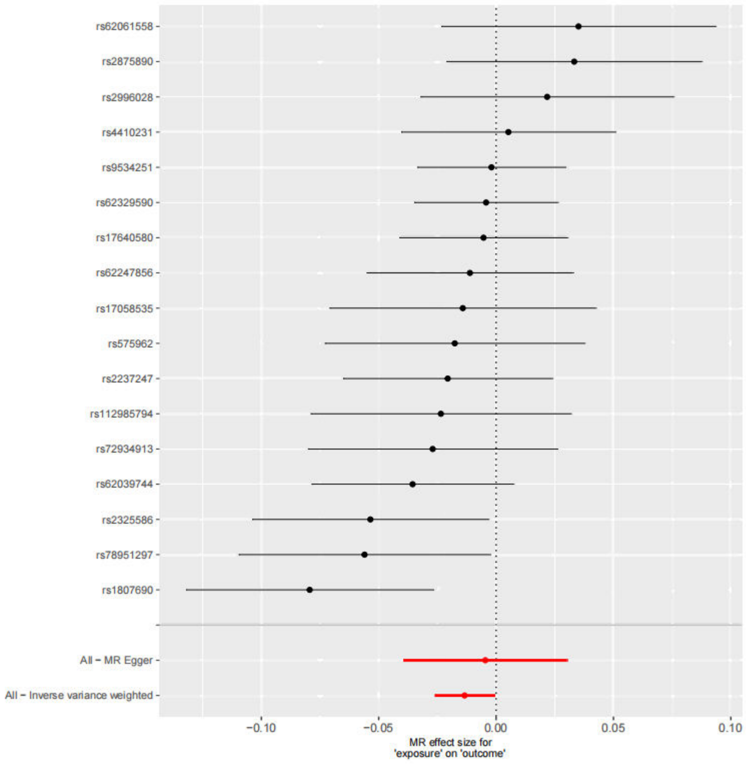
rs62061558
rs2875890
rs2996028
rs4410231
rs9534251
rs62329590
rs17640580
rs62247856
rs17058535
rs575962
rs2237247
rs112985794
rs72934913
rs62039744
rs2325586
rs78951297
rs1807690
All − MR Egger
All − Inverse variance weighted
−0.10
−0.05
0.00
0.05
0.10
MR effect size for
'exposure' on 'outcome'

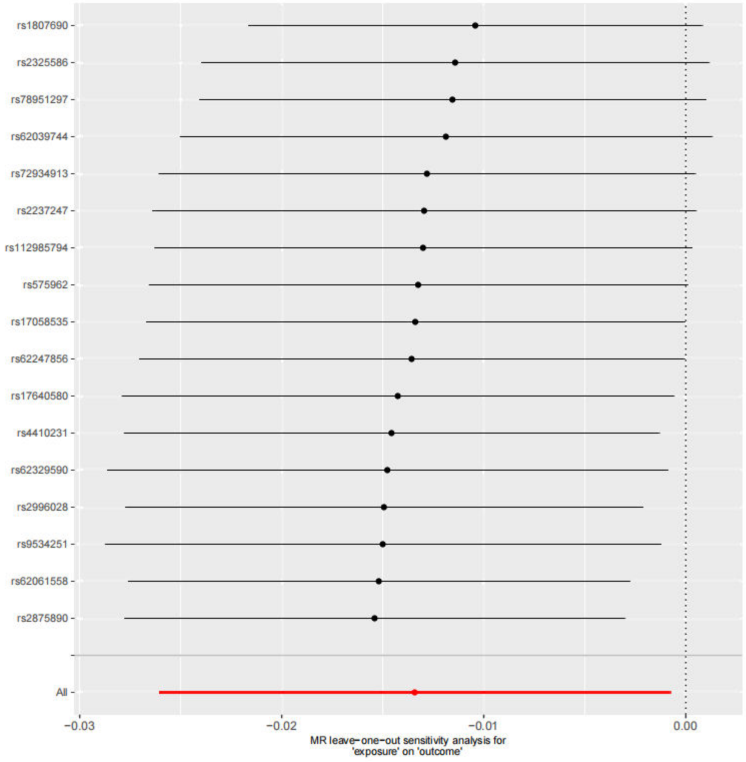
rs1807690
rs2325586
rs78951297
rs62039744
rs72934913
rs2237247
rs112985794
rs575962
rs17058535
rs62247856
rs17640580
rs4410231
rs62329590
rs2996028
rs9534251
rs62061558
rs2875890
All
−0.03
−0.02
−0.01
0.00
MR leave−one−out sensitivity analysis for
'exposure' on 'outcome'

GCST90257094

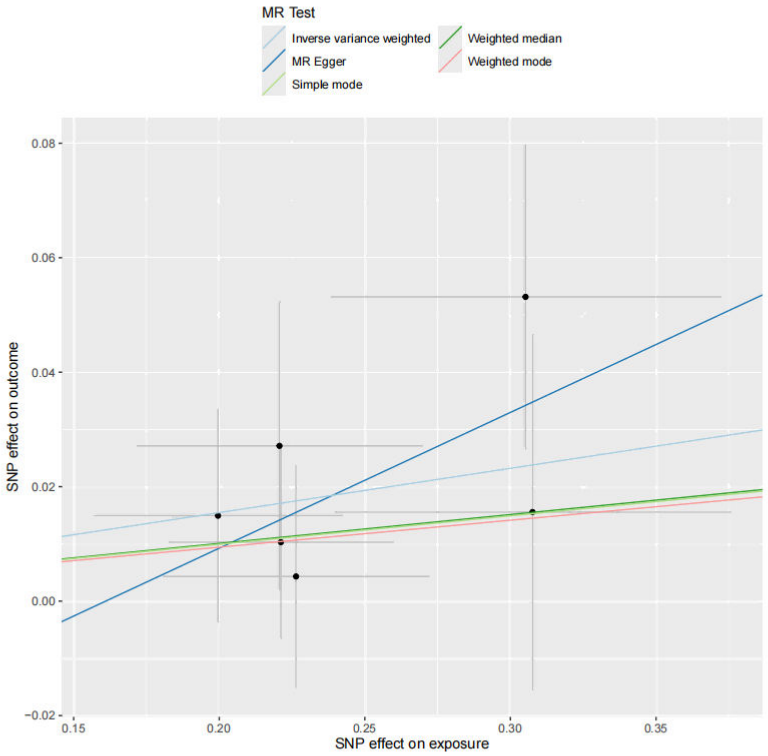

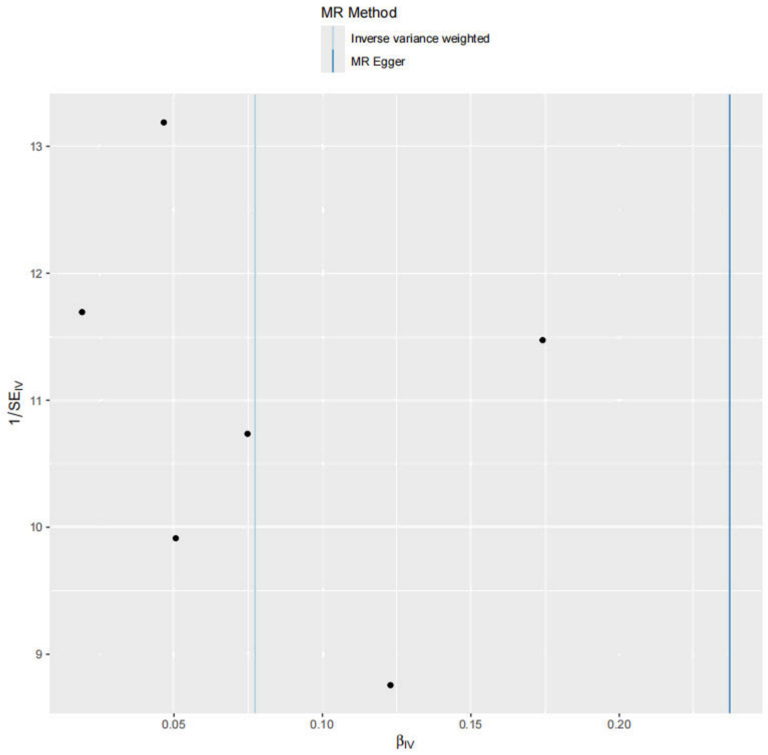

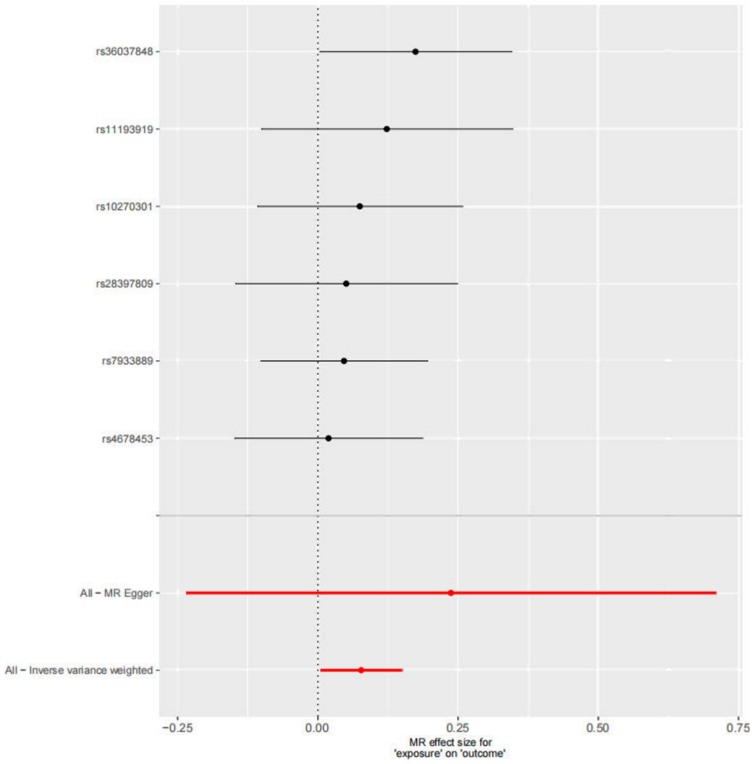
rs36037848
rs11193919
rs10270301
rs28397809
rs7933889
rs4678453
All − MR Egger
All − Inverse variance weighted
−0.25
0.00
0.25
0.50
0.75
MR effect size for
'exposure' on 'outcome'

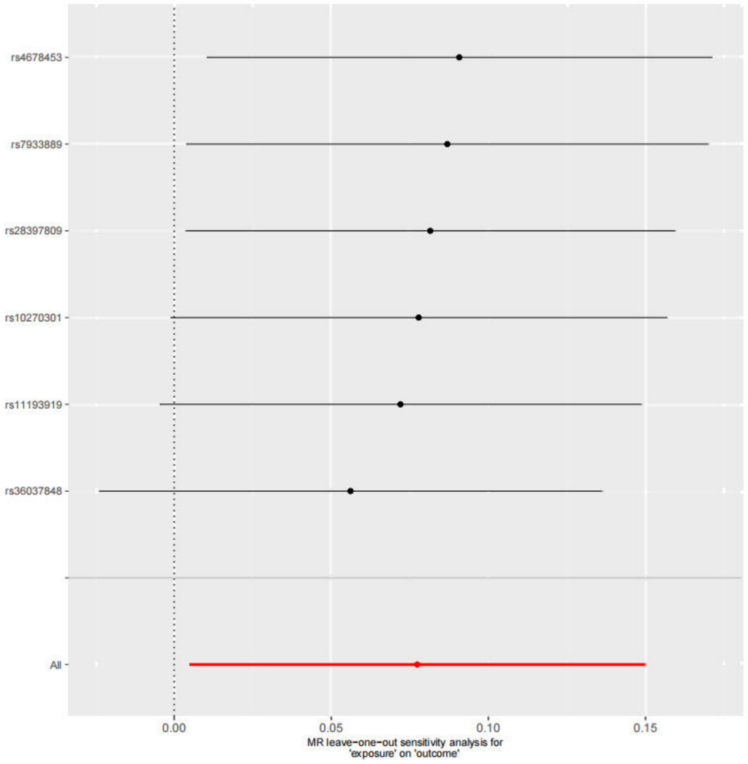
rs4678453
rs7933889
rs28397809
rs10270301
rs11193919
rs36037848
All
0.00
0.05
0.10
0.15
MR leave−one−out sensitivity analysis for
'exposure' on 'outcome'

GCST90257021

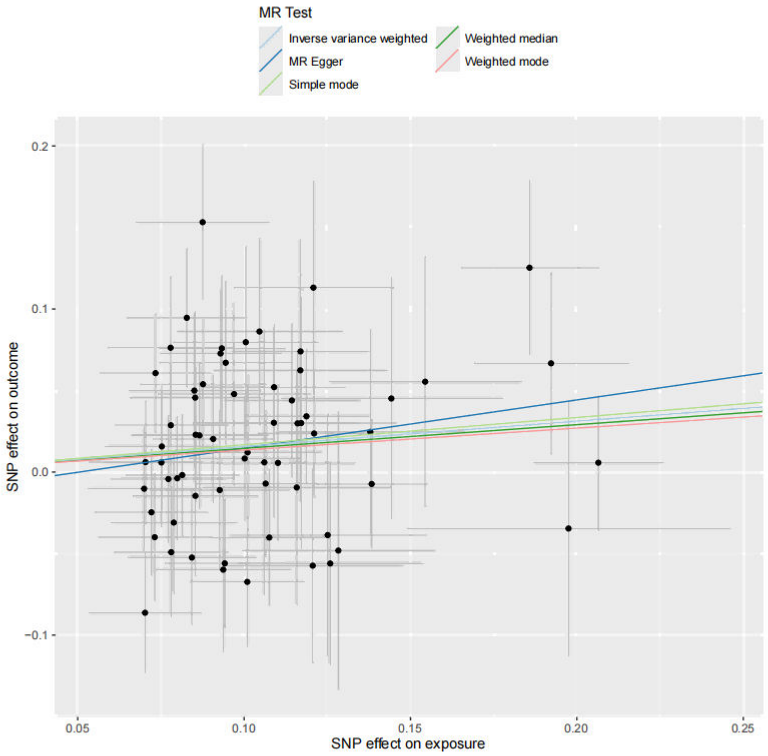

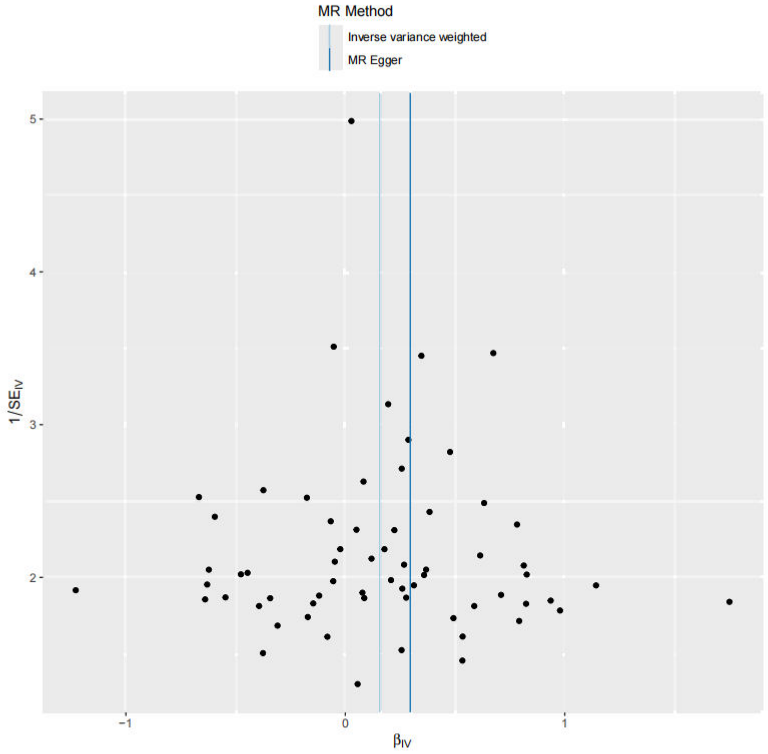

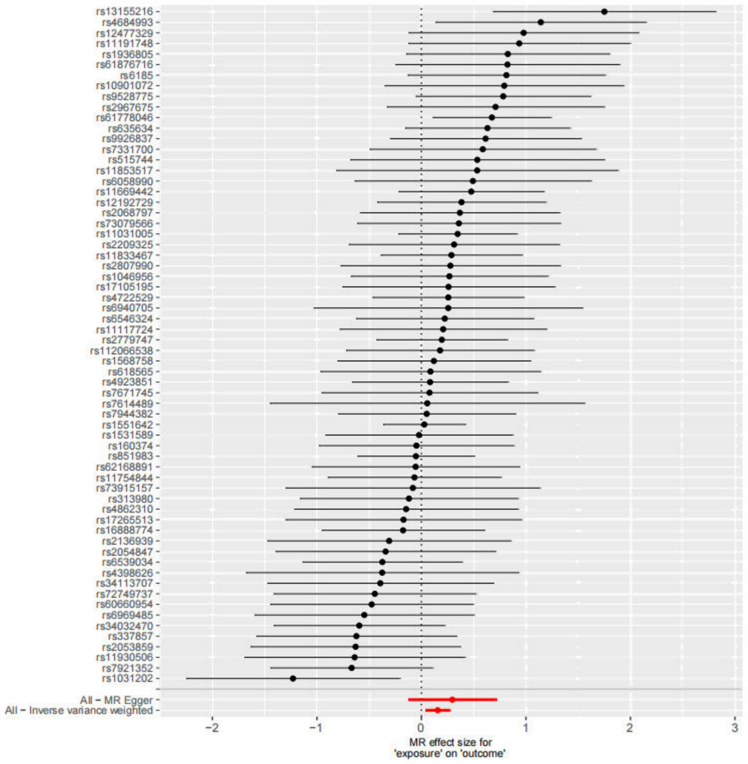
All − MR Egger
All − Inverse variance weighted
MR effect size for
'exposure' on 'outcome'

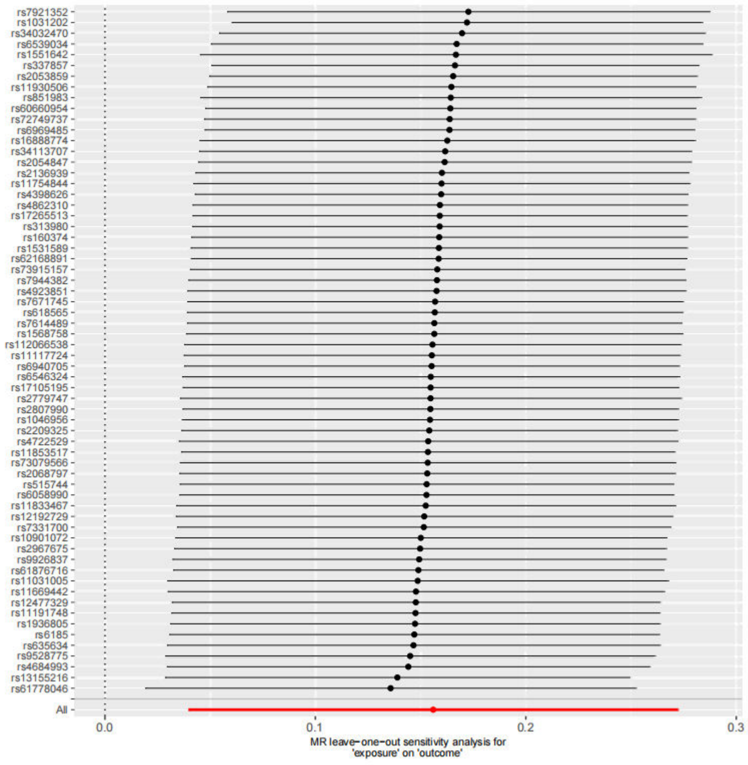
All
MR leave−one−out sensitivity analysis for
'exposure' on 'outcome'

GCST90257022

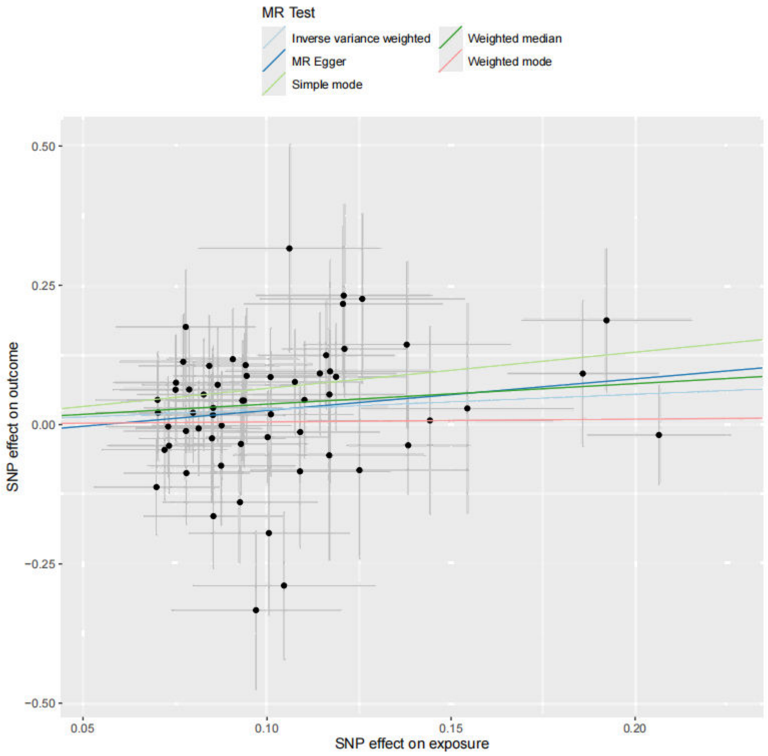

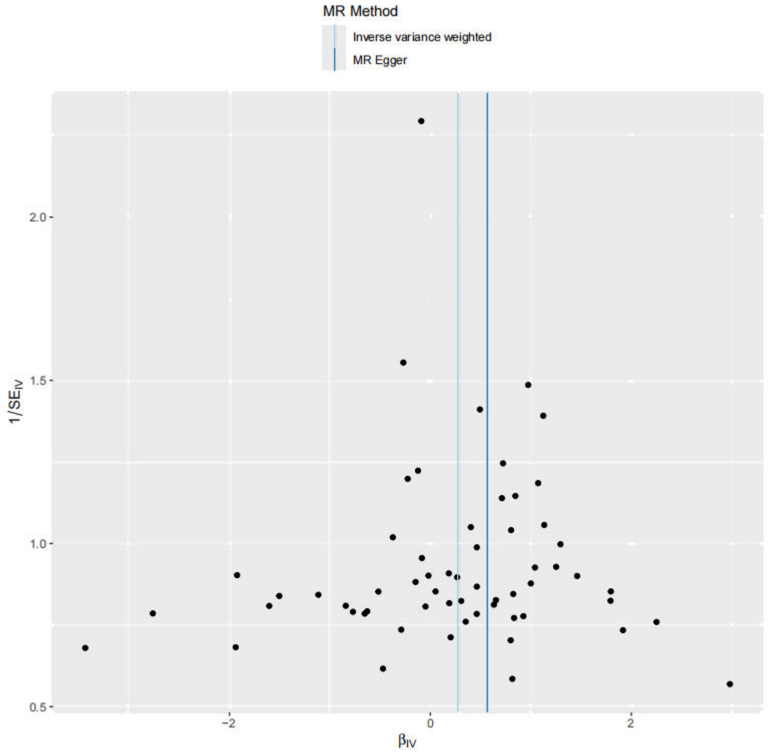

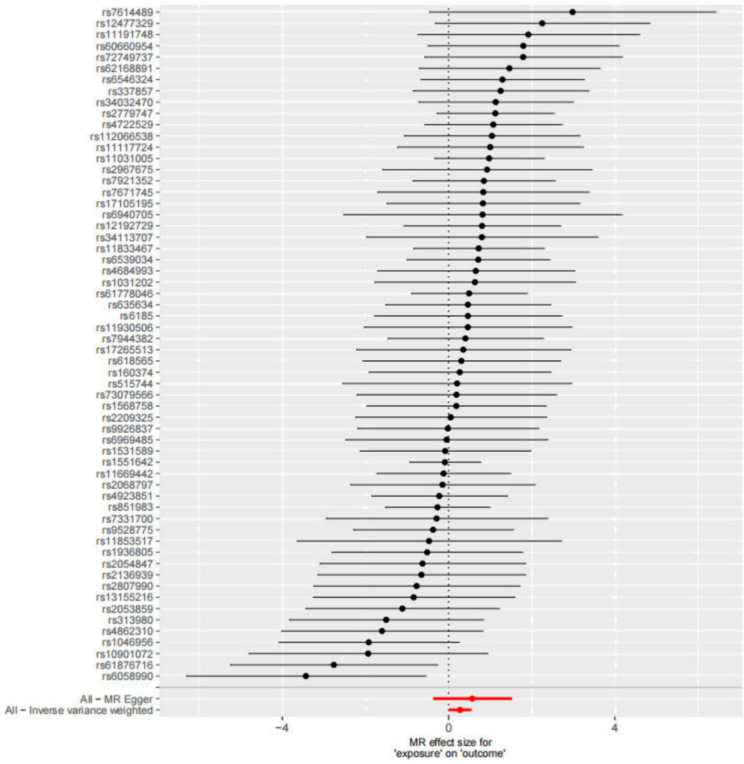
rs7614489
rs12477329
rs11191748
rs60660954
rs72749737
rs62168891
rs6546324
rs337857
rs34032470
rs2779747
rs4722529
rs112066538
rs11117724
rs11031005
rs2967675
rs7921352
rs7671745
rs17105195
rs6940705
rs12192729
rs34113707
rs11833467
rs6539034
rs4684993
rs1031202
rs61778046
rs635634
rs6185
rs11930506
rs7944382
rs17265513
rs618565
rs160374
rs515744
rs73079566
rs1568758
rs2209325
rs9926837
rs6969485
rs1531589
rs1551642
rs11669442
rs2068797
rs4923851
rs851983
rs7331700
rs9528775
rs11853517
rs1936805
rs2054847
rs2136939
rs2807990
rs13155216
rs2053859
rs313980
rs4862310
rs1046956
rs10901072
rs61876716
rs6058990
All − MR Egger
All − Inverse variance weighted
−4
0
4
MR effect size for
'exposure' on 'outcome'

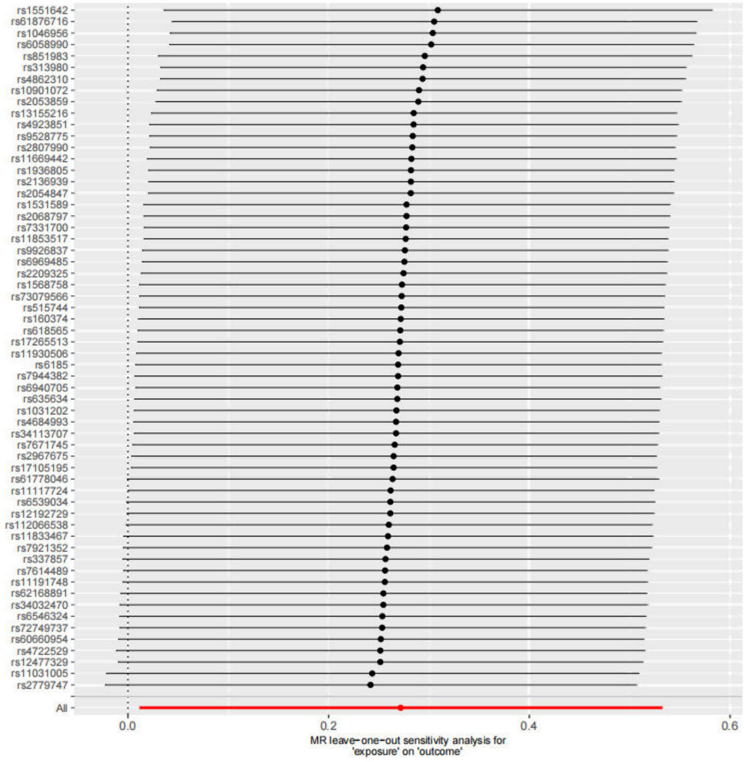
rs1551642
rs61876716
rs1046956
rs6058990
rs851983
rs313980
rs4862310
rs10901072
rs2053859
rs13155216
rs4923851
rs9528775
rs2807990
rs11669442
rs1936805
rs2136939
rs2054847
rs1531589
rs2068797
rs7331700
rs11853517
rs9926837
rs6969485
rs2209325
rs1568758
rs73079566
rs515744
rs160374
rs618565
rs17265513
rs11930506
rs6185
rs7944382
rs6940705
rs635634
rs1031202
rs4684993
rs34113707
rs7671745
rs2967675
rs17105195
rs61778046
rs11117724
rs6539034
rs12192729
rs112066538
rs11833467
rs7921352
rs337857
rs7614489
rs11191748
rs62168891
rs34032470
rs6546324
rs72749737
rs60660954
rs4722529
rs12477329
rs11031005
rs2779747
All
0.0
0.2
0.4
0.6
MR leave−one−out sensitivity analysis for
'exposure' on 'outcome'

GCST90257027

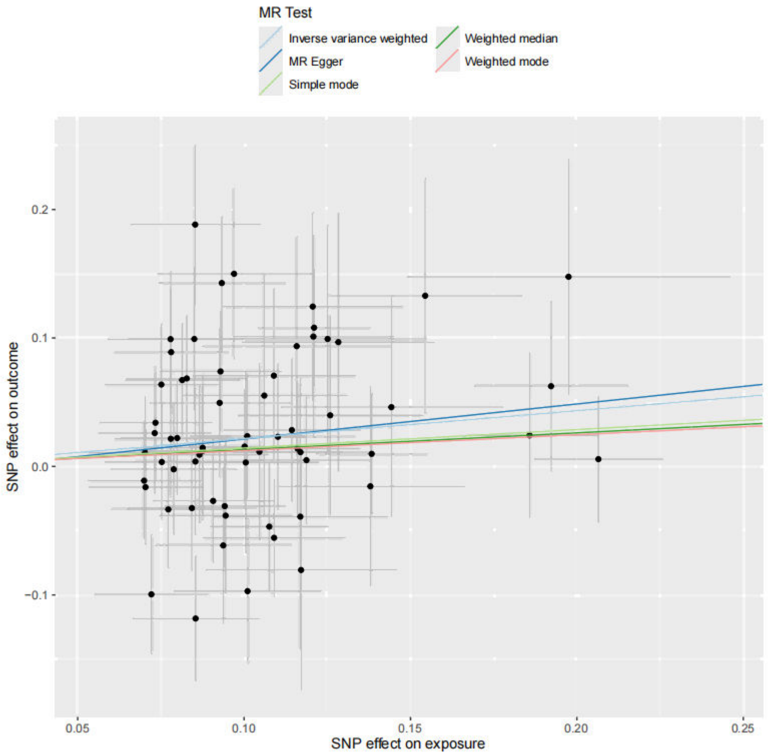

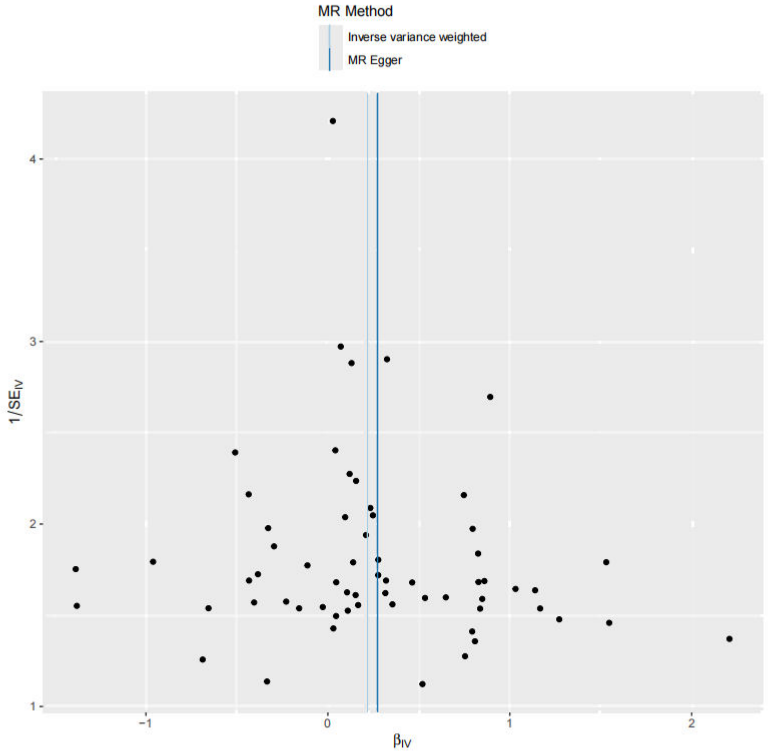

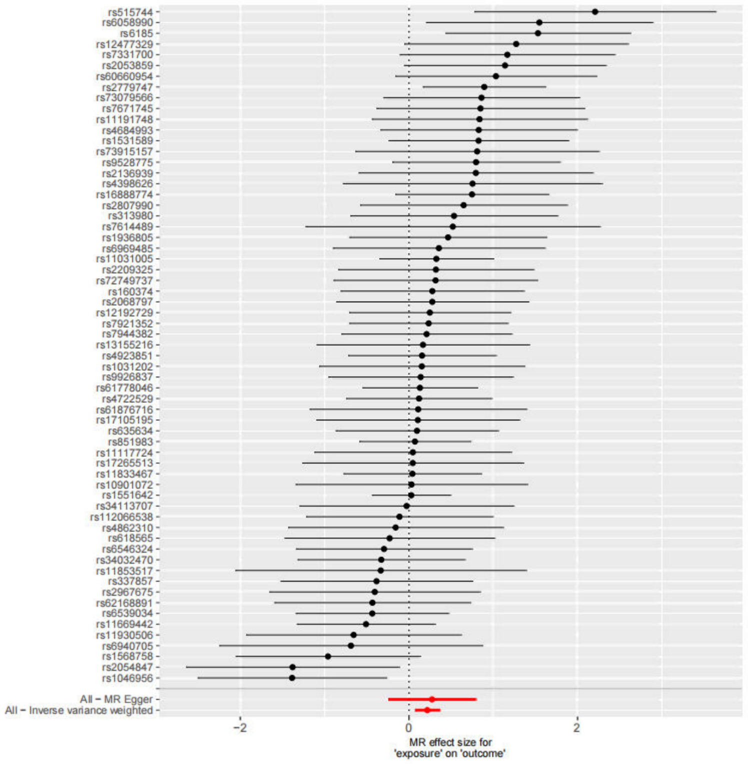
rs515744
rs6058990
rs6185
rs12477329
rs7331700
rs2053859
rs60660954
rs2779747
rs73079566
rs7671745
rs11191748
rs4684993
rs1531589
rs73915157
rs9528775
rs2136939
rs4398626
rs16888774
rs2807990
rs313980
rs7614489
rs1936805
rs6969485
rs11031005
rs2209325
rs72749737
rs160374
rs2068797
rs12192729
rs7921352
rs7944382
rs13155216
rs4923851
rs1031202
rs9926837
rs61778046
rs4722529
rs61876716
rs17105195
rs635634
rs851983
rs11117724
rs17265513
rs11833467
rs10901072
rs1551642
rs34113707
rs112066538
rs4862310
rs618565
rs6546324
rs34032470
rs11853517
rs337857
rs2967675
rs62168891
rs6539034
rs11669442
rs11930506
rs6940705
rs1568758
rs2054847
rs1046956
All − MR Egger
All − Inverse variance weighted
−2
0
2
MR effect size for
'exposure' on 'outcome'

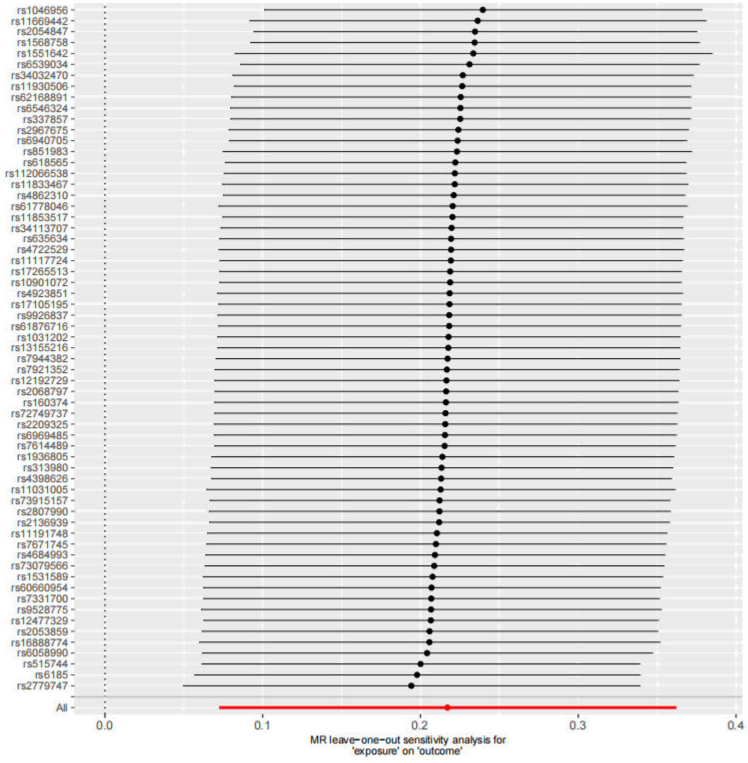
rs1046956
rs11669442
rs2054847
rs1568758
rs1551642
rs6539034
rs34032470
rs11930506
rs62168891
rs6546324
rs337857
rs2967675
rs6940705
rs851983
rs618565
rs112066538
rs11833467
rs4862310
rs61778046
rs11853517
rs34113707
rs635634
rs4722529
rs11117724
rs17265513
rs10901072
rs4923851
rs17105195
rs9926837
rs61876716
rs1031202
rs13155216
rs7944382
rs7921352
rs12192729
rs2068797
rs160374
rs72749737
rs2209325
rs6969485
rs7614489
rs1936805
rs313980
rs4398626
rs11031005
rs73915157
rs2807990
rs2136939
rs11191748
rs7671745
rs4684993
rs73079566
rs1531589
rs60660954
rs7331700
rs9528775
rs12477329
rs2053859
rs16888774
rs6058990
rs515744
rs6185
rs2779747
All
0.0
0.1
0.2
0.3
0.4
MR leave−one−out sensitivity analysis for
'exposure' on 'outcome'

GCST90257031

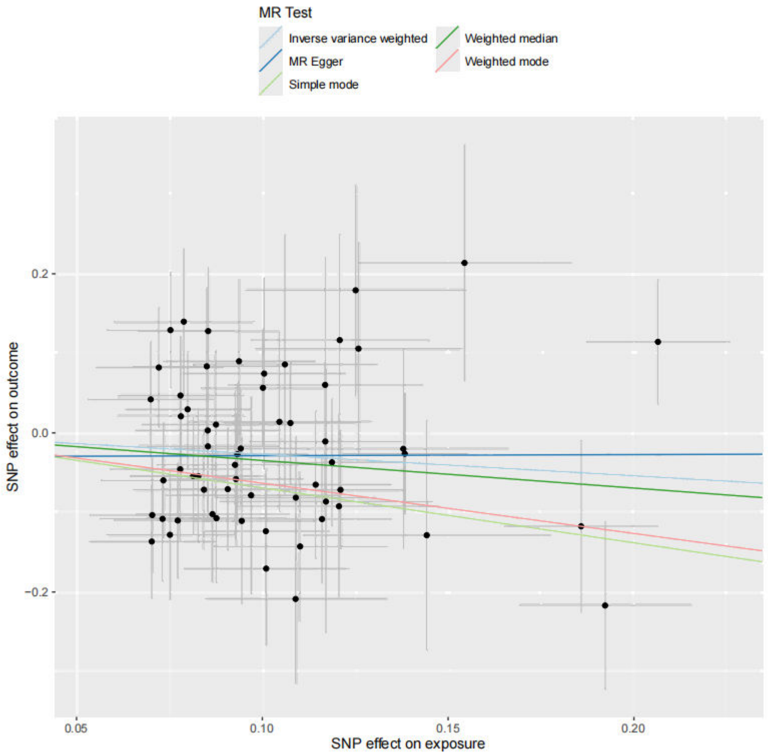

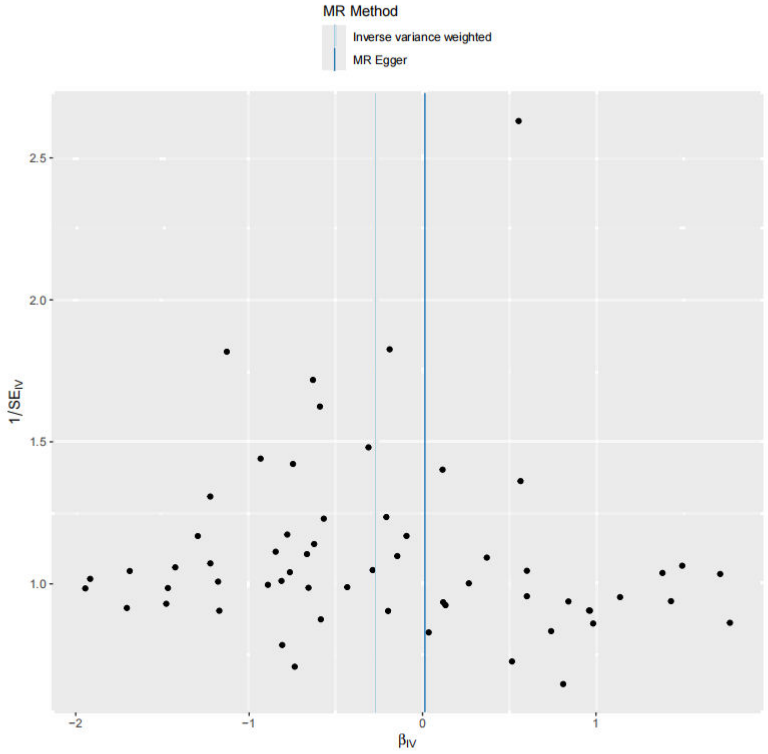

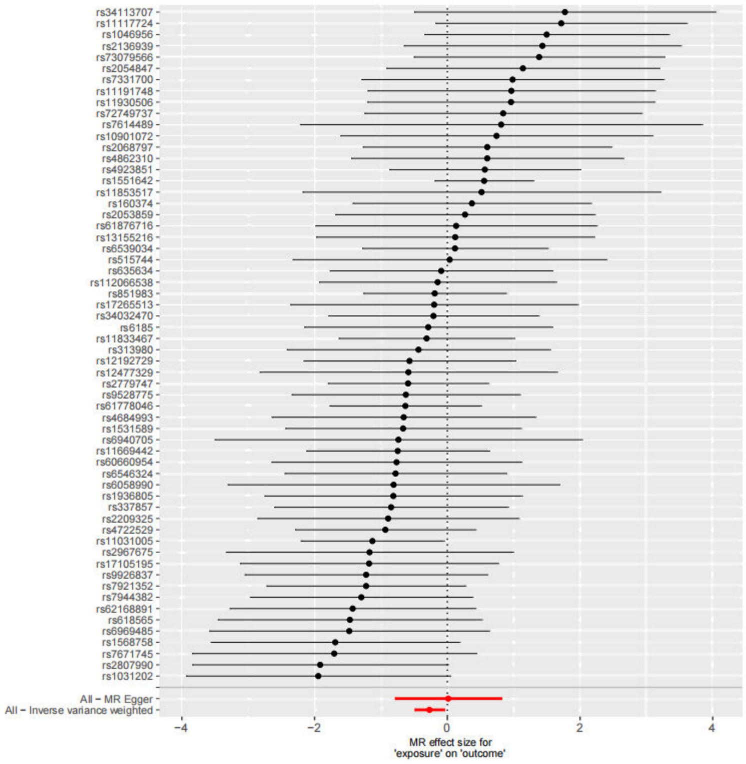
rs34113707
rs11117724
rs1046956
rs2136939
rs73079566
rs2054847
rs7331700
rs11191748
rs11930506
rs72749737
rs7614489
rs10901072
rs2068797
rs4862310
rs4923851
rs1551642
rs11853517
rs160374
rs2053859
rs61876716
rs13155216
rs6539034
rs515744
rs635634
rs112066538
rs851983
rs17265513
rs34032470
rs6185
rs11833467
rs313980
rs12192729
rs12477329
rs2779747
rs9528775
rs61778046
rs4684993
rs1531589
rs6940705
rs11669442
rs60660954
rs6546324
rs6058990
rs1936805
rs337857
rs2209325
rs4722529
rs11031005
rs2967675
rs17105195
rs9926837
rs7921352
rs7944382
rs62168891
rs618565
rs6969485
rs1568758
rs7671745
rs2807990
rs1031202
All − MR Egger
All − Inverse variance weighted
−4
−2
0
2
4
MR effect size for
'exposure' on 'outcome'

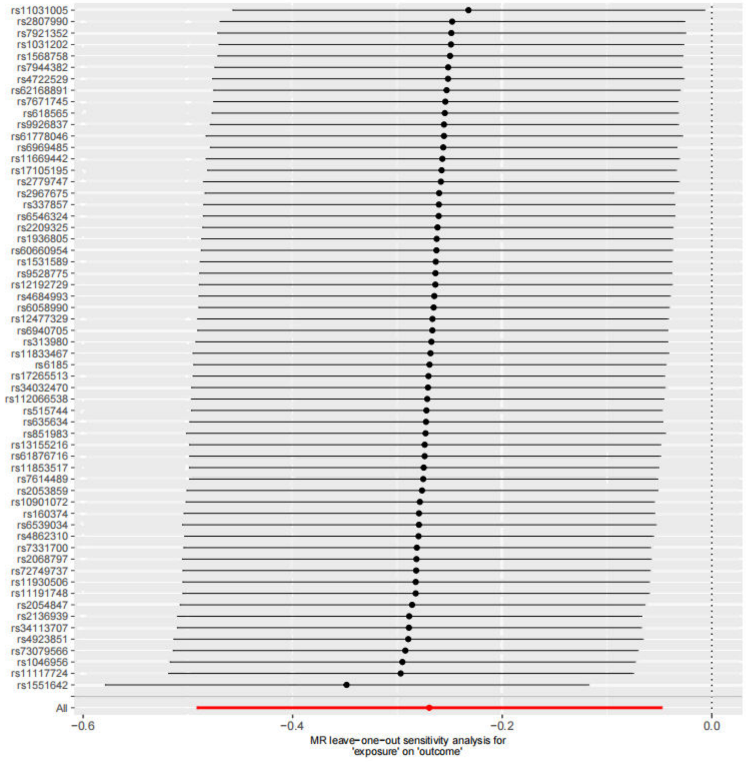
rs11031005
rs2807990
rs7921352
rs1031202
rs1568758
rs7944382
rs4722529
rs62168891
rs7671745
rs618565
rs9926837
rs61778046
rs6969485
rs11669442
rs17105195
rs2779747
rs2967675
rs337857
rs6546324
rs2209325
rs1936805
rs60660954
rs1531589
rs9528775
rs12192729
rs4684993
rs6058990
rs12477329
rs6940705
rs313980
rs11833467
rs6185
rs17265513
rs34032470
rs112066538
rs515744
rs635634
rs851983
rs13155216
rs61876716
rs11853517
rs7614489
rs2053859
rs10901072
rs160374
rs6539034
rs4862310
rs7331700
rs2068797
rs72749737
rs11930506
rs11191748
rs2054847
rs2136939
rs34113707
rs4923851
rs73079566
rs1046956
rs11117724
rs1551642
All
−0.6
−0.4
−0.2
0.0
MR leave−one−out sensitivity analysis for
'exposure' on 'outcome'

GCST90257072

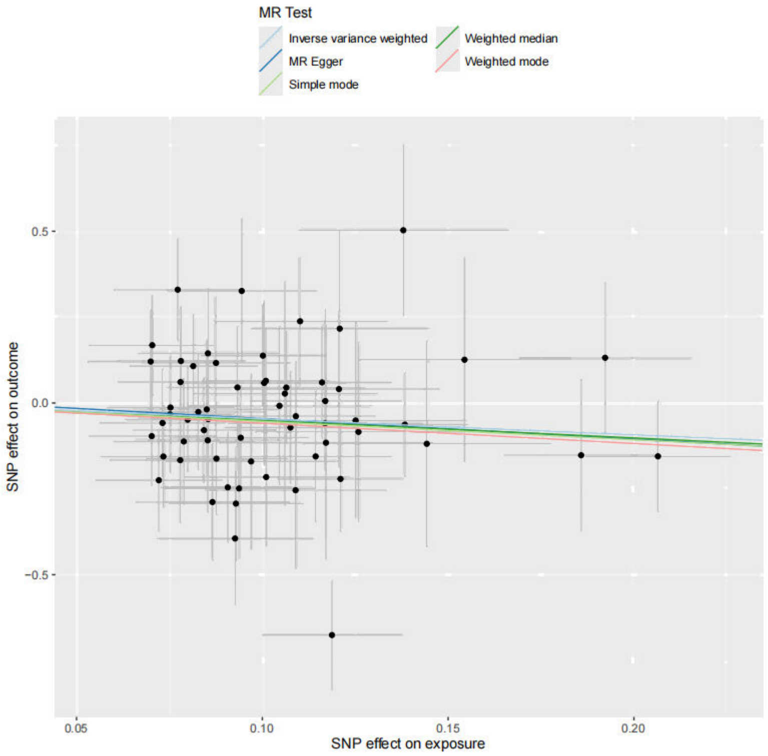

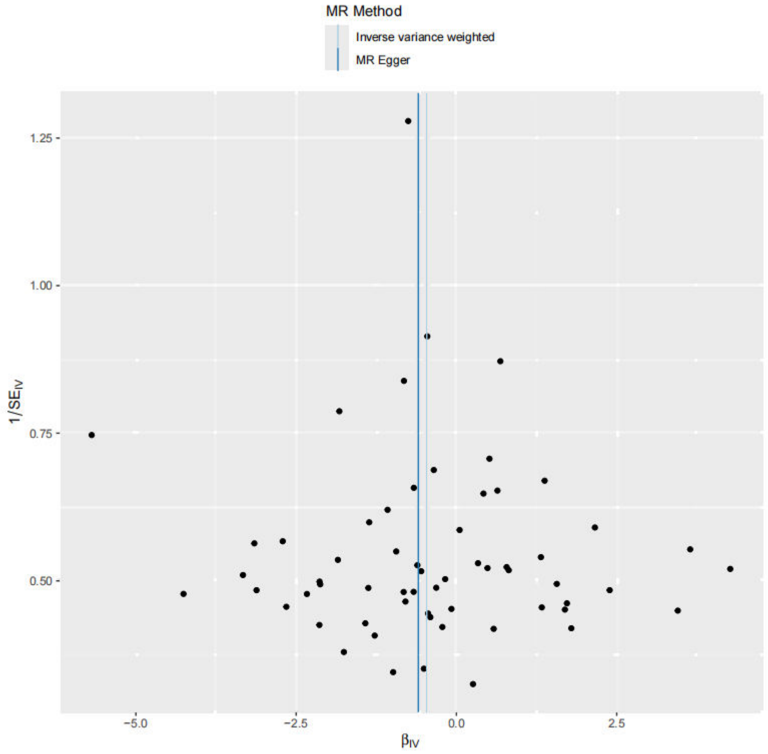

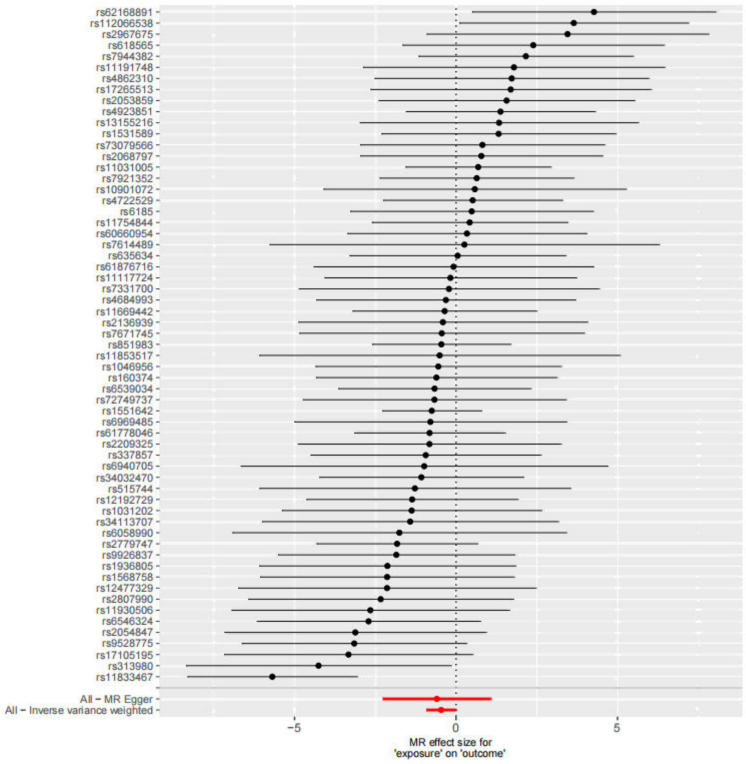
rs62168891
rs112066538
rs2967675
rs618565
rs7944382
rs11191748
rs4862310
rs17265513
rs2053859
rs4923851
rs13155216
rs1531589
rs73079566
rs2068797
rs11031005
rs7921352
rs10901072
rs4722529
rs6185
rs11754844
rs60660954
rs7614489
rs635634
rs61876716
rs11117724
rs7331700
rs4684993
rs11669442
rs2136939
rs7671745
rs851983
rs11853517
rs1046956
rs160374
rs6539034
rs72749737
rs1551642
rs6969485
rs61778046
rs2209325
rs337857
rs6940705
rs34032470
rs515744
rs12192729
rs1031202
rs34113707
rs6058990
rs2779747
rs9926837
rs1936805
rs1568758
rs12477329
rs2807990
rs11930506
rs6546324
rs2054847
rs9528775
rs17105195
rs313980
rs11833467
All − MR Egger
All − Inverse variance weighted
−5
0
5
MR effect size for
'exposure' on 'outcome'

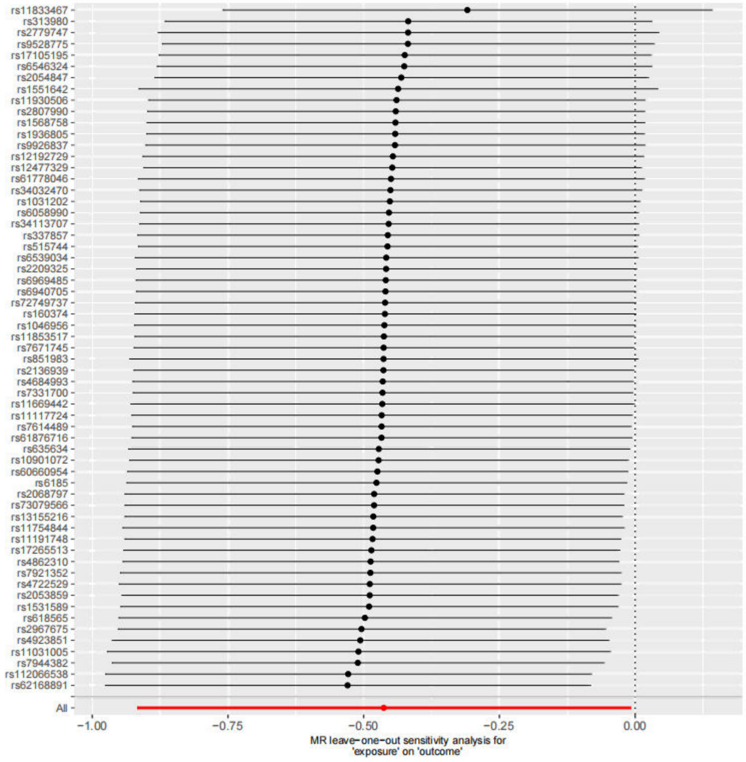
rs11833467
rs313980
rs2779747
rs9528775
rs17105195
rs6546324
rs2054847
rs1551642
rs11930506
rs2807990
rs1568758
rs1936805
rs9926837
rs12192729
rs12477329
rs61778046
rs34032470
rs1031202
rs6058990
rs34113707
rs337857
rs515744
rs6539034
rs2209325
rs6969485
rs6940705
rs72749737
rs160374
rs1046956
rs11853517
rs7671745
rs851983
rs2136939
rs4684993
rs7331700
rs11669442
rs11117724
rs7614489
rs61876716
rs635634
rs10901072
rs60660954
rs6185
rs2068797
rs73079566
rs13155216
rs11754844
rs11191748
rs17265513
rs4862310
rs7921352
rs4722529
rs2053859
rs1531589
rs618565
rs2967675
rs4923851
rs11031005
rs7944382
rs112066538
rs62168891
All
−1.00
−0.75
−0.50
−0.25
0.00
MR leave−one−out sensitivity analysis for
'exposure' on 'outcome'

GCST90257024

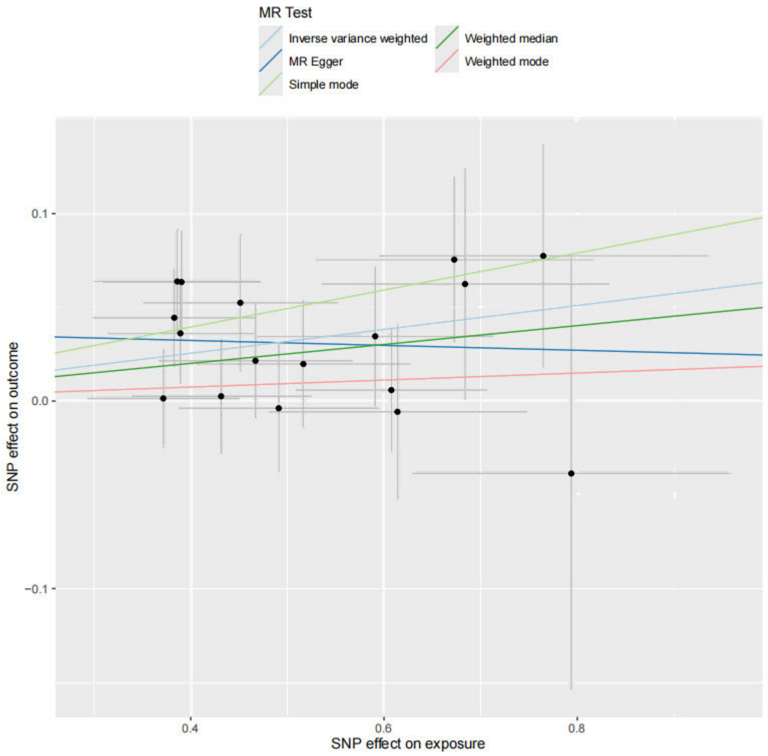

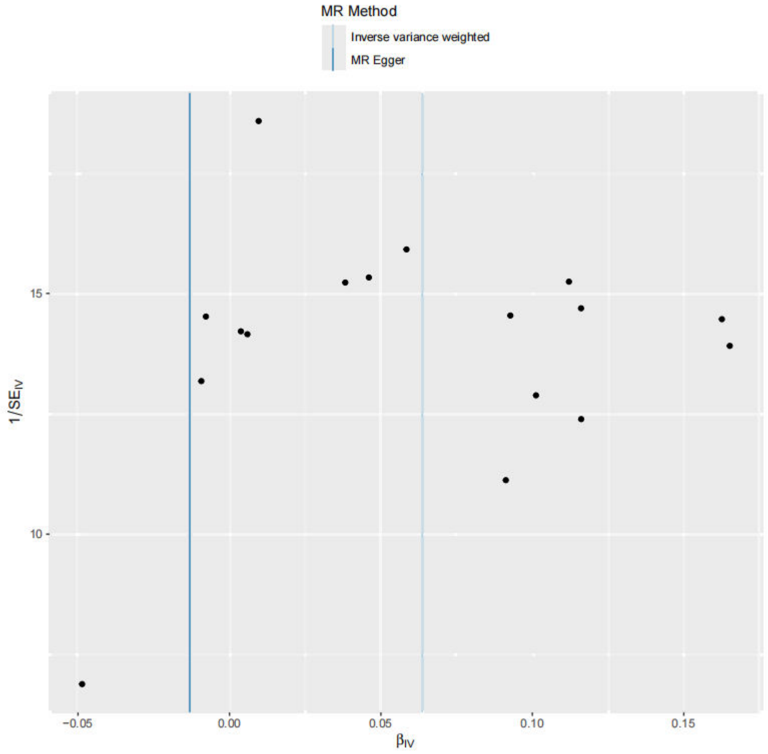

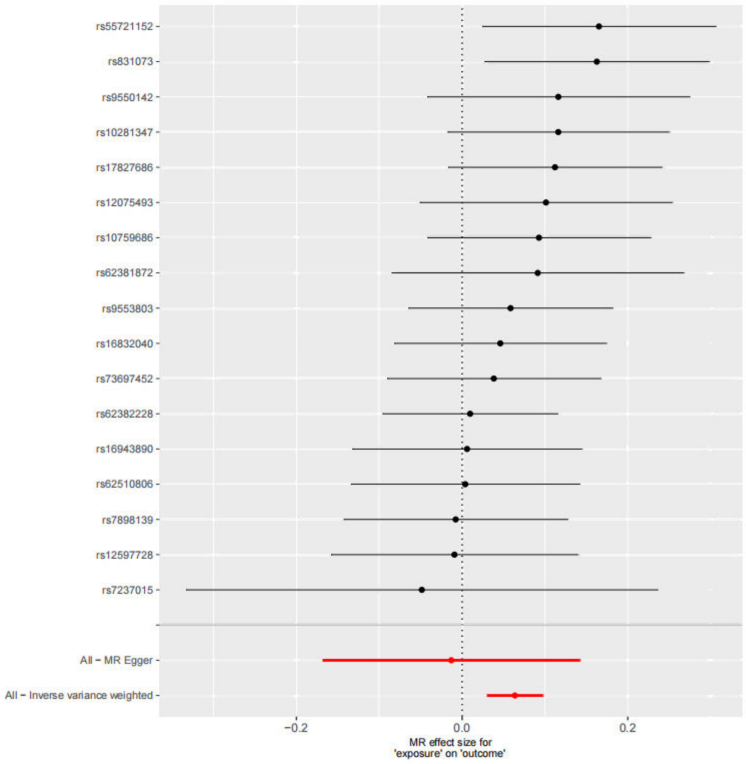
rs55721152
rs831073
rs9550142
rs10281347
rs17827686
rs12075493
rs10759686
rs62381872
rs9553803
rs16832040
rs73697452
rs62382228
rs16943890
rs62510806
rs7898139
rs12597728
rs7237015
All − MR Egger
All − Inverse variance weighted
−0.2
0.0
0.2
MR effect size for
'exposure' on 'outcome'

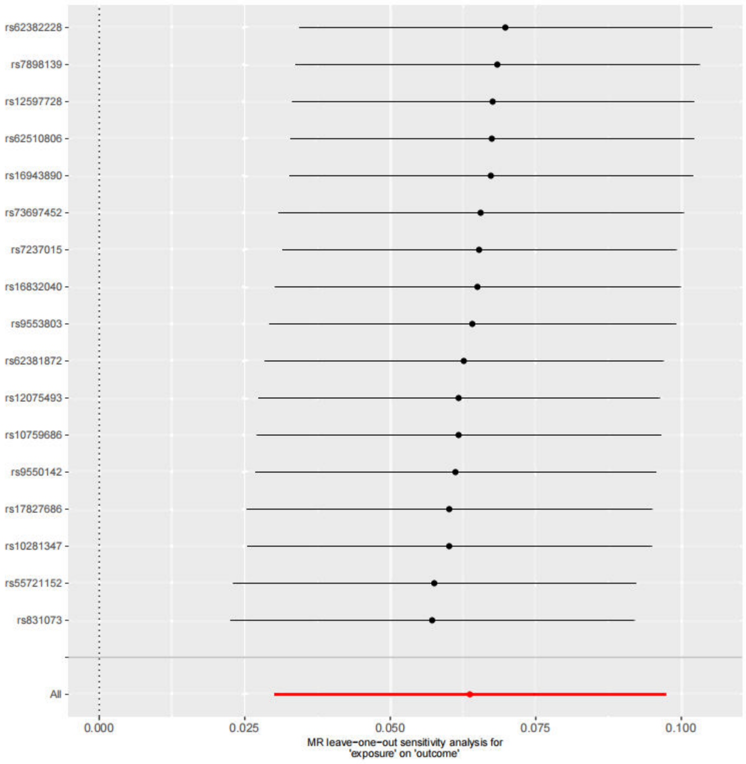
rs62382228
rs7898139
rs12597728
rs62510806
rs16943890
rs73697452
rs7237015
rs16832040
rs9553803
rs62381872
rs12075493
rs10759686
rs9550142
rs17827686
rs10281347
rs55721152
rs831073
All
0.000
0.025
0.050
0.075
0.100
MR leave−one−out sensitivity analysis for
'exposure' on 'outcome'

GCST90257029

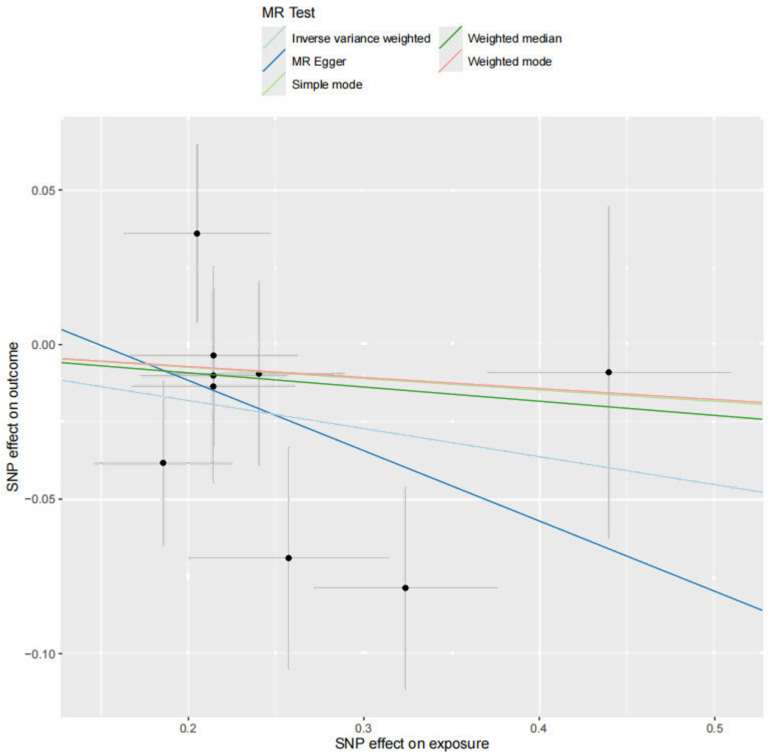

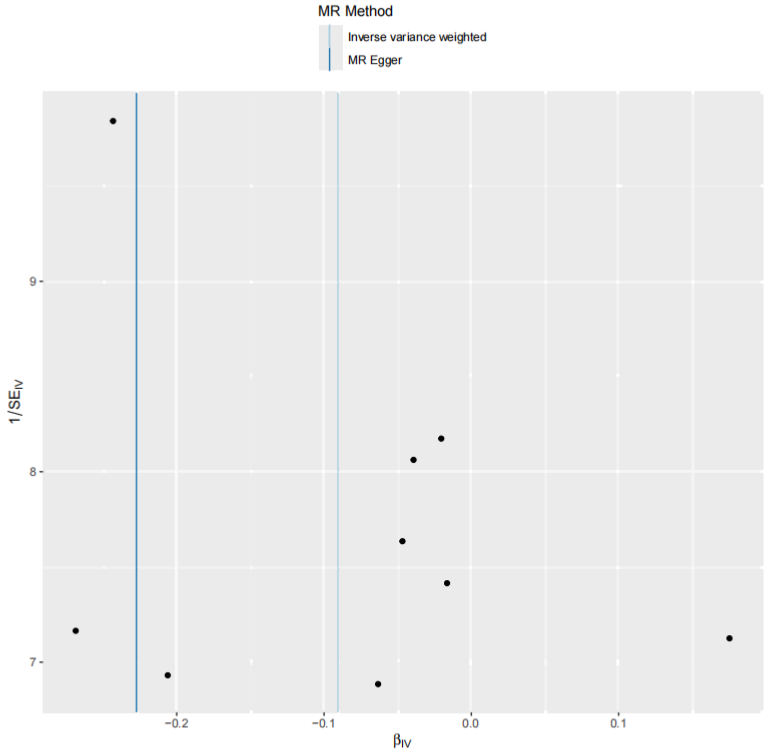

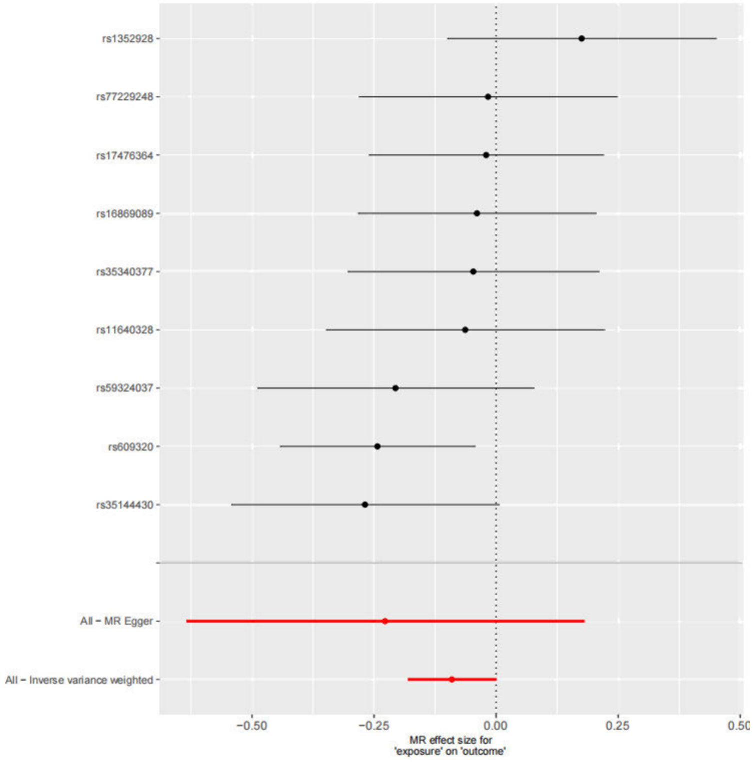
rs1352928
rs77229248
rs17476364
rs16869089
rs35340377
rs11640328
rs59324037
rs609320
rs35144430
All − MR Egger
All − Inverse variance weighted
−0.50
−0.25
0.00
0.25
0.50
MR effect size for
'exposure' on 'outcome'

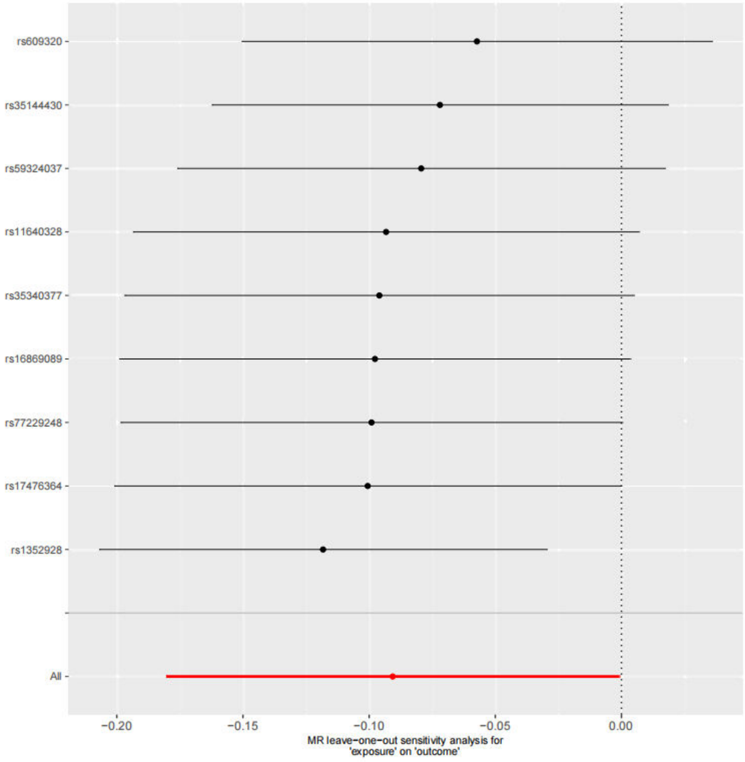
rs609320
rs35144430
rs59324037
rs11640328
rs35340377
rs16869089
rs77229248
rs17476364
rs1352928
All
−0.20
−0.15
−0.10
−0.05
0.00
MR leave−one−out sensitivity analysis for
'exposure' on 'outcome'

GCST90257021

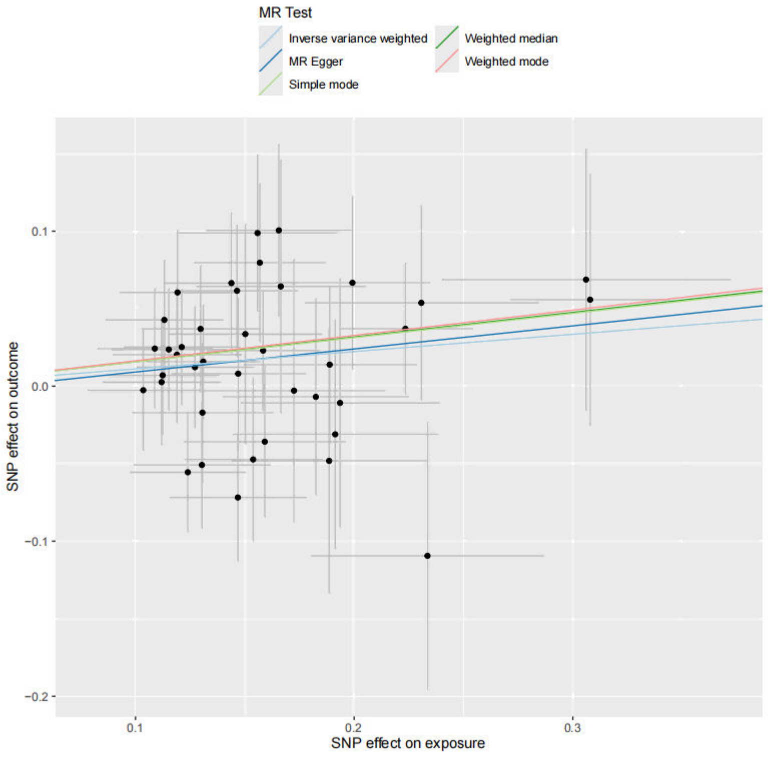

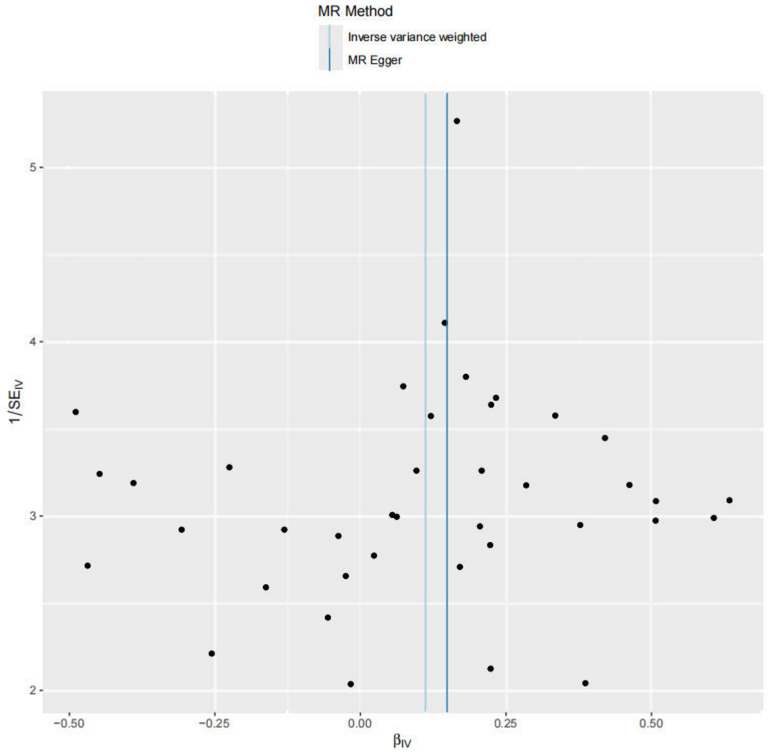

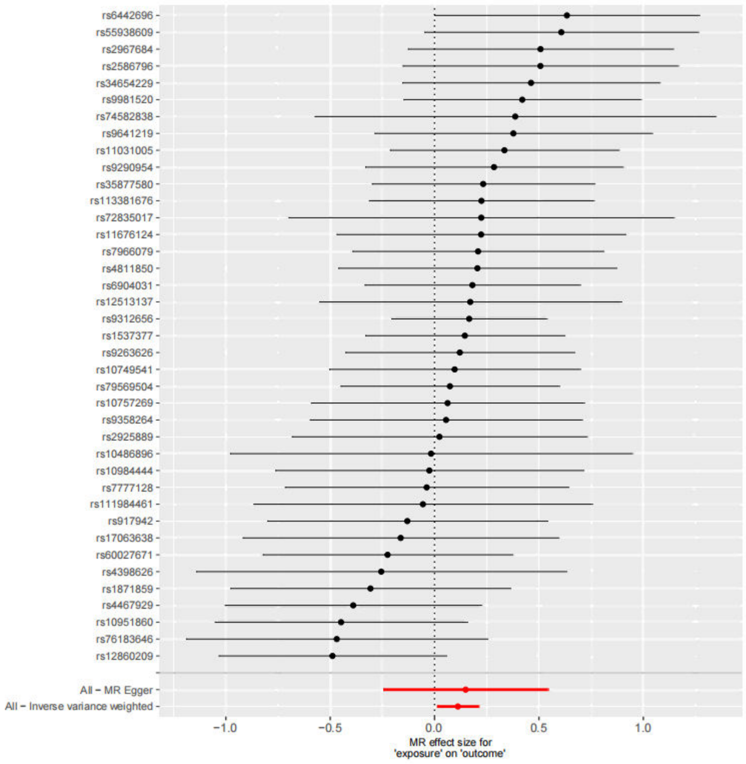

rs6442696
rs55938609
rs2967684
rs2586796
rs34654229
rs9981520
rs74582838
rs9641219
rs11031005
rs9290954
rs35877580
rs113381676
rs72835017
rs11676124
rs7966079
rs4811850
rs6904031
rs12513137
rs9312656
rs1537377
rs9263626
rs10749541
rs79569504
rs10757269
rs9358264
rs2925889
rs10486896
rs10984444
rs7777128
rs111984461
rs917942
rs17063638
rs60027671
rs4398626
rs1871859
rs4467929
rs10951860
rs76183646
rs12860209
All − MR Egger
All − Inverse variance weighted
−1.0
−0.5
0.0
0.5
1.0
MR effect size for
'exposure' on 'outcome'

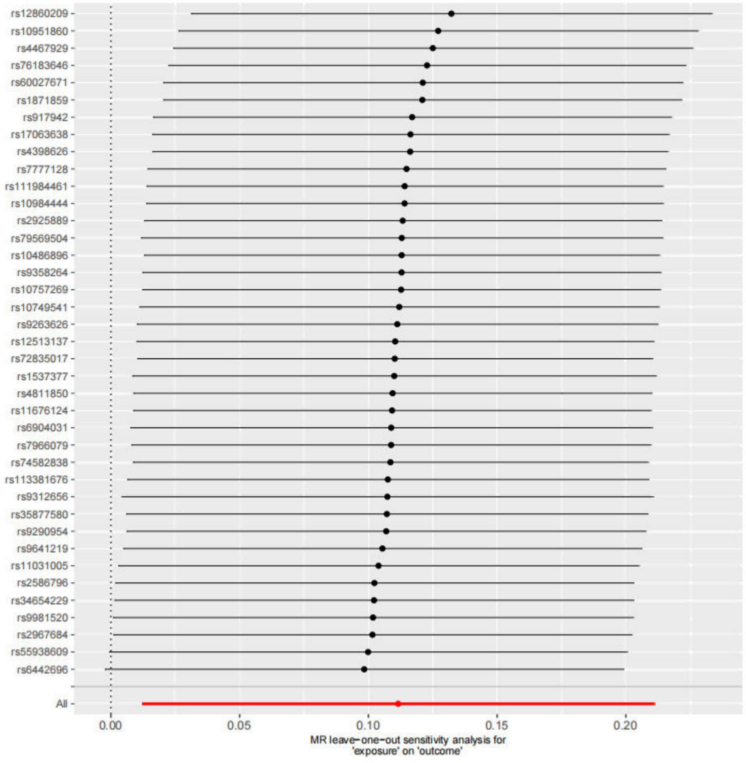

rs12860209
rs10951860
rs4467929
rs76183646
rs60027671
rs1871859
rs917942
rs17063638
rs4398626
rs7777128
rs111984461
rs10984444
rs2925889
rs79569504
rs10486896
rs9358264
rs10757269
rs10749541
rs9263626
rs12513137
rs72835017
rs1537377
rs4811850
rs11676124
rs6904031
rs7966079
rs74582838
rs113381676
rs9312656
rs35877580
rs9290954
rs9641219
rs11031005
rs2586796
rs34654229
rs9981520
rs2967684
rs55938609
rs6442696
All
0.00
0.05
0.10
0.15
0.20
MR leave−one−out sensitivity analysis for
'exposure' on 'outcome'

GCST90257059

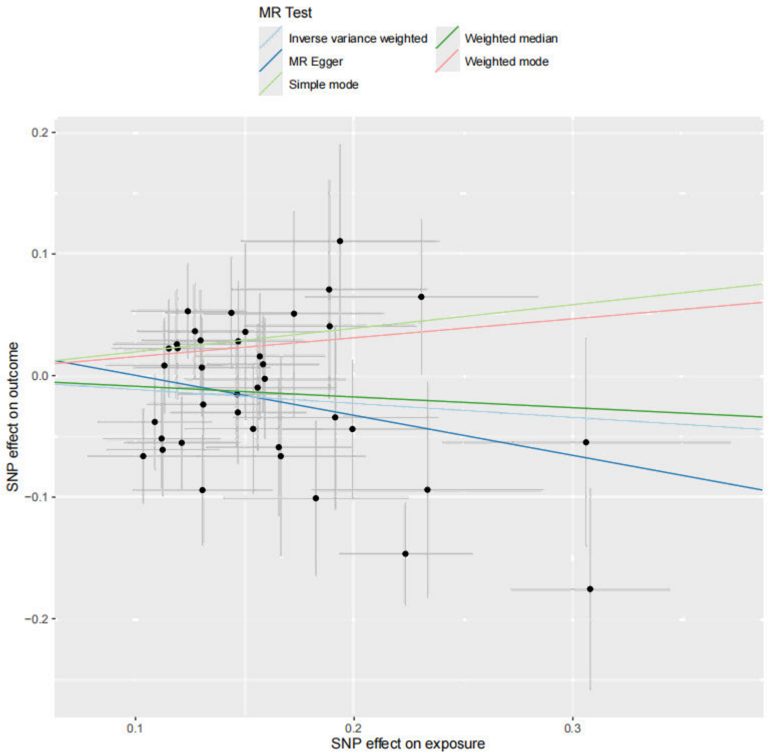

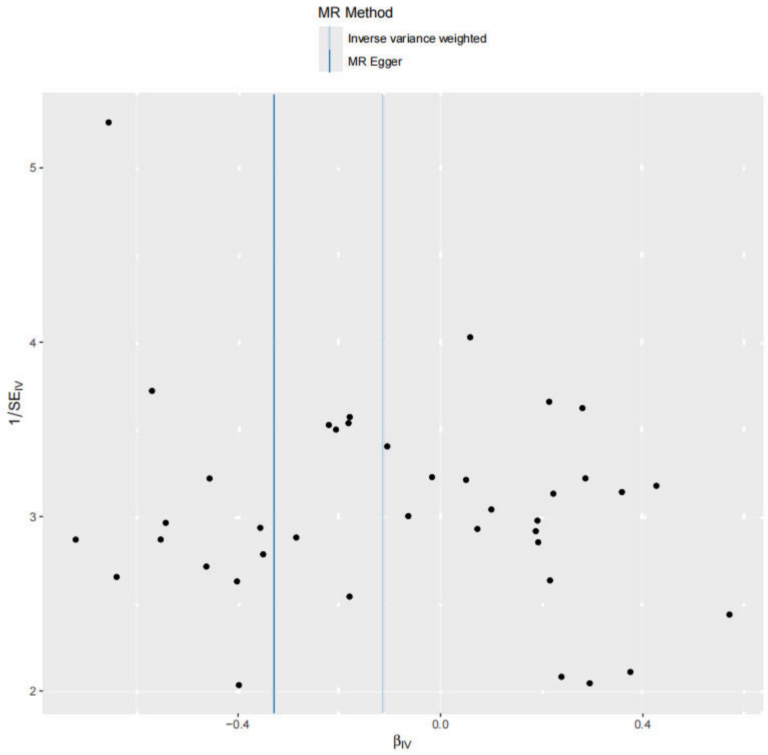

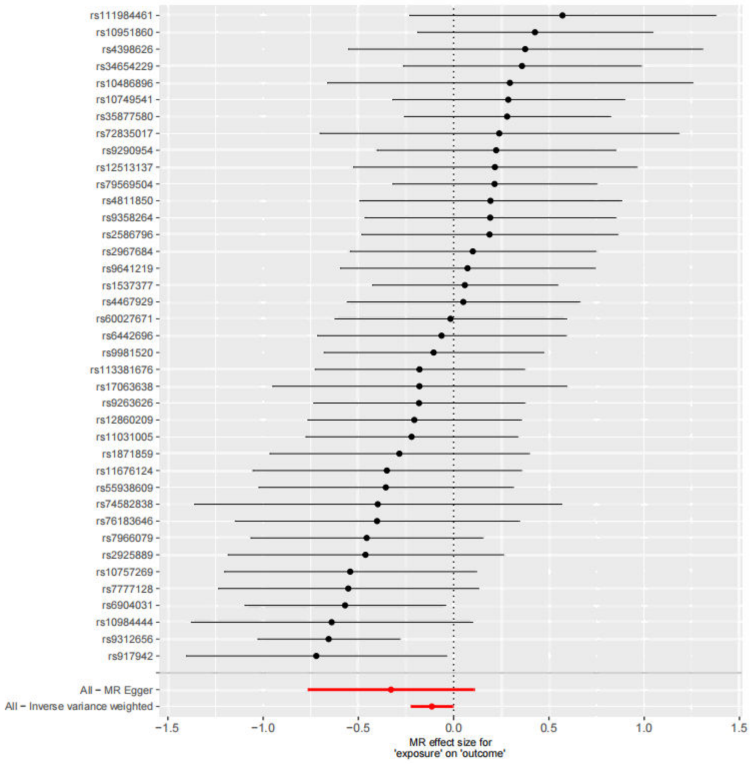
rs111984461
rs10951860
rs4398626
rs34654229
rs10486896
rs10749541
rs35877580
rs72835017
rs9290954
rs12513137
rs79569504
rs4811850
rs9358264
rs2586796
rs2967684
rs9641219
rs1537377
rs4467929
rs60027671
rs6442696
rs9981520
rs113381676
rs17063638
rs9263626
rs12860209
rs11031005
rs1871859
rs11676124
rs55938609
rs74582838
rs76183646
rs7966079
rs2925889
rs10757269
rs7777128
rs6904031
rs10984444
rs9312656
rs917942
All − MR Egger
All − Inverse variance weighted
−1.5
−1.0
−0.5
0.0
0.5
1.0
1.5
MR effect size for
'exposure' on 'outcome'

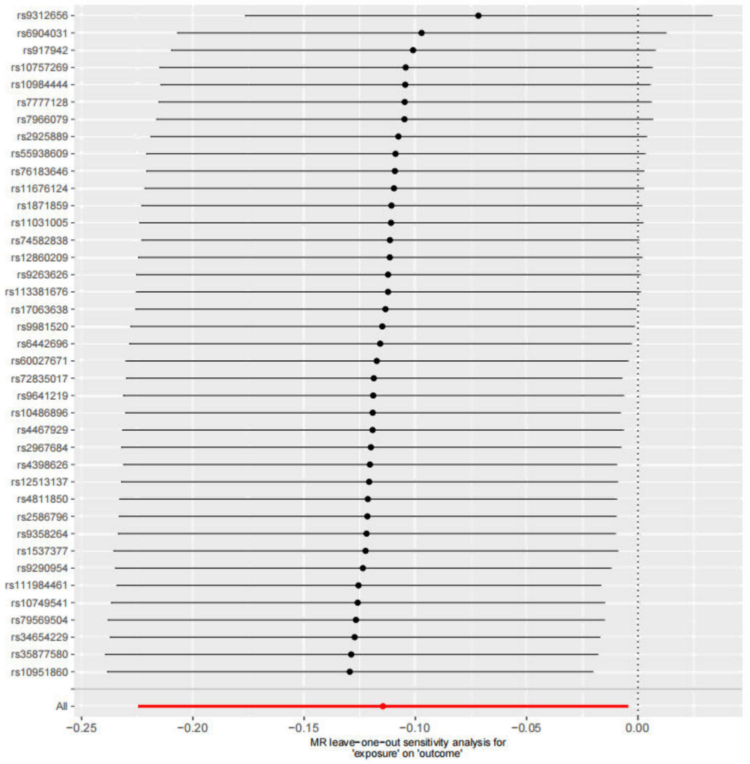
rs9312656
rs6904031
rs917942
rs10757269
rs10984444
rs7777128
rs7966079
rs2925889
rs55938609
rs76183646
rs11676124
rs1871859
rs11031005
rs74582838
rs12860209
rs9263626
rs113381676
rs17063638
rs9981520
rs6442696
rs60027671
rs72835017
rs9641219
rs10486896
rs4467929
rs2967684
rs4398626
rs12513137
rs4811850
rs2586796
rs9358264
rs1537377
rs9290954
rs111984461
rs10749541
rs79569504
rs34654229
rs35877580
rs10951860
All
−0.25
−0.20
−0.15
−0.10
−0.05
0.00
MR leave−one−out sensitivity analysis for
'exposure' on 'outcome'

GCST90257099

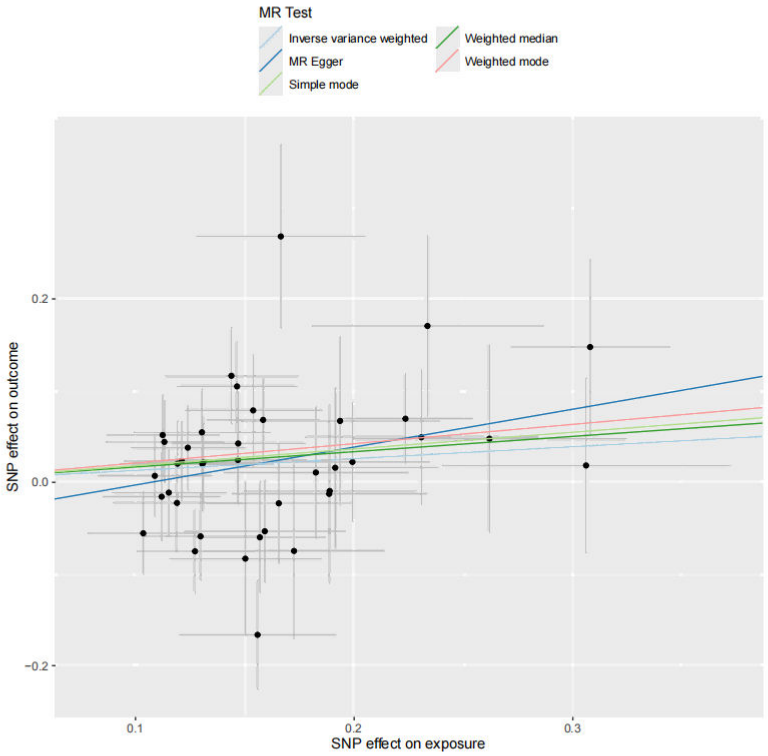

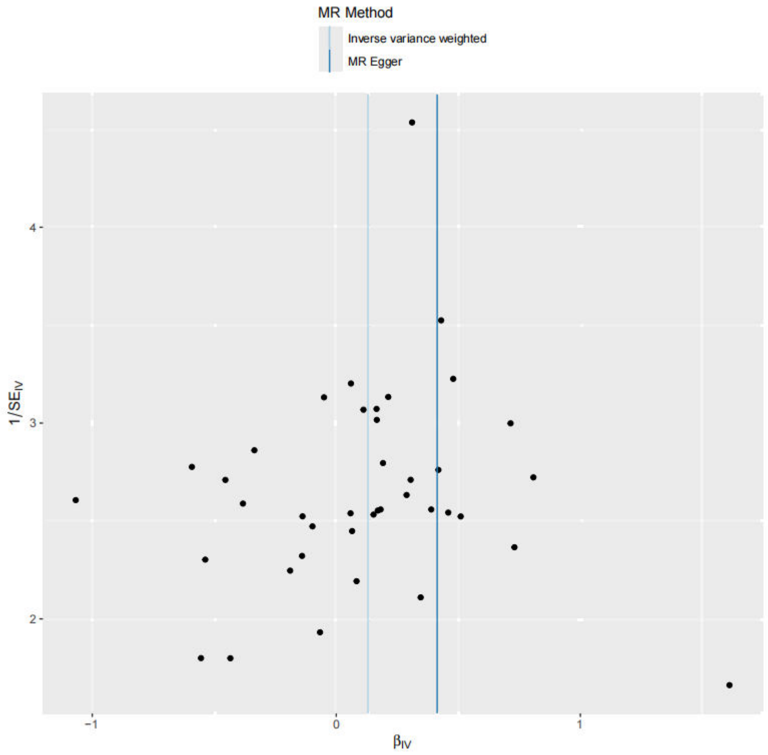

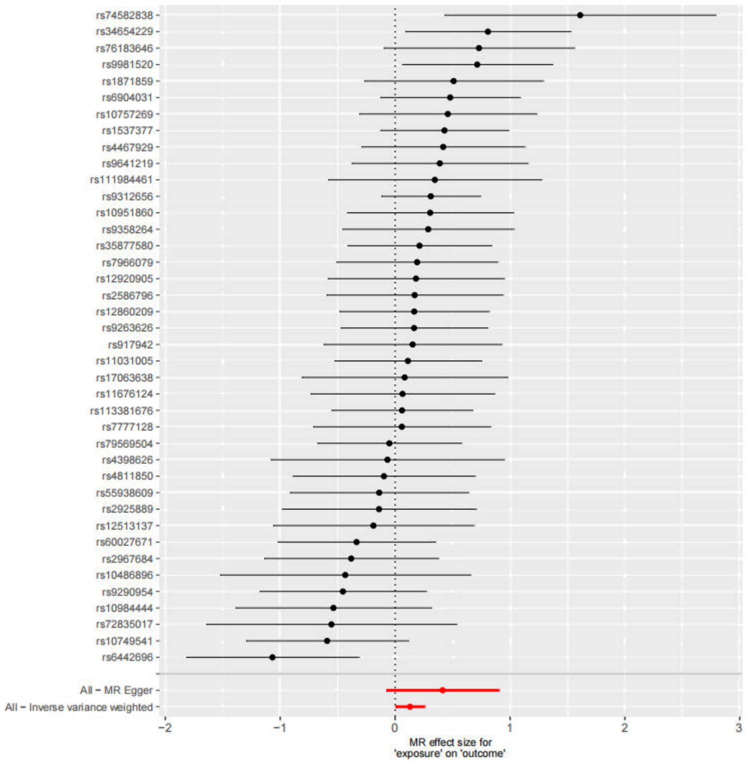
rs74582838
rs34654229
rs76183646
rs9981520
rs1871859
rs6904031
rs10757269
rs1537377
rs4467929
rs9641219
rs111984461
rs9312656
rs10951860
rs9358264
rs35877580
rs7966079
rs12920905
rs2586796
rs12860209
rs9263626
rs917942
rs11031005
rs17063638
rs11676124
rs113381676
rs7777128
rs79569504
rs4398626
rs4811850
rs55938609
rs2925889
rs12513137
rs60027671
rs2967684
rs10486896
rs9290954
rs10984444
rs72835017
rs10749541
rs6442696
All − MR Egger
All − Inverse variance weighted
−2
−1
0
1
2
3
MR effect size for
'exposure' on 'outcome'

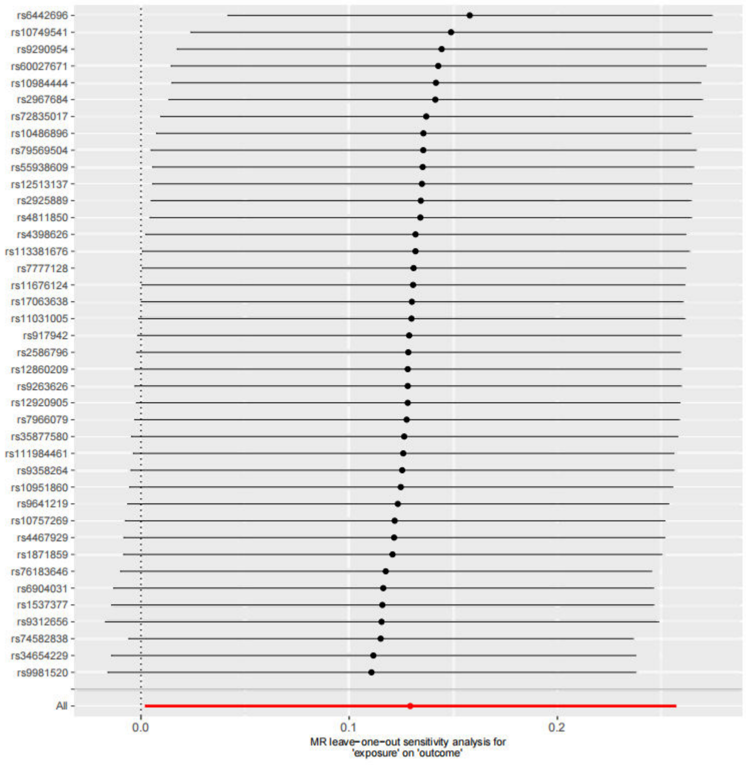
rs6442696
rs10749541
rs9290954
rs60027671
rs10984444
rs2967684
rs72835017
rs10486896
rs79569504
rs55938609
rs12513137
rs2925889
rs4811850
rs4398626
rs113381676
rs7777128
rs11676124
rs17063638
rs11031005
rs917942
rs2586796
rs12860209
rs9263626
rs12920905
rs7966079
rs35877580
rs111984461
rs9358264
rs10951860
rs9641219
rs10757269
rs4467929
rs1871859
rs76183646
rs6904031
rs1537377
rs9312656
rs74582838
rs34654229
rs9981520
All
0.0
0.1
0.2
MR leave−one−out sensitivity analysis for
'exposure' on 'outcome'

GCST90257063

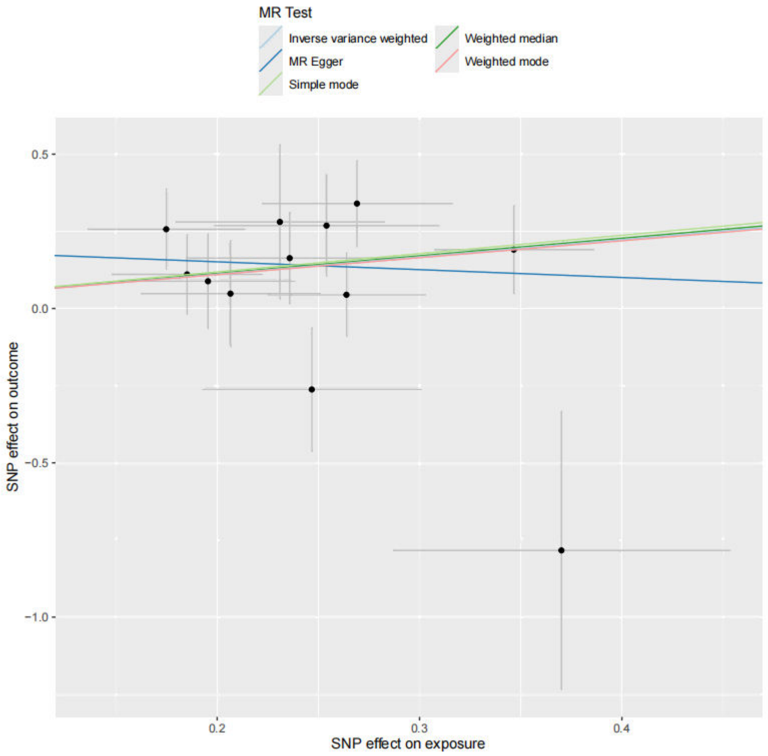

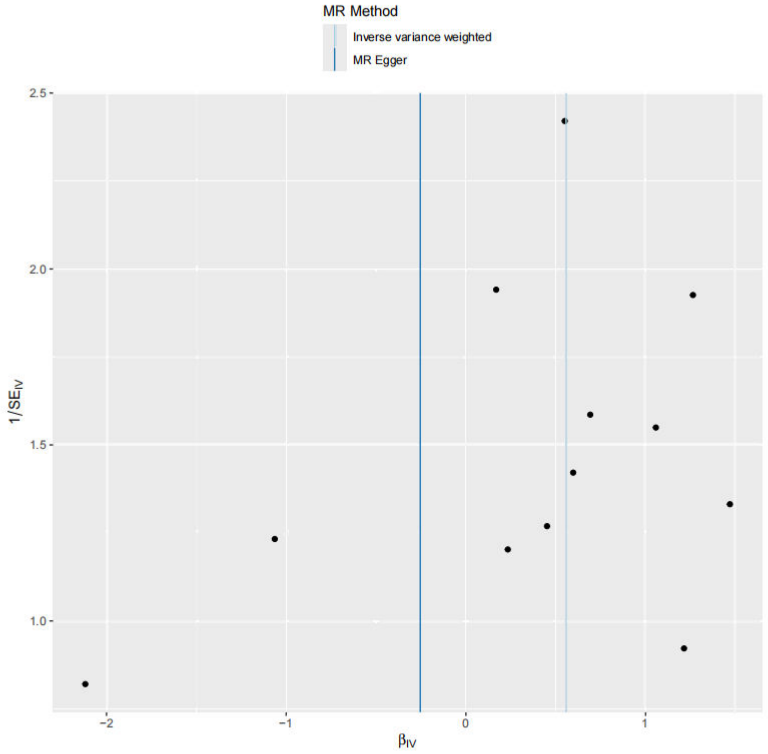

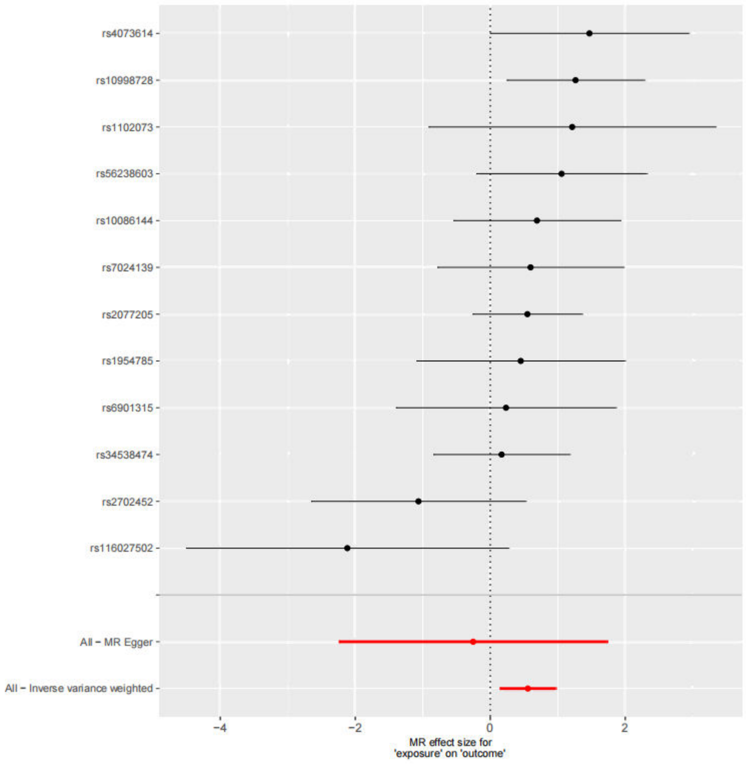

rs4073614
rs10998728
rs1102073
rs56238603
rs10086144
rs7024139
rs2077205
rs1954785
rs6901315
rs34538474
rs2702452
rs116027502
All − MR Egger
All − Inverse variance weighted
−4
−2
0
2
MR effect size for
'exposure' on 'outcome'

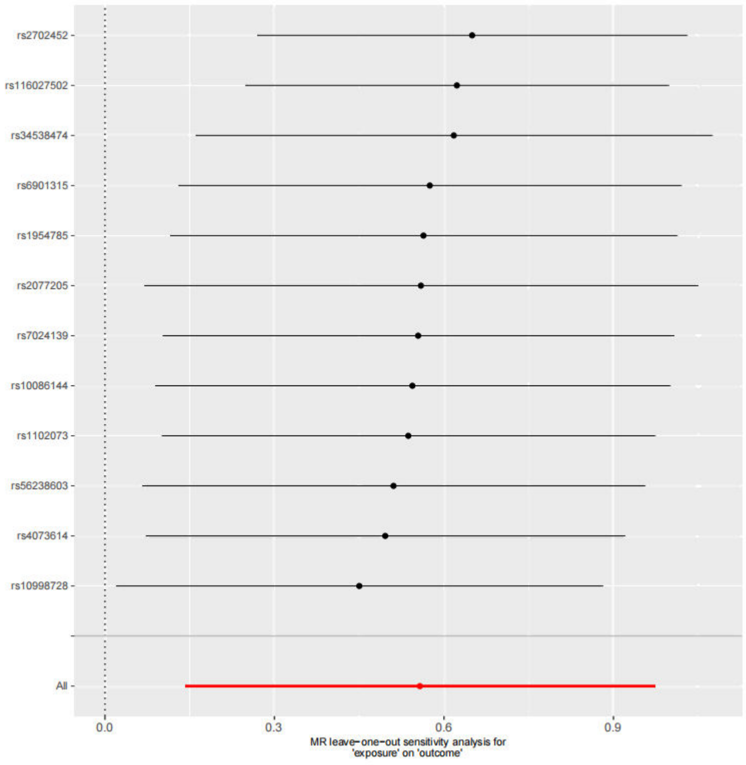

rs2702452
rs116027502
rs34538474
rs6901315
rs1954785
rs2077205
rs7024139
rs10086144
rs1102073
rs56238603
rs4073614
rs10998728
All
0.0
0.3
0.6
0.9
MR leave−one−out sensitivity analysis for
'exposure' on 'outcome'

GCST90257075

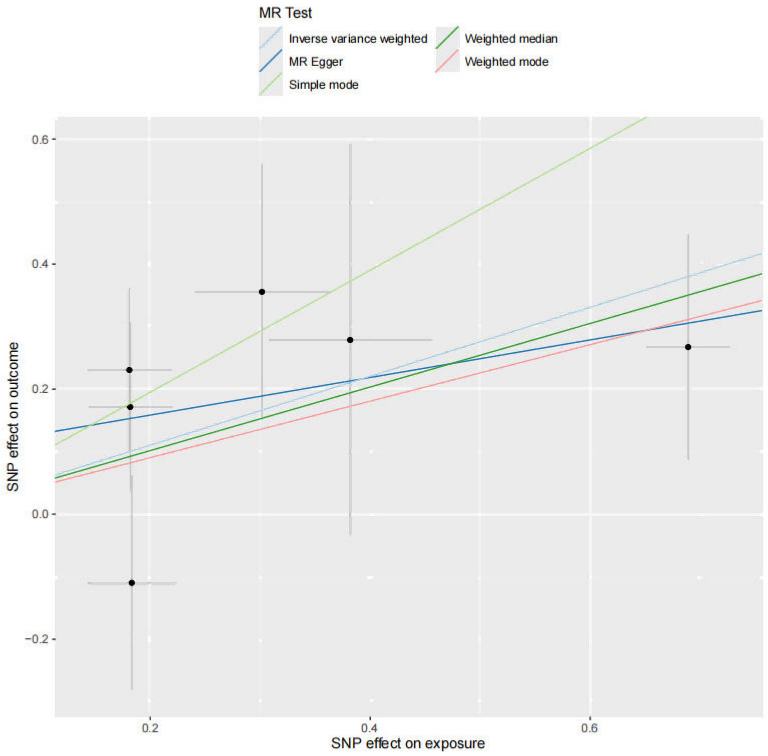

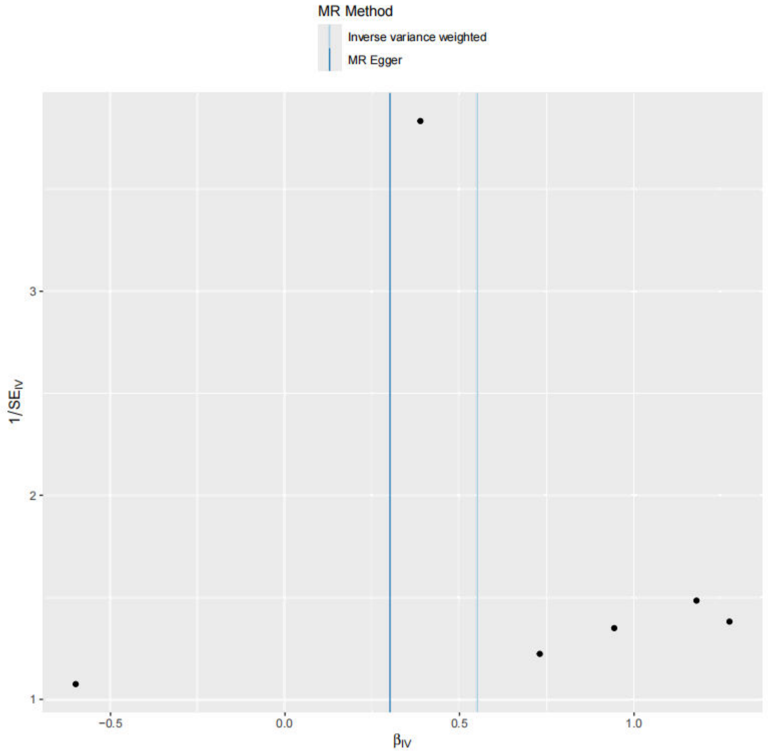

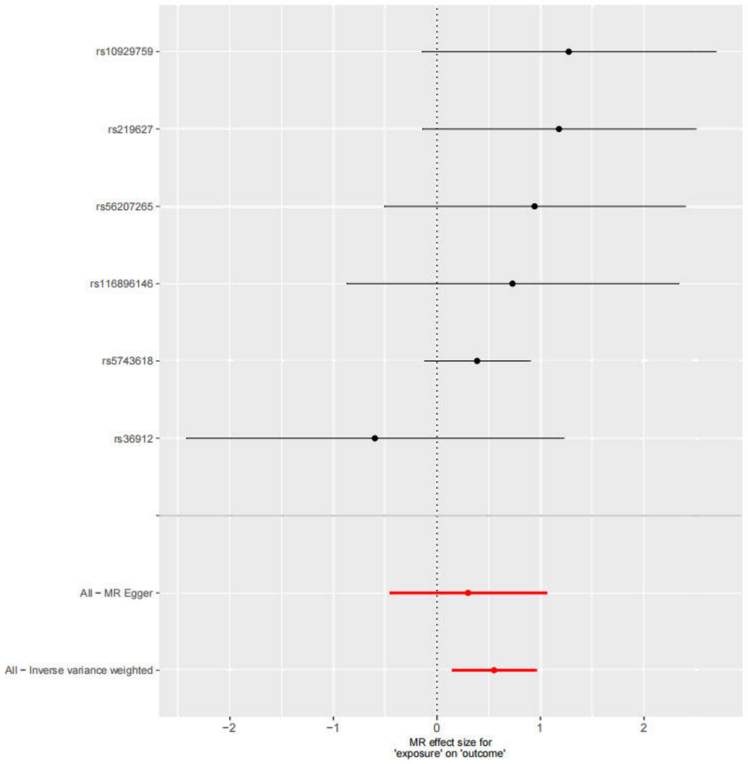
rs10929759
rs219627
rs56207265
rs116896146
rs5743618
rs36912
All − MR Egger
All − Inverse variance weighted
−2
−1
0
1
2
MR effect size for
'exposure' on 'outcome'

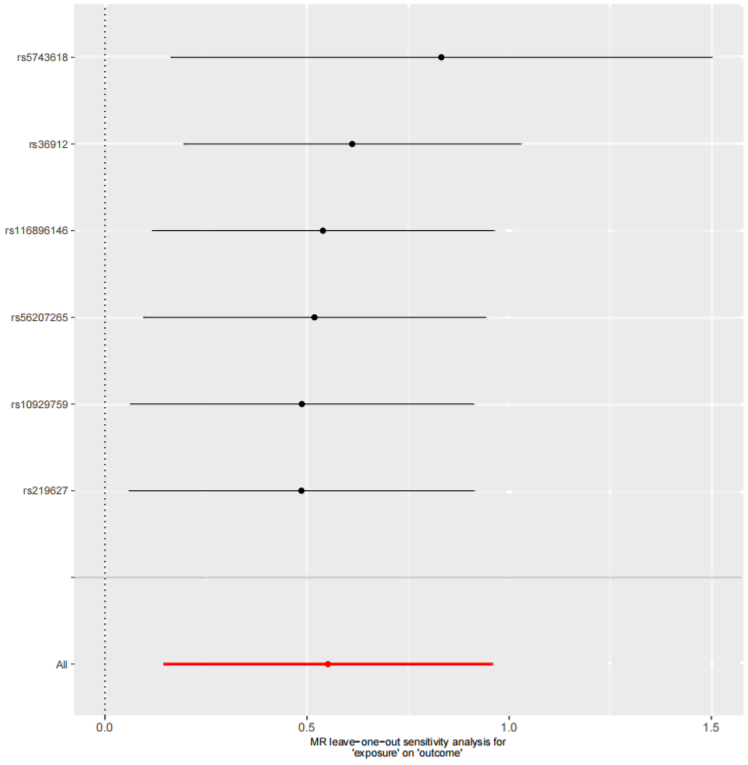
rs5743618
rs36912
rs116896146
rs56207265
rs10929759
rs219627
All
0.0
0.5
1.0
1.5
MR leave−one−out sensitivity analysis for
'exposure' on 'outcome'

GCST90257082

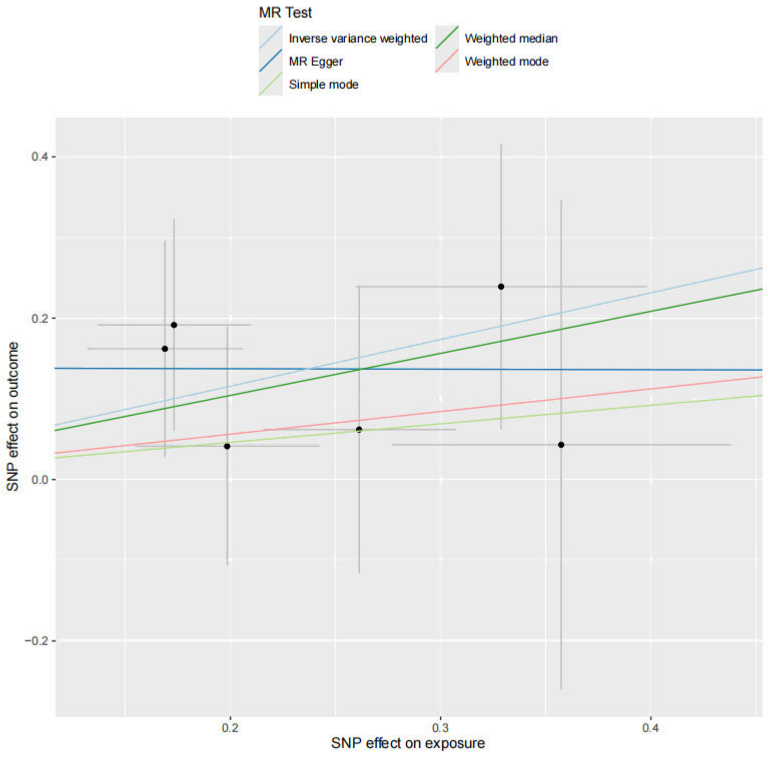

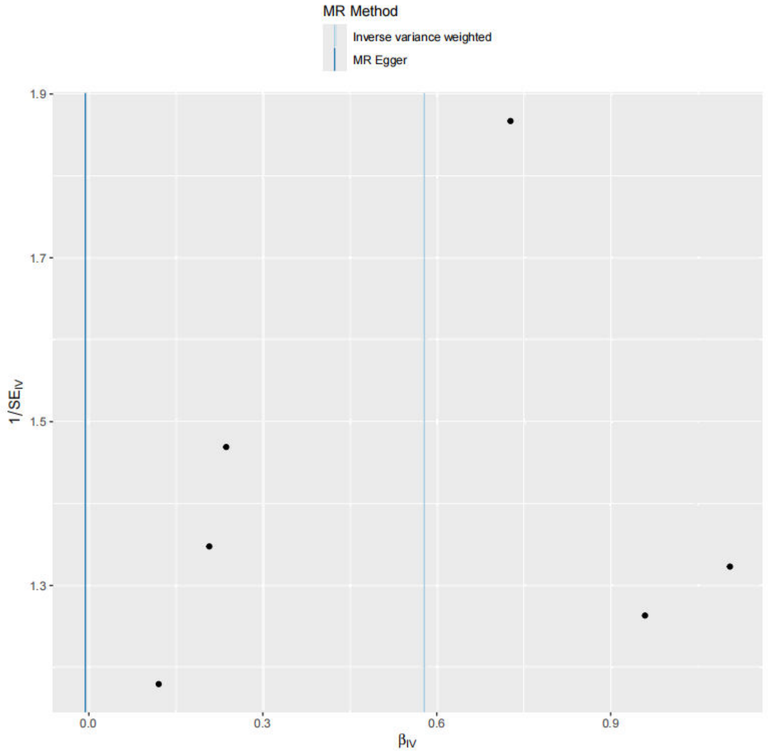

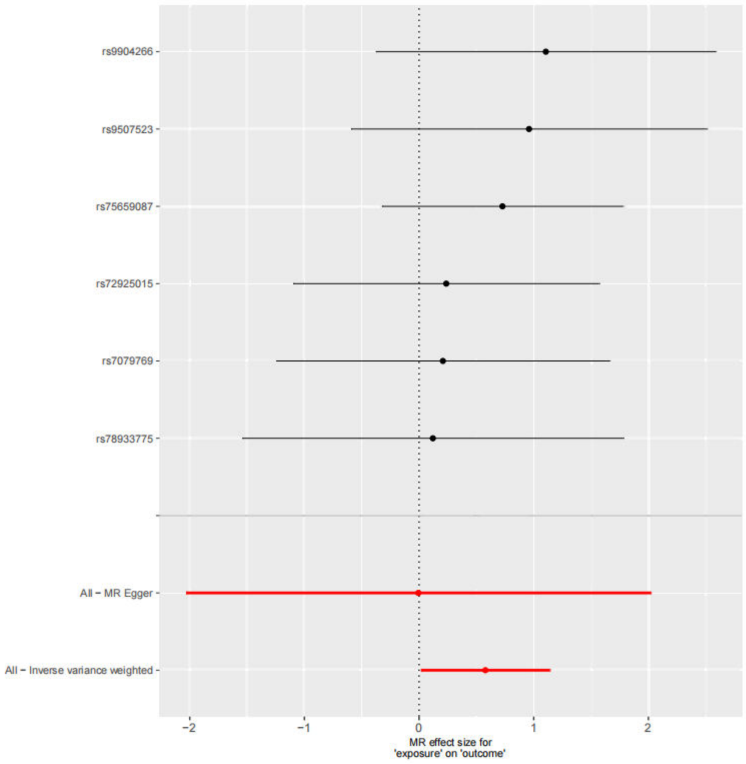
rs9904266
rs9507523
rs75659087
rs72925015
rs7079769
rs78933775
All − MR Egger
All − Inverse variance weighted
−2
−1
0
1
2
MR effect size for
'exposure' on 'outcome'

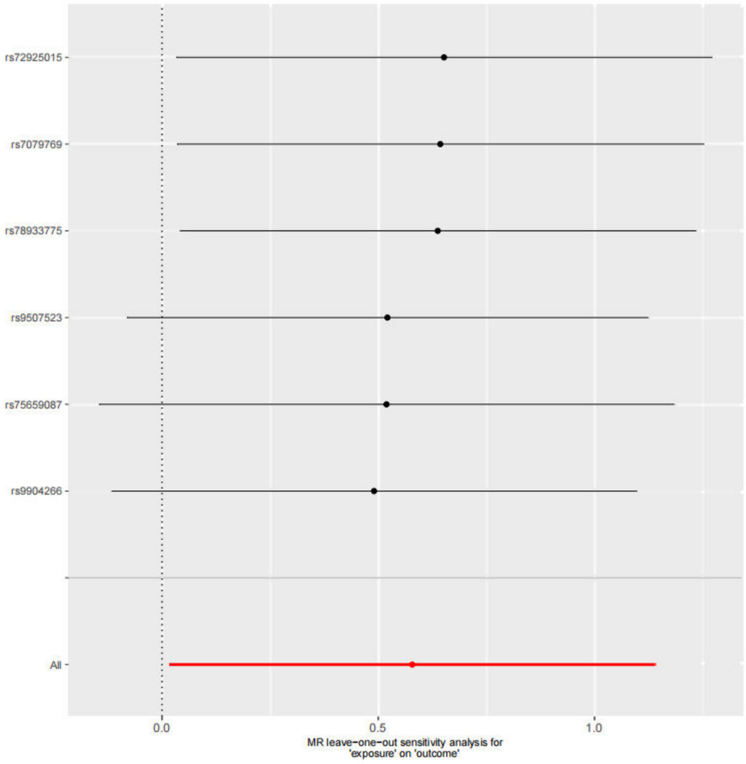
rs72925015
rs7079769
rs78933775
rs9507523
rs75659087
rs9904266
All
0.0
0.5
1.0
MR leave−one−out sensitivity analysis for
'exposure' on 'outcome'

GCST90257092

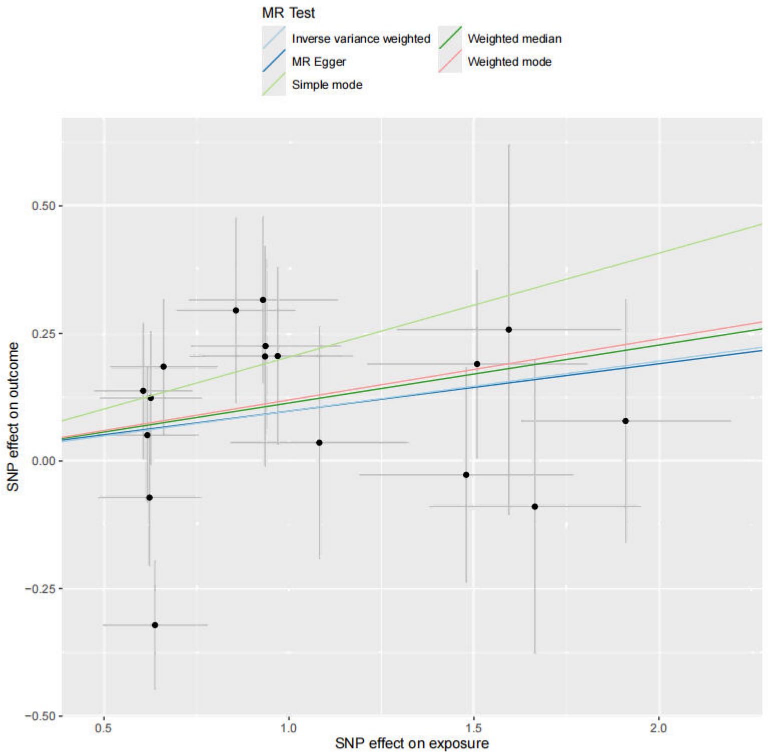

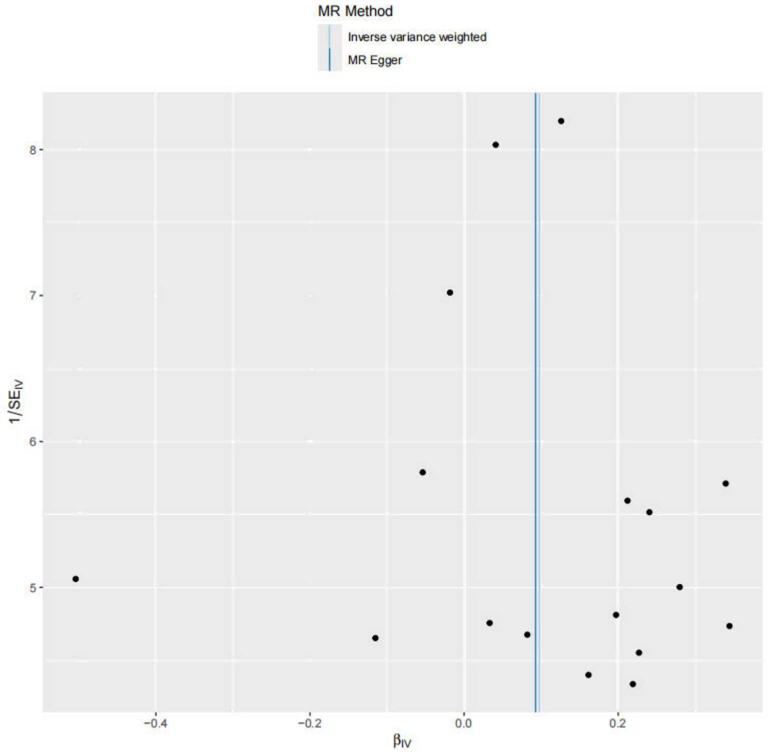

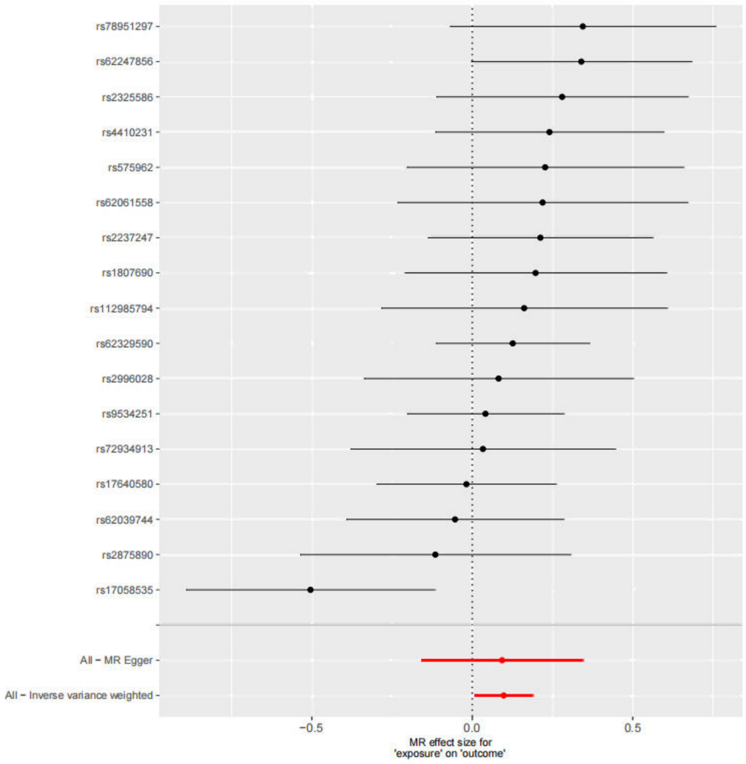

rs78951297
rs62247856
rs2325586
rs4410231
rs575962
rs62061558
rs2237247
rs1807690
rs112985794
rs62329590
rs2996028
rs9534251
rs72934913
rs17640580
rs62039744
rs2875890
rs17058535
All − MR Egger
All − Inverse variance weighted
−0.5
0.0
0.5
MR effect size for
'exposure' on 'outcome'

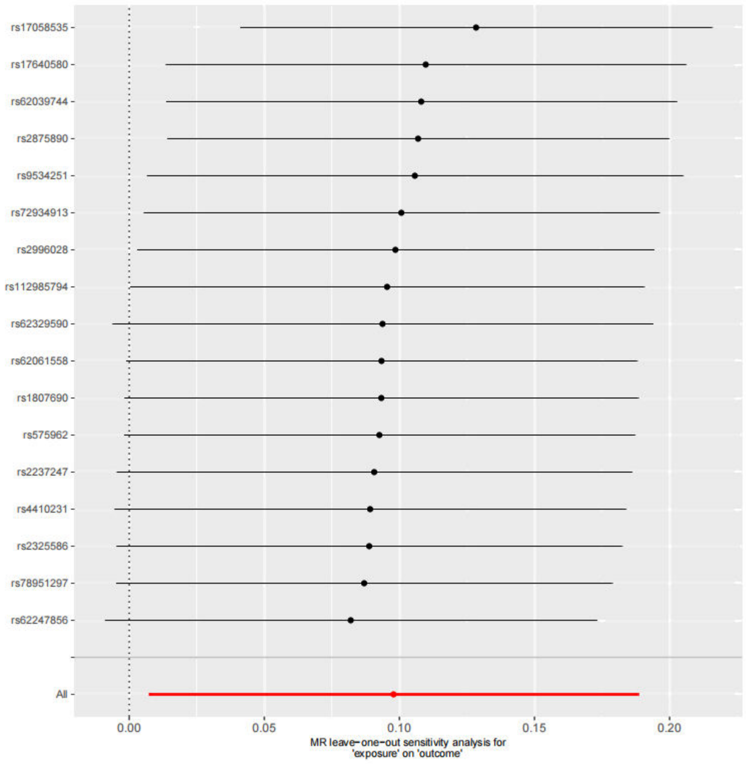

rs17058535
rs17640580
rs62039744
rs2875890
rs9534251
rs72934913
rs2996028
rs112985794
rs62329590
rs62061558
rs1807690
rs575962
rs2237247
rs4410231
rs2325586
rs78951297
rs62247856
All
0.00
0.05
0.10
0.15
0.20
MR leave−one−out sensitivity analysis for
'exposure' on 'outcome'

GCST90257101

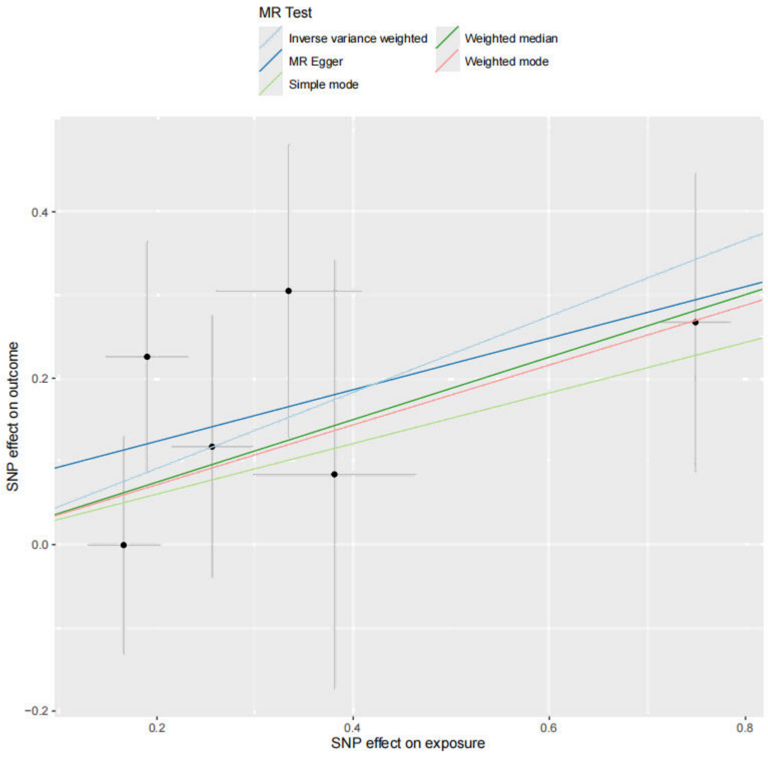

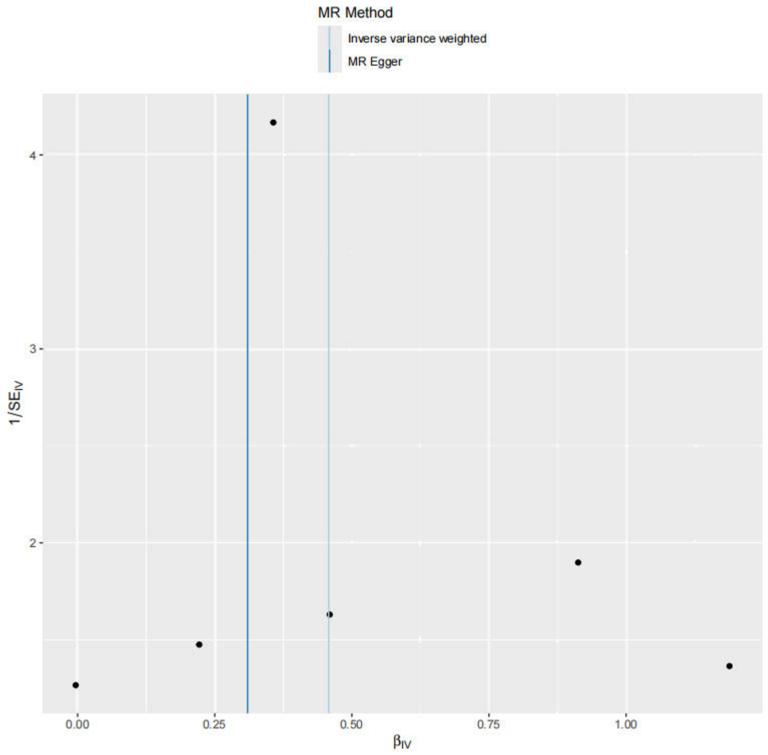

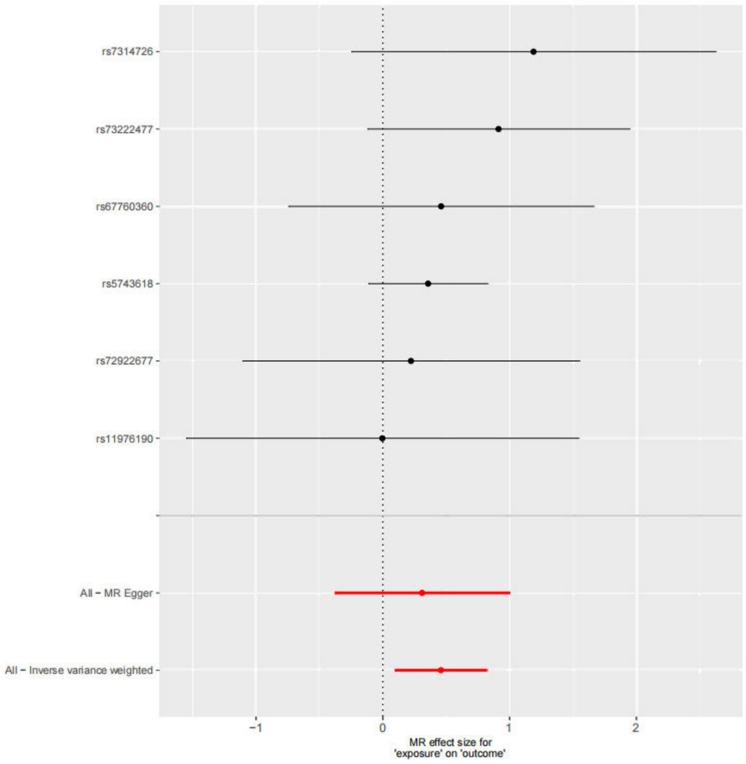

rs7314726
rs73222477
rs67760360
rs5743618
rs72922677
rs11976190
All − MR Egger
All − Inverse variance weighted
−1
0
1
2
MR effect size for
'exposure' on 'outcome'

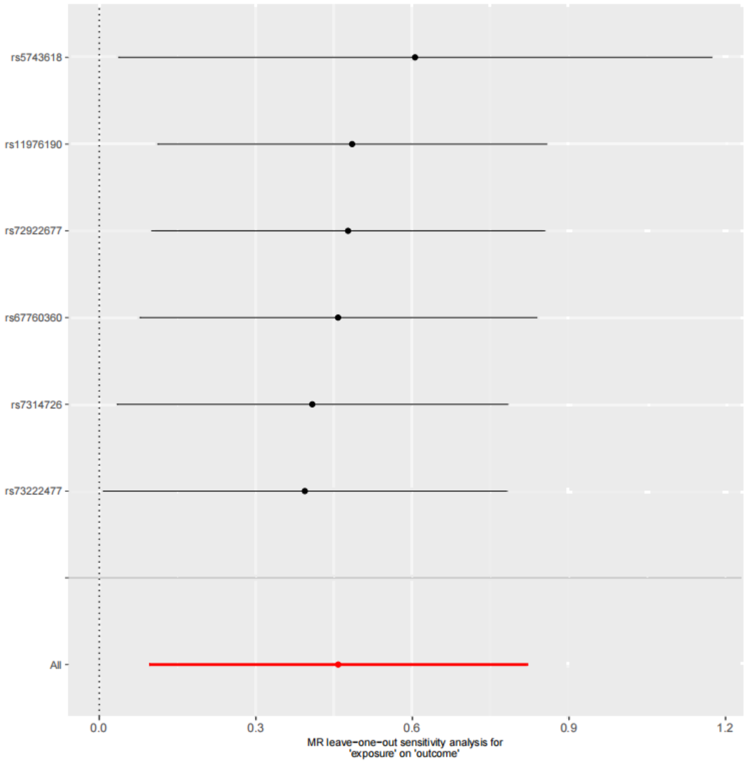

rs5743618
rs11976190
rs72922677
rs67760360
rs7314726
rs73222477
All
0.0
0.3
0.6
0.9
1.2
MR leave−one−out sensitivity analysis for
'exposure' on 'outcome'

GCST90257026

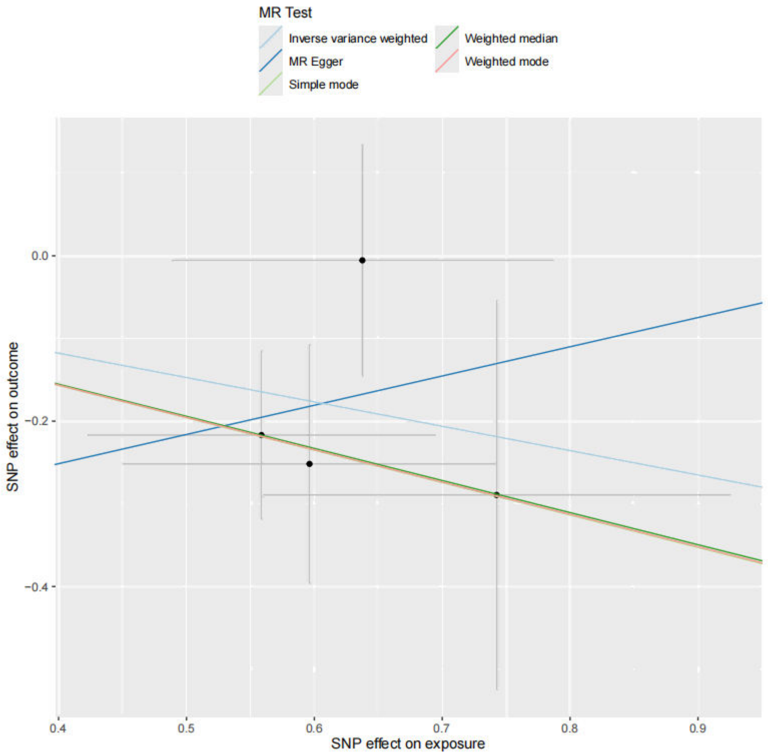

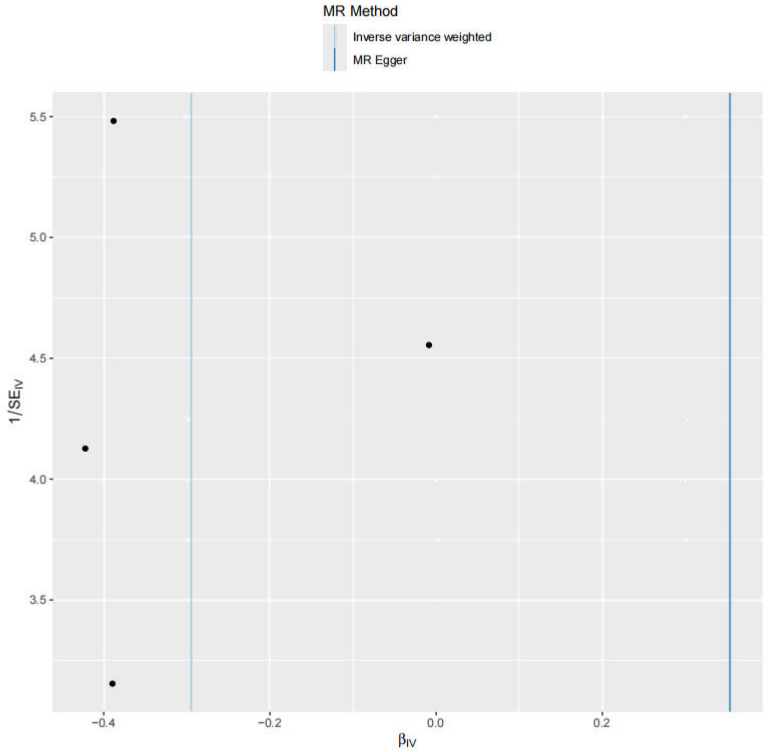

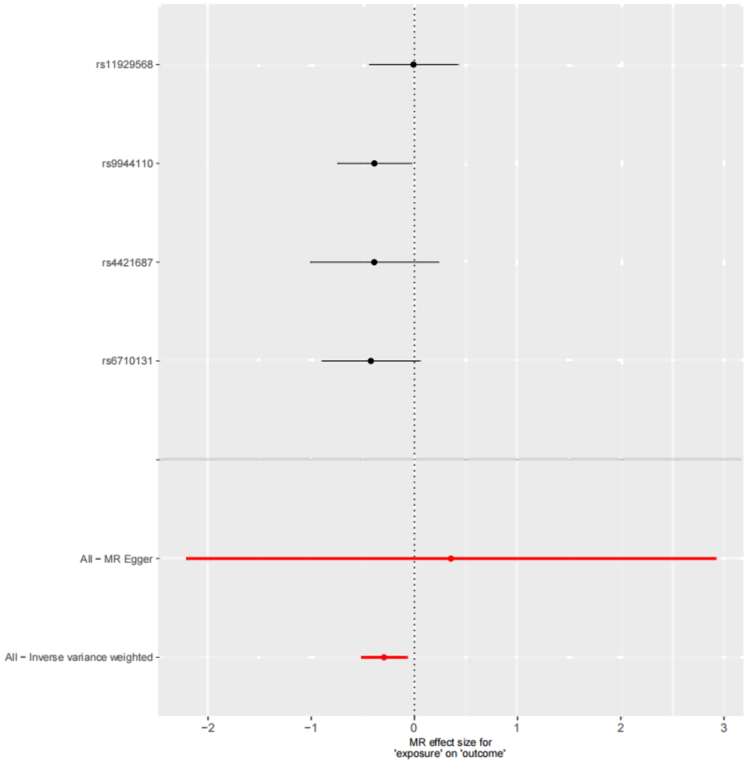
rs11929568
rs9944110
rs4421687
rs6710131
All − MR Egger
All − Inverse variance weighted
−2
−1
0
1
2
3
MR effect size for
'exposure' on 'outcome'

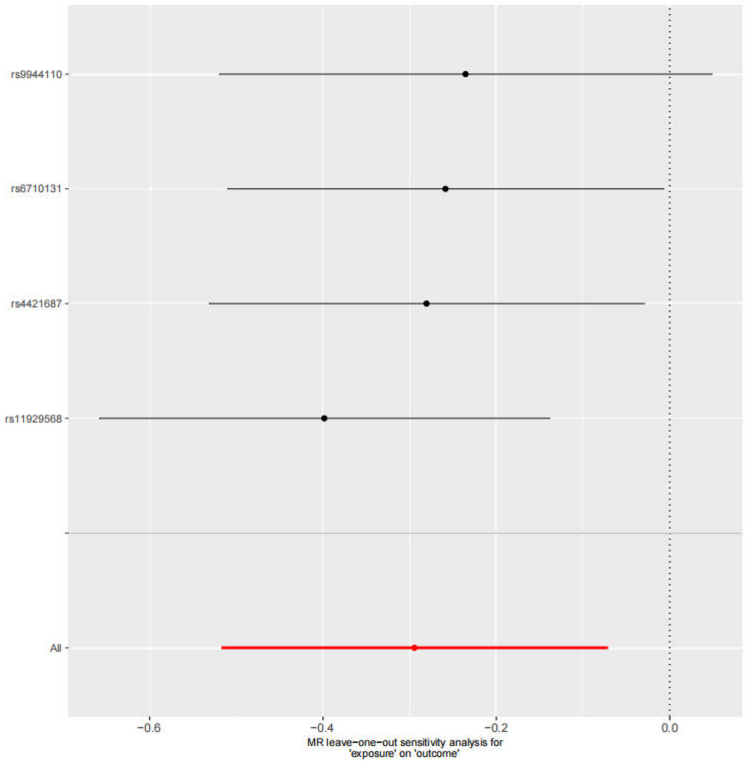
rs9944110
rs6710131
rs4421687
rs11929568
All
−0.6
−0.4
−0.2
0.0
MR leave−one−out sensitivity analysis for
'exposure' on 'outcome'

GCST90257033

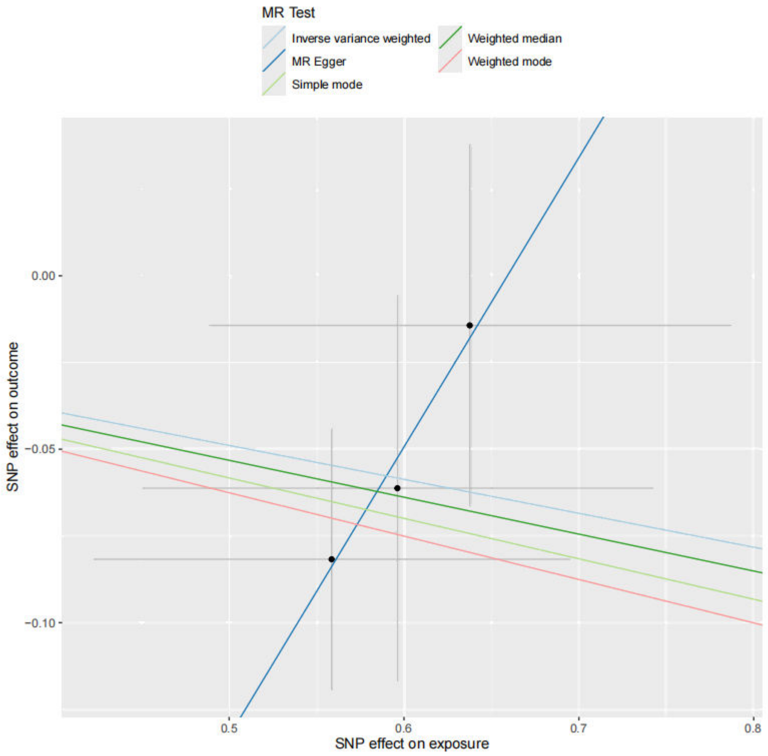

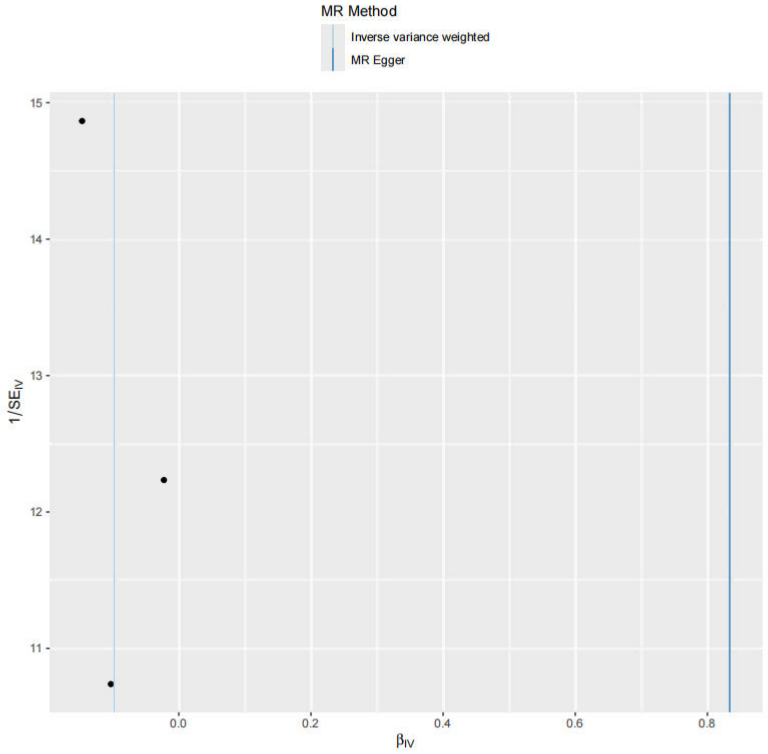

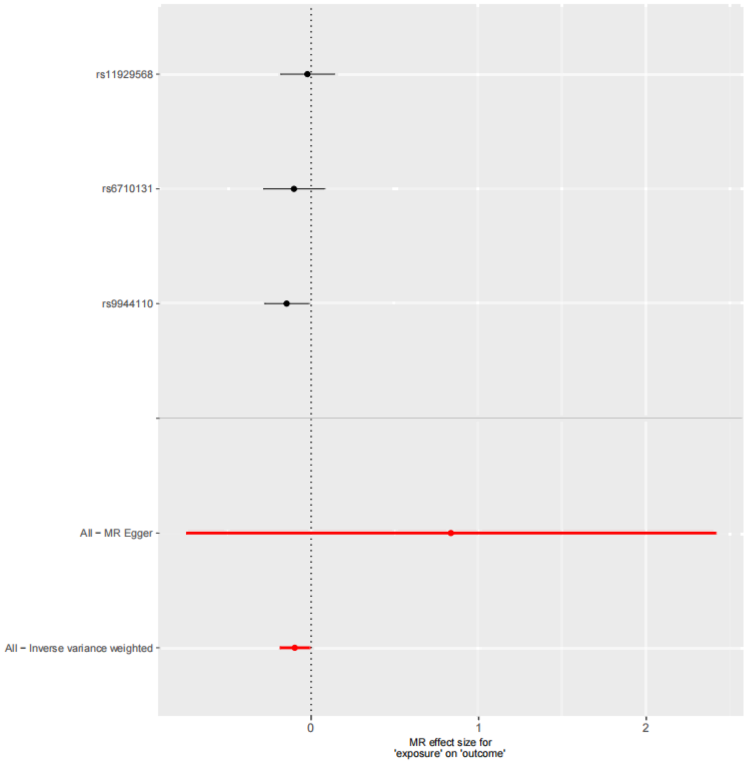

rs11929568
rs6710131
rs9944110
All – MR Egger
All – Inverse variance weighted
0
1
2
MR effect size for
'exposure' on 'outcome'

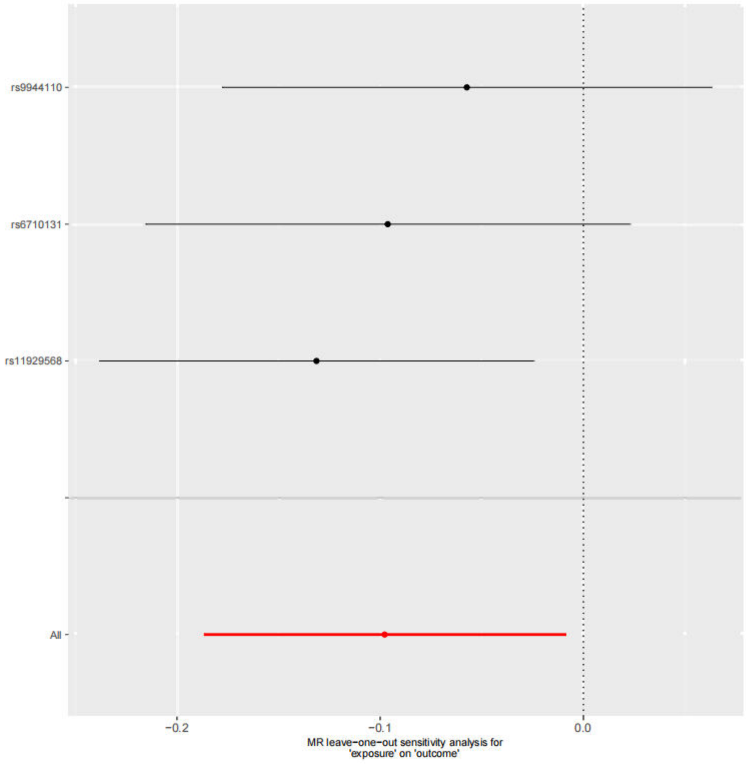

rs9944110
rs6710131
rs11929568
All
−0.2
−0.1
0.0
MR leave−one−out sensitivity analysis for
'exposure' on 'outcome'

GCST90257049

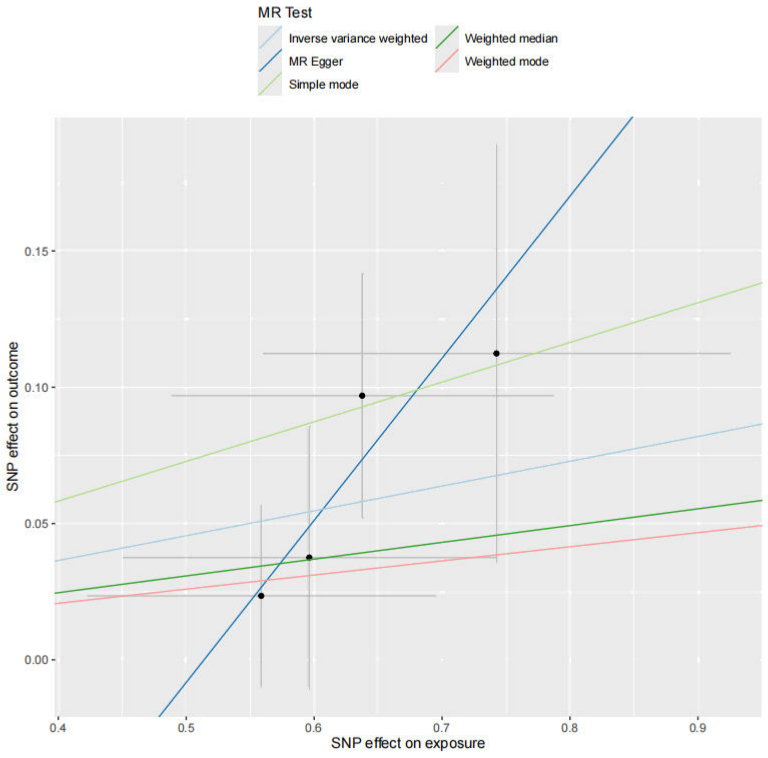

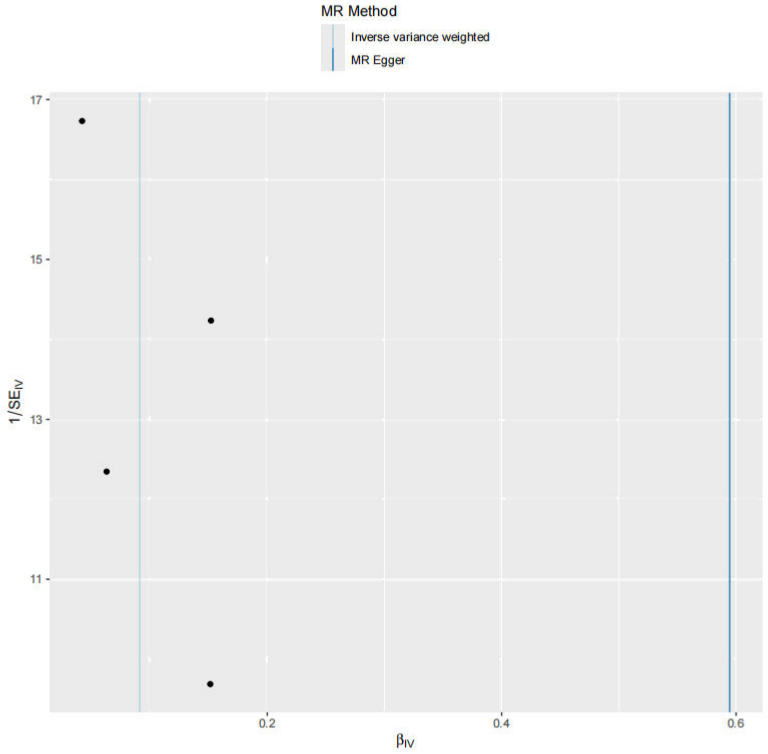

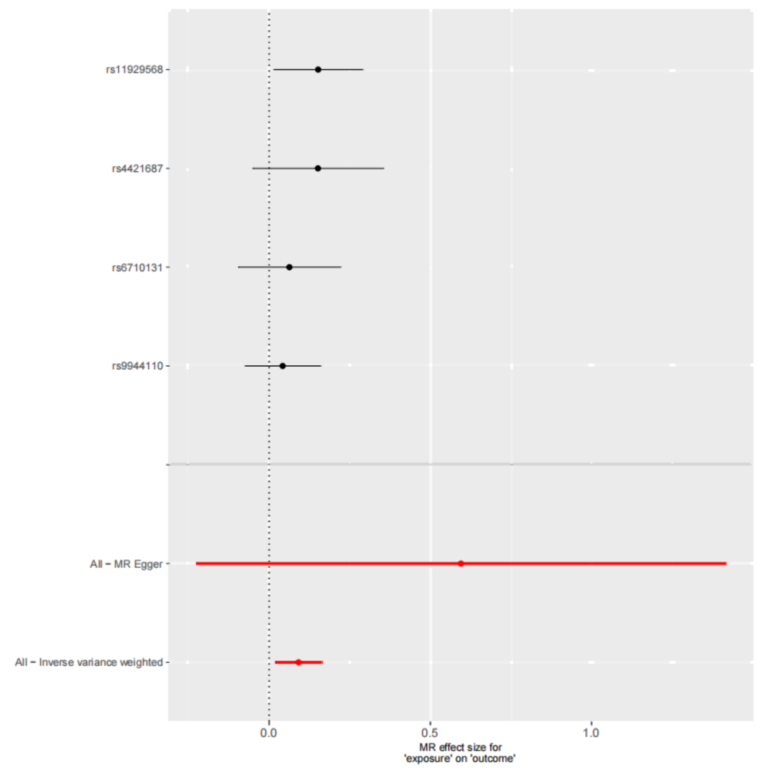
rs11929568
rs4421687
rs6710131
rs9944110
All − MR Egger
All − Inverse variance weighted
0.0
0.5
1.0
MR effect size for
'exposure' on 'outcome'

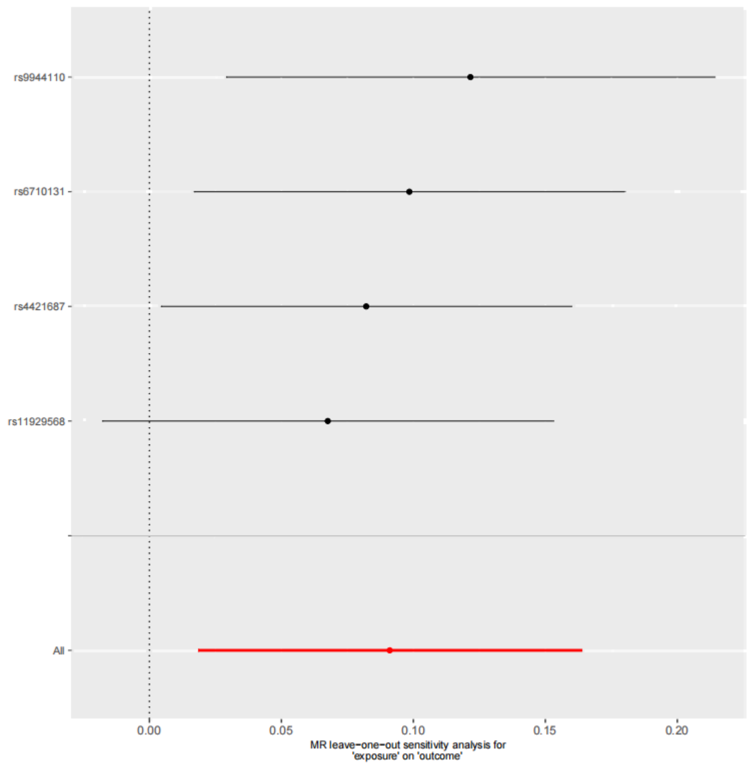
rs9944110
rs6710131
rs4421687
rs11929568
All
0.00
0.05
0.10
0.15
0.20
MR leave−one−out sensitivity analysis for
'exposure' on 'outcome'

GCST90257051

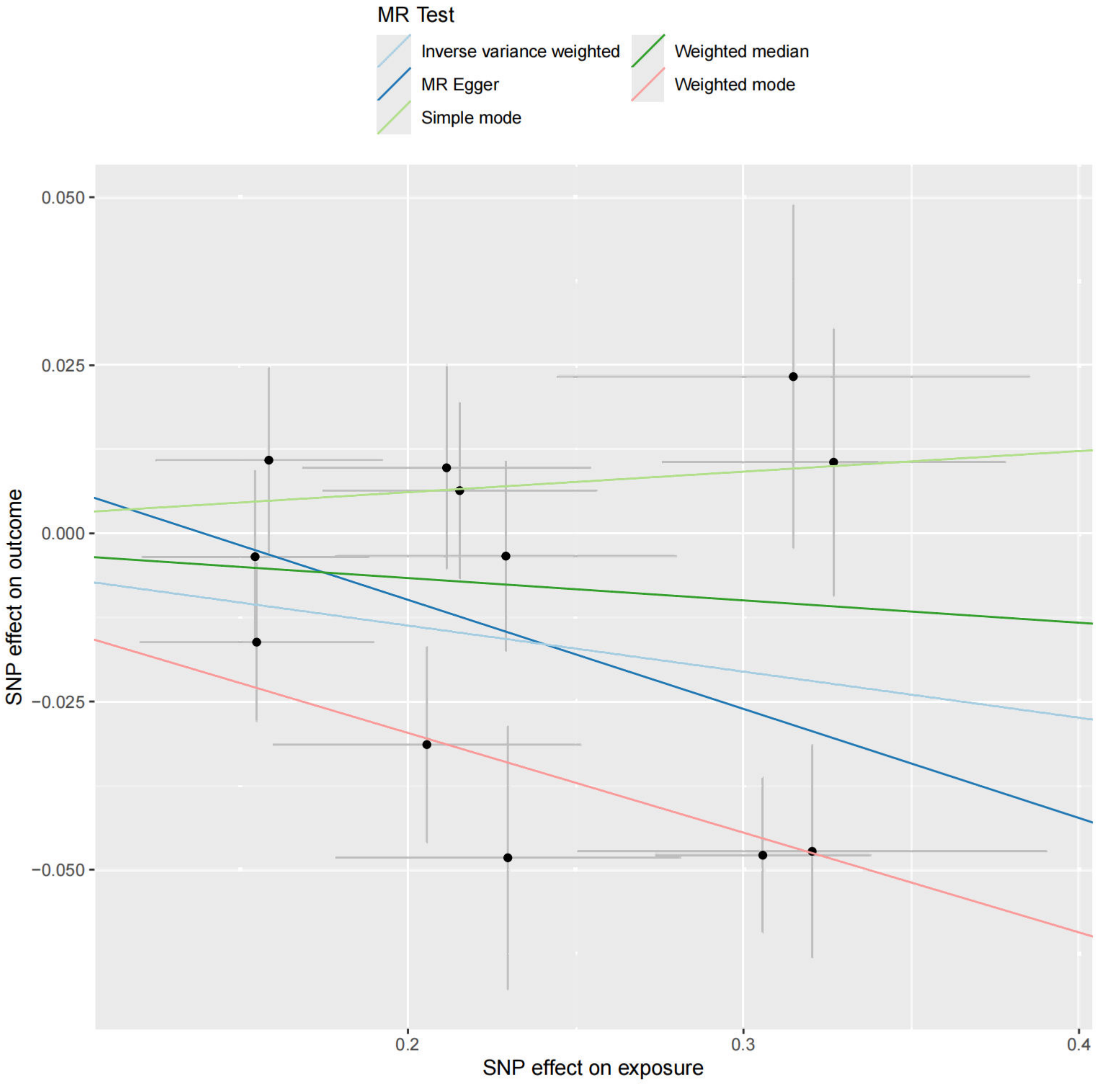

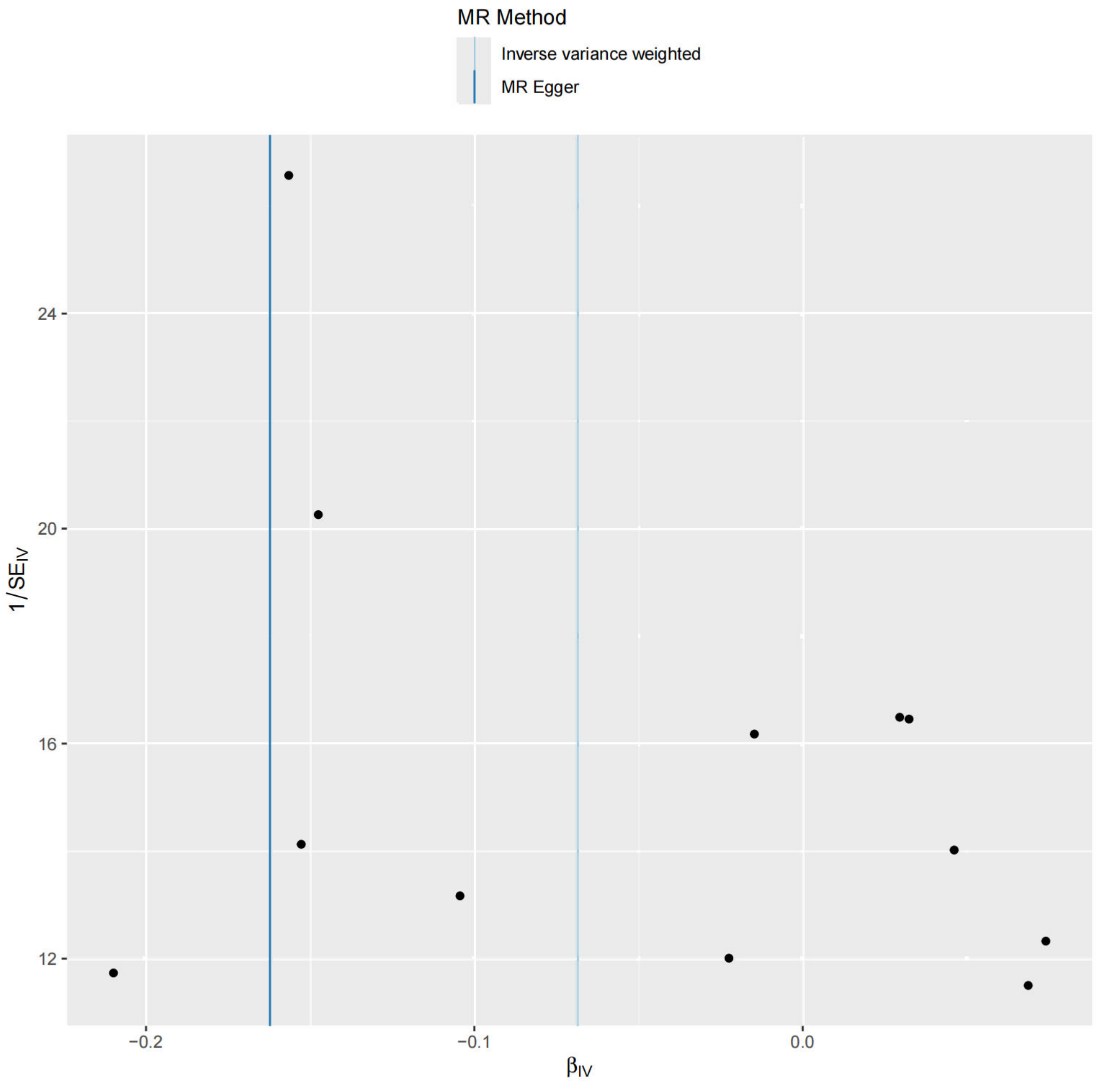

MR Method
Inverse variance weighted
MR Egger
24
20
16
12
1/SE$_{IV}$
−0.2
−0.1
0.0
β$_{IV}$

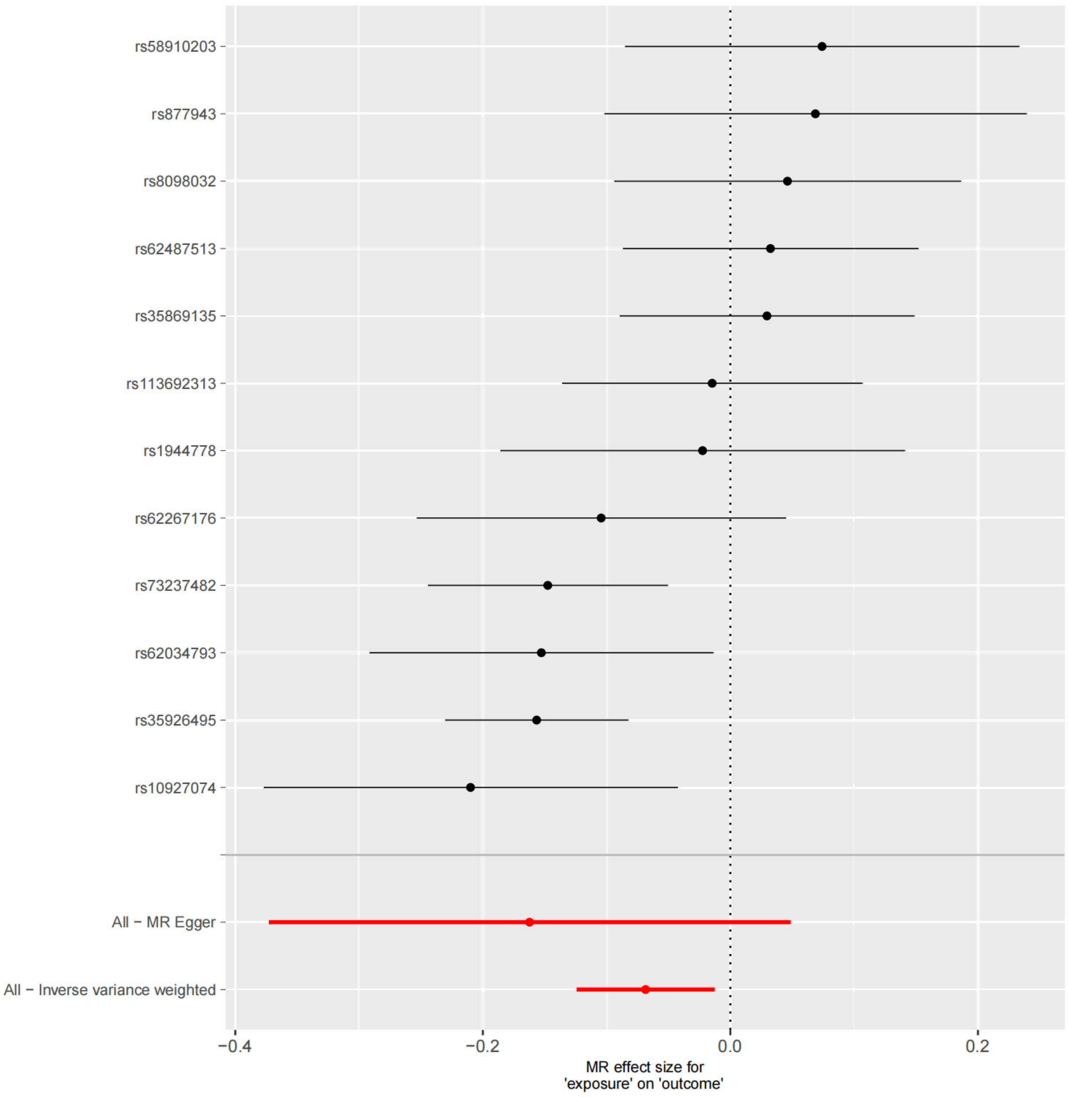

rs58910203
rs877943
rs8098032
rs62487513
rs35869135
rs113692313
rs1944778
rs62267176
rs73237482
rs62034793
rs35926495
rs10927074
All − MR Egger
All − Inverse variance weighted
−0.4
−0.2
0.0
0.2
MR effect size for
'exposure' on 'outcome'

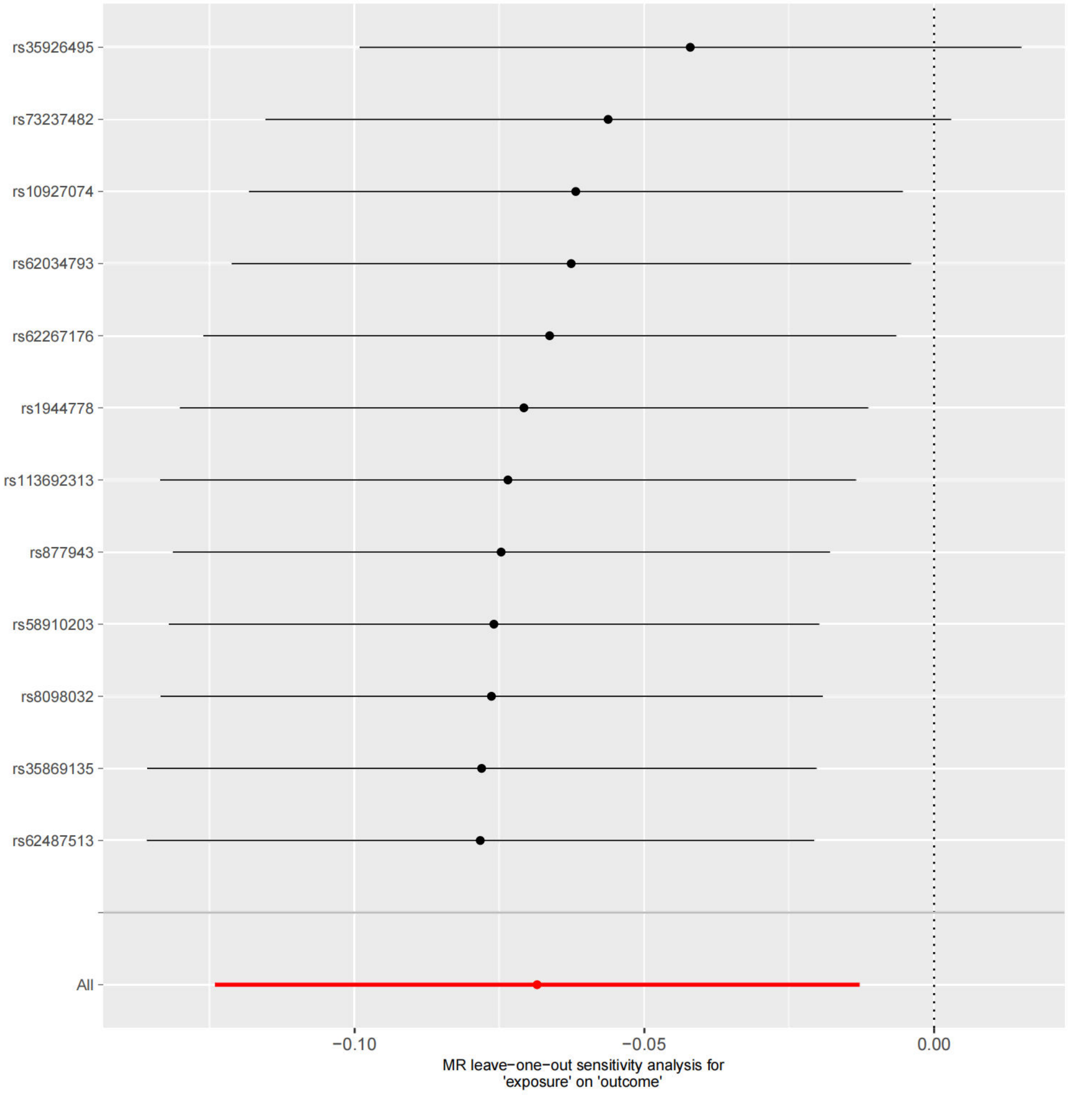

rs35926495
rs73237482
rs10927074
rs62034793
rs62267176
rs1944778
rs113692313
rs877943
rs58910203
rs8098032
rs35869135
rs62487513
All
−0.10
−0.05
0.00
MR leave−one−out sensitivity analysis for
'exposure' on 'outcome'

GCST90257053

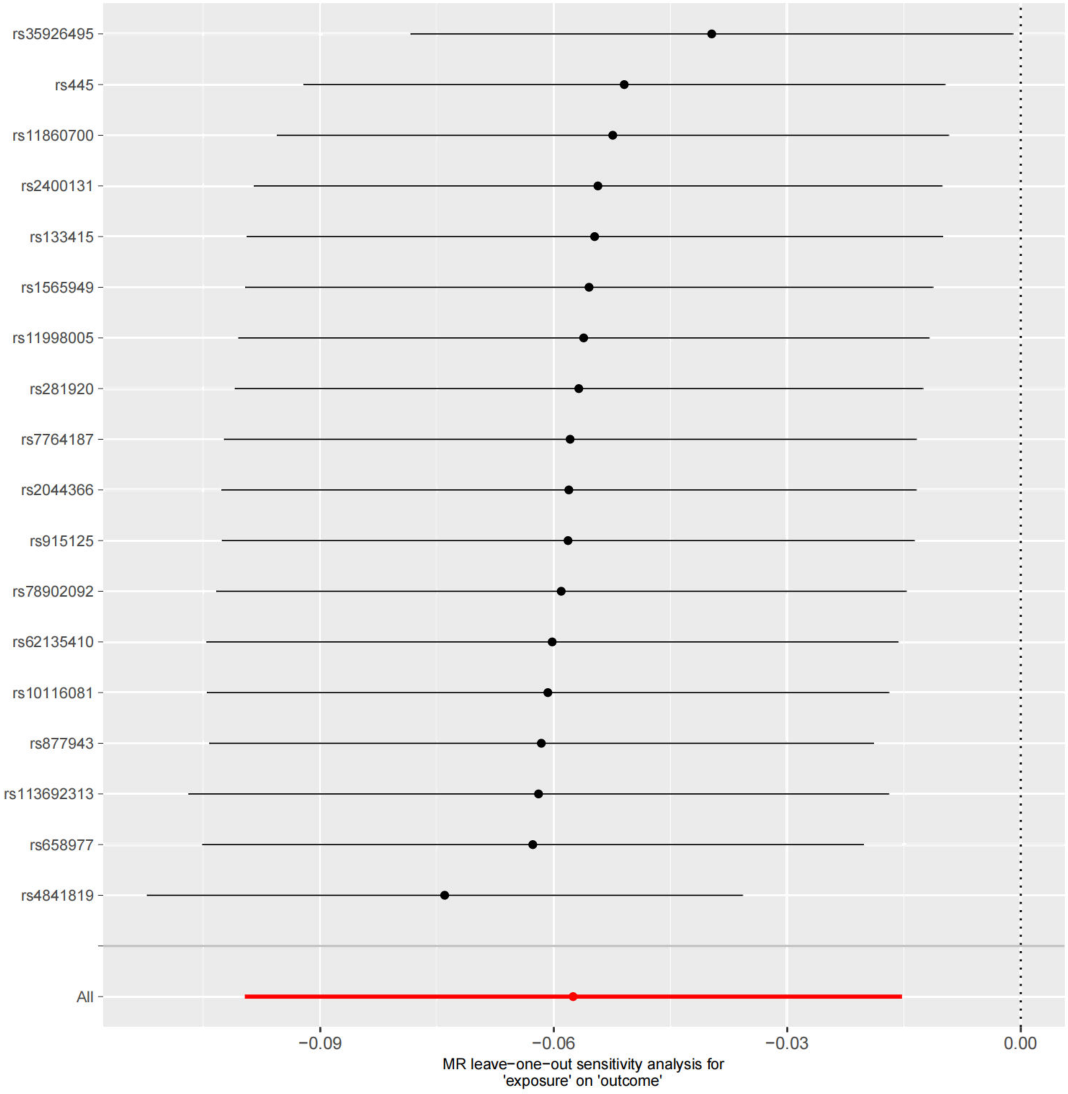

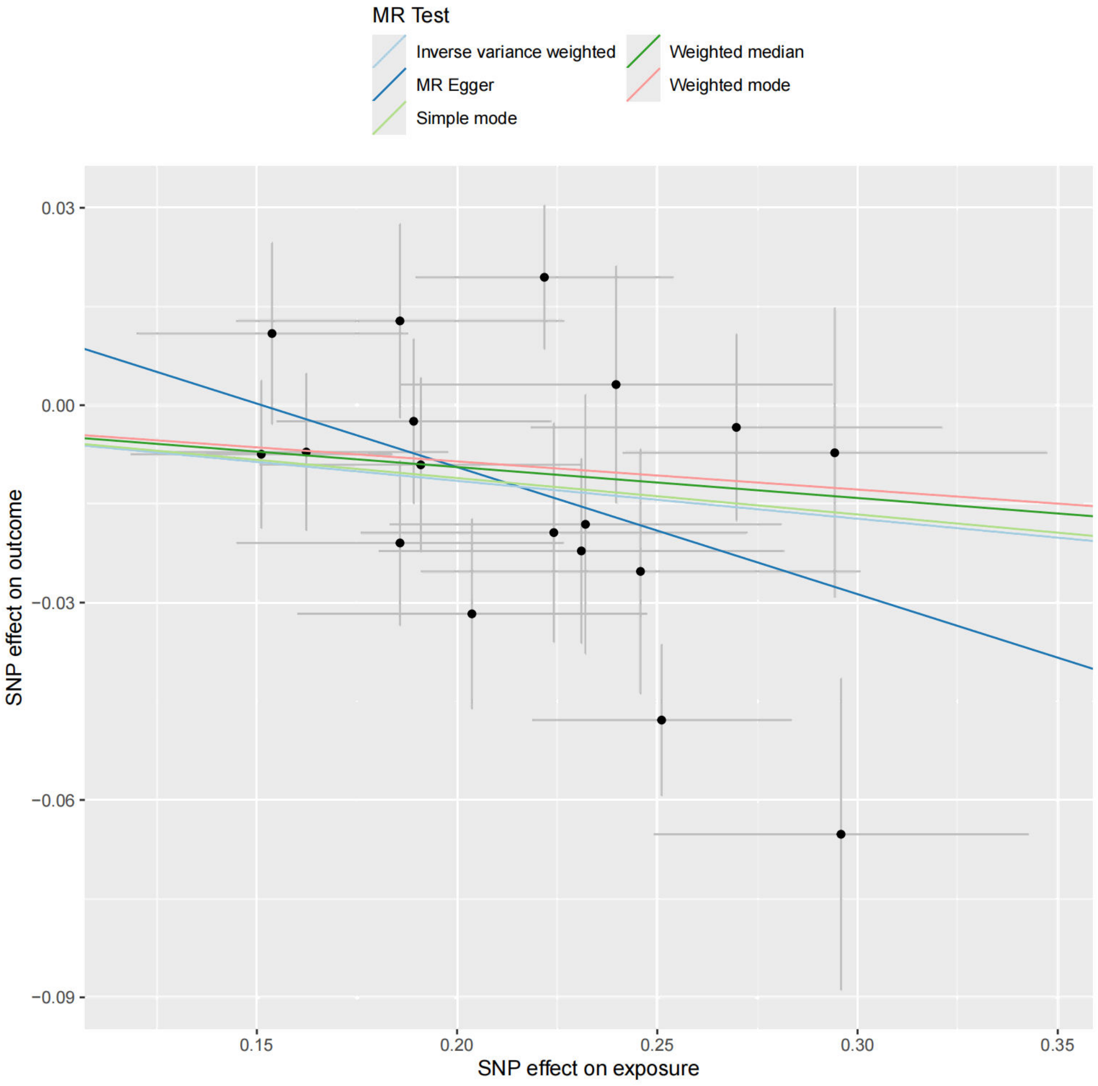
MR Test
Inverse variance weighted
MR Egger
Simple mode
Weighted median
Weighted mode
0.03
0.00
−0.03
−0.06
−0.09
SNP effect on outcome
0.15
0.20
0.25
0.30
0.35
SNP effect on exposure

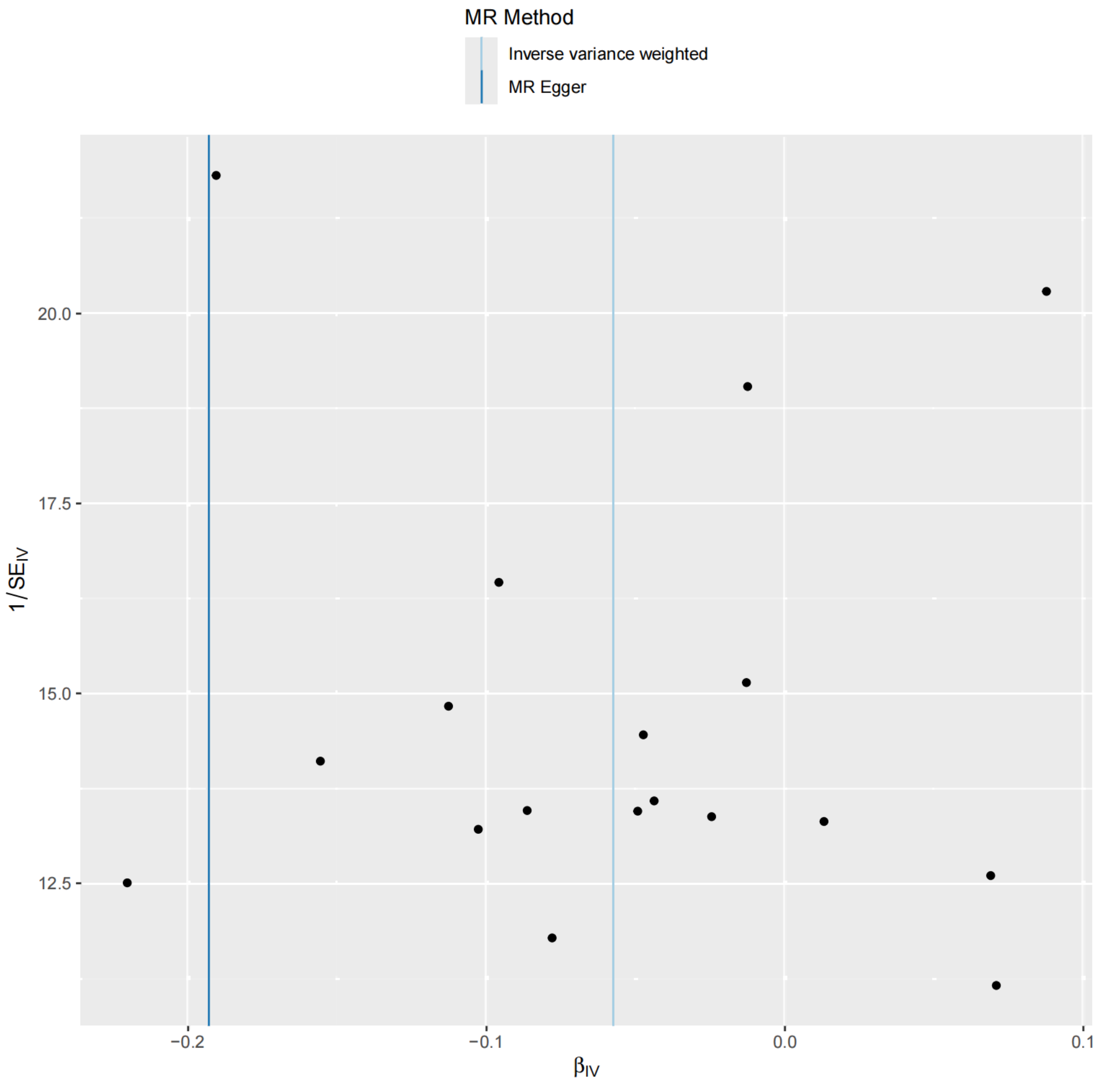

MR Method
Inverse variance weighted
MR Egger
20.0
17.5
15.0
12.5
1/SE_IV
−0.2
−0.1
0.0
0.1
β_IV

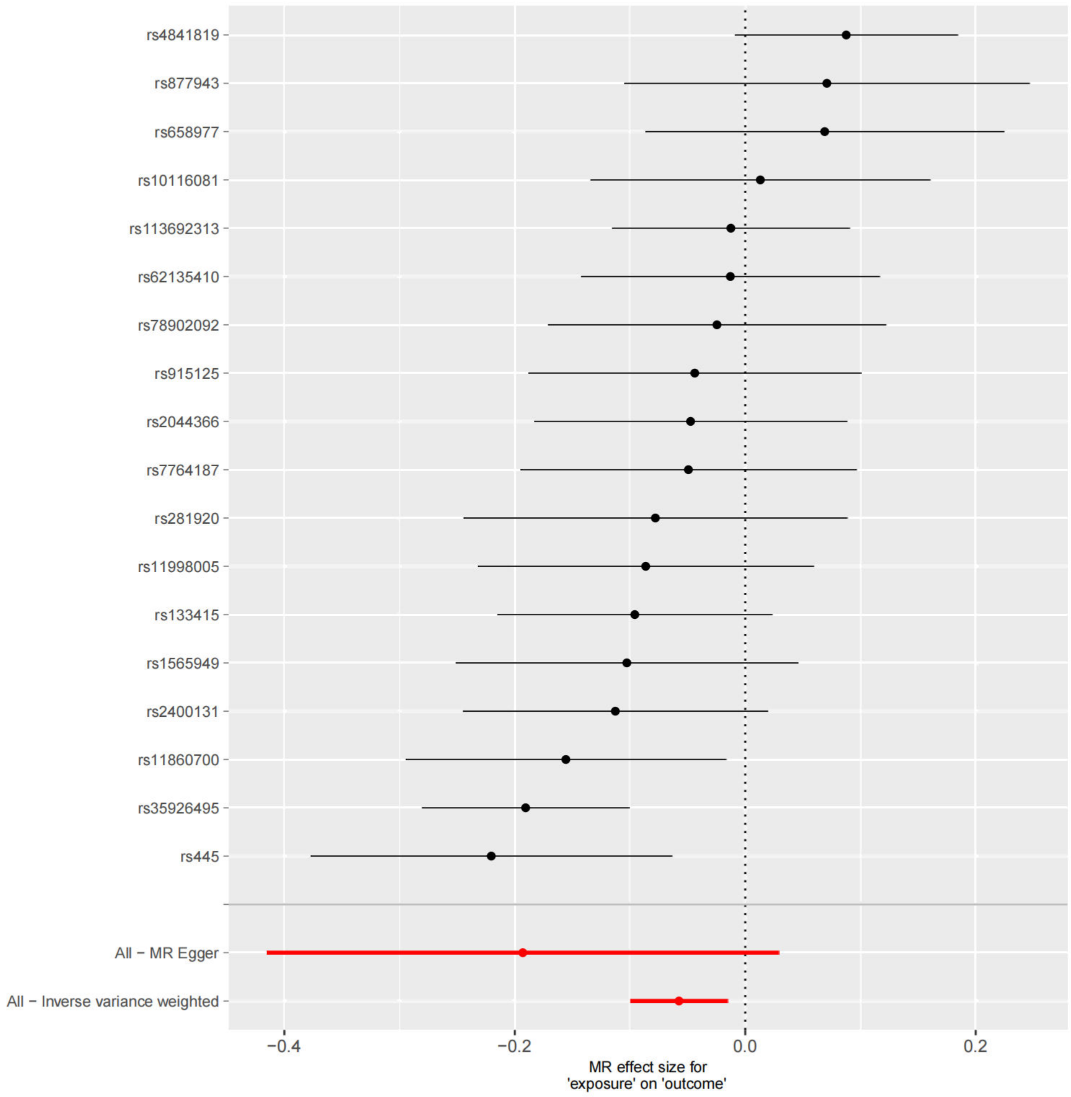

rs4841819
rs877943
rs658977
rs10116081
rs113692313
rs62135410
rs78902092
rs915125
rs2044366
rs7764187
rs281920
rs11998005
rs133415
rs1565949
rs2400131
rs11860700
rs35926495
rs445
All − MR Egger
All − Inverse variance weighted
−0.4
−0.2
0.0
0.2
MR effect size for
'exposure' on 'outcome'

GCST90257054

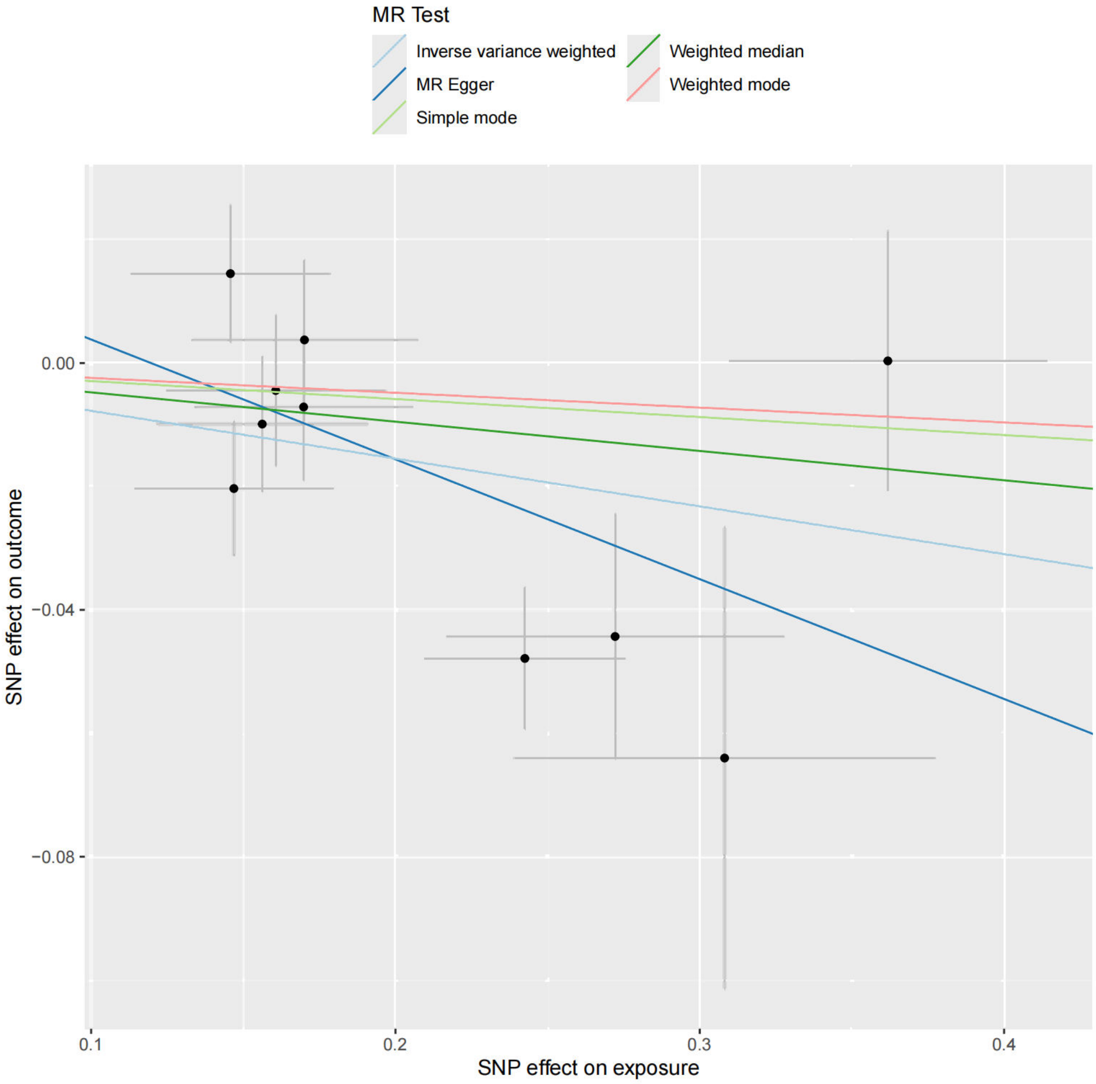

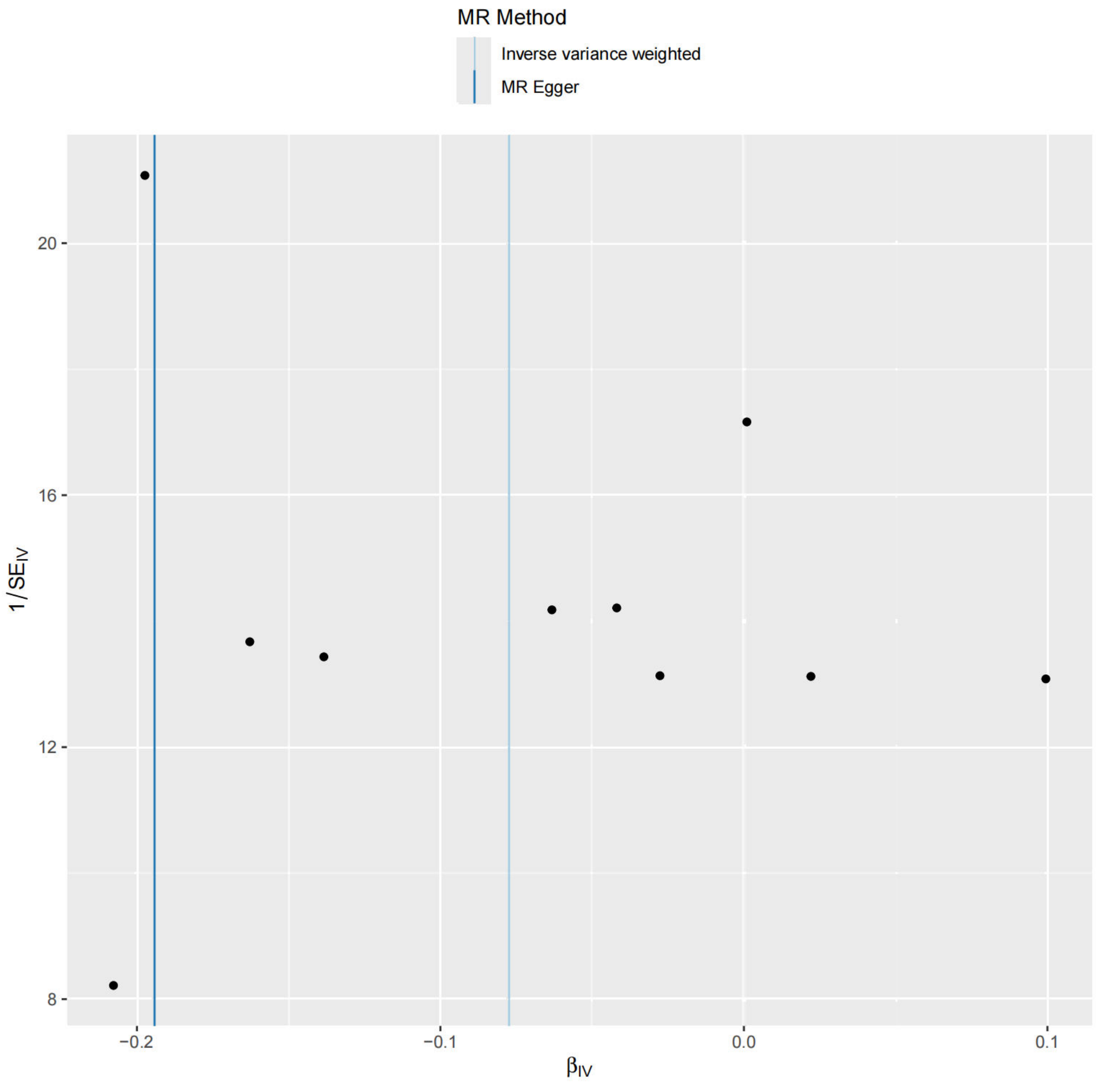

MR Method
Inverse variance weighted
MR Egger
20
16
12
8
1/SE_IV
−0.2
−0.1
0.0
0.1
β_IV

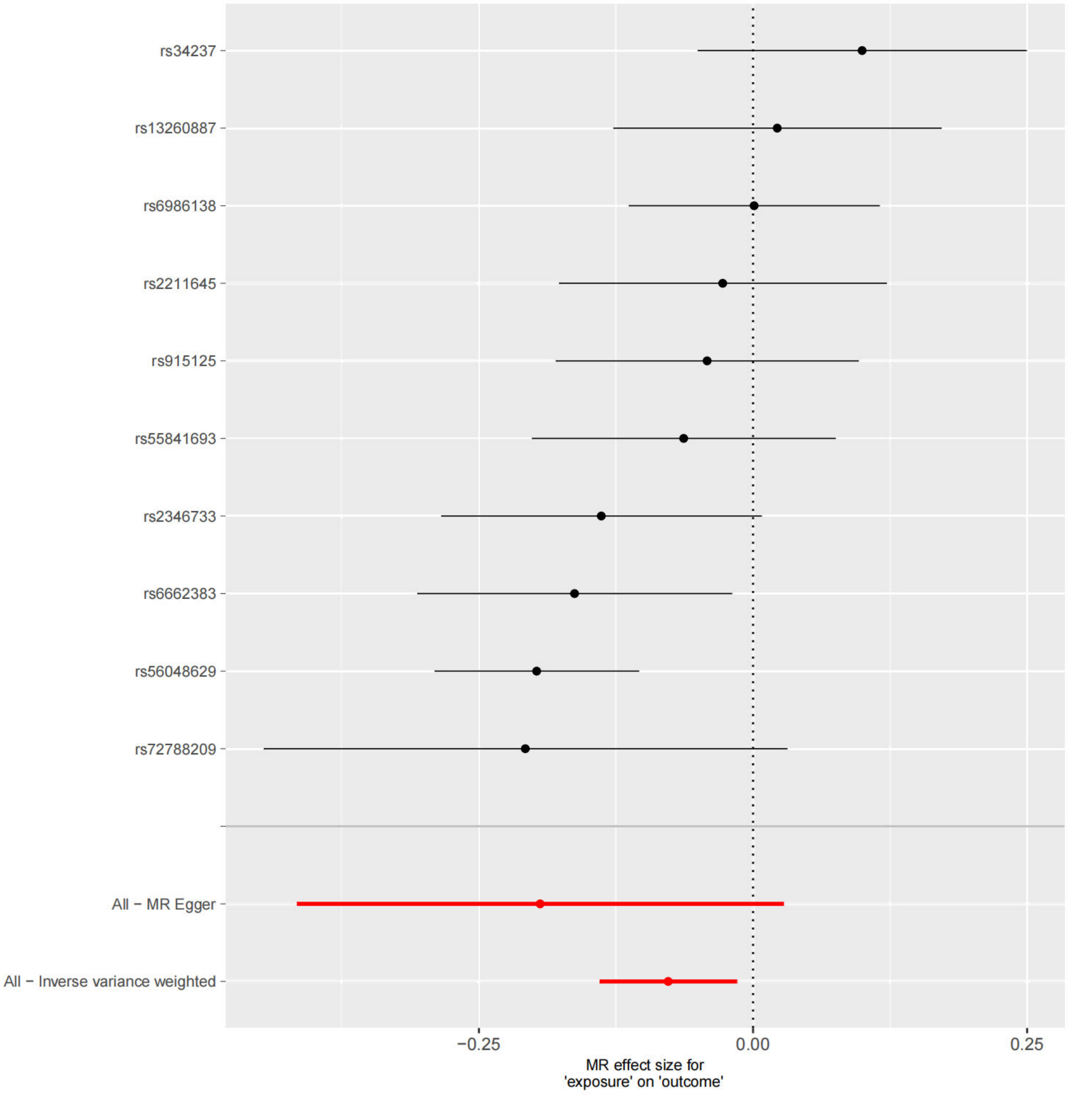

rs34237
rs13260887
rs6986138
rs2211645
rs915125
rs55841693
rs2346733
rs6662383
rs56048629
rs72788209
All − MR Egger
All − Inverse variance weighted
−0.25
0.00
0.25
MR effect size for
'exposure' on 'outcome'

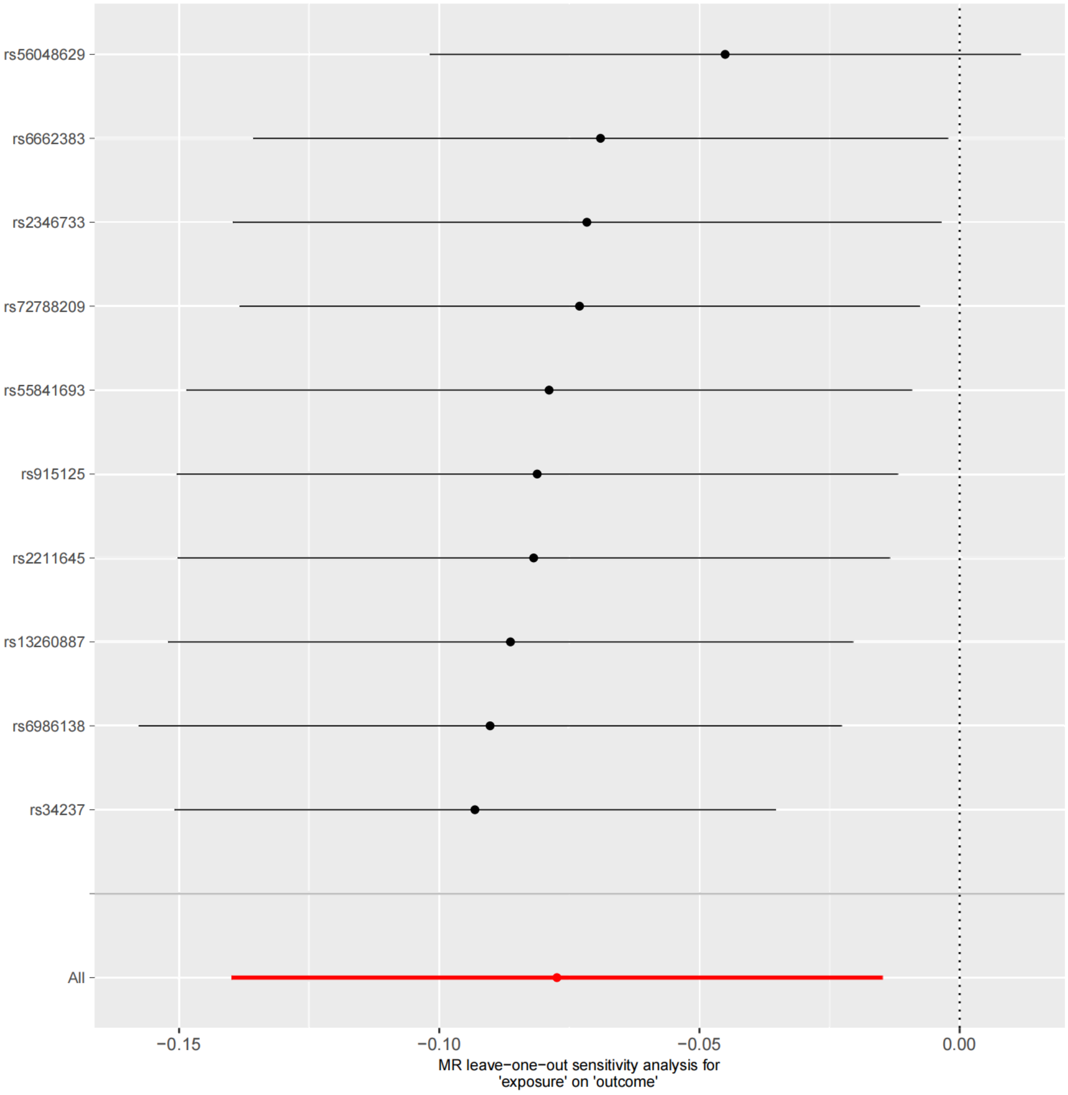
rs56048629
rs6662383
rs2346733
rs72788209
rs55841693
rs915125
rs2211645
rs13260887
rs6986138
rs34237
All
−0.15
−0.10
−0.05
0.00
MR leave−one−out sensitivity analysis for
'exposure' on 'outcome'

GCST90257077

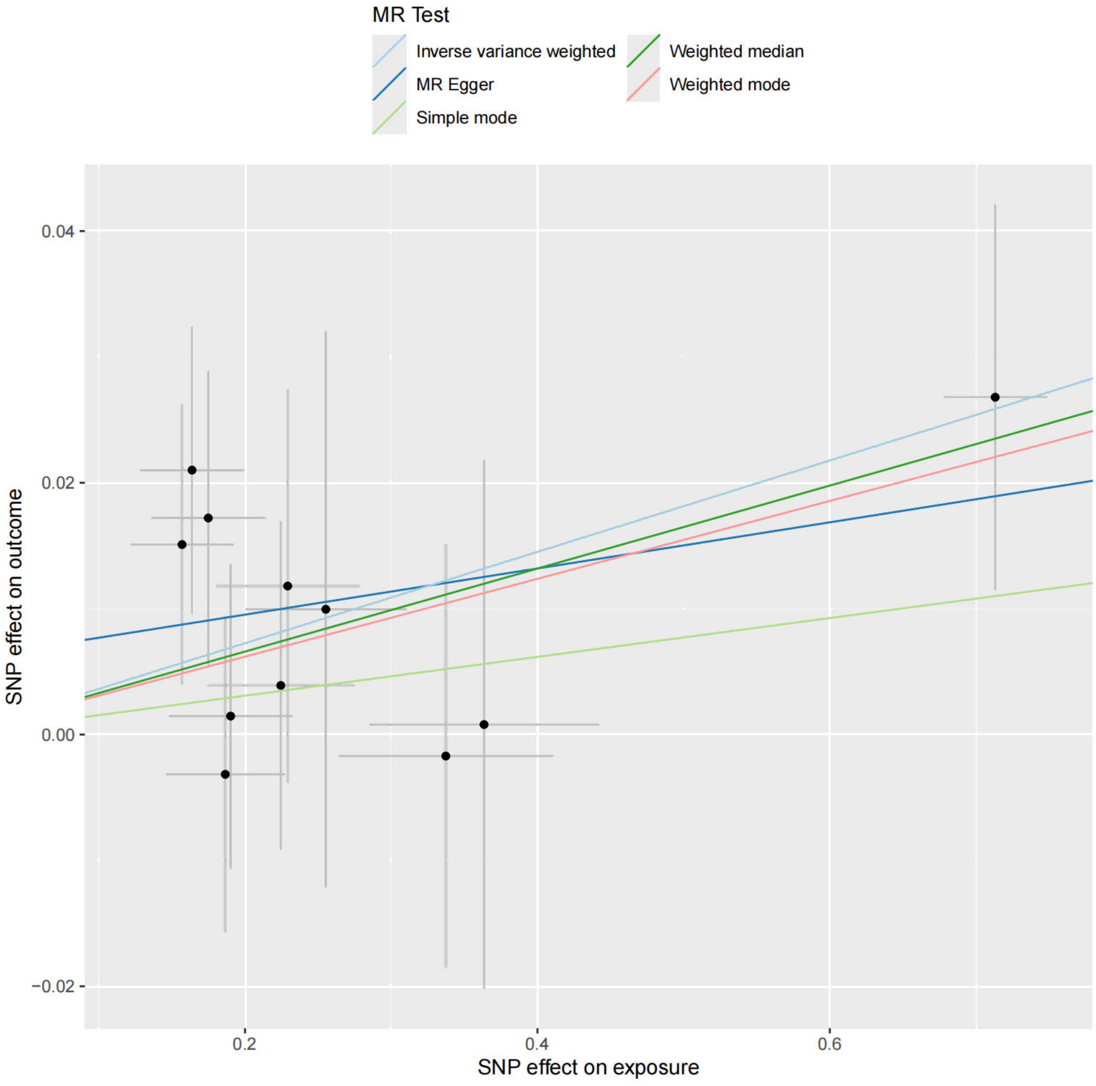

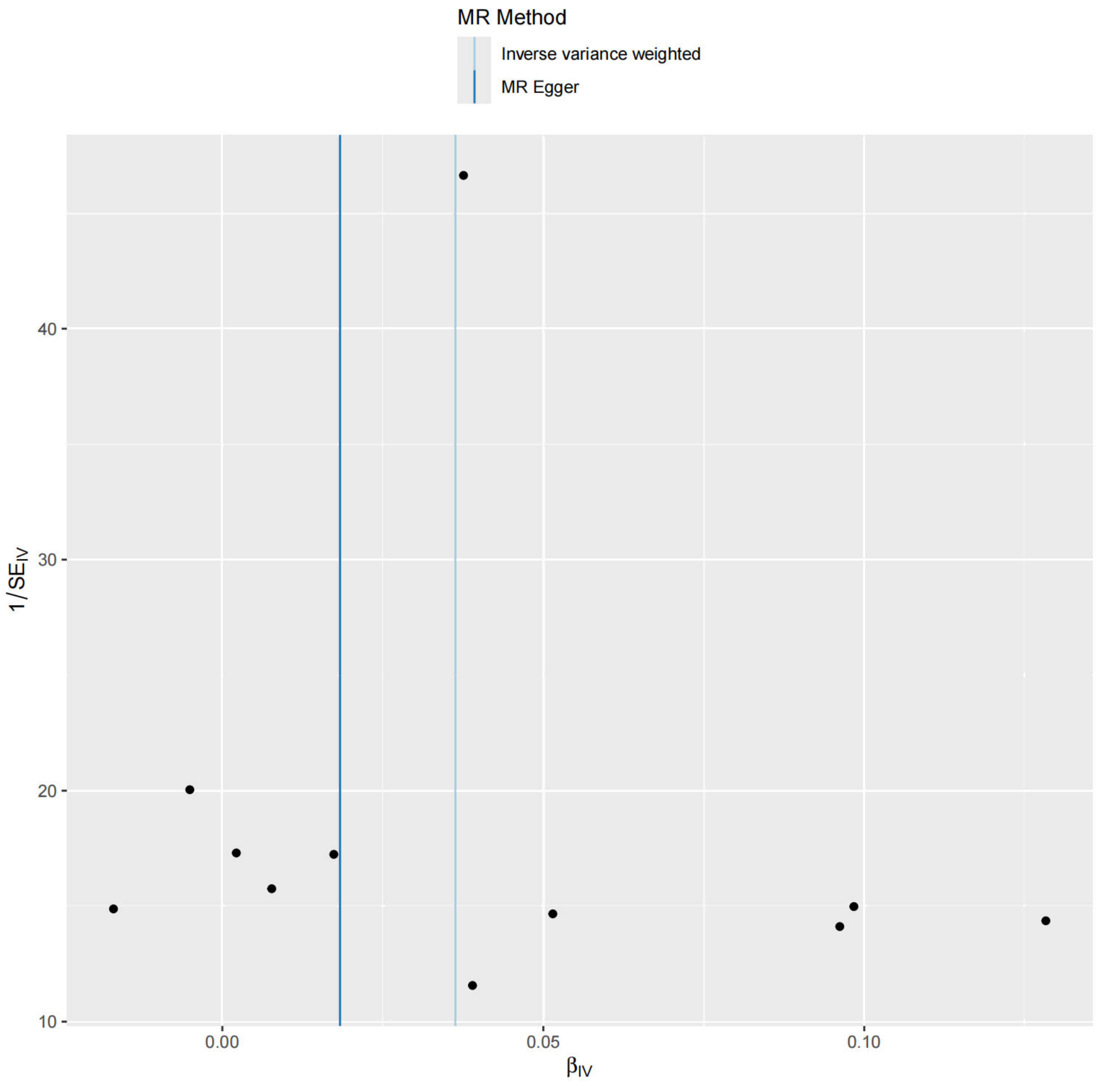
MR Method
Inverse variance weighted
MR Egger
40
30
20
10
$1/SE_{IV}$
0.00
0.05
0.10
β_{IV}

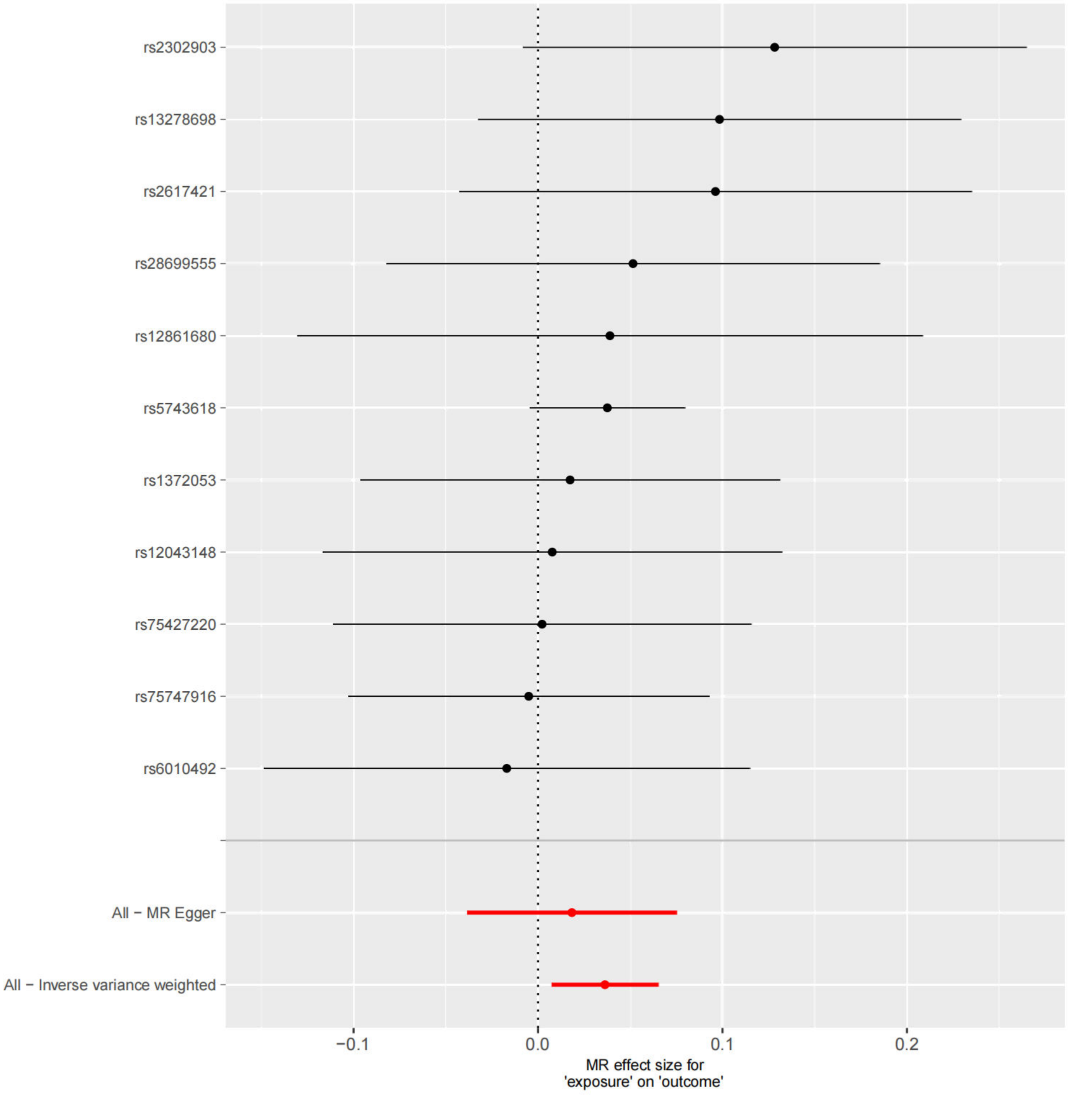
rs2302903
rs13278698
rs2617421
rs28699555
rs12861680
rs5743618
rs1372053
rs12043148
rs75427220
rs75747916
rs6010492
All − MR Egger
All − Inverse variance weighted
−0.1
0.0
0.1
0.2
MR effect size for
'exposure' on 'outcome'

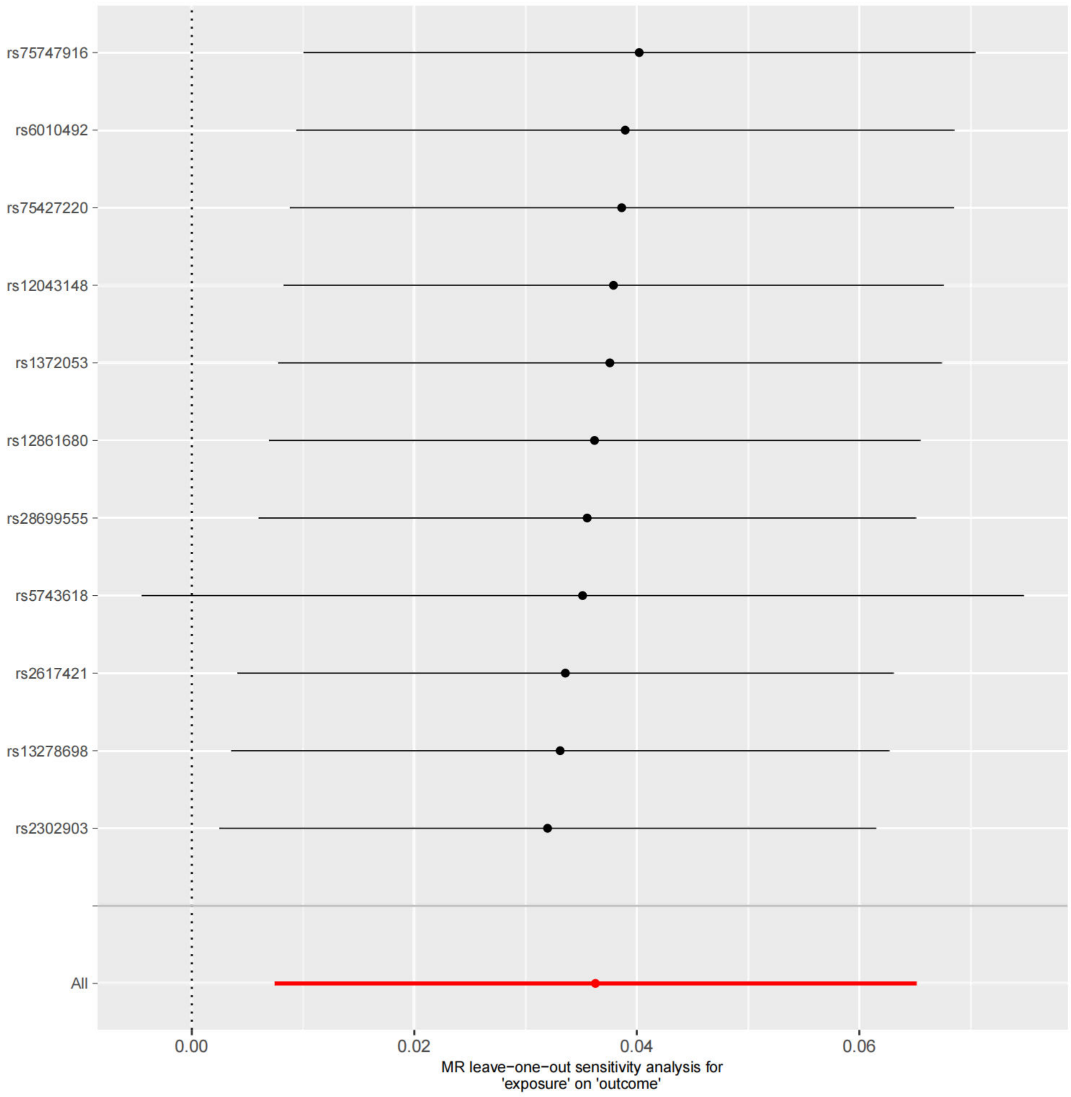
rs75747916
rs6010492
rs75427220
rs12043148
rs1372053
rs12861680
rs28699555
rs5743618
rs2617421
rs13278698
rs2302903
All
0.00
0.02
0.04
0.06
MR leave−one−out sensitivity analysis for
'exposure' on 'outcome'

GCST90257082

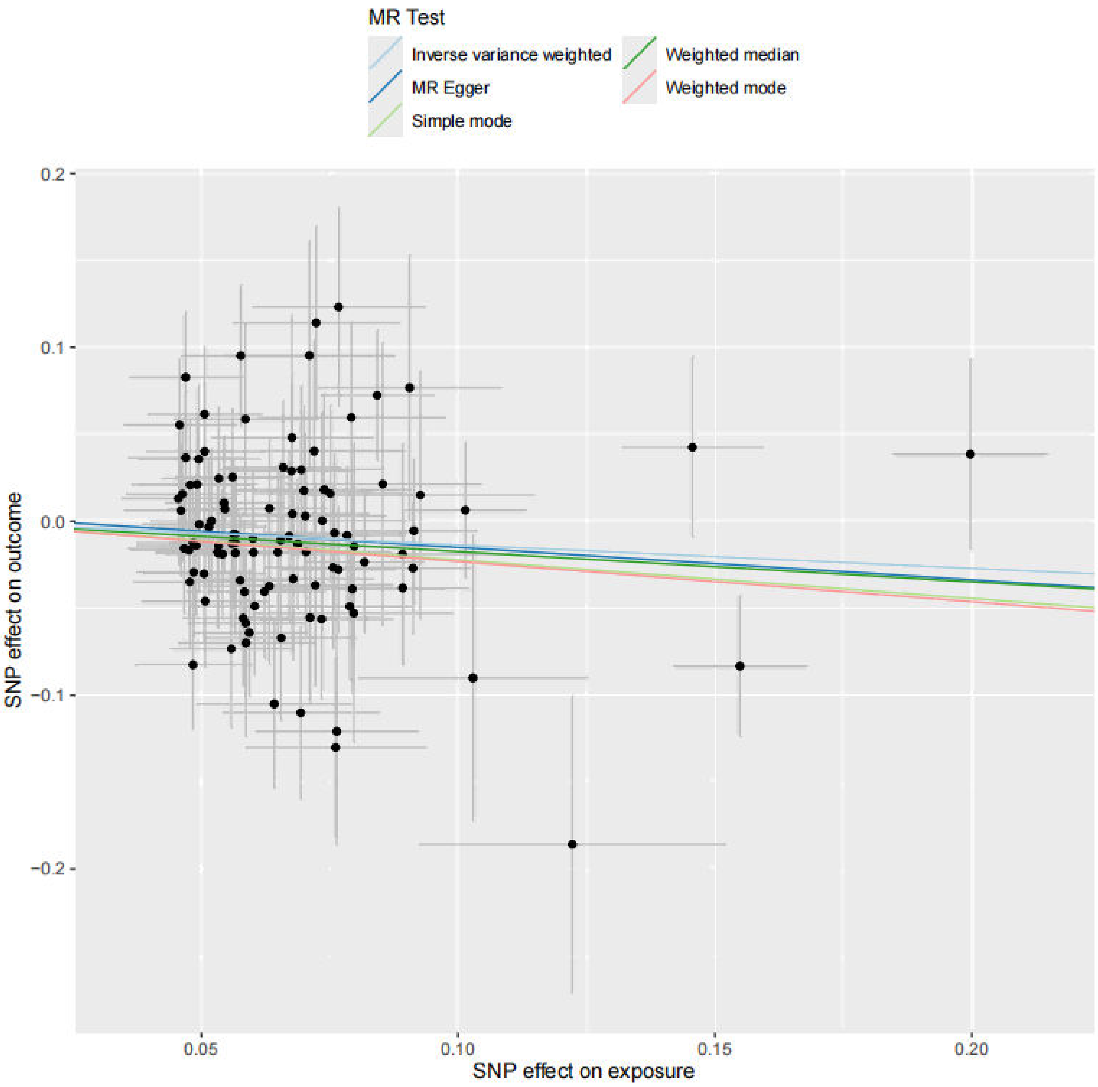

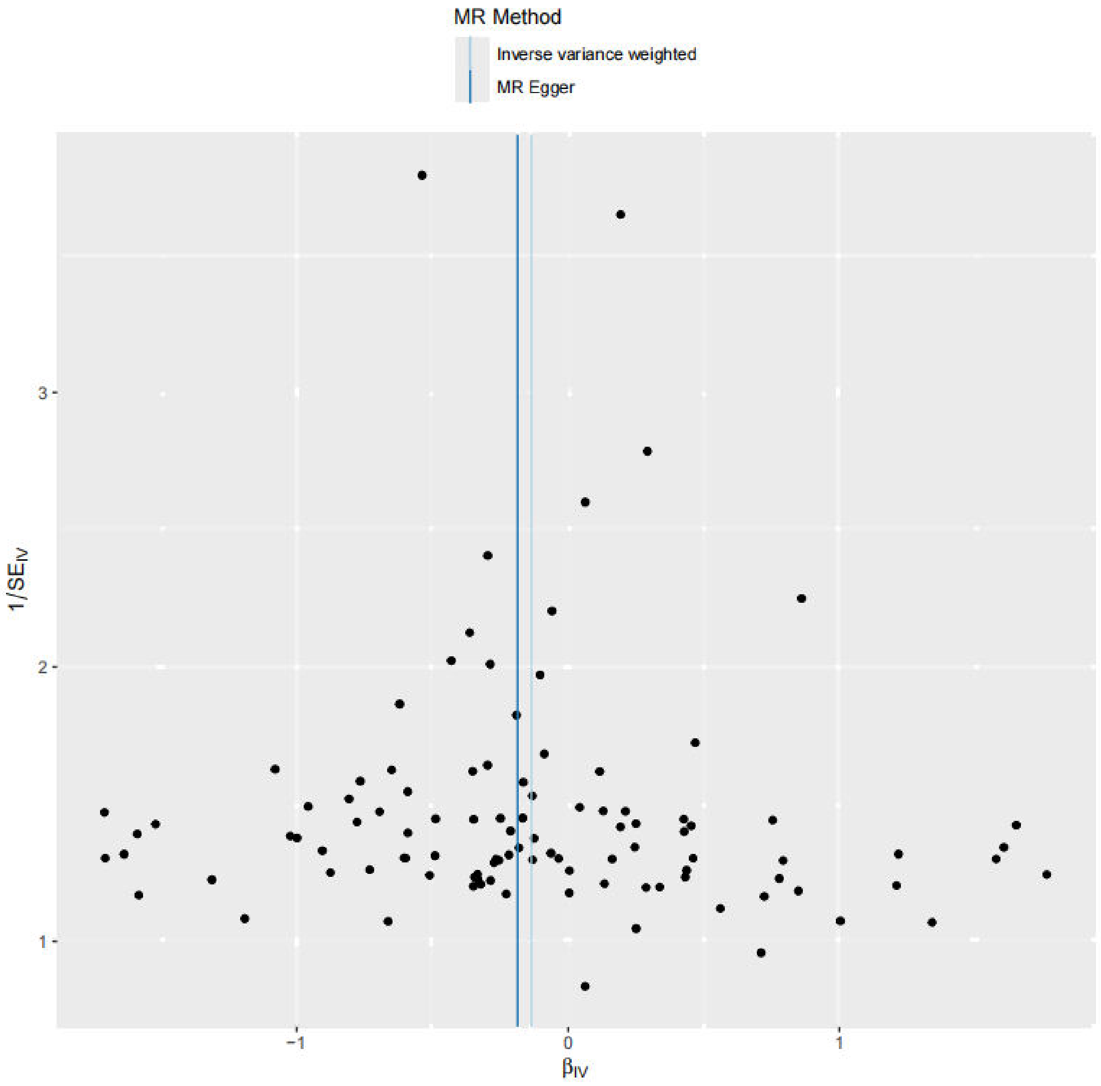
MR Method
Inverse variance weighted
MR Egger
1/SE$_{IV}$
3
2
1
−1
0
1
β$_{IV}$

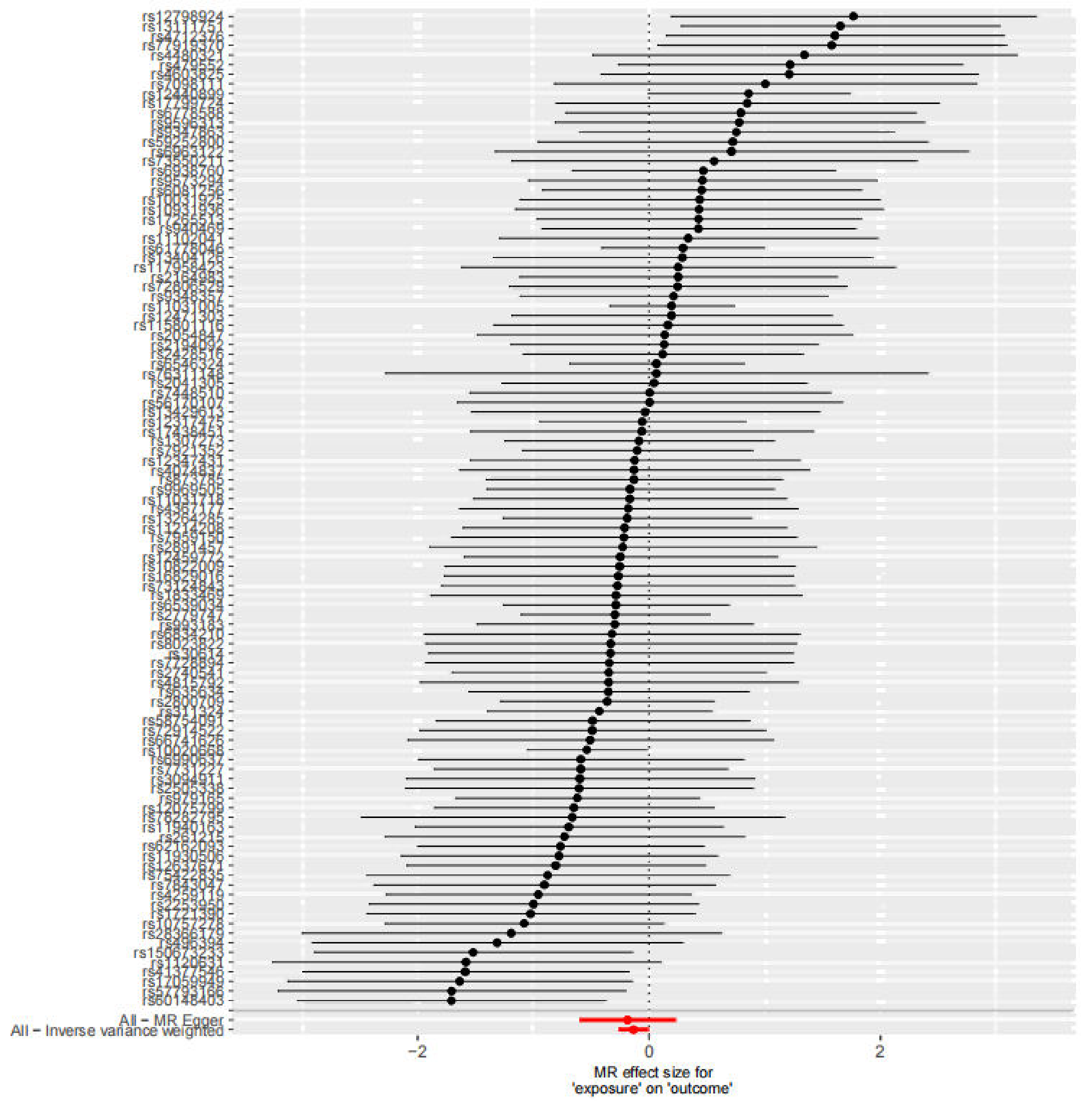

rs12798924
All – MR Egger
All – Inverse variance weighted
−2
0
2
MR effect size for
'exposure' on 'outcome'

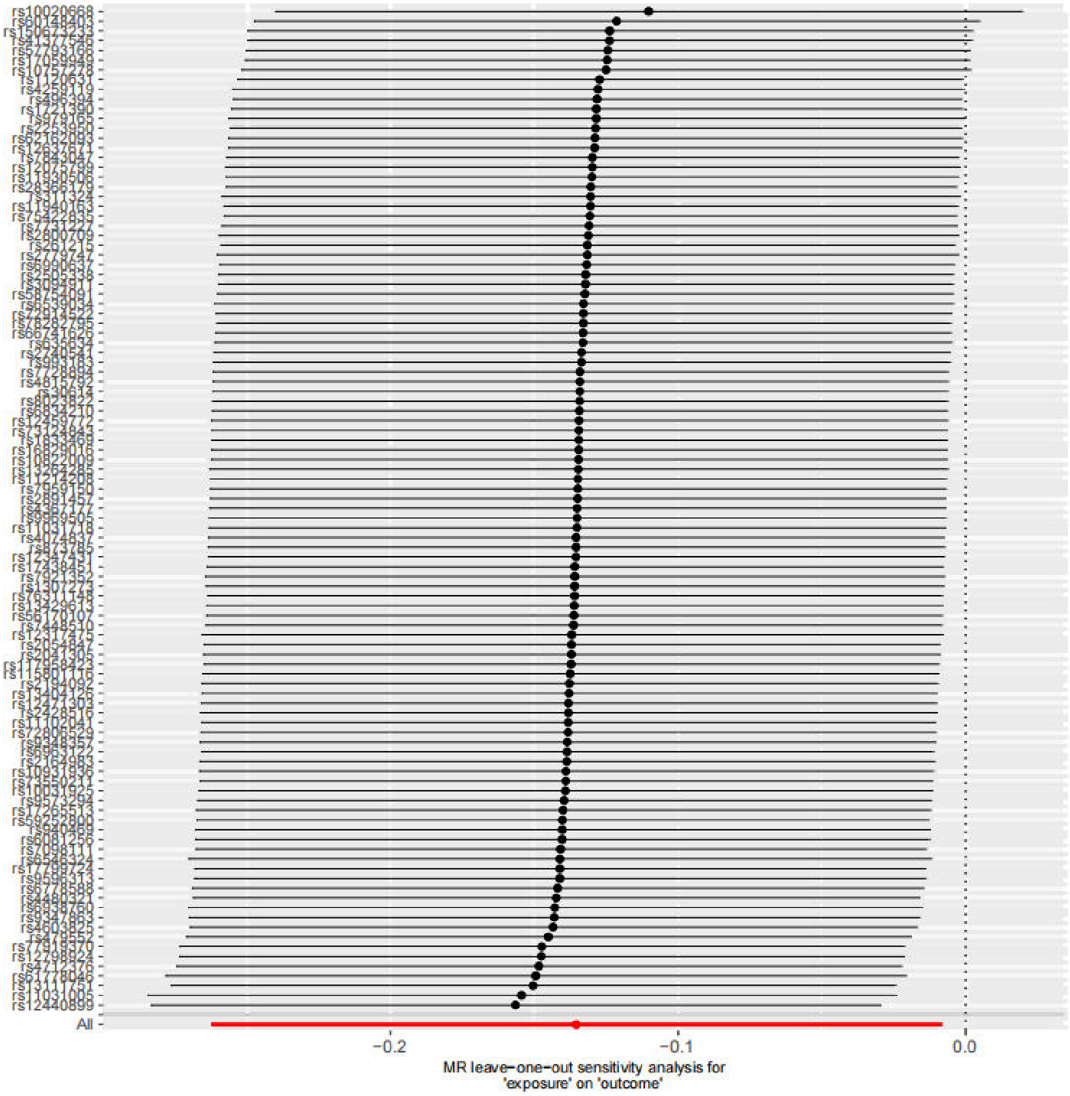
rs10020668
rs12440899
All
−0.2
−0.1
0.0
MR leave−one−out sensitivity analysis for
'exposure' on 'outcome'

GCST90257088

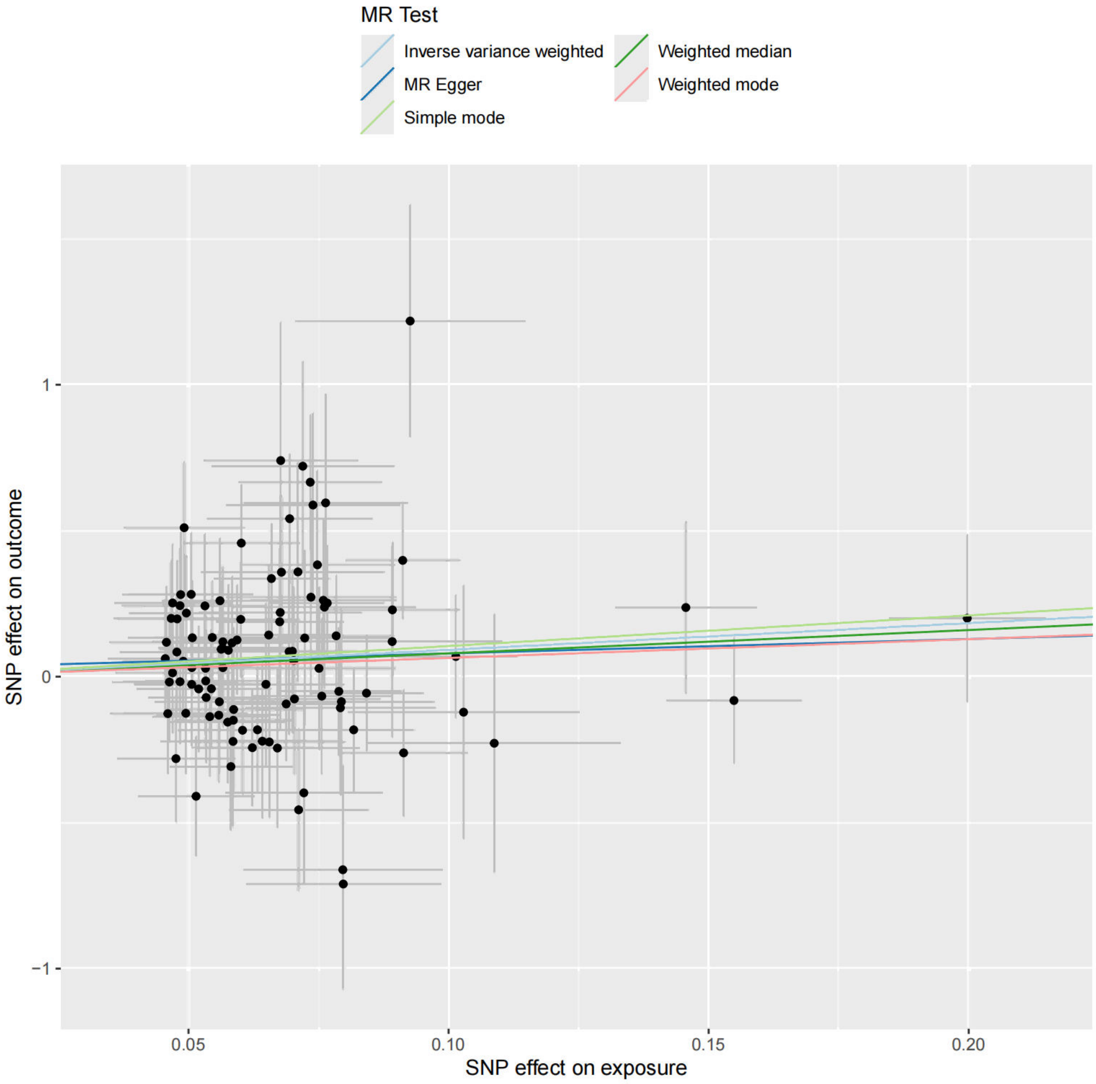

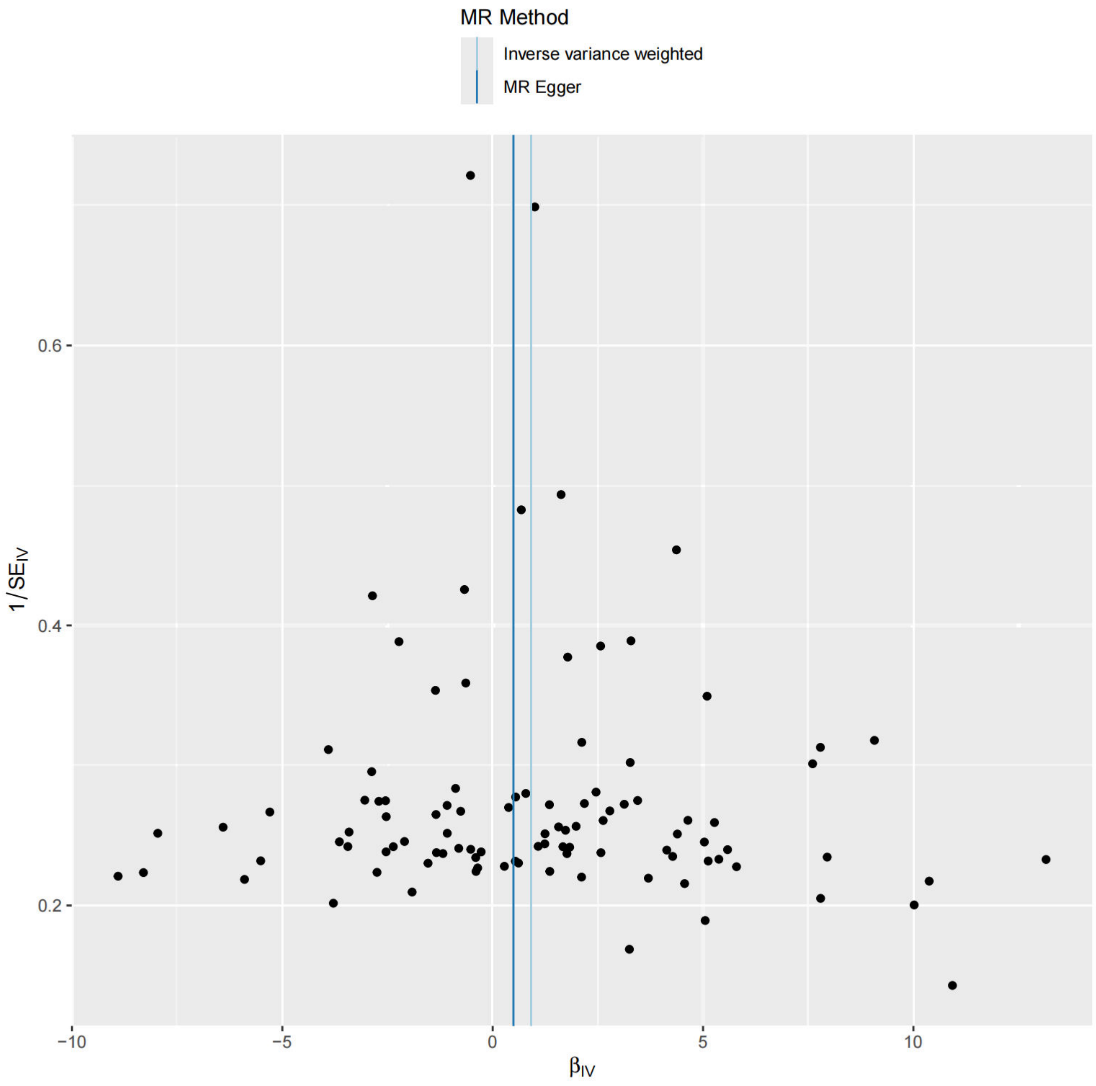

MR Method
Inverse variance weighted
MR Egger
1/SE$_{IV}$
0.6
0.4
0.2
−10
−5
0
5
10
β$_{IV}$

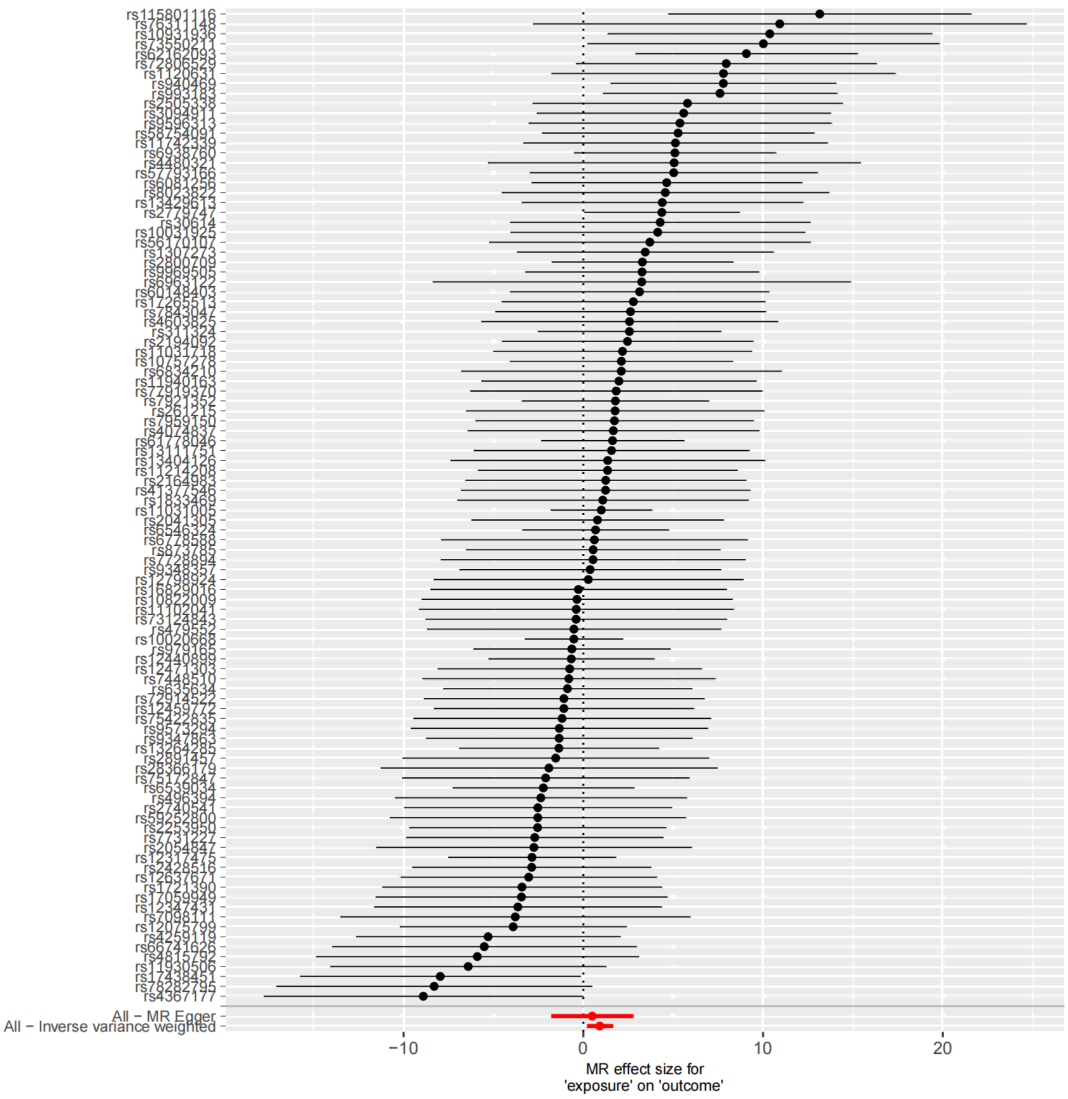
All − MR Egger
All − Inverse variance weighted
−10
0
10
20
MR effect size for
'exposure' on 'outcome'

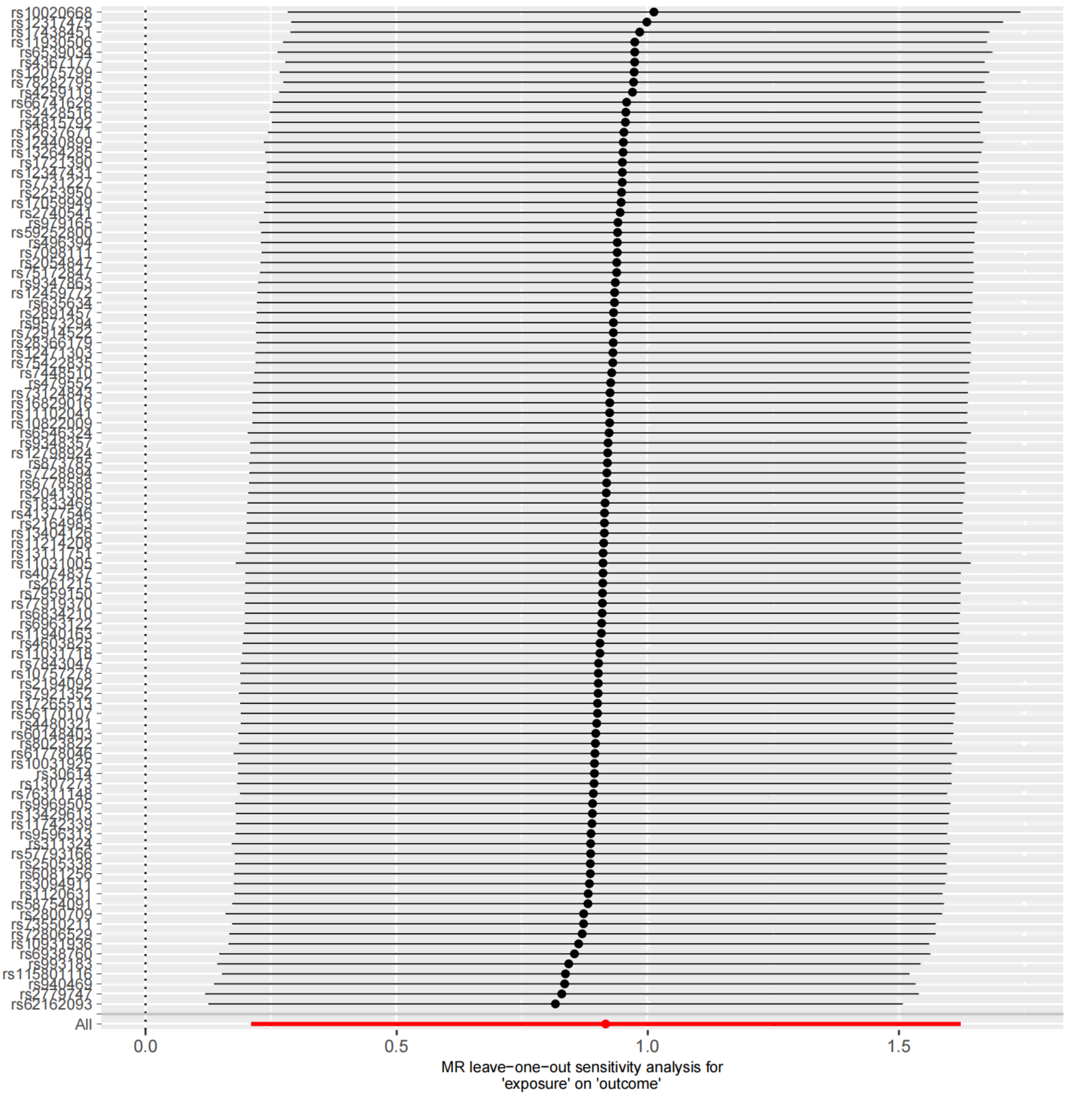
All
0.0
0.5
1.0
1.5
MR leave−one−out sensitivity analysis for
'exposure' on 'outcome'

GCST90257089

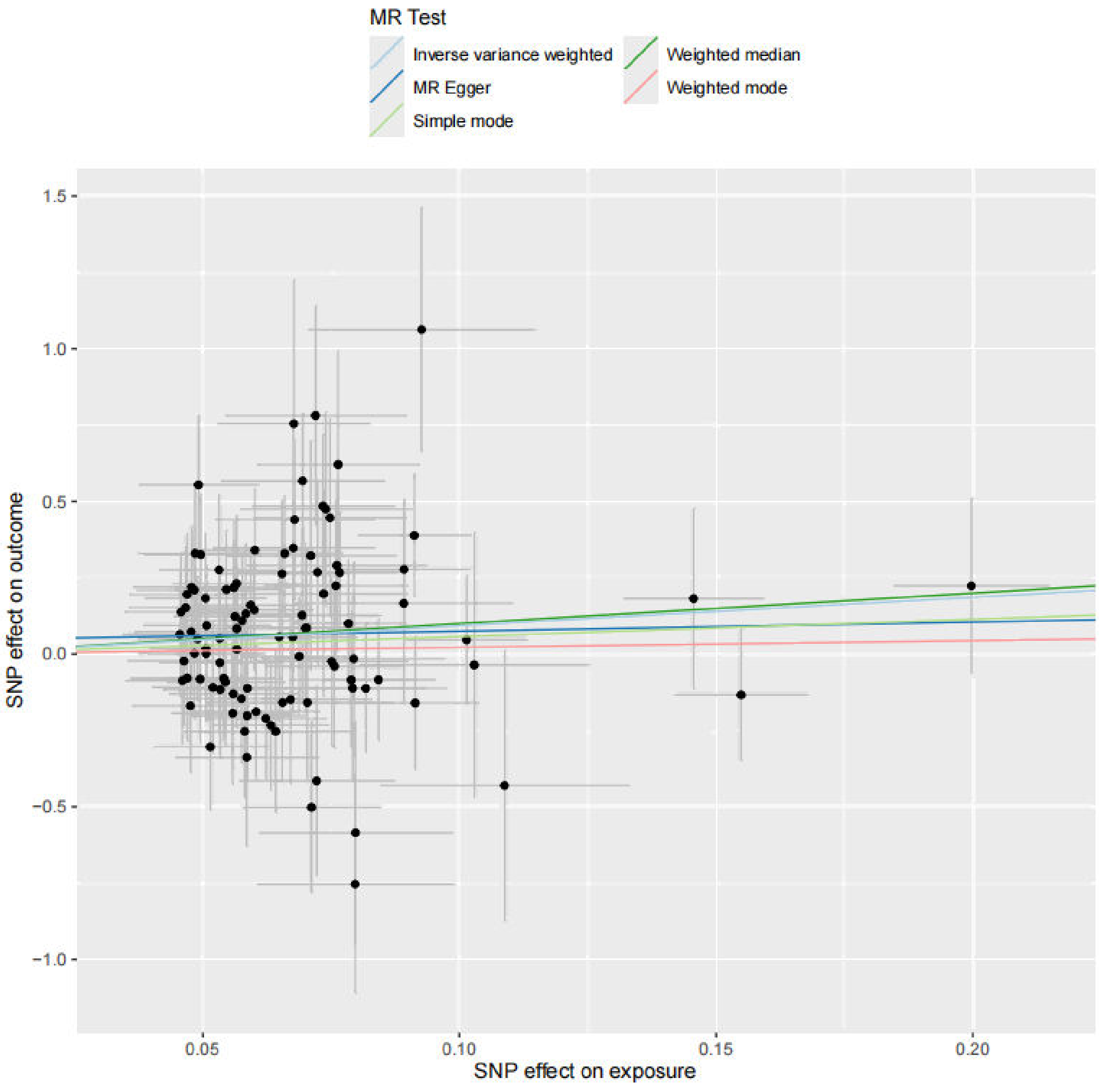

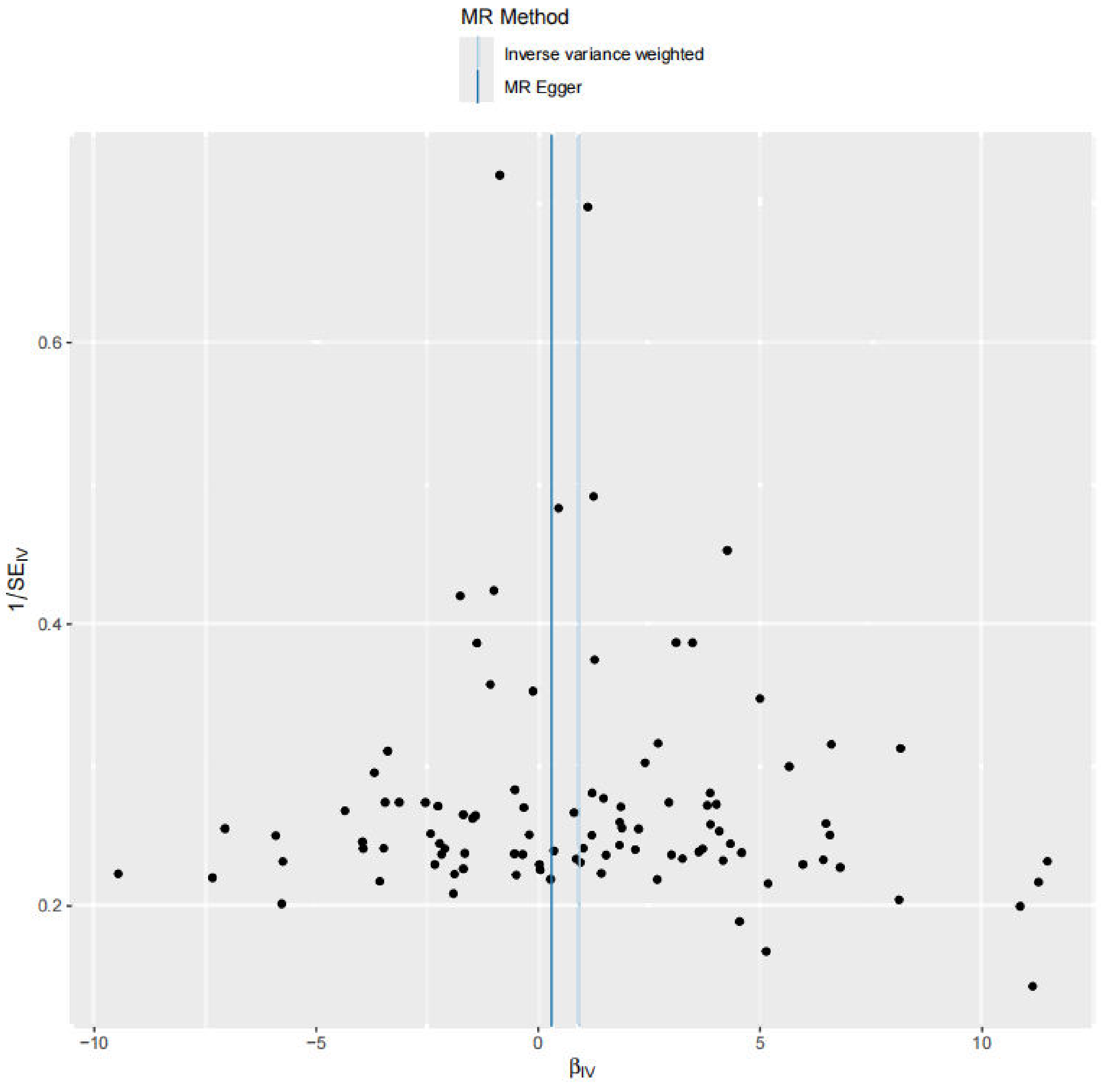
MR Method
Inverse variance weighted
MR Egger
1/SE_IV
0.6
0.4
0.2
−10
−5
0
5
10
β_IV

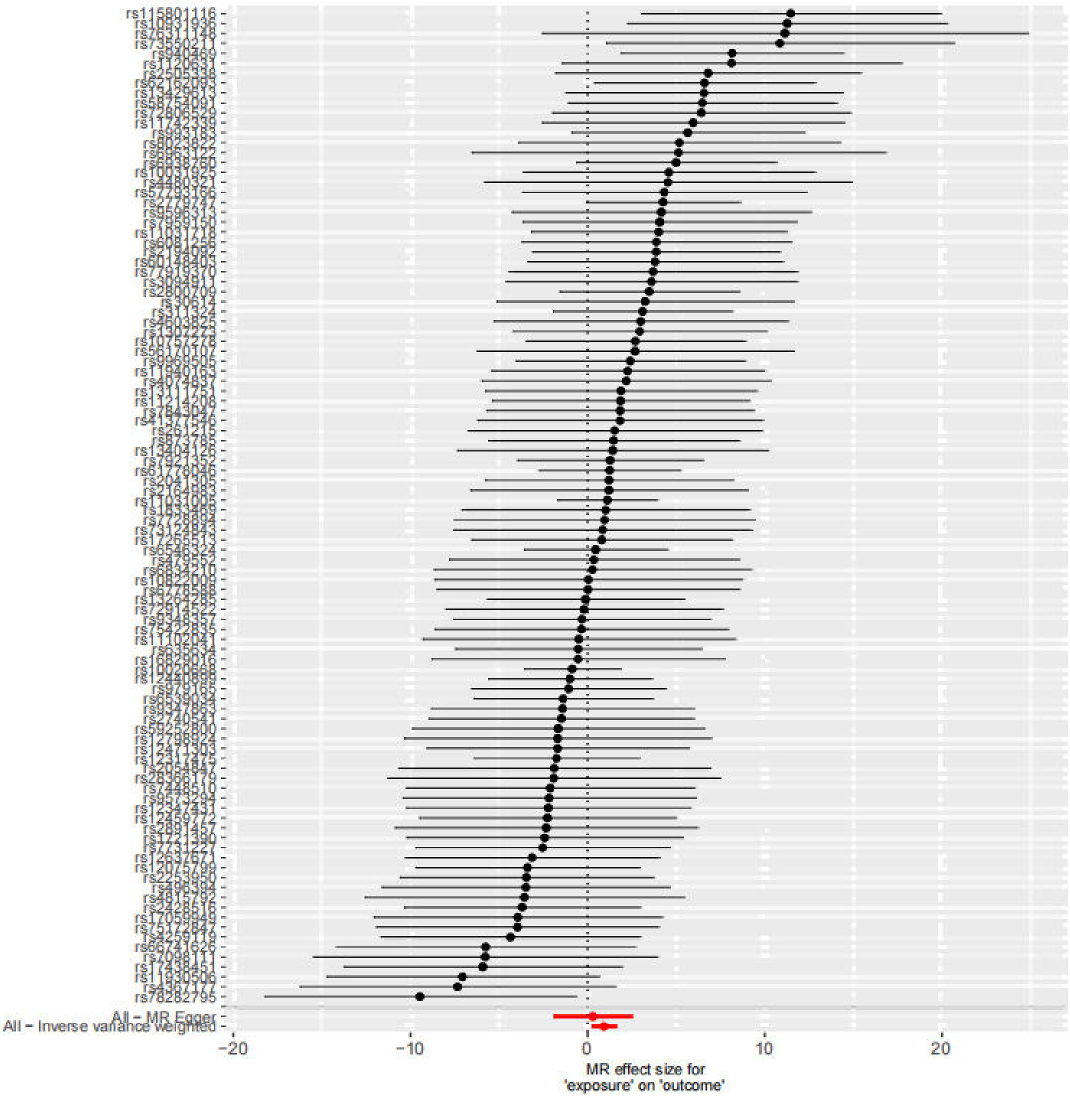
rs115801116
rs78282795
All – MR Egger
All – Inverse variance weighted
−20
−10
0
10
20
MR effect size for
'exposure' on 'outcome'

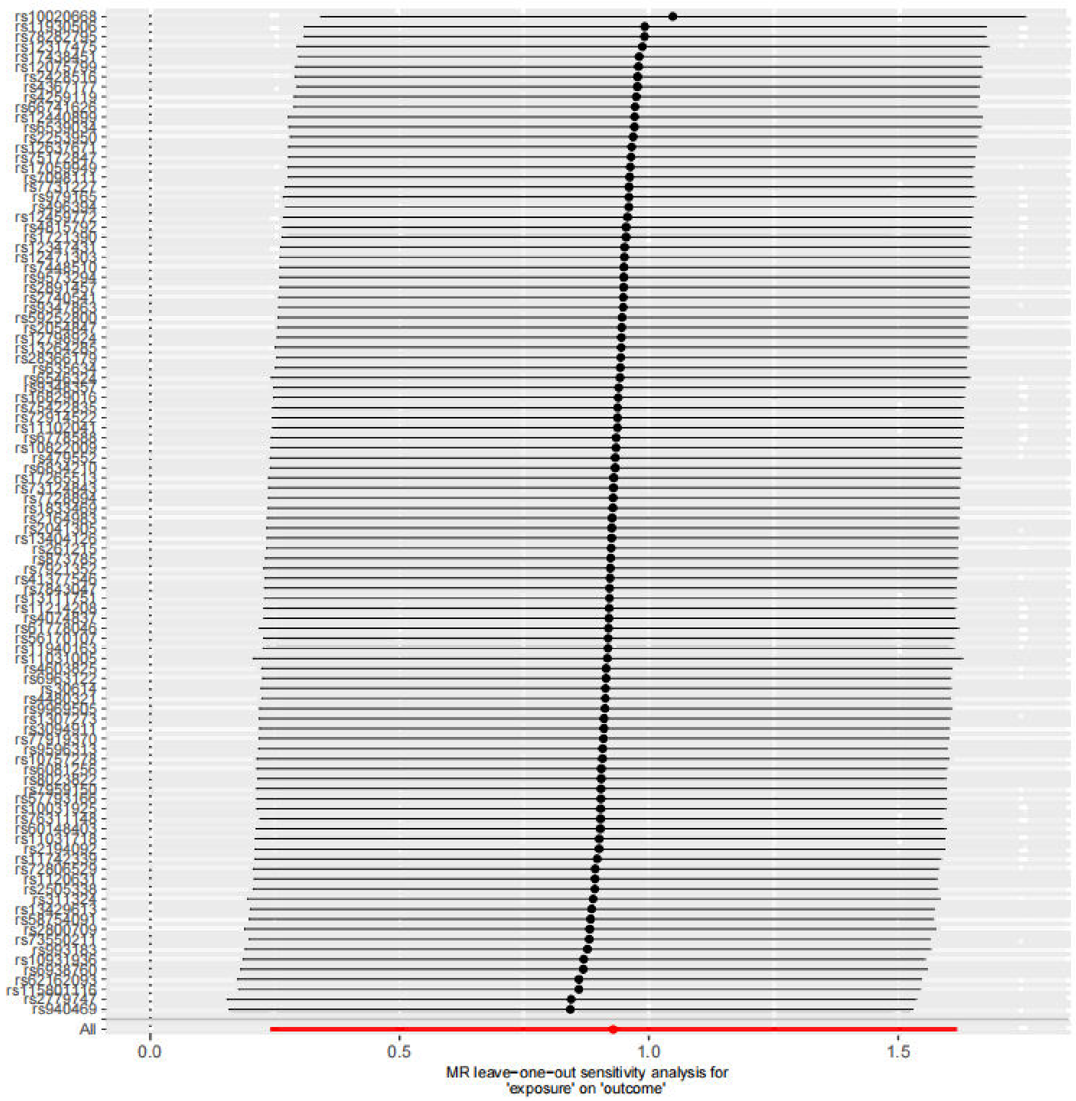
rs10020668
rs940469
All
0.0
0.5
1.0
1.5
MR leave−one−out sensitivity analysis for
'exposure' on 'outcome'

GCST90257039

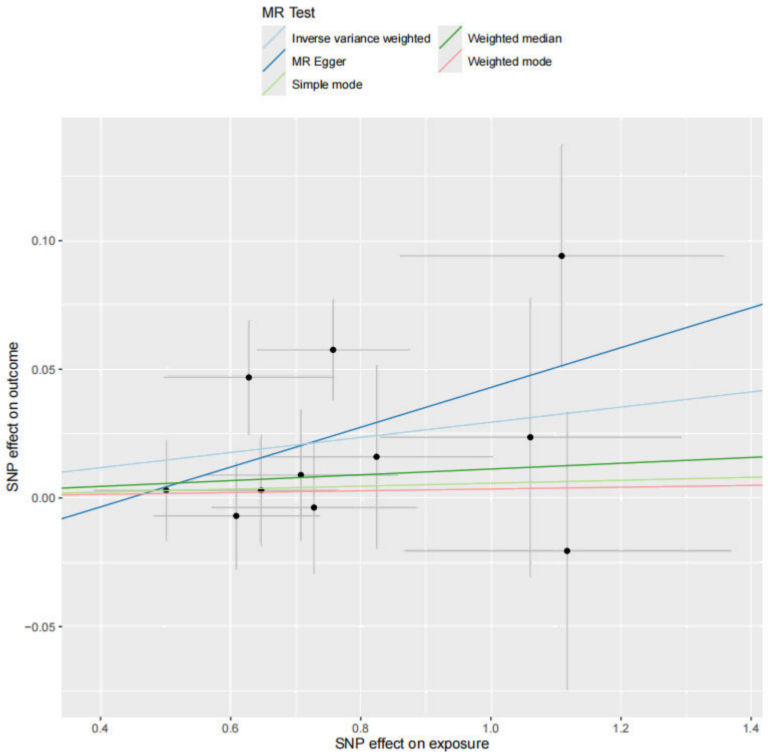

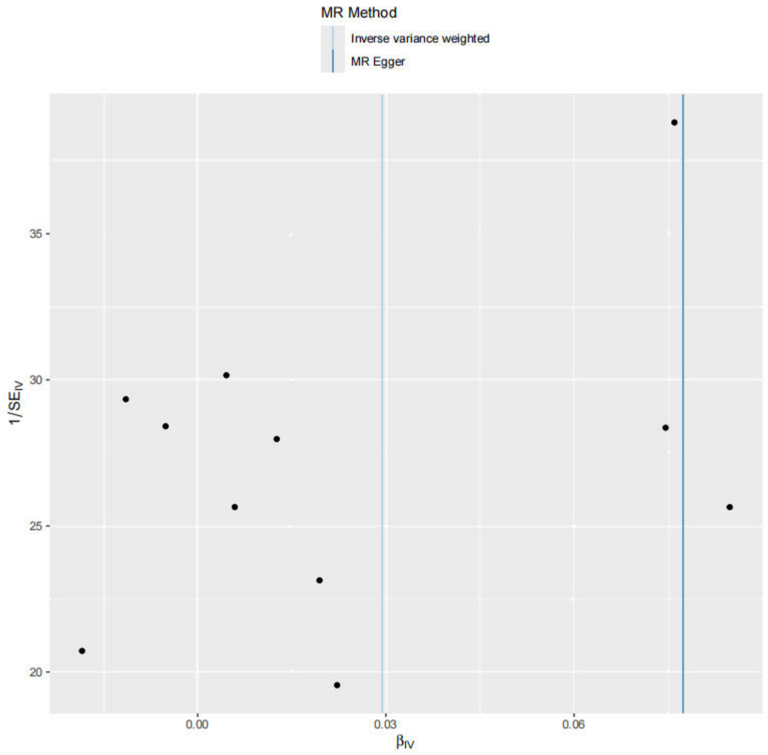

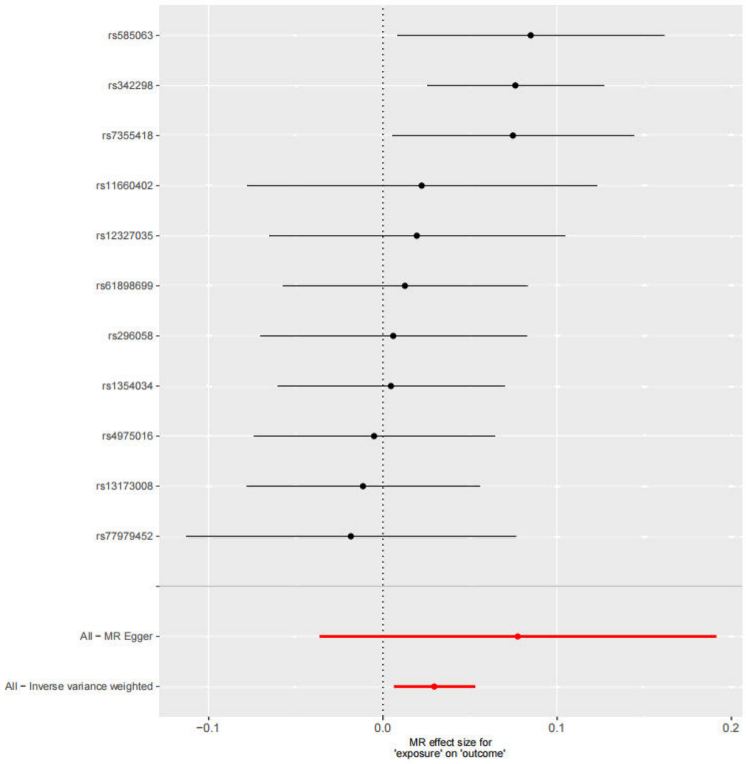

rs585063
rs342298
rs7355418
rs11660402
rs12327035
rs61898699
rs296058
rs1354034
rs4975016
rs13173008
rs77979452
All − MR Egger
All − Inverse variance weighted
−0.1
0.0
0.1
0.2
MR effect size for
'exposure' on 'outcome'

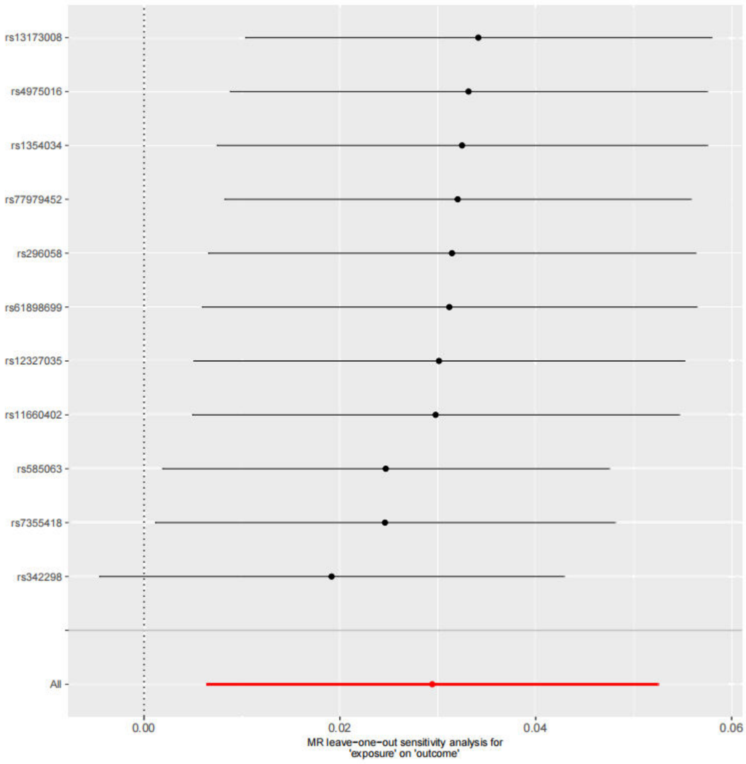

rs13173008
rs4975016
rs1354034
rs77979452
rs296058
rs61898699
rs12327035
rs11660402
rs585063
rs7355418
rs342298
All
0.00
0.02
0.04
0.06
MR leave−one−out sensitivity analysis for
'exposure' on 'outcome'

GCST90257053

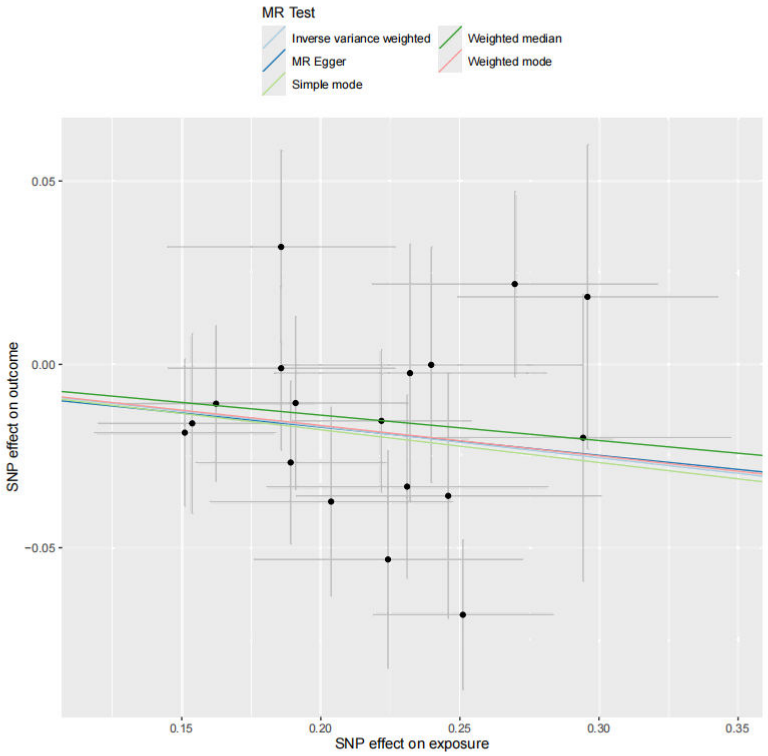

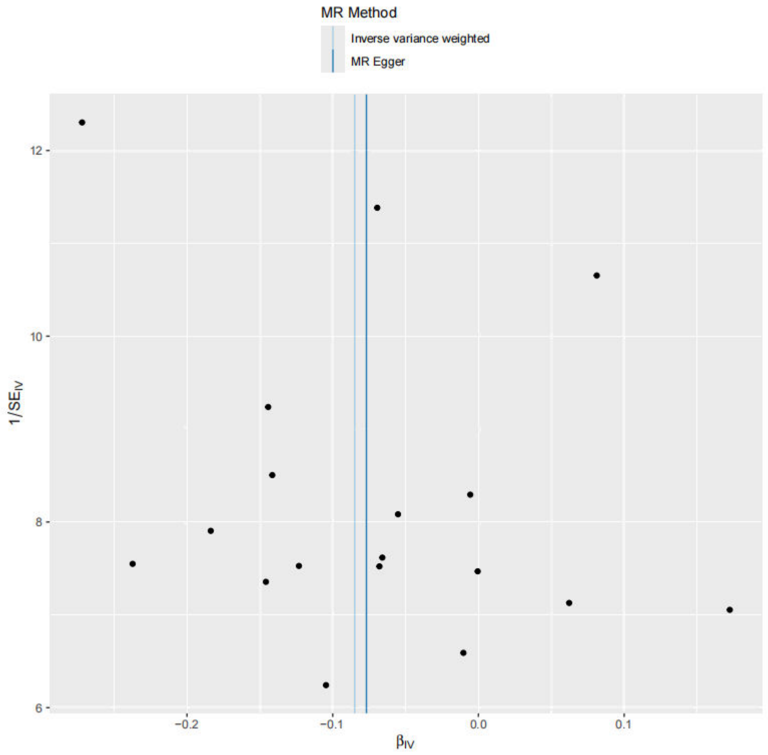

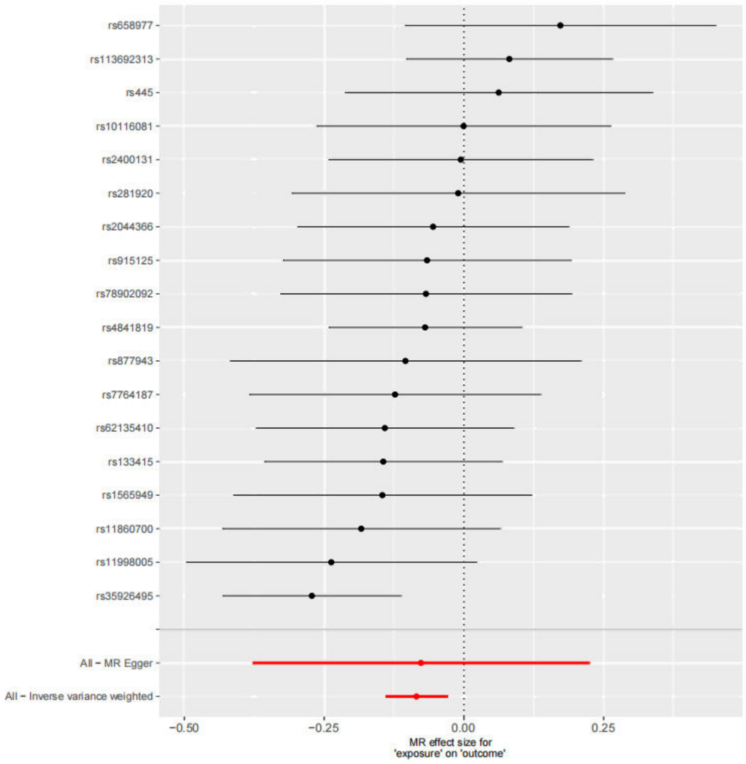
rs658977
rs113692313
rs445
rs10116081
rs2400131
rs281920
rs2044366
rs915125
rs78902092
rs4841819
rs877943
rs7764187
rs62135410
rs133415
rs1565949
rs11860700
rs11998005
rs35926495
All − MR Egger
All − Inverse variance weighted
−0.50
−0.25
0.00
0.25
MR effect size for
'exposure' on 'outcome'

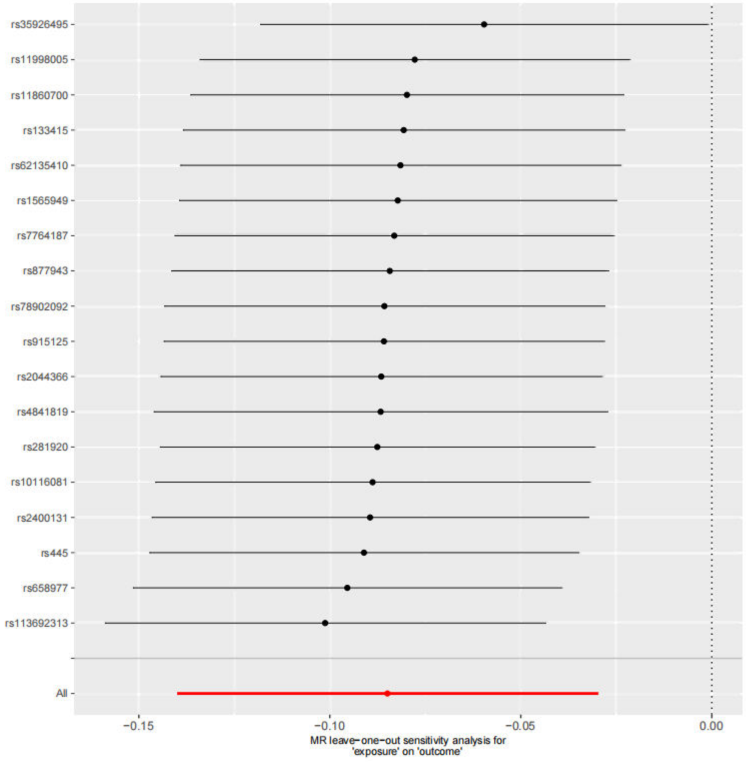
rs35926495
rs11998005
rs11860700
rs133415
rs62135410
rs1565949
rs7764187
rs877943
rs78902092
rs915125
rs2044366
rs4841819
rs281920
rs10116081
rs2400131
rs445
rs658977
rs113692313
All
−0.15
−0.10
−0.05
0.00
MR leave−one−out sensitivity analysis for
'exposure' on 'outcome'

GCST90257054

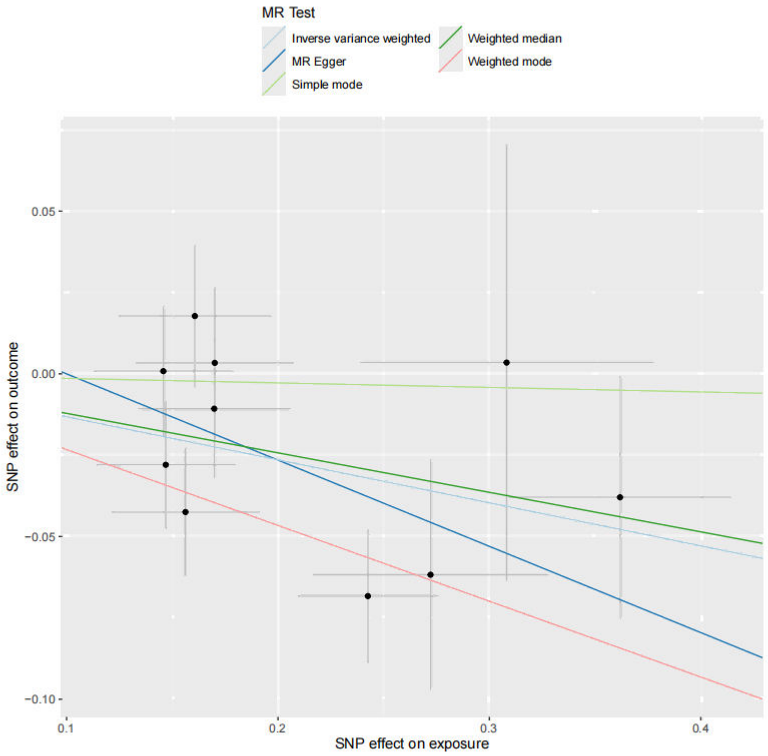

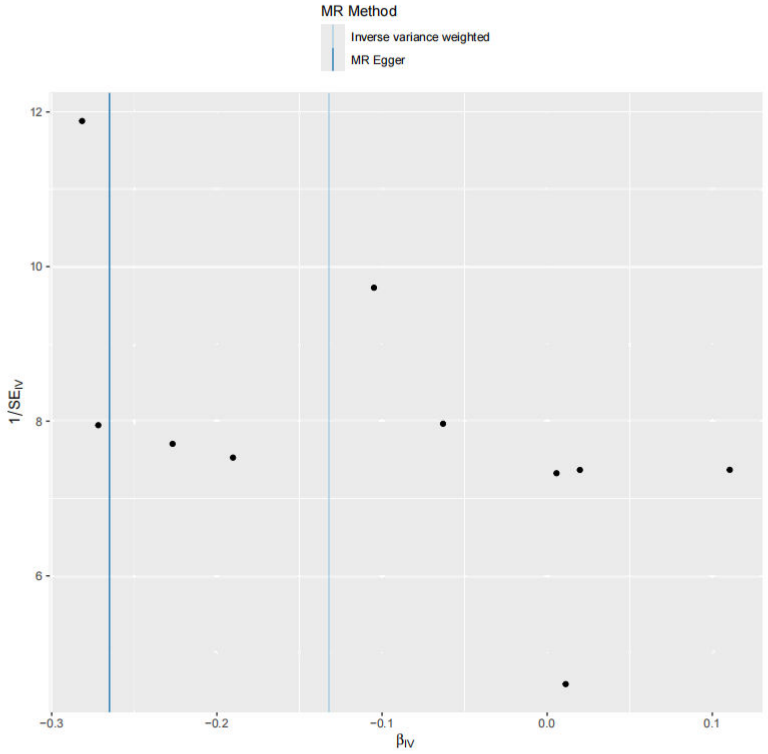

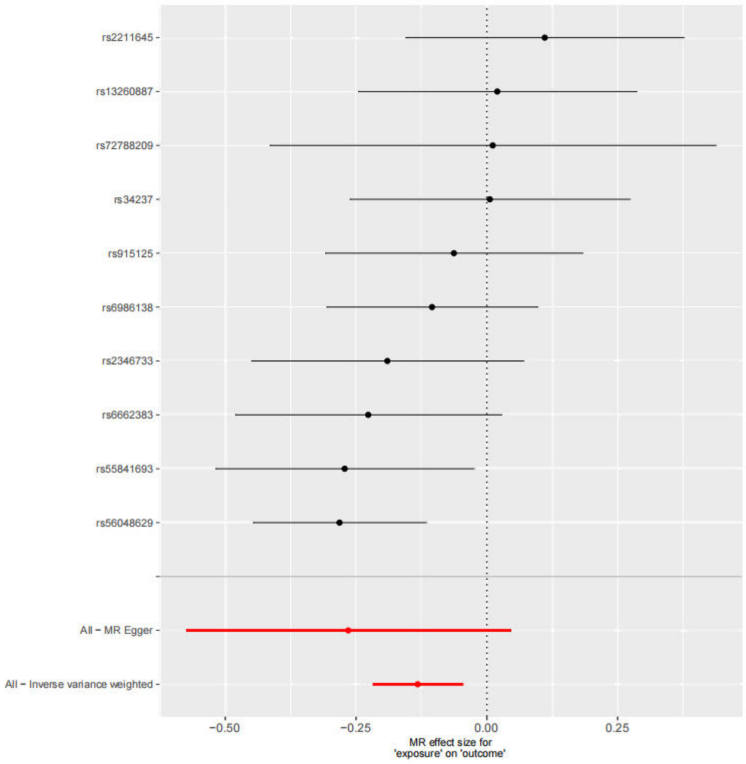

rs2211645
rs13260887
rs72788209
rs34237
rs915125
rs6986138
rs2346733
rs6662383
rs55841693
rs56048629
All − MR Egger
All − Inverse variance weighted
−0.50
−0.25
0.00
0.25
MR effect size for
'exposure' on 'outcome'

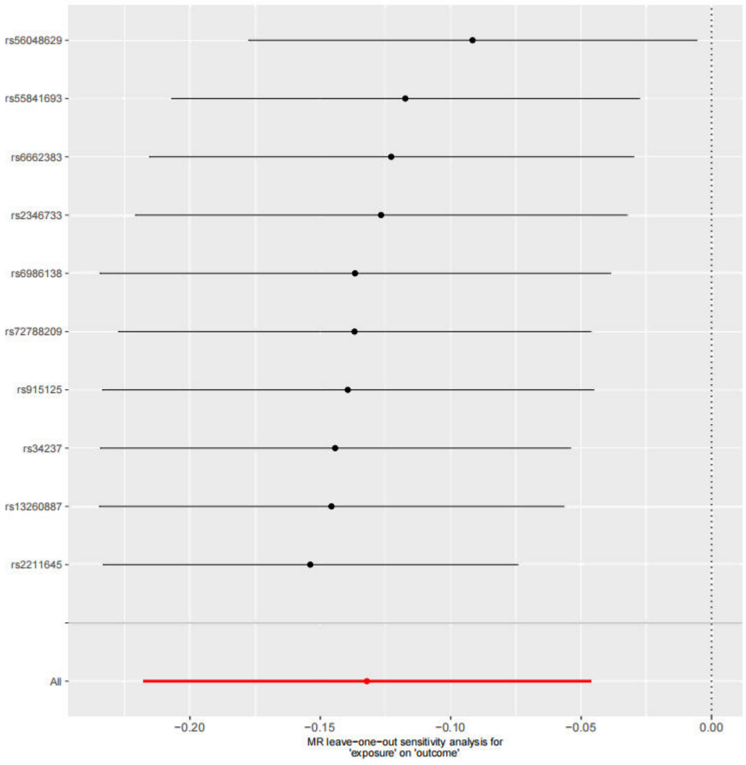

rs56048629
rs55841693
rs6662383
rs2346733
rs6986138
rs72788209
rs915125
rs34237
rs13260887
rs2211645
All
−0.20
−0.15
−0.10
−0.05
0.00
MR leave−one−out sensitivity analysis for
'exposure' on 'outcome'

GCST90257057

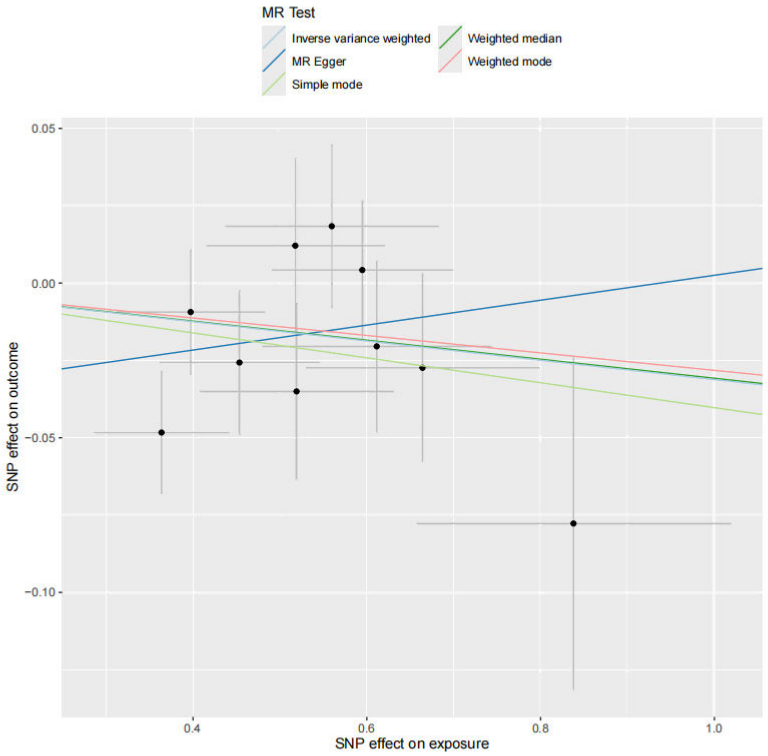

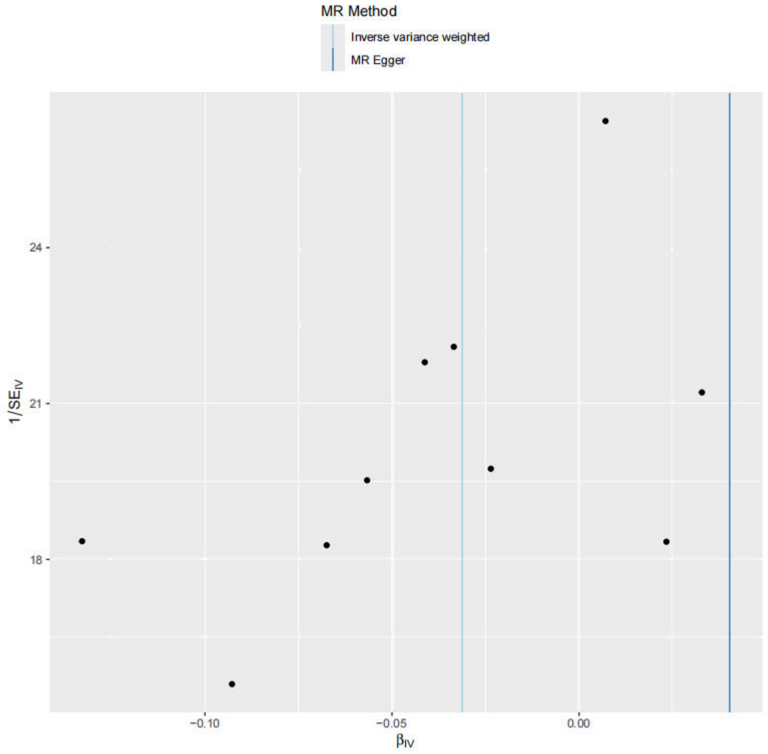

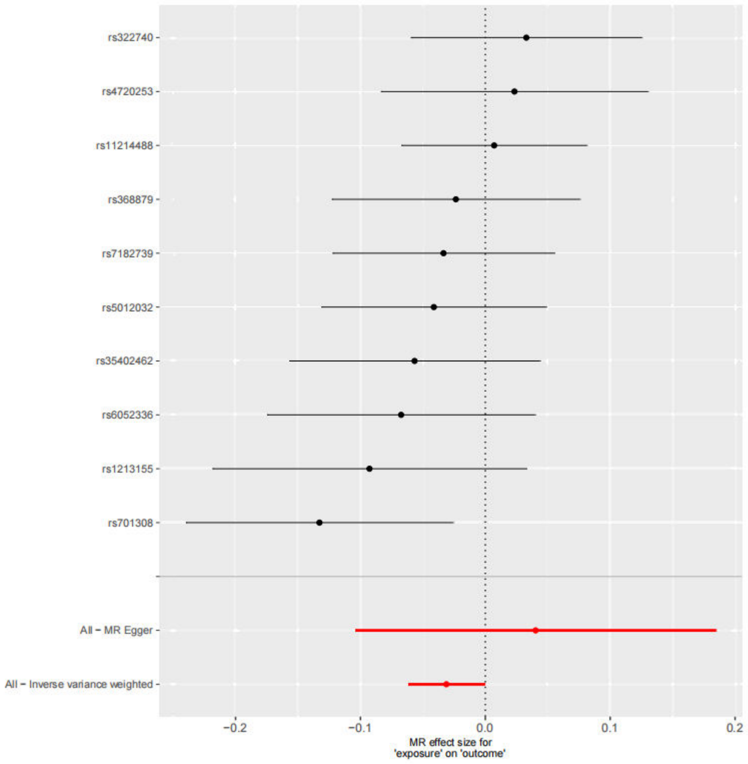

rs322740
rs4720253
rs11214488
rs368879
rs7182739
rs5012032
rs35402462
rs6052336
rs1213155
rs701308
All − MR Egger
All − Inverse variance weighted
−0.2
−0.1
0.0
0.1
0.2
MR effect size for
'exposure' on 'outcome'

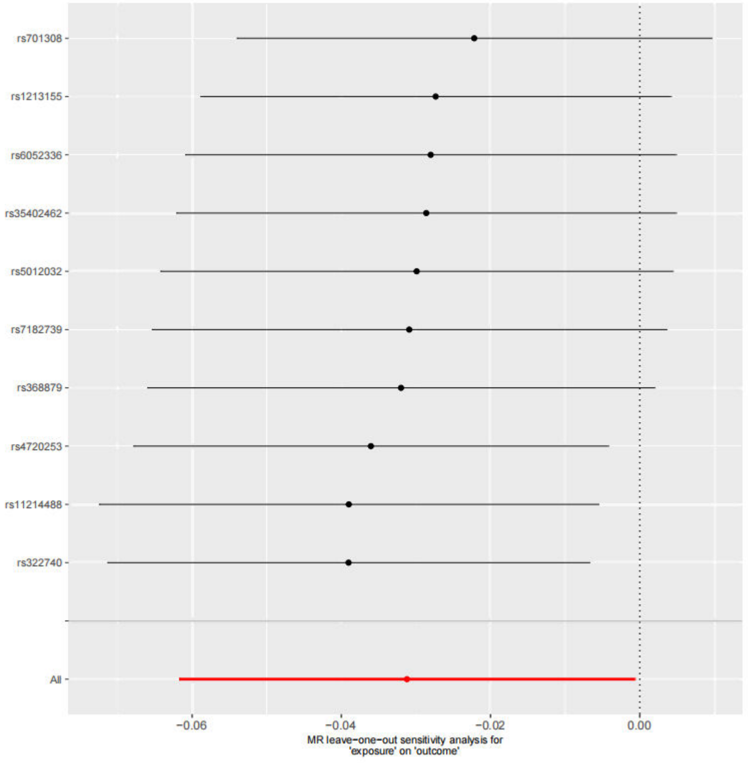

rs701308
rs1213155
rs6052336
rs35402462
rs5012032
rs7182739
rs368879
rs4720253
rs11214488
rs322740
All
−0.06
−0.04
−0.02
0.00
MR leave−one−out sensitivity analysis for
'exposure' on 'outcome'

GCST90257090

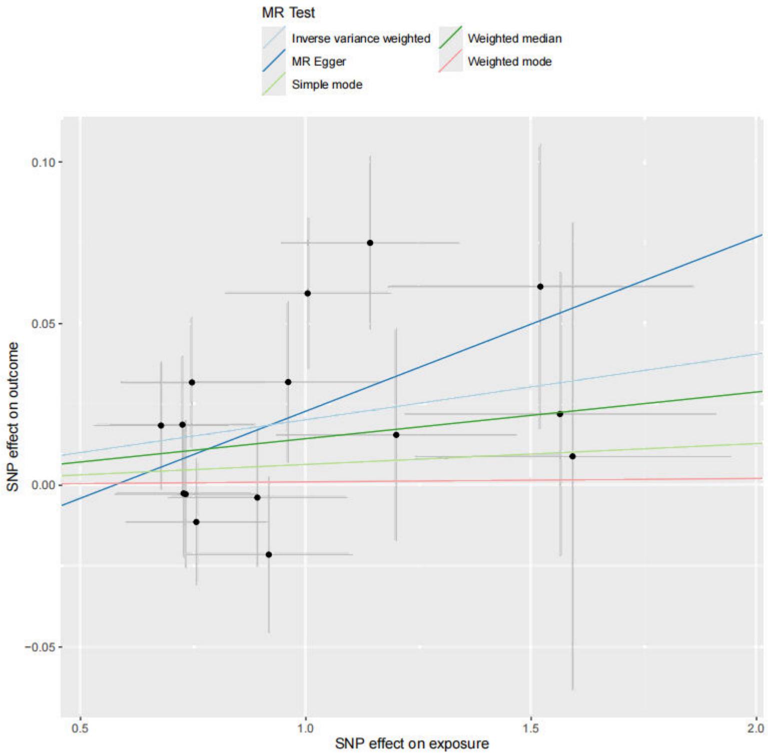

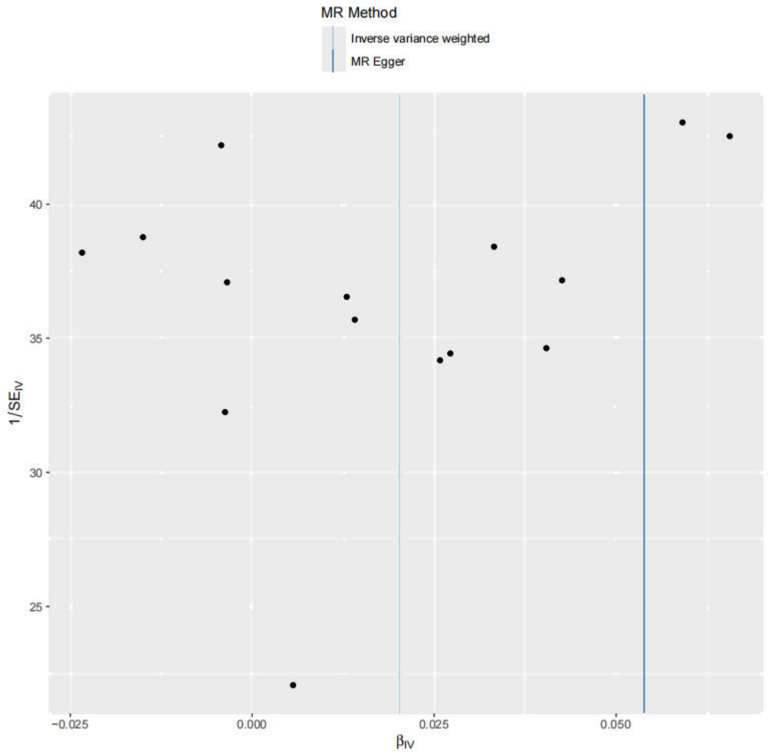

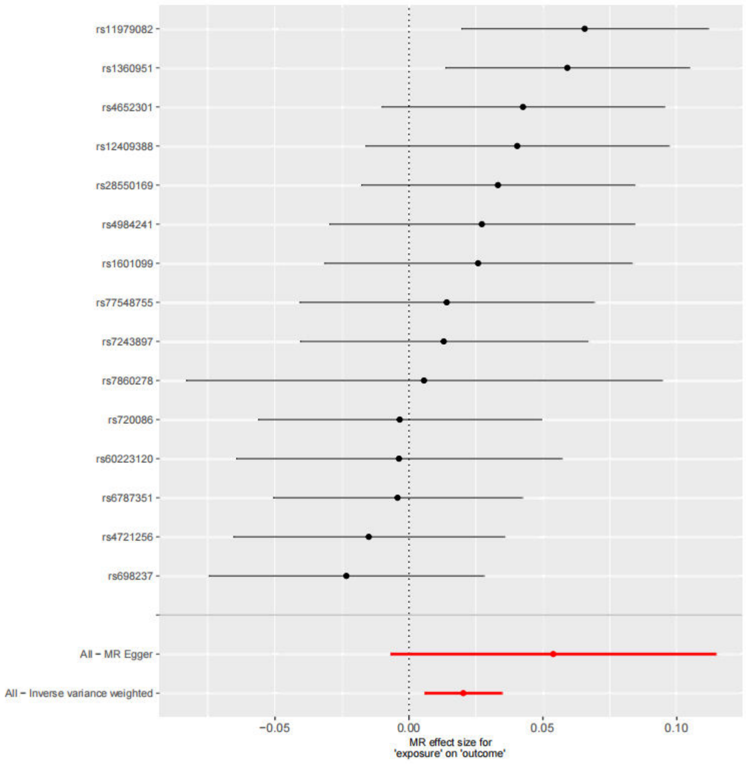
rs11979082
rs1360951
rs4652301
rs12409388
rs28550169
rs4984241
rs1601099
rs77548755
rs7243897
rs7860278
rs720086
rs60223120
rs6787351
rs4721256
rs698237
All − MR Egger
All − Inverse variance weighted
−0.05
0.00
0.05
0.10
MR effect size for
'exposure' on 'outcome'

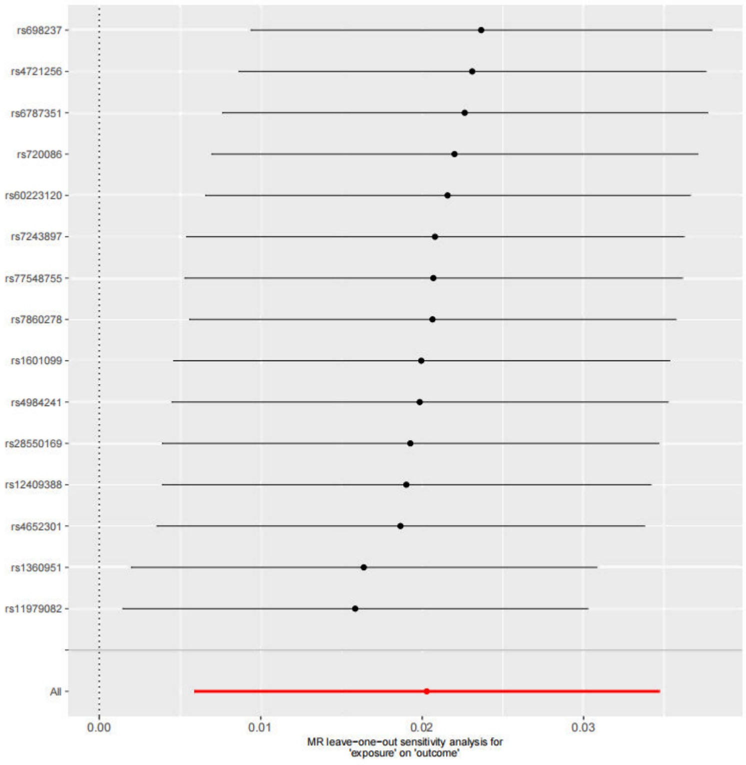
rs698237
rs4721256
rs6787351
rs720086
rs60223120
rs7243897
rs77548755
rs7860278
rs1601099
rs4984241
rs28550169
rs12409388
rs4652301
rs1360951
rs11979082
All
0.00
0.01
0.02
0.03
MR leave−one−out sensitivity analysis for
'exposure' on 'outcome'

GCST90257092

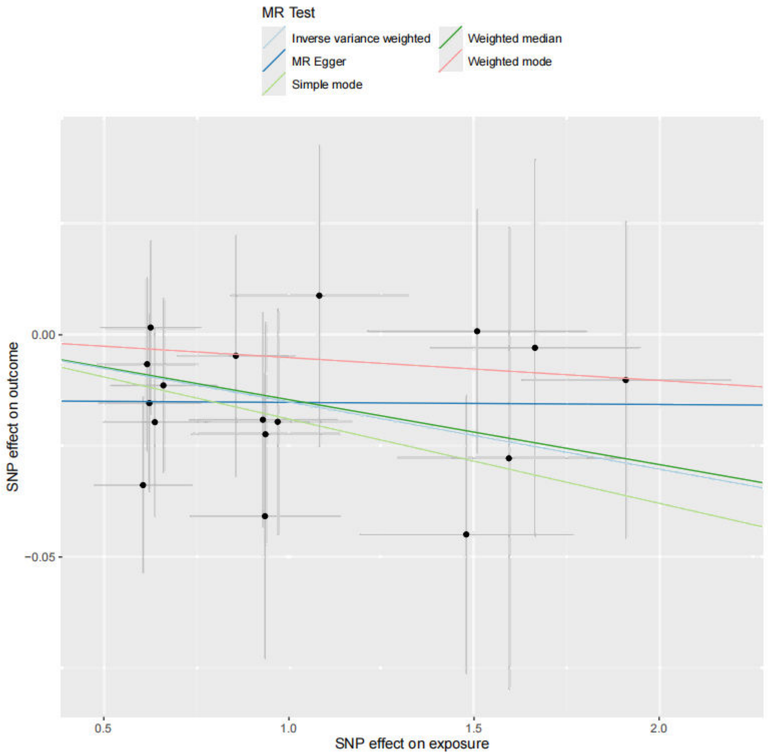

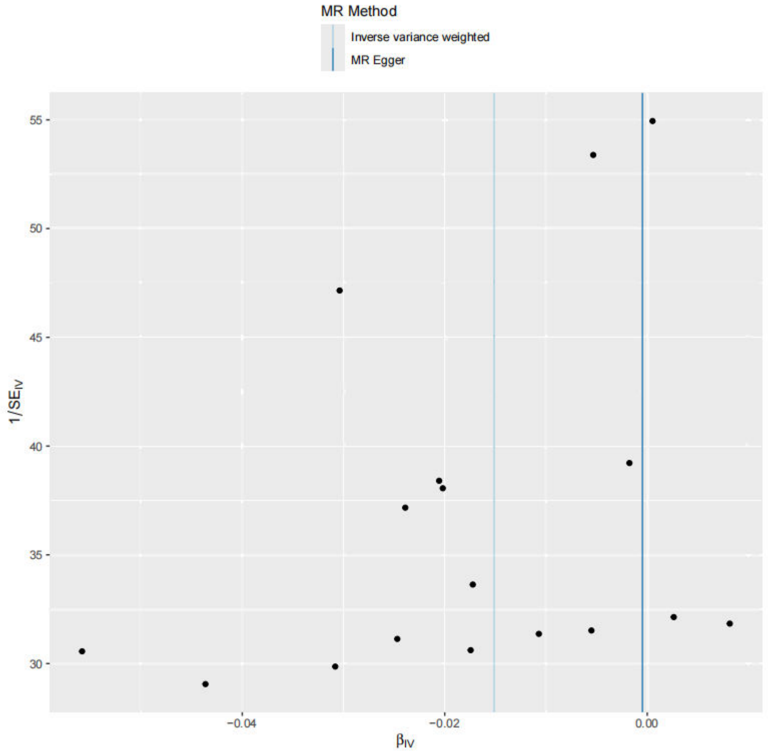

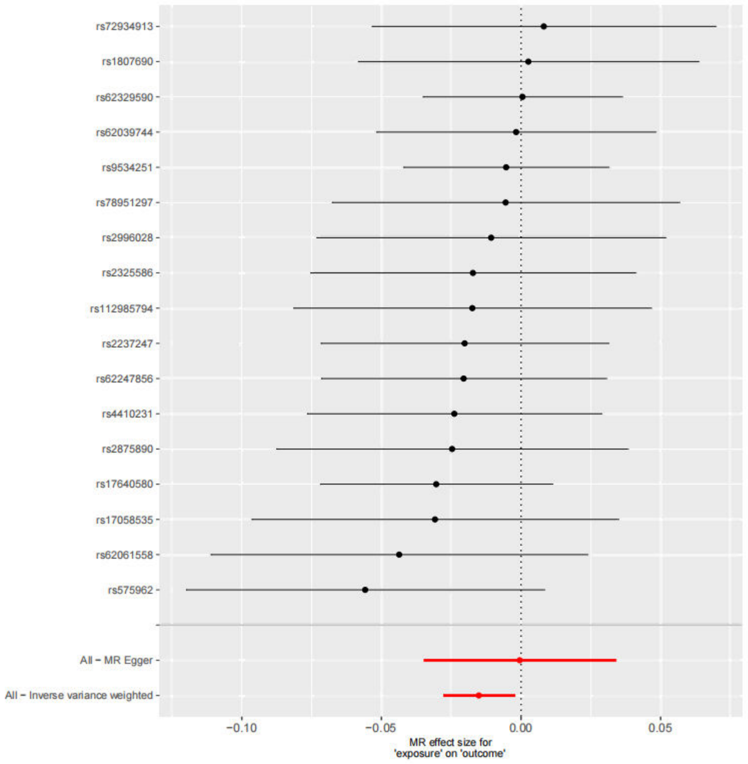
rs72934913
rs1807690
rs62329590
rs62039744
rs9534251
rs78951297
rs2996028
rs2325586
rs112985794
rs2237247
rs62247856
rs4410231
rs2875890
rs17640580
rs17058535
rs62061558
rs575962
All − MR Egger
All − Inverse variance weighted
−0.10
−0.05
0.00
0.05
MR effect size for
'exposure' on 'outcome'

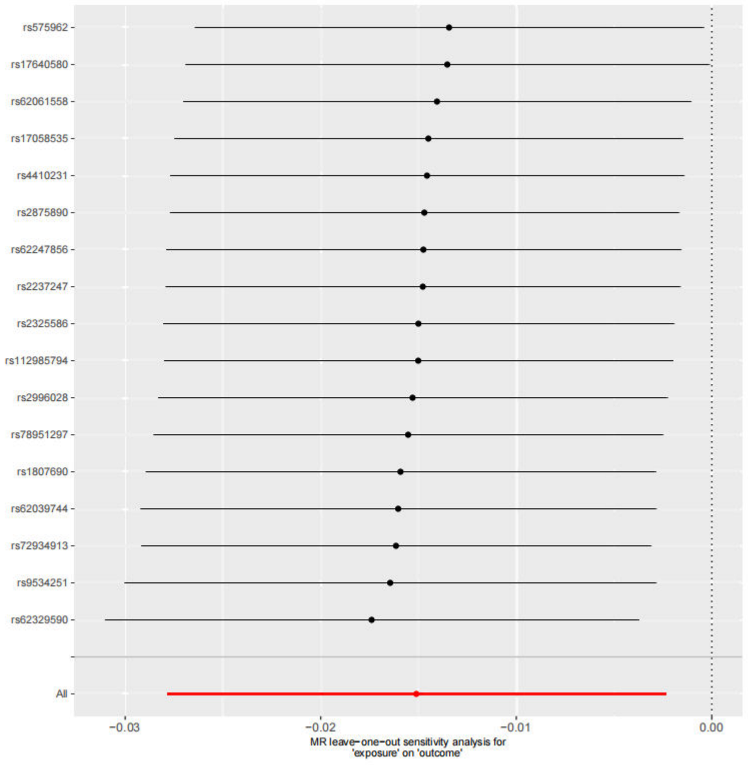
rs575962
rs17640580
rs62061558
rs17058535
rs4410231
rs2875890
rs62247856
rs2237247
rs2325586
rs112985794
rs2996028
rs78951297
rs1807690
rs62039744
rs72934913
rs9534251
rs62329590
All
−0.03
−0.02
−0.01
0.00
MR leave−one−out sensitivity analysis for
'exposure' on 'outcome'

GCST90257105

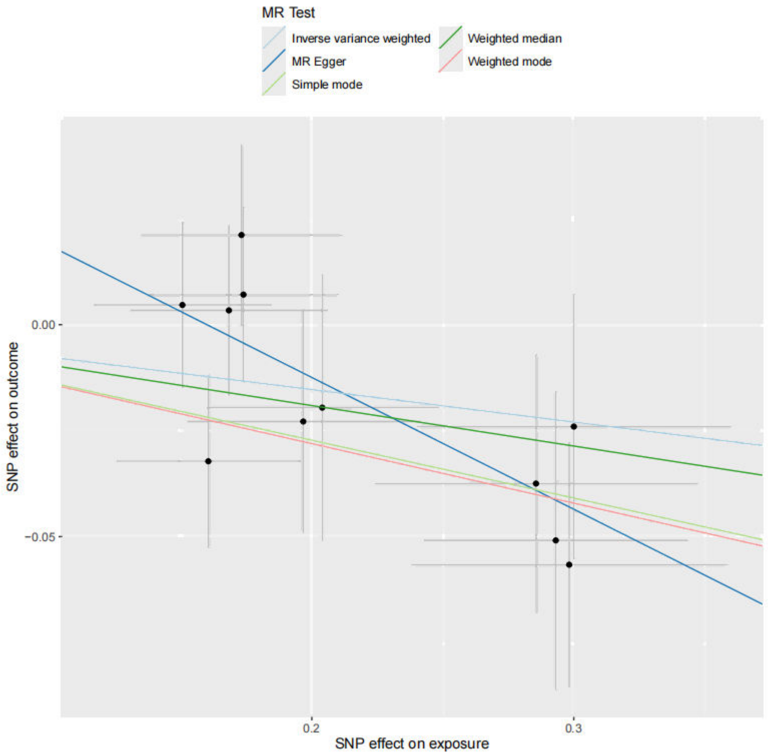

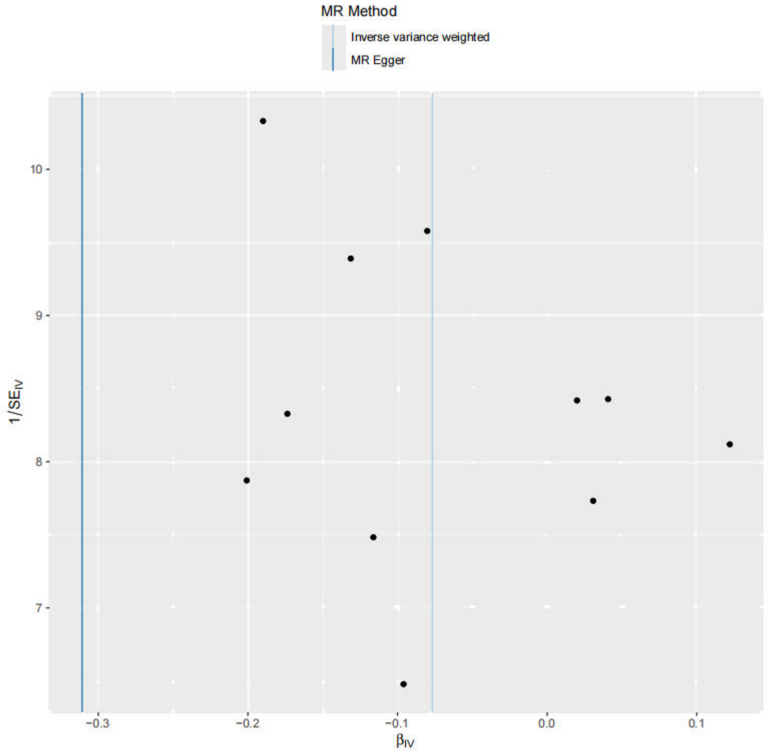

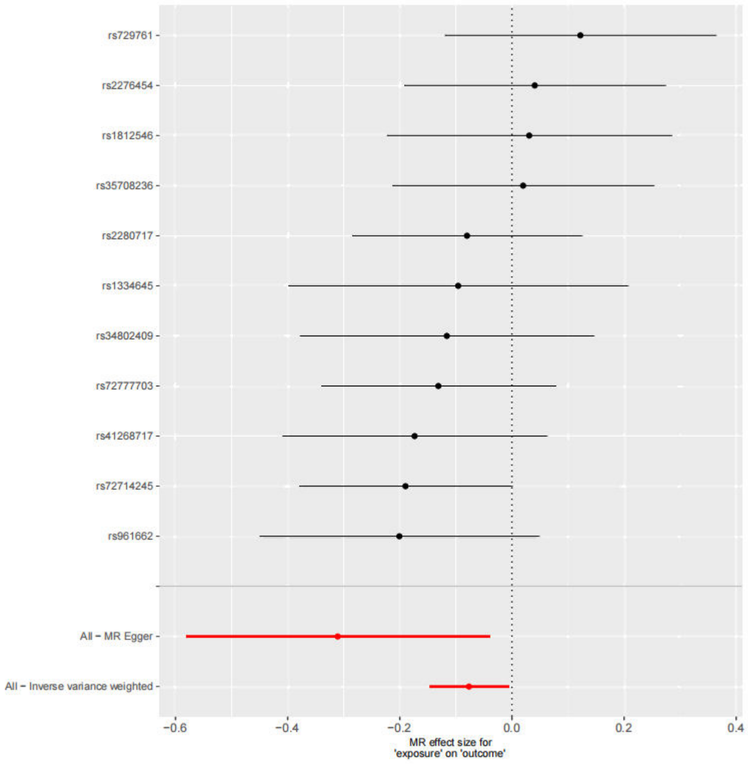
rs729761
rs2276454
rs1812546
rs35708236
rs2280717
rs1334645
rs34802409
rs72777703
rs41268717
rs72714245
rs961662
All − MR Egger
All − Inverse variance weighted
−0.6
−0.4
−0.2
0.0
0.2
0.4
MR effect size for
'exposure' on 'outcome'

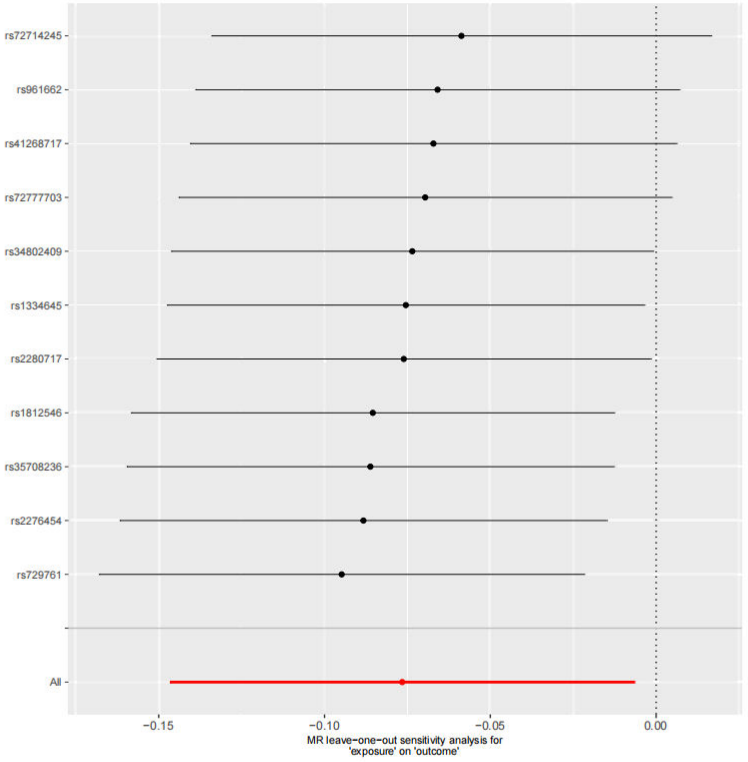
rs72714245
rs961662
rs41268717
rs72777703
rs34802409
rs1334645
rs2280717
rs1812546
rs35708236
rs2276454
rs729761
All
−0.15
−0.10
−0.05
0.00
MR leave−one−out sensitivity analysis for
'exposure' on 'outcome'

GCST90257029

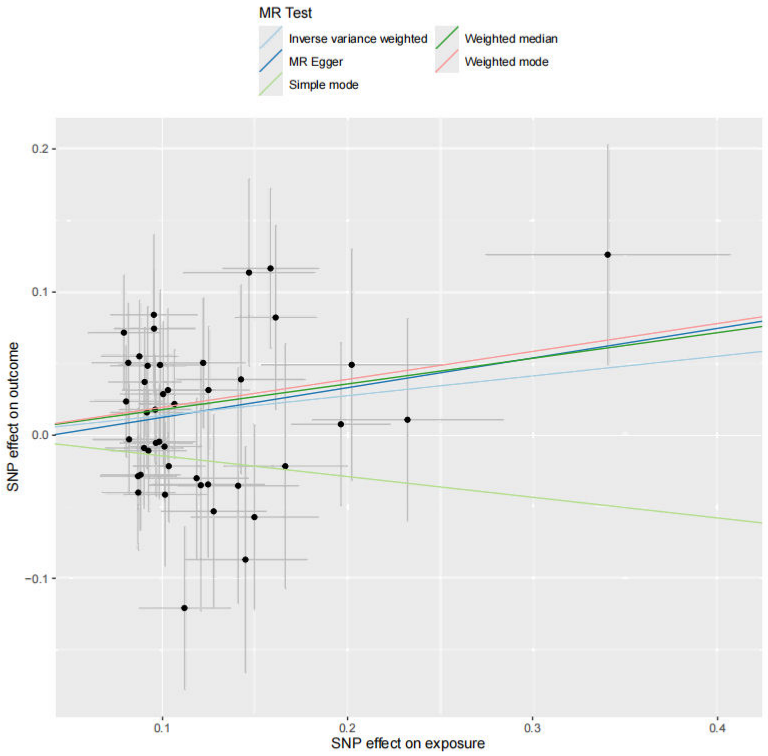

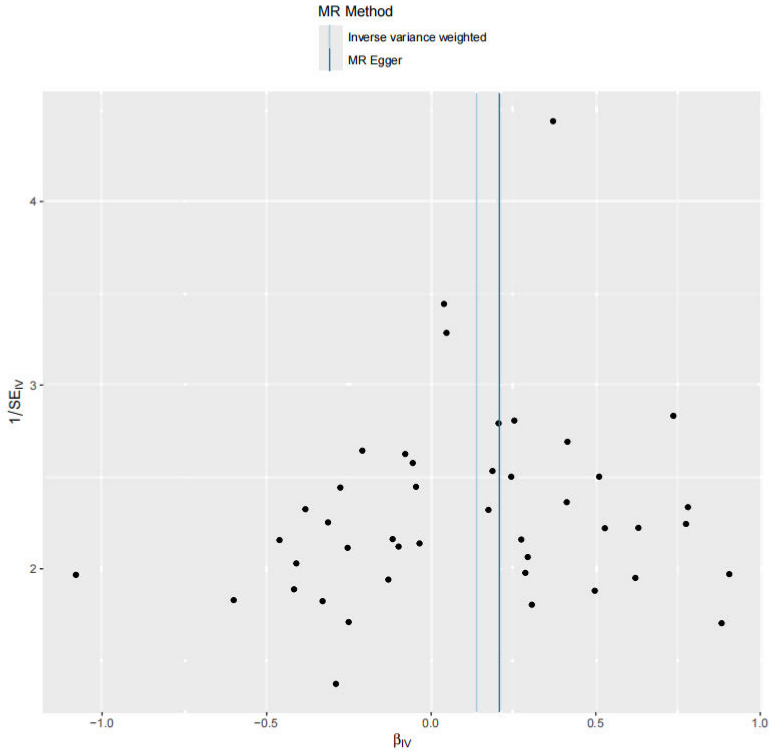

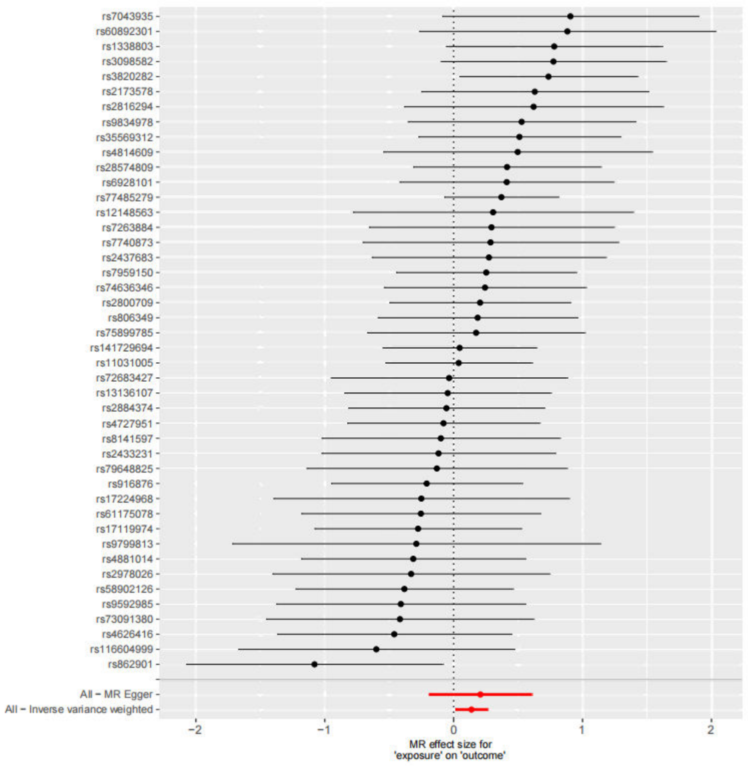
rs7043935
rs60892301
rs1338803
rs3098582
rs3820282
rs2173578
rs2816294
rs9834978
rs35569312
rs4814609
rs28574809
rs6928101
rs77485279
rs12148563
rs7263884
rs7740873
rs2437683
rs7959150
rs74636346
rs2800709
rs806349
rs75899785
rs141729694
rs11031005
rs72683427
rs13136107
rs2884374
rs4727951
rs8141597
rs2433231
rs79648825
rs916876
rs17224968
rs61175078
rs17119974
rs9799813
rs4881014
rs2978026
rs58902126
rs9592985
rs73091380
rs4626416
rs116604999
rs862901
All − MR Egger
All − Inverse variance weighted
−2
−1
0
1
2
MR effect size for
'exposure' on 'outcome'

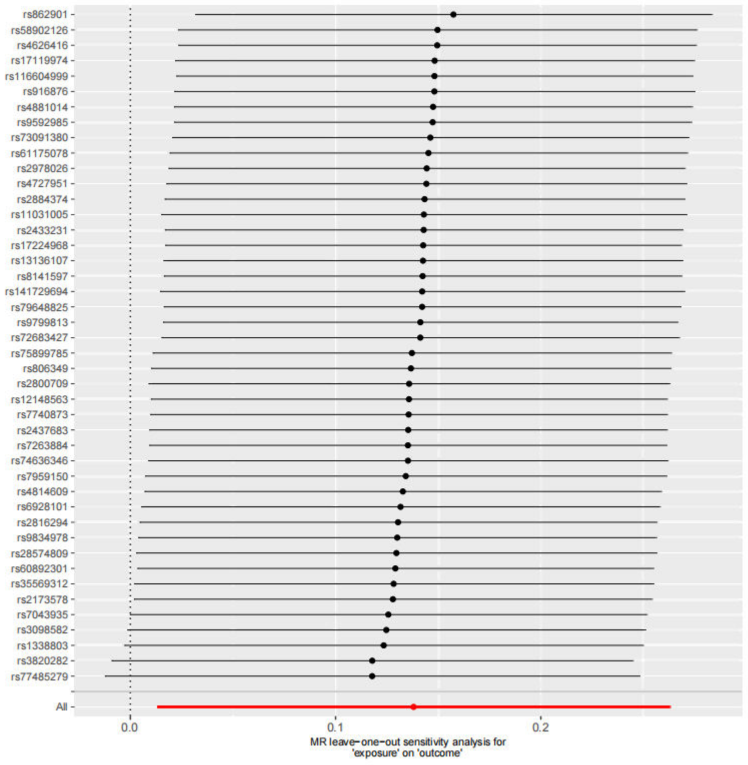
rs862901
rs58902126
rs4626416
rs17119974
rs116604999
rs916876
rs4881014
rs9592985
rs73091380
rs61175078
rs2978026
rs4727951
rs2884374
rs11031005
rs2433231
rs17224968
rs13136107
rs8141597
rs141729694
rs79648825
rs9799813
rs72683427
rs75899785
rs806349
rs2800709
rs12148563
rs7740873
rs2437683
rs7263884
rs74636346
rs7959150
rs4814609
rs6928101
rs2816294
rs9834978
rs28574809
rs60892301
rs35569312
rs2173578
rs7043935
rs3098582
rs1338803
rs3820282
rs77485279
All
0.0
0.1
0.2
MR leave−one−out sensitivity analysis for
'exposure' on 'outcome'

GCST90257070

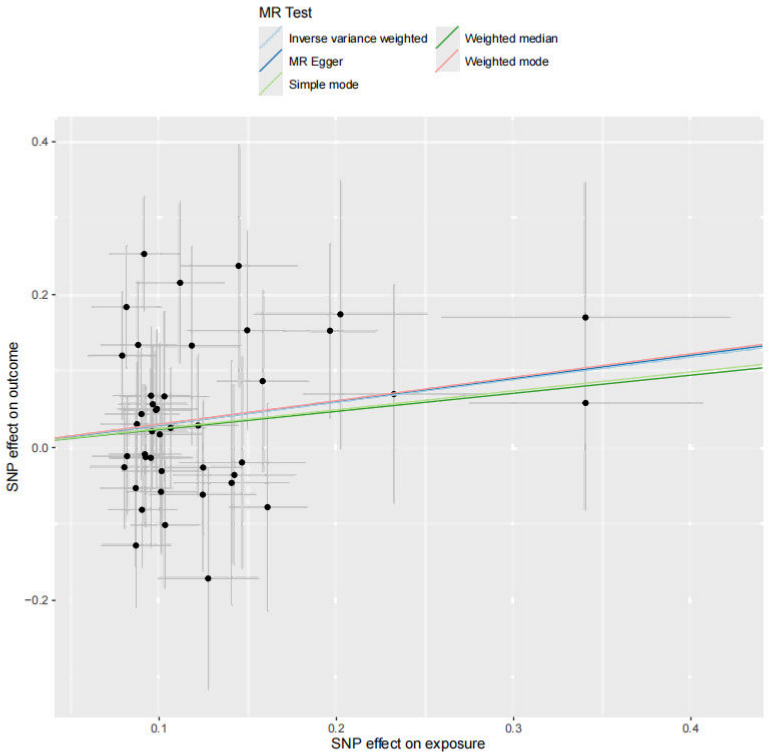

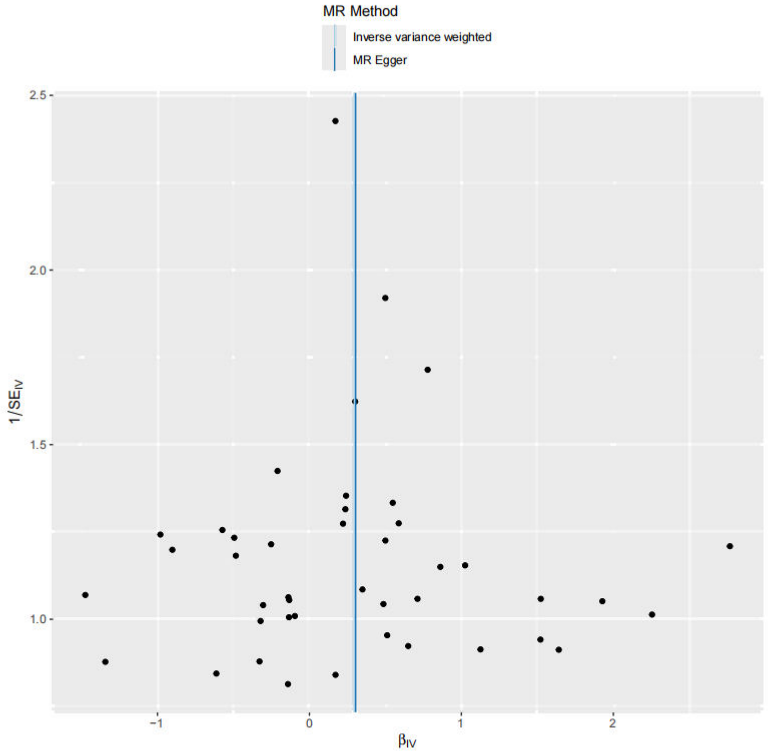

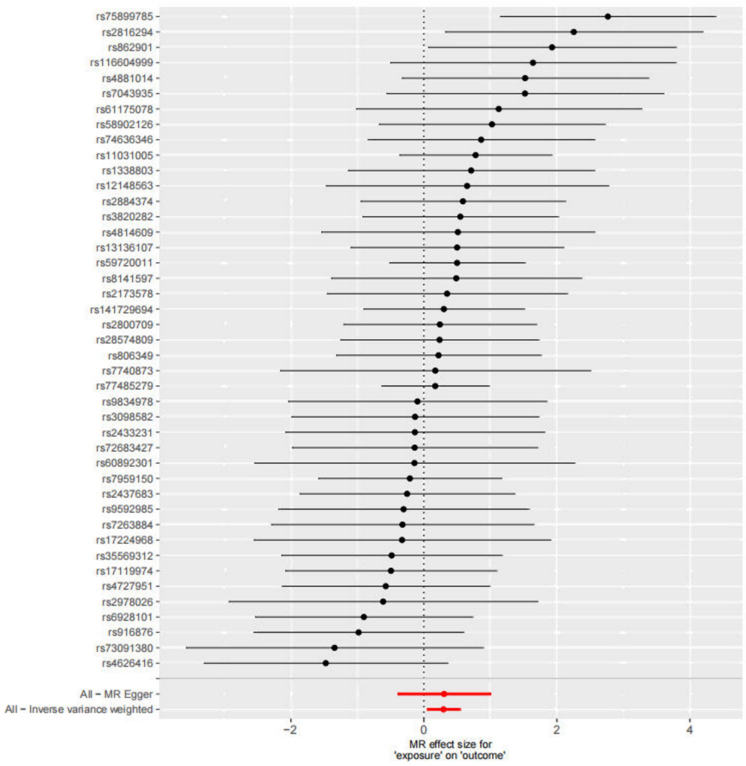
rs75899785
rs2816294
rs862901
rs116604999
rs4881014
rs7043935
rs61175078
rs58902126
rs74636346
rs11031005
rs1338803
rs12148563
rs2884374
rs3820282
rs4814609
rs13136107
rs59720011
rs8141597
rs2173578
rs141729694
rs2800709
rs28574809
rs806349
rs7740873
rs77485279
rs9834978
rs3098582
rs2433231
rs72683427
rs60892301
rs7959150
rs2437683
rs9592985
rs7263884
rs17224968
rs35569312
rs17119974
rs4727951
rs2978026
rs6928101
rs916876
rs73091380
rs4626416
All − MR Egger
All − Inverse variance weighted
−2
0
2
4
MR effect size for
'exposure' on 'outcome'

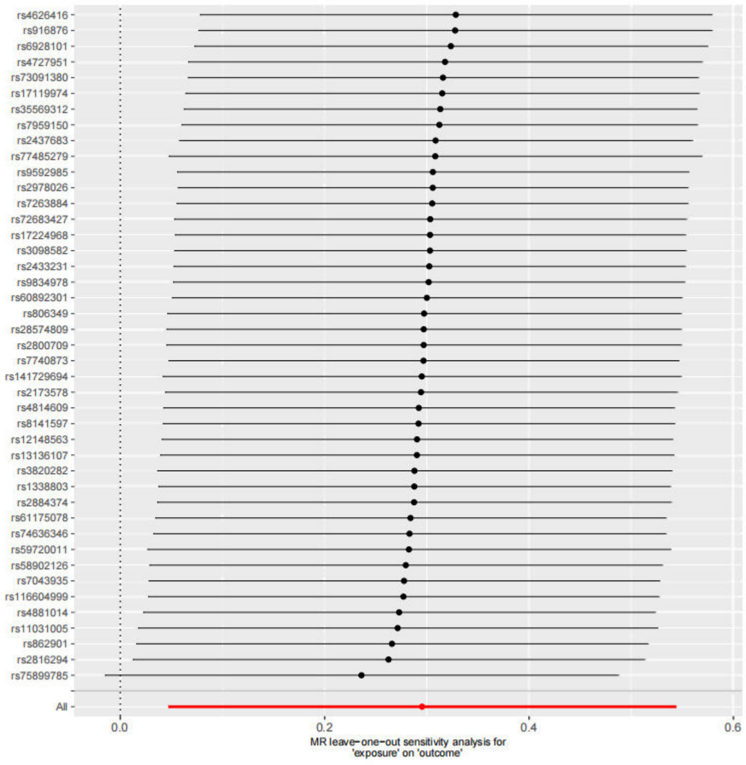
rs4626416
rs916876
rs6928101
rs4727951
rs73091380
rs17119974
rs35569312
rs7959150
rs2437683
rs77485279
rs9592985
rs2978026
rs7263884
rs72683427
rs17224968
rs3098582
rs2433231
rs9834978
rs60892301
rs806349
rs28574809
rs2800709
rs7740873
rs141729694
rs2173578
rs4814609
rs8141597
rs12148563
rs13136107
rs3820282
rs1338803
rs2884374
rs61175078
rs74636346
rs59720011
rs58902126
rs7043935
rs116604999
rs4881014
rs11031005
rs862901
rs2816294
rs75899785
All
0.0
0.2
0.4
0.6
MR leave−one−out sensitivity analysis for
'exposure' on 'outcome'

GCST90257095

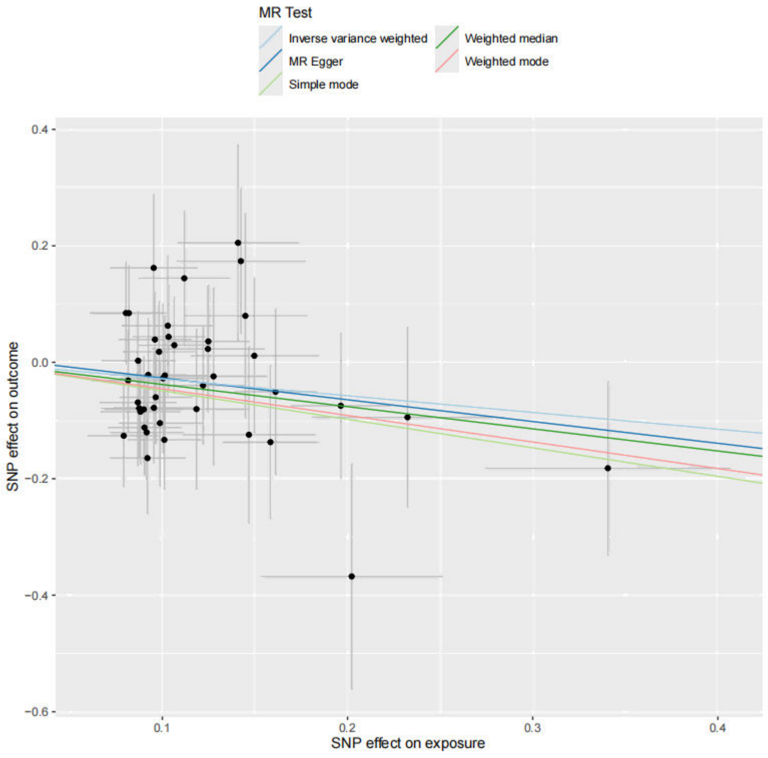

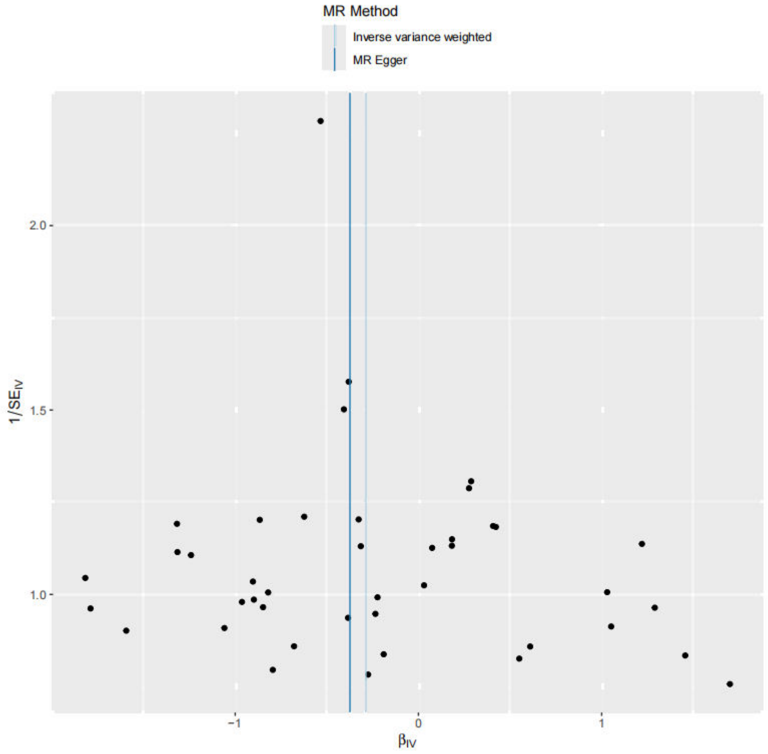

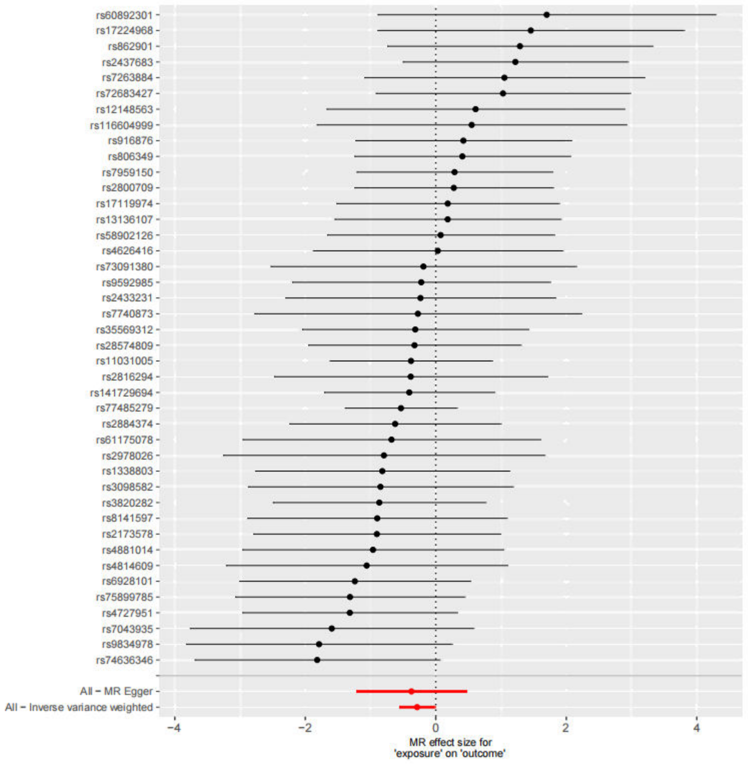
rs60892301
rs17224968
rs862901
rs2437683
rs7263884
rs72683427
rs12148563
rs116604999
rs916876
rs806349
rs7959150
rs2800709
rs17119974
rs13136107
rs58902126
rs4626416
rs73091380
rs9592985
rs2433231
rs7740873
rs35569312
rs28574809
rs11031005
rs2816294
rs141729694
rs77485279
rs2884374
rs61175078
rs2978026
rs1338803
rs3098582
rs3820282
rs8141597
rs2173578
rs4881014
rs4814609
rs6928101
rs75899785
rs4727951
rs7043935
rs9834978
rs74636346
All − MR Egger
All − Inverse variance weighted
−4
−2
0
2
4
MR effect size for
'exposure' on 'outcome'

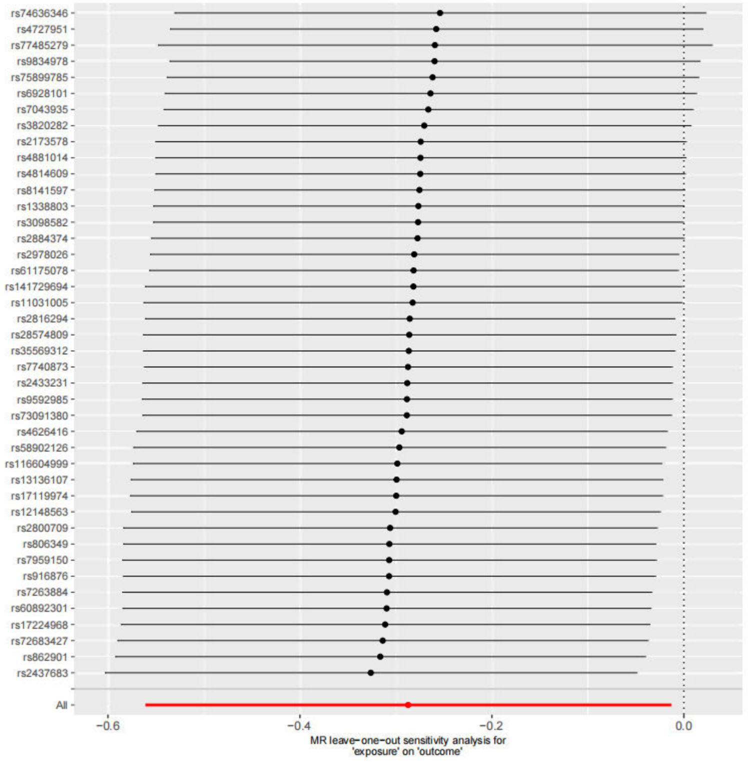
rs74636346
rs4727951
rs77485279
rs9834978
rs75899785
rs6928101
rs7043935
rs3820282
rs2173578
rs4881014
rs4814609
rs8141597
rs1338803
rs3098582
rs2884374
rs2978026
rs61175078
rs141729694
rs11031005
rs2816294
rs28574809
rs35569312
rs7740873
rs2433231
rs9592985
rs73091380
rs4626416
rs58902126
rs116604999
rs13136107
rs17119974
rs12148563
rs2800709
rs806349
rs7959150
rs916876
rs7263884
rs60892301
rs17224968
rs72683427
rs862901
rs2437683
All
−0.6
−0.4
−0.2
0.0
MR leave−one−out sensitivity analysis for
'exposure' on 'outcome'
